# Electrolyte‐Controlled Regiodivergent Continuous Flow Electroselenocyclisations

**DOI:** 10.1002/anie.202509811

**Published:** 2025-08-03

**Authors:** Sagar Arepally, Hanaa Gieman, Thomas Wirth

**Affiliations:** ^1^ School of Chemistry Cardiff University Park Place, Main Building, Cardiff Cymru/Wales CF10 3AT UK

**Keywords:** Alkynes, Continuous flow electrochemistry, Electroselenocyclisation, Isoquinoline and isoindole derivatives, Regioselectivity

## Abstract

Supporting electrolytes, beyond traditionally serving as ionic conductors in electrochemistry, can influence regioselectivity in electrochemical synthesis by acting as base. We present a single‐pass continuous flow electrolysis method enabling selective activation of diselenides and oximes to access seleno‐substituted isoquinoline and isoindole derivatives. Mechanistic studies showed a radical pathway via iminoxyl radicals to isoindole *N*‐oxides and an ionic mechanism leading to isoquinoline *N*‐oxides. This process leverages *N*‐activated neutral isoquinolinium products for diverse downstream modifications. Our findings highlight the untapped potential of supporting electrolytes in regiodivergent electrochemical transformations and provide a sustainable and scalable platform for continuous flow approaches for synthesising selenium‐containing heterocycles with biological relevance.

## Graphical abstract

A single‐pass, continuous‐flow electrolysis strategy – finely tuned by electrolyte choice – unlocks regiodivergent electroselenocyclisations of diselenides and oximes. By selectively guiding radical or ionic pathways through controlled substrate activation, this method enables a sustainable and highly selective synthesis of seleno‐functionalised isoquinoline and isoindole *N*‐oxides, expanding the toolbox for heterocyclic scaffold synthesis in green chemistry.

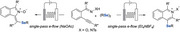

Organic electrochemistry has emerged as a green and sustainable platform for synthetic chemistry, utilising traceless electrons to mediate redox transformations without the need for external chemical reagents. It enables the generation of reactive intermediates and allows precise control over redox processes through electric current or electrode potential, facilitating diverse electrochemical transformations.^[^ ^1^, ^2^, ^3^, ^4^, ^5^, ^6^ ^]^ Despite these advantages, most electrochemical reactions are still conducted in batch, often suffering from limitations such as low productivity, high electrolyte concentrations, and difficulties in scaling up. Recently, single‐pass flow electrolysis has garnered significant interest due to its potential in continuous processing and its importance for industrial applications.^[^ ^7^, ^8^, ^9^, ^10^, ^11^, ^12^ ^]^ Continuous flow microreactors offer distinct benefits over batch systems, including improved mass transfer, shorter residence times, and a higher electrode surface‐to‐volume ratio. These features enhance reaction efficiency, reduce side reactions, and simplify scale‐up through parallel reactor designs, making complex transformations more practical and efficient.^[^ ^13^, ^14^, ^15^, ^16^ ^]^ In this context, the application of single‐pass continuous flow electrochemistry remains underexplored, particularly for the generation of reactive selenium species.^[^ ^17^ ^]^

Traditionally, supporting electrolytes are regarded solely as ionic conductors, facilitating electron transfer and maintaining stable electrochemical conditions.^[^ ^18^ ^]^ Recent advancements in electrochemical synthesis have discovered their dual role as reagents and mediators.^[^ ^19^ ^]^ These electrolytes have gained prominence, particularly in protocols where substrate oxidation occurs as an initial step or is mediated by halide anions to achieve specific reactivity. However, there are only few examples where supporting electrolytes acting as a base in assisting substrate oxidations.^[^ ^20^, ^21^ ^]^ We demonstrate that the choice of electrolyte is pivotal in selectively activating one substrate over another in electrochemical reactions, directly impacting both the reaction outcome and regioselectivity. This highlights their untapped potential as a base in regiodivergent electrochemical synthesis, an area yet to be fully explored.

Given the growing interest in incorporating selenium substituents into organic molecules to enhance the pharmacokinetic profiles of drug candidates (Scheme 1a),^[^ ^22^, ^23^, ^24^, ^25^ ^]^ it is anticipated that the use of diselenides in selenocyclisations will become increasingly important. Despite the progress in selenocyclisations using stoichiometric oxidants and transition metal catalysts to access seleno‐substituted isoquinoline derivatives,^[^ ^26^, ^27^, ^28^, ^29^, ^30^ ^]^ the development of an electrochemical strategy for this transformation remains unaddressed (Scheme 1b).

**Scheme 1:**
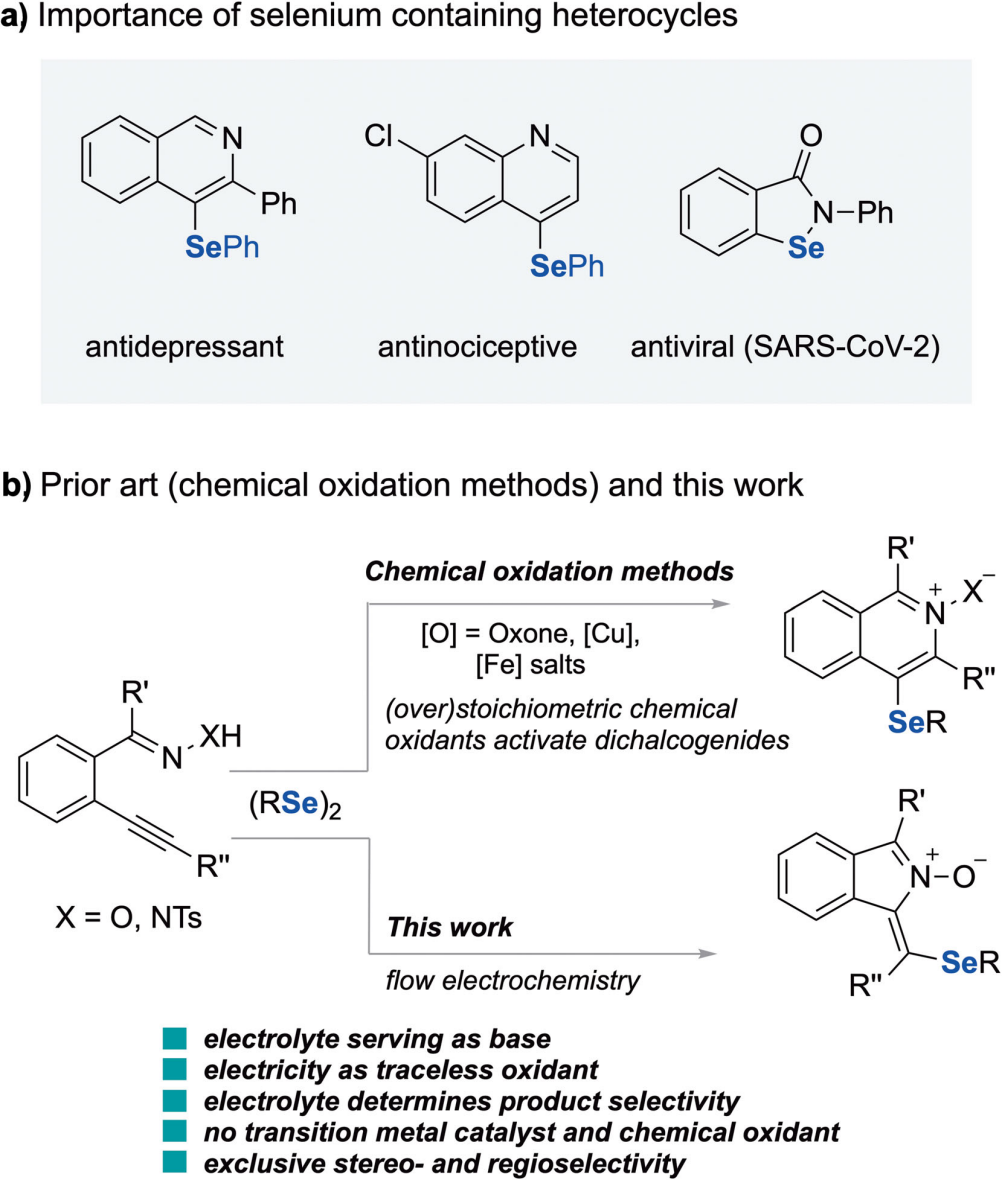
a) Selected examples of selenium‐containing bioactive molecules; b) Overview of prior procedures and present work.

Isoindole derivatives are integral to numerous biologically active molecules and natural products, exhibiting significant pharmaceutical potential.^[^ ^31^ ^]^ Various strategies have been developed for their synthesis, including metal‐catalysed C ─ H activation.^[^ ^32^ ^]^ Isoindole *N*‐oxides are highly versatile intermediates enabling diverse chemical transformations.^[^ ^33^, ^34^ ^]^ The photochemical synthesis of isoindole *N*‐oxides has limitations,^[^ ^35^, ^36^ ^]^ as the method only focuses on 2′‐alkynylacetophenone oximes. The reactions were performed on an analytical scale with the products being characterised without isolation or purification.

The electrochemical selenofunctionalisation of double and triple bonds offers a direct route to seleno‐substituted heterocycles.^[^ ^37^ ^]^ While this strategy has been explored in batch electrolysis, its potential in continuous flow systems remains underdeveloped.^[^ ^38^, ^39^, ^40^ ^]^ Photochemical approaches also generate selenium radicals under mild, oxidant‐free conditions by homolytic Se─Se bond cleavage using visible light, offering an alternative pathway to these transformations.^[^ ^41^ ^]^ We present here an electrolyte‐directed synthesis of seleno‐substituted isoquinoline and isoindole derivatives and demonstrate the synthetic versatility of *N*‐activated isoquinolinium species by leveraging the N─O or N─N bond as a platform for further chemical modifications.

*Ortho*‐alkynylaryl oxime **1** and diphenyl diselenide **2** were selected as model substrates to explore this approach. The optimisation experiments were conducted in an undivided electrochemical flow cell using a graphite anode and a nickel cathode. Key findings are summarised in Table 1. Notably, the highest yield (92%) of the 6‐*endo* selenocyclisation product, 1‐methyl‐3‐phenyl‐4‐(phenylselanyl)isoquinoline 2‐oxide **3**, was obtained using Et_4_NBF_4_ (1 equiv.) as the supporting electrolyte at room temperature under potentiostatic conditions at 22 mA (6.8 F mol^−1^) in a solvent mixture of MeCN and HFIP (5:1). The reaction was conducted with a residence time of 12 min at a flow rate of 0.05 mL min^−1^ in a single pass, undivided electrochemical microfluidic cell with an interelectrode distance of 0.5 mm (Table 1, entry 1).^[^ ^42^ ^]^ The choice of electrode materials, solvents, electrolytes and charge had a significant impact on the reaction outcome, as detailed in Supporting Information (Section 2.2). For example, a decrease in charge led to incomplete conversion of starting material with 63% yield (Table 1, entry 2), and replacing the nickel cathode with a graphite or platinum cathode yielded product **3** in only 77% or 79% yield, respectively (Table 1, entries 3 and 4). During electrolyte screening, most electrolytes such as *n*Bu_4_NBF_4_, LiClO_4_, *n*Bu_4_NPF_6_ and *n*Bu_4_NHSO_4_ demonstrated lower efficiency than Et_4_NBF_4_ (see Supporting Information). However, the use of NaOAc led to an exclusive formation of isoindole *N*‐oxide **4** as a regioisomer of **3**. Due to the limited solubility of NaOAc in MeCN/HFIP, the reaction was initially performed in batch electrolysis, achieving 55% yield (Table 1, entry 5). In the continuous flow setup, water (1.25 M NaOAc aq. solution) was added to ensure complete solubility. Even under these modified conditions, isoindole *N*‐oxide **4** was obtained in 43% yield, alongside some unidentifiable byproducts. The yield was improved through further optimisation (Table 1, entry 6) and revealed that the use of methanol in the solvent system improved the solubility of NaOAc and afforded **4** in 88% yield in MeCN/MeOH (1:1) (Table 1, entry 7). Only NaOAc and Bu_4_NOAc produced isoindole *N*‐oxide **4** exclusively and we assume that the counterion acts as a mediator influencing the electrochemical environment by facilitating oxime deprotonation, thereby promoting the 5‐*endo* cyclisation (Table 1, entries 7 and 8). A mixture of Et_4_NBF_4_ and NaOAc produced both products **3** and **4**, highlighting the role of electrolytes in determining product selectivity (Table 1, entry 10). Additional competitive experiments were conducted using the oximes leading to products **5**, **8**, **40** and **44** under the reaction conditions used in Table 1, entry 10. The 4‐methoxy oxime afforded both products, **5** and **40** in 15% and 9% yield while the 4‐fluoro oxime gave isoindole *N*‐oxide **44** (38%) with only trace amounts of isoquinoline *N*‐oxide **8** (<2%), respectively. These results further support the significant role of the electrolyte in controlling product selectivity.

**Table 1:** Optimisation of reaction conditions for electroselenocyclisations.^a)^

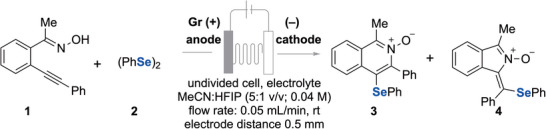						
Entry	Electrolyte (equiv.)	Charge (F mol^−1^)	Cathode	Conver‐sion (%)^b)^	Yield 3 (%)^b)^	Yield 4 (%)^b)^
1	Et_4_NBF_4_ (1)	6.8	Ni	>99	92 (85)^c)^	<2
2	Et_4_NBF_4_ (1)	4.7	Ni	64	63	<2
3	Et_4_NBF_4_ (1)	6.8	Pt	>99	77	<2
4	Et_4_NBF_4_ (1)	6.8	Gr	>99	79	<2
5	NaOAc (1)^e)^	6.8	Ni	85, >99^d)^	<2	43, 55^d)^
6	NaOAc (2)^f)^	6.8	Ni	78	<2	76 (74)^c)^
7	NaOAc (2)^g)^	8.9	Pt	>99	<2	88 (87)^c)^
8	*n*Bu_4_NOAc (2)^g)^ 8.9		Pt	94	<2	58
9	NaOAc (1) + 6.8 *n*Bu_4_NOAc (1)^g^ ^)_^		Pt	90	<2	77
10	NaOAc (1) + Et_4_NBF_4_ (1)^g)^	6.8	Pt	84	10	34
11	–	6.8	Ni	89	3	34

^a)^ For experimental details, see the Supporting Information.

^b)^ *Z/E* = 50:1 for **4** (major isomer assigned as Z) and yields determined by ^1^H NMR using CH_2_Br_2_ as internal standard.

^c)^ Isolated yield in parentheses.

^d)^ Reaction performed under batch electrolysis conditions.

^e)^ NaOAc was dis‐solved in H_2_O (1.25 M) for the flow electrochemical reaction.

^f)^ NaOAc was dis‐solved in 0.2 mL of MeOH (1.25 M).

^g)^ Solvent: MeCN:MeOH (1:1 v/v, 0.04 M).

For the formation of isoindole *N*‐oxide **4**, we observed that lower charges resulted in incomplete conversion of the starting material. Increasing the charge led to complete conversion with improved yields. Therefore, a higher charge (8.9 F mol^−1^) was used for isoindole substrates compared to isoquinoline substrates (6.8 F mol^−1^), based on their different reactivity profiles under electrochemical conditions. Detailed reaction optimisation for isoindole *N*‐oxide is available in Supporting Information (Section 2.2). Control experiments confirmed that electricity is indispensable for both transformations. In the absence of an added electrolyte, the reaction still proceeds but with reduced efficiency along with unidentifiable by‐products (Table 1, entry 11).

Having identified the two sets of optimised reaction conditions, initially the scope of the isoquinoline synthesis was explored (Scheme 2). The reaction of substrates **1** and **2** provided the target product **3** (85% yield) with exclusive regioselectivity, determined by NMR spectroscopic analysis. The electronic structure of the aryl substituent on the aryne does not affect the cyclisation (products **3**–**10**). Different diselenides were also employed efficiently (products **12**–**18**). The use of a TMS‐substituted *ortho*‐alkynylaryl ketoximes formed the TMS‐free isoquinoline *N*‐oxides (**11**, **15**, **24**) in 39%–54% yield, respectively. An electrogenerated base facilitates TMS deprotection in the presence of a protic solvent. This deprotection may occur even in the starting material before its reaction with selenium species, causing lower yields. Substituents on the aromatic moiety in the diselenides showed no significant impact on the reaction, as similar yields were observed for different substituted diaryl diselenides (**16**–**18**). Encouraged by the robustness of this method, the generality of more challenging *ortho*‐alkynylaryl aldoximes (R’ = H) was explored, as aromatic aldoximes are typically oxidised to nitrile oxides or nitriles under electrochemical conditions.^[^ ^43^ ^]^ Remarkably, substrates with electron‐donating groups on the aryl alkyne (**20, 25**), thienyl substituents (**21**, **22**) and substituted diaryl diselenides (**23**) produced the desired products in good yields with regiocontrol under these conditions. Notably, this class of isoquinolinium *N*‐oxides is particularly valuable, as offering ample opportunity for further transformations at the C1 position (see Scheme 7). Good performance was also achieved using diphenyl ditelluride as a reagent, yielding isoquinoline *N*‐oxides (**26**, **27**) in 48% and 83% yield, respectively. In addition to the desired products, a non‐isolable *N*‐oxide product lacking the tellurium group was also detected.

**Scheme 2:**
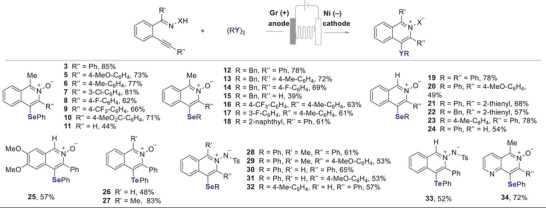
Scope for isoquinoline formation. Reactions carried out on a 0.4 mmol scale. 6.8 F mol^−1^, 22 mA, Et_4_NBF_4_ (1 equiv.), MeCN:HFIP (5:1 v/v; 0.04 M), rt, interelectrode distance 0.5 mm, flow rate 0.05 mL/min, undivided cell (reaction conditions A). For procedure details, see the Supporting Information. MeCN: HFIP:DCE (1:1:0.2, 0.04 M) was used for the synthesis of **26** – **33**. All products were obtained with a regioselectivity ratio of >50:1.

As a second class of substrates, *ortho*‐alkynylaryl hydrazides, were used under slightly modified electrochemical conditions, yielding isoquinolinium imides (**28**–**32**) in lower yields due to incomplete conversion and the formation of unidentifiable products, likely resulting from the sensitivity of tosylhydrazides to the basic conditions generated during electrolysis.^[^ ^44^ ^]^ The structure of compound **28** was further proven by X‐ray analysis.^[^ ^45^ ^]^ In contrast, no reaction occurred with diphenyl disulfide, probably due to the stronger S─S bond compared to Se─Se and Te─Te bonds, preventing the formation of electrophilic sulphur species under the given electrochemical conditions.

Moreover, *ortho*‐alkynylaryl hydrazides derived from aldehydes were also compatible for this tandem annulation reactions (R’ = H), giving the desired products (**30**–**32**). *Ortho*‐alkynylaryl hydrazides and aldoximes reacted inefficiently with diphenyl ditellurides, yielding moderate reaction efficiencies and incomplete conversion of the starting materials (**27**, **33**). The heteroaryl ketoxime gave the desired isoquinoline *N*‐oxide **34** smoothly, while the alkyne‐tethered aliphatic oxime was fully consumed but led to a complex mixture with no detectable product (for unsuccessful substrates see Supporting Information). We then investigated the applicability to alkenes and used *ortho*‐alkenylaryl hydrazide **35a** under standard reaction conditions (Scheme 3). While the fully oxidised product **30** was obtained, it was produced in only 25% yield and accompanied by several unknown byproducts with complete conversion of **35a**. Unfortunately, propargyl ester **35b** (R″ = CH_2_‐OCOPh) was unreactive under the reaction conditions.

**Scheme 3:**
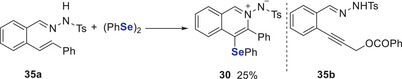
Cyclisation of *ortho*‐alkenylaryl hydrazide **35a**.

The generality of isoindole *N*‐oxide formation was investigated (Scheme 4). Many aryl‐substituted compounds were converted smoothly to afford the corresponding isoindole *N*‐oxides with excellent regio‐ and stereoselectivity (**4**, **36**−**48**). The structure of product **36** was further confirmed through X‐ray analysis.^[^ ^45^ ^]^ Even an electron‐rich derivative cyclised to product **46** in 60% yield under modified electrochemical reaction conditions. Isoindole *N*‐oxide **48** was obtained from the heteroaryl ketoxime in 51% yield. Flow electrolysis of *ortho*‐alkenylaryl ketoximes with dibenzyl diselenide was carried out at a low constant current of 10 mA (3.1 F mol^−1^) to improve the yields of products **38** and **41**, as optimised conditions at constant current initially gave poor yields (<20%) along with unidentifiable byproducts with complete conversion of its starting material. A modified solvent system consisting of MeCN:MeOH:HFIP (5:0.5:1 v/v/v; 0.04 M) was then employed to further improve the yields of **40** and **41**. The addition of HFIP resulted in clean reaction profiles for these transformations. The products **42**, **45** and **46** resulted in mixtures of separable stereoisomers, with the minor isomer formed in around 10% yield, but the stereochemistry of the main product could not be determined. However, for most substrates, only a single isomer was obtained exclusively. The origin of this stereochemical outcome is not yet fully understood. Computational studies, along with KIE experiments and time‐resolved techniques (e.g., in situ NMR or online MS), are planned as part of our future work to better understand the mechanism and selectivity. Ortho‐alkenylaryl aldoximes and tosylhydrazides were not converted to isoindole derivatives, as they decomposed into complex mixtures during electrolysis. As mentioned earlier, aldoximes **49a** are generally challenging substrates,^[^ ^43^ ^]^ and tosylhydrazides **49b** are sensitive under basic conditions, prone to side reactions.^[^ ^44^ ^]^ Additionally, the alkyne‐tethered aliphatic alkyl oxime **49c** showed poor reactivity under condition B, with only ∼24% conversion and no desired product detected. Disulfides and ditellurides were non‐productive under conditions B, leading to complex reaction mixtures with no desired product detected.

**Scheme 4:**
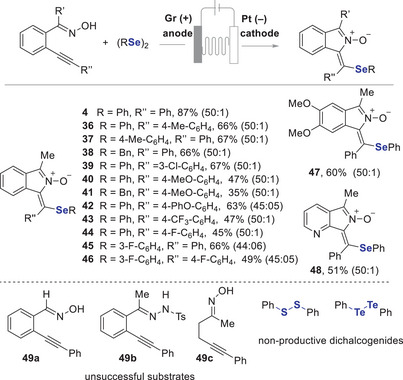
Synthesis of isoindole *N*‐oxides from *ortho*‐alkynylaryl ketoximes. Reactions carried out on a 0.4 mmol scale. 8.9 F mol^−1^, 27 mA, NaOAc (2 equiv), MeCN:MeOH (1:1 v/v; 0.04 M), rt, interelectrode distance 0.5 mm, flow rate 0.05 mL min^−1^, undivided cell (reaction conditions B). Stereoselectivity in parentheses. All products were obtained with a regioselectivity ratio of >50:1. For experimental details, see Supporting Information.

To understand the mechanism of this regiodivergent electrosynthesis, a reaction of **1** with phenylselenyl bromide was carried out under standard reaction conditions B, and the 6‐membered isoquinoline *N*‐oxide **3** was obtained in 97% yield. Compound **3** was also obtained in 84% yield in a reaction without electricity (Scheme 5a). Further, we carried out radical inhibition experiments under conditions A, product **3** was formed in both cases. In the presence of BHT, product **3** was obtained in 63% yield. Notably, when TEMPO was used, both product **3** (32%) and product **4** (18%) were formed (Scheme 5b). For the formation of product **4**, we believe that TEMPO may function not only as a radical scavenger but also as a redox mediator facilitating oxime activation, which consistent with literature report.^[^ ^20^ ^]^

**Scheme 5:**
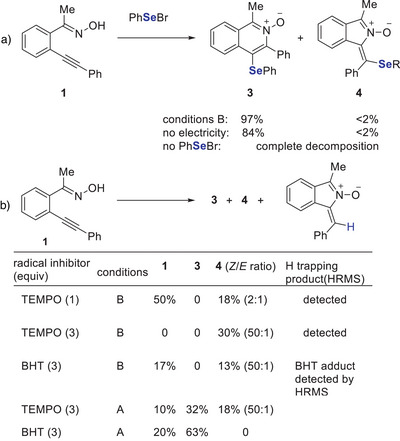
Supporting mechanistic experiments.

These findings support that the reaction may proceed via an ionic pathway that furnishes isoquinoline *N*‐oxide **3**. We carried out similar trapping experiments under conditions B. When TEMPO (1 equiv.) was used, we observed a reduced yield of product **4** and low conversion of the starting material. In contrast, other trapping agent (BHT) and TEMPO (3 equiv.) showed high conversion, suggesting that these scavengers partially suppress the cyclisation process. High‐resolution mass spectrometry (HRMS) analysis of reaction mixtures (see Supporting Information) confirmed the formation of radical adducts, including isoindole *N*‐oxide bearing a 3,5‐di‐*tert*‐butyl‐4‐hydroxyphenyl substituent (from BHT) and 1‐benzylidene‐3‐methyl‐1*H*‐isoindole 2‐oxide. These experiments suggest that the reaction involved a radical induced cyclisation to afford isoindole *N*‐oxide **4**.

Cyclic voltammetry (CV) experiments were conducted to further elucidate the reaction mechanism under both reaction conditions A and B (Figure 1). Under conditions A, diphenyl diselenide exhibited a sharper and steeper anodic curve, indicating a faster and more pronounced oxidation. A similar electrochemical behaviour was observed for the mixture of (PhSe)_2_ and oxime **1**, with enhanced current. The broader anodic wave associated with oxime **1** suggests a slower and less efficient oxidation compared to (PhSe)_2_, as evidenced by the lower current response (Figure 1a). These results suggest that (PhSe)_2_ undergoes consecutive oxidations to produce electrophilic selenium species, which subsequently activate the alkyne functionality, leading to the formation of the selenirenium cation **I** (Scheme 6a). It has been reported that electrochemical oxidation of diselenides such as (PhSe)_2_ generates reactive selenium cation species (PhSe⁺), which can efficiently activate alkynes through electrophilic addition, facilitating downstream transformations.^[^ ^46^ ^]^

**Figure 1:**
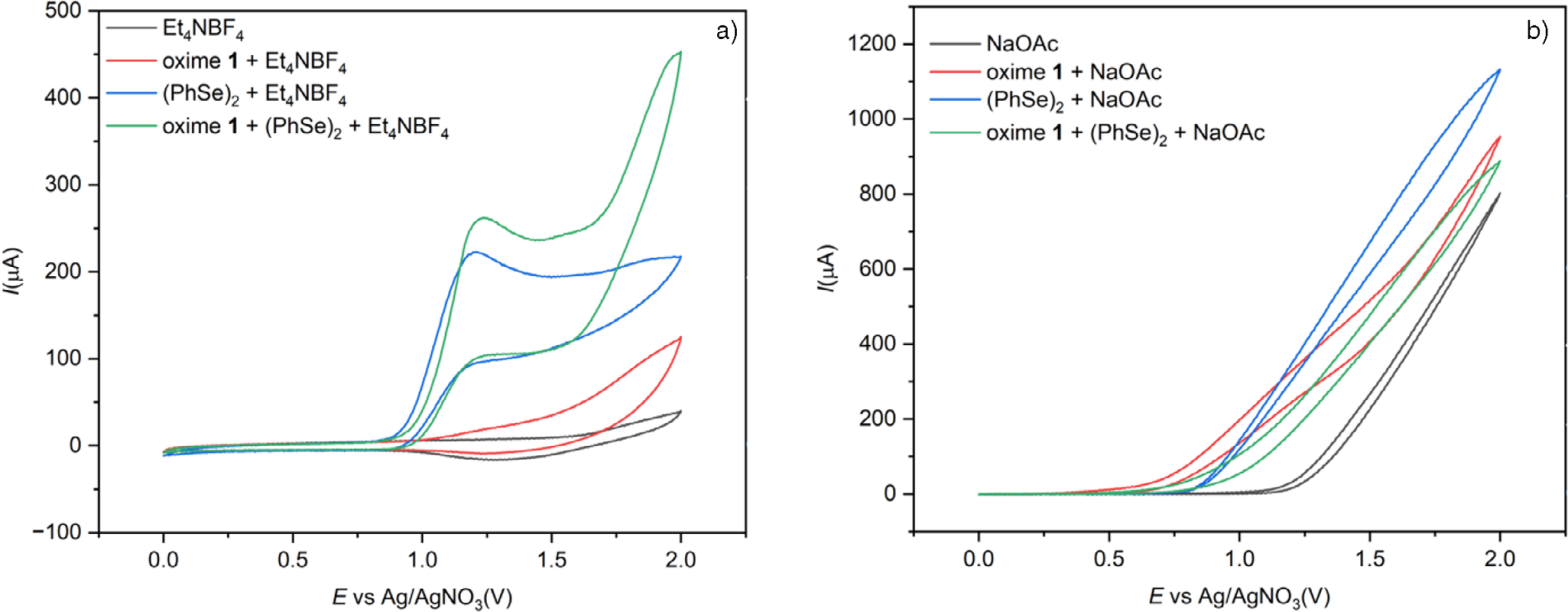
Cyclic voltammetry studies of **1** and (PhSe)_2_ with different electrolytes.

**Scheme 6:**
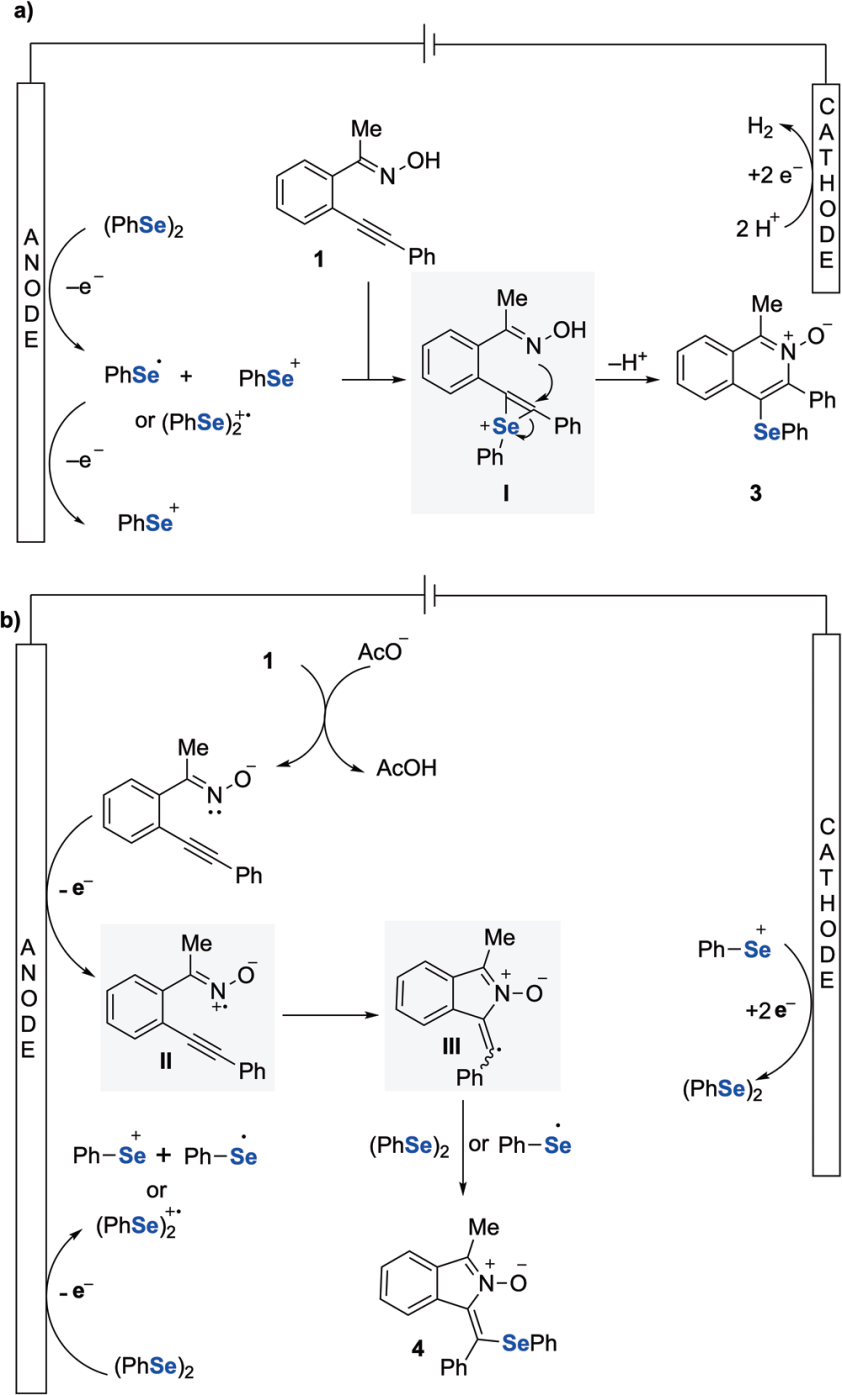
a) Proposed mechanism for isoquinoline *N*‐oxide formation. b) Proposed mechanism for isoindol *N*‐oxide formation.

Under conditions B, the supporting electrolyte NaOAc itself exhibited a significant oxidation peak, demonstrating its electrochemical activity. Notably, both (PhSe)_2_ and oxime **1** displayed distinct oxidation curves, with onset oxidation potentials of 0.78  and 0.52 V, respectively (Figure 1b).

The lower onset oxidation potential and higher current observed for oxime **1** are likely due to the involvement of the NaOAc electrolyte. Preliminary rotating disk electrode experiments suggest kinetically limited oxidation of oxime species; further experiments are needed to confirm this, which we aim to pursue in future studies. In contrast, when using Et_4_NBF_4_ as the supporting electrolyte, oxime **1** exhibited an onset oxidation potential of 0.89 V, with minimal oxidation currents (Figure 1a). These results suggest that acetate facilitates the deprotonation of oxime **1**, generating a more reactive intermediate anion that is easier to oxidise (Scheme 6b). The resulting intermediate **II** undergoes radical cyclisation to form intermediate **III**. Further radical trapping experiments indicated that the oxidised intermediate **III**, derived from oxime **1**, was trapped by BHT and a hydrogen radical (see Supporting Information). Radical intermediate **III** is then trapped by (PhSe)_2_ or proceeds with a radical coupling with PhSe^•^ to generate the isoindole *N*‐oxide **4**. Selenium cation species generated during anodic oxidation may be reduced at the cathode to regenerate (PhSe)_2_ (Scheme 6b), as reported previously.^[^ ^46^ ^]^ They may also react with nucleophiles like acetate (AcO^−^), although we did not observe any such side products. These possibilities highlight additional redox events that may occur under electrochemical conditions.

To highlight the synthetic utility and scalability of the reaction, a gram‐scale synthesis and several valuable transformations of *N*‐activated isoquinoline derivatives were performed (Scheme 7). The scale‐up of this regiodivergent reaction proceeded smoothly, affording products **19** and **30** in good yields, with corresponding productivities of 0.091  and 0.074 mmol h^−1^, respectively (see Supporting Information). Faradaic efficiencies for products **3** and **4** under optimised conditions were 33% and 42%, respectively. Heterocyclic *N*‐oxides have gained significant attention due to their distinctive biological activities and their role as key building blocks in many bioactive natural products and commercially available drugs.^[^ ^47^ ^]^ Importantly, the introduction of an *N*‐oxide functionality dramatically alters the reactivity of the parent heterocycle, enabling it to participate in a wide range of chemical transformations.^[^ ^48^, ^49^ ^]^ The isoquinoline framework represents a vital structural motif commonly found in natural products.^[^ ^50^, ^51^ ^]^ However, the synthesis of substituted 1‐aminoisoquinolines often faces significant challenges, including complex procedures, harsh reaction conditions and the use of expensive starting materials, which limit their structural diversity.^[^ ^52^, ^53^, ^54^ ^]^ Our protocol addresses these issues by offering a straightforward, efficient and metal‐free approach for the synthesis of *Se*‐substituted 1‐aminoisoquinoline **50**, thereby providing a practical and versatile route for constructing these valuable compounds. In addition, we have utilised Se‐modified isoquinoline *N*‐oxide **19** in a Petasis‐type reaction to **51**, for phosphorylation to **52** and in cycloadditions leading to **53** to synthesise valuable isoquinoline derivatives. Also, the *N*‐activated isoquinolinium imide **30** was used in cycloadditions, enabling the formation of valuable complex heterocyclic frameworks (**54**, **55**). These transformations would not be achievable using the parent heterocycles.

**Scheme 7:**
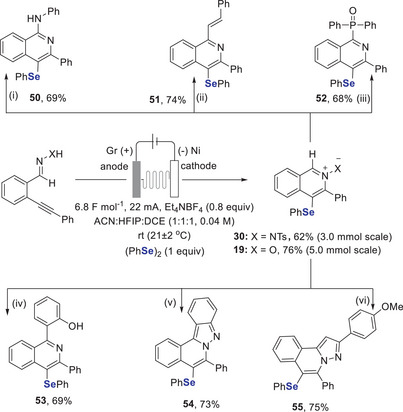
Reaction scale‐up and synthetic utility of isoquinoline *N*‐oxides and isoquinolinium imides. i) AgBF_4_ (15 mol%), dry DMF (0.25 M), 50 °C,16 h, ii) NMP (0.3 M), 130 °C, 6 h, iii) AgOTf (10 mol%), 4 Å MS (50 mg), 1,4‐dioxane (0.1 M), 40 °C,12 h, iv) TBAF (1.5 equiv), DCM (0.25 M), 30 °C,12 h, v) TBAF (1.5 equiv), CH_2_Cl_2_ (0.25 M), 30 °C,12 h, vi) DBU (2.5 equiv), AgOTf (10 mol%), CCl_4_ (0.1 M), 25 °C,10 h.

In summary, we have developed an electrolyte‐controlled strategy to dictate regioselectivity and reaction outcomes in electrochemical transformations through selective substrate activation. Utilising single‐pass continuous flow electrolysis, we succeeded with a highly selective electrochemical activation of diselenides and oximes, leading to the efficient synthesis of seleno‐substituted isoquinolines and isoindoles. Mechanistic studies, including radical trapping experiments and CV, revealed a radical pathway involving iminoxyl radicals for the synthesis of isoindole *N*‐oxides and an ionic pathway for isoquinoline *N*‐oxides. Furthermore, we demonstrated the synthetic utility of *N*‐activated neutral isoquinolinium derivatives in site‐selective functionalisations, enabling the introduction of diverse functional groups at distinct positions that are otherwise difficult to accomplish from the parent heterocycles.

## Supporting Information

The authors have cited additional references within the Supporting Information.^[^ ^30^, ^55^, ^56^, ^57^, ^58^, ^59^, ^60^, ^61^, ^62^, ^63^, ^64^, ^65^, ^66^, ^67^, ^68^, ^69^, ^70^, ^71^, ^72^, ^73^, ^74^, ^75^, ^76^ ^]^

## Conflict of Interests

The authors declare no conflict of interest.

## Supporting information



Supporting Information

## Data Availability

The data that support the findings of this study are available in the Supporting Information of this article.
