# Supplementary material for: Electrolyte‐Controlled Regiodivergent Continuous Flow Electroselenocyclisations
Source: Angew Chem Int Ed Engl. 2025 Aug 3;64(38):e202509811. doi: 10.1002/anie.202509811 (PMC12435410; doi:10.1002/anie.202509811)

Supporting Information

Contents

1. General information……S2
1.1. Reagents, solvents and experimental conditions...... S2
1.2. Analytical techniques S2
1.3. Compound purification S2
1.4. Electrochemical setup S3
2. Experimental details……S4
2.1. Details for the synthesis of all starting materials......S4-9
2.2. Flow electrolysis optimization details S9-11
2.3. General electrolysis procedures for synthesis of isoquinoline/isoindole derivativesS11-14
2.4. Mechanistic experiments......S15-16
2.5. Cyclic voltammetry experiments and rotating disk electrode (RDE) studies......S16-19
2.6. Characterization data for starting materials S20-28
2.7. Characterization data for isoquinoline *N*-oxide/imide derivatives S28-40
2.8. Characterization data for isoindole *N*-oxide derivatives S40-46
2.9. Experimental procedure and characterization data for synthetic applications S46-50
3. References……S51
4. NMR Spectra……S52-169

1. General information

1.1. Reagents, solvents and experimental conditions

All reagents were purchased from Alfa Aesar, Sigma-Aldrich, Fluorochem, Acros Organics, Fisher Scientific and used without further purification, except otherwise stated. Dry solvents such THF, toluene and acetonitrile were obtained after passing these previously degassed solvents through activated alumina columns (MBraun, SPS-800). 1,2 dichloroethane solvent used without further purification. Reactions involving air and moisture sensitive reagents were carried out in oven-dried glassware under an atmosphere of argon using standard Schlenk technique. Reaction temperatures are referred to as the temperature of the heating medium, unless otherwise stated.

1.2. Analytical techniques

NMR-spectra were recorded on Bruker DPX 300, 400 or 500 spectrometers. All spectral data was acquired at 295 K. Deuterated solvents for NMR analysis were purchased from Sigma Aldrich. ^{1}H and ^{13}C chemicals shifts (δ) are quoted in parts per million (ppm) against tetramethylsilane (TMS, δ = 0.00 ppm) and were internally referenced to residual $CHCl_3$ (7.26 ppm for ^{1}H, 77.16 ppm for ^{13}C) or DMSO (2.50 ppm for ^{1}H, 39.52 ppm for ^{13}C). ^{19}F chemicals shifts (δ) are quoted in parts per million (ppm) and were calibrated using absolute referencing to the ^{1}H NMR spectrum. Coupling constants (*J*) are reported in Hertz (Hz) to the nearest 0.1 Hz. The following abbreviations (or combinations thereof) were used to explain multiplicities: s = singlet, d = doublet, t = triplet, q = quartet, quintet, p = pentet, m = multiplet.

High-resolution mass spectra (HRMS) were obtained by the MS service of Cardiff University on a Water LCR Premier XE-TOF. Ions were generated by the Atmospheric Pressure Ionization Techniques (APCI), Electrospray (ESI) and Electron Ionization (EI).

The infrared spectra were obtained by using ATR-FTIR IRAffinity, Shimadzu instrument.

Melting points (m.p.) were determined with a Stuart melting point SMP11 with variable heater using manual 300 °C thermometer and are not corrected.

The cyclic voltammogram studies were performed in an Orygalys OGF500 Potentiostat / Galvanostat with OGFPWR power supply. Working electrode: glassy carbon electrode, counter electrode: Pt wire; reference electrode: $Ag/AgNO_3$; solvents: HFIP, MeCN and MeOH; scan rate, v = 100 mV/s; supporting electrolytes: Bu_4NOAc, NaOAc and Et_4NBF_4.

Thin layer chromatography was carried out on Merck silica gel 60 F_{254} (0.20 mm) pre-coated aluminum sheets and were visualized using UV light (254 nm) and stained with p-anisaldehyde solution.

1.3. Compound purification

Flash chromatography (Combi Flash NEXTGEN 300+ TELEDYNE ISCO) was carried out using silica gel (Redi Sep Rf Gold, 24 Gram Flash Column), eluting with the specified solvent system

as mentioned. Solvents for chromatographic purification (*n*-hexane, MeOH and EtOAc) were purchased from commercial sources and used directly.

1.4. Electrochemical setup

Flow electrochemical experiments were carried out with an Ion electrochemical reactor from Vapourtec Ltd. In this setup, working and counter electrodes (5 cm × 5 cm) were separated by a 0.5 mm thick FEP spacer resulting in a reactor volume of 600 μL and exposed electrode surface area of 12 cm^2. Rigid graphite (99.95% purity), Platinum foil (99.95% purity), Nickel foil (99.95% purity) were procured from Goodfellow and glassy carbon electrode was obtained from Vapourtec. KR Analytical Ltd Fusion 100 Touch syringe pumps were used to pump the reagent solution through the assembled undivided flow electrochemical reactor and was collected in suitable volumetric flask or vial. Aim-TTi Digital Bench Power Supply (280 W, 2 Output, 0 → 35 V, 0 → 4 A) was used for electrolysis under constant current conditions (Figure S1).

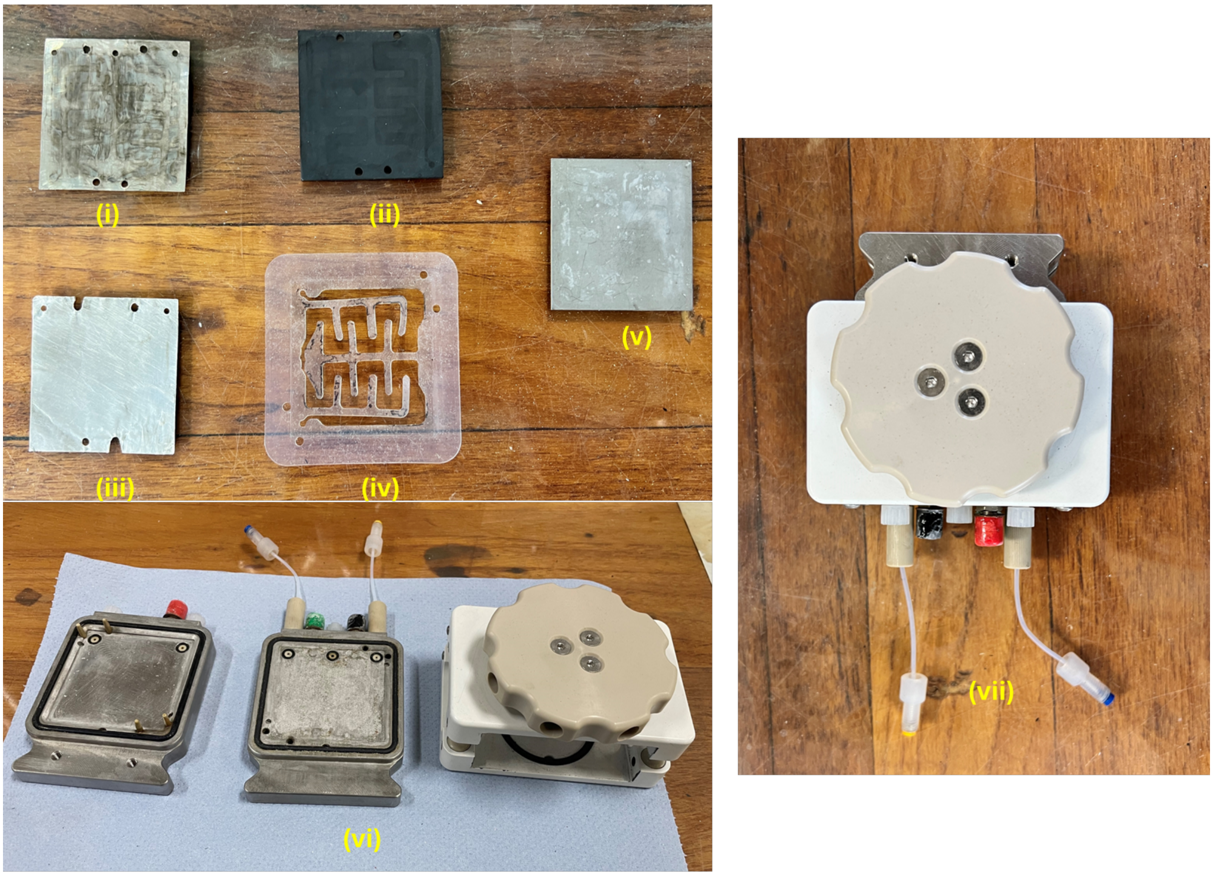

Figure S1. The Vapourtec Ion Electrochemical Flow Reactor. (i) Nickel electrode, (ii) Graphite electrode, (iii) Platinum electrode, (iv) FPE spacer with channel, (v) Stainless steel spacer (vi) Disassembled components (vii) Assembled reactor.

Batch electrochemical experiments were carried out with an Electrasyn 2.0 device with 10 mL Electrasyn vials. Nickel plate and graphite (5 cm x 0.8 cm x 0.2 cm) electrodes were obtained

from IKA. Platinum foil (5 cm x 0.5 cm) was wrapped around a piece of PTFE (Polytetrafluoroethylene) block (5 cm x 1.0 cm x 0.2 cm) to prepare the platinum electrode.

2. Experimental details

2.1. Details for the synthesis of all starting materials

The starting materials, 2-alkynylbenzaldehyde and acetophenone *N*-tosylhydrazones (oximes), were prepared using procedures that are analogous to the literature procedures.[55-57]

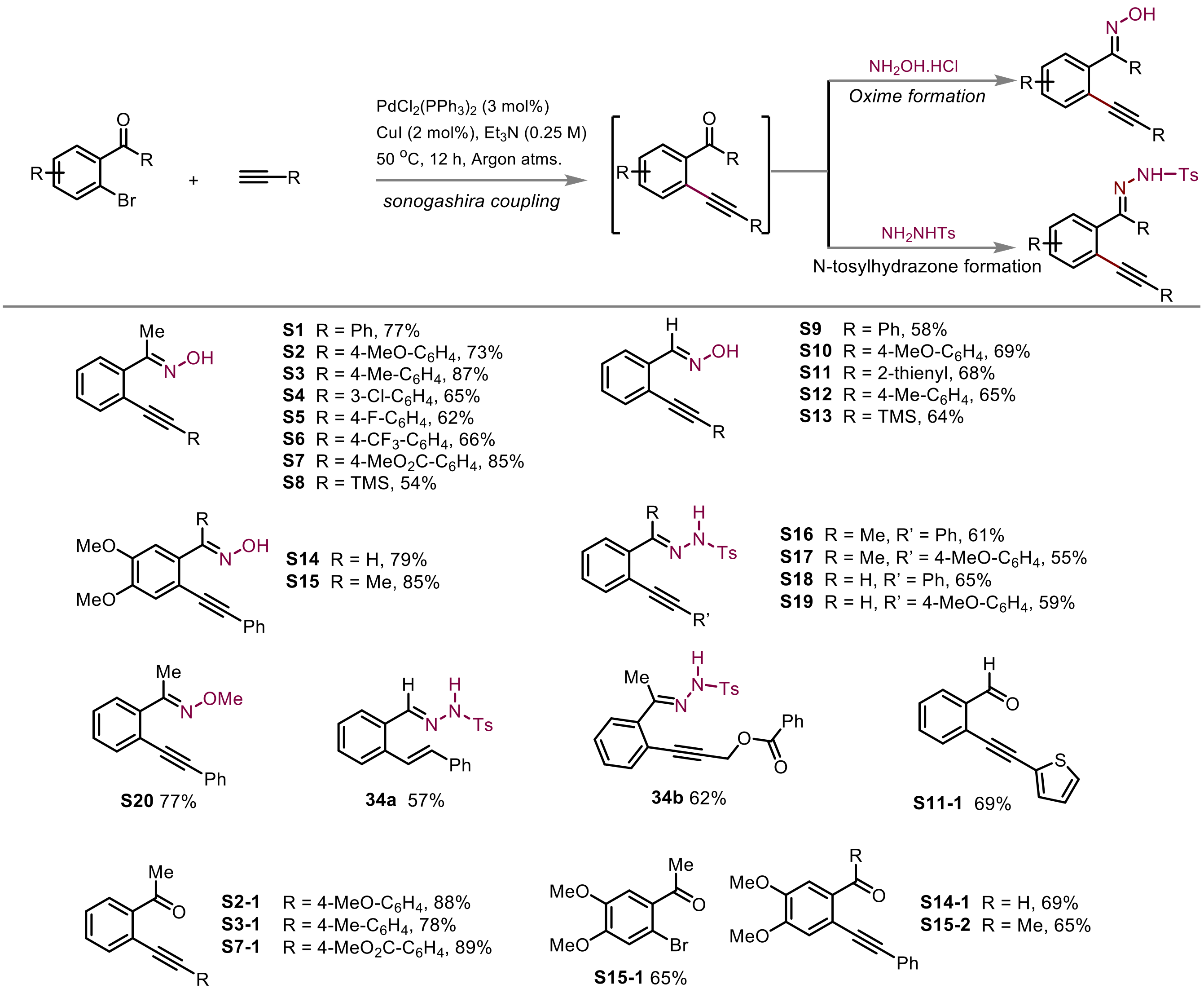

2.1.1. General procedure for the preparation of 2-alkynyl benzaldehyde/acetophenone (GP1)

In an oven dried 100 mL round-bottom flask, a mixture of 2-bromobenzaldehydes or acetophenone (5.0 mmol), CuI (2.0 mol%), $PdCl_2(PPh_3)_2$ (3.0 mol%), and Et_3N (25 mL, 0.2 M) was stirred under an argon atmosphere. A terminal alkyne (5.5 mmol) was added dropwise at room temperature over 5 minutes. The reaction mixture was then heated to 50 °C in a sand bath

and stirred for 8–12 hours. After confirming completion of reaction by TLC, the mixture was cooled to room temperature and concentrated under reduced pressure to remove triethylamine. The crude product was filtered through a short silica pad using diethyl ether. For some starting materials, further purification was done by flash column chromatography on silica gel, using a gradient elution of petroleum ether and ethyl acetate (50:1 to 20:1). The final products, 2-alkynylbenzaldehydes/acetophenones (**S2-1**, **S3-1**, **S7-1**, **S11-1**, **S14-1** and **S15-2**), were obtained in 65%–89% yield.

2.1.2. General procedure for synthesizing 2-alkynyl acetophenone oximes (GP2)

In a 50 mL oven-dried round-bottom flask, the crude 2-alkynyl acetophenone product (obtained from GP1) was combined with $NH_2OH{\cdot}HCl$ (2.5 equiv) and pyridine (3.0 equiv) in dry methanol (0.3 M). The reaction mixture was stirred at 40°C in a sand bath for 2 to 4 hours, and reaction was monitored by TLC, confirming the consumption of 2-alkynyl acetophenone. After completion, the reaction mixture was cooled to room temperature, diluted with 25 mL of water, and extracted with diethyl ether or dichloromethane. The organic layer was dried over anhydrous $MgSO_4$, filtered, and concentrated under reduced pressure. Purification by flash column chromatography over silica gel (hexanes/ethyl acetate, 1:0 to 9:1) yielded the desired 2-alkynyl acetophenone oxime products (**S1-S8**, **S15**).

2.1.3. General procedure for synthesizing 2-alkynylbenzaldehyde oximes (GP3)

In a 50 mL oven-dried round-bottom flask, the crude 2-alkynylbenzaldehyde (obtained from GP1) was dissolved in a 15% (v/v) H_2O/EtOH solution (0.7 M). $NH_2OH{\cdot}HCl$ (2 equiv) was then added, and the reaction mixture was stirred at room temperature for 1 to 3 hours under nitrogen atmosphere. The reaction progress was monitored by TLC, confirming the consumption of 2-alkynylbenzaldehyde. Afterward, most of the ethanol was removed under reduced pressure, and water was added. The mixture was extracted with dichloromethane or diethyl ether, dried over anhydrous $MgSO_4$, and concentrated under reduced pressure. The crude product was purified by flash column chromatography on silica gel (eluted with hexanes/ethyl acetate, 1:0 to 9.5:0.5), yielding the corresponding 2-alkynylbenzaldehyde oximes (**S9-S13**, **S14**).

2.1.4. General procedure for synthesizing 2-alkynyl benzaldehyde and acetophenone N-tosylhydrazones (GP4)

A solution of H_2NNHTs (1.1 equiv) in methanol (0.5 M) was stirred at room temperature while adding pre-prepared 2-alkynylbenzaldehyde and acetophenone (1.0 equiv, 3 mmol). The reaction progress was monitored by TLC. After 0.5 to 2 hours, the crude products precipitated at room temperature or, in some cases, at 0°C. The precipitates were collected by filtration or decantation, washed with cold methanol, and dried under vacuum to obtain pure products. The filtrates were concentrated under reduced pressure to recover additional precipitates, and the work-up was repeated to maximize product yield. The 2-alkynylbenzaldehyde and acetophenone N-tosylhydrazones (**S16-S19**, **34a, 34b**) were obtained in 55% to 65% yields.

2.1.5. General procedure for synthesizing 2-alkynyl acetophenone oximes (S4, S6)

In a 50 mL oven-dried round-bottom flask, the crude 2-alkynyl acetophenone product (obtained from GP1) was combined with $NH_2OH{\cdot}HCl$ (2.5 equiv) and NaOAc (3.0 equiv) in dry acetonitrile (0.3 M). The reaction mixture was stirred at 70°C in a sand bath for 4 hours, and reaction was monitored by TLC, confirming the consumption of 2-alkynyl acetophenone. After completion, the reaction mixture was cooled to room temperature. The crude product was filtered through a short celite pad using diethyl ether and concentrated under reduced pressure. Purification by flash column chromatography over silica gel (hexanes/ethyl acetate, 1:0 to 9:1) yielded the desired 2-alkynyl acetophenone oxime products with non-isolable impurities (**S4, S6**).

2.1.6. Experimental procedure for synthesizing 2-alkenyl benzaldehyde *N*-tosylhydrazone (34a-1)

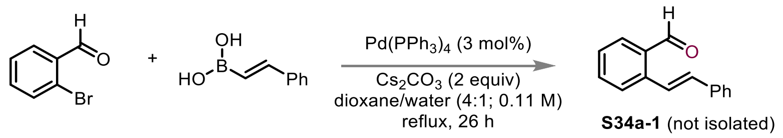

S34a-1 (not isolated)

The precursor (**34a-1**) for **34a** was prepared following a reported procedure.[58] A solution of 2-bromobenzaldehyde (0.5 g, 2.7 mmol) in a degassed dioxane/water mixture (20 mL/5 mL) was treated with [$Pd(PPh_3)_4$] (93 mg, 0.8 mmol, 3 mol%), Cs_2CO_3 (1.71 g, 5.4 mmol, 2 equiv), and trans-2-phenylvinylboronic acid (480 mg, 3.245 mmol, 1.2 equiv). The mixture was refluxed for 26 hours, then cooled, and water (15 mL) was added. After organic layer separation, the aqueous layer was extracted with CH_2Cl_2 (2 x 20 mL). The combined organic layers were dried over $MgSO_4$, filtered, and concentrated under reduced pressure. The crude product (**34a-1**) was directly used to synthesize 2-alkenyl benzaldehyde *N*-tosylhydrazone **34a** following **GP4**.

2.1.7. Experimental procedure for synthesizing 1-(2-(phenylethynyl)phenyl)ethan-1-one O-methyl oxime (S20)

The compound **S20** was prepared following a reported procedure.[30] In a 50 mL oven-dried round-bottom flask, the crude 2-alkynyl acetophenone precursor (5 mmol) from step 1 was dissolved in 10 mL of ethanol. Pyridine (10 mmol, 2 equiv) and methoxyammonium chloride (7.5 mmol, 1.5 equiv) were then added sequentially. The mixture was stirred at room temperature for 12 hours. After completion, the reaction mixture was extracted with ethyl acetate. The organic layer was washed with water and brine, dried over $MgSO_4$, filtered, and concentrated under reduced pressure. The crude product was purified by column chromatography to obtain a yellow liquid, **S20**.

2.1.8. Experimental procedure for synthesizing 1-(2-bromo-4,5-dimethoxyphenyl)ethan-1-one (S15-1)

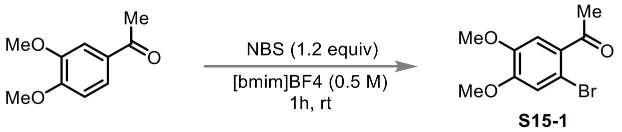

S15-1

The precursor (**S15-1**) for **S15** was prepared following a reported procedure.[59] A commercially available 1-(3,4-dimethoxyphenyl)ethan-1-one (5 mmol) was mixed with N-bromosuccinimide

(12 mmol, 1.2 equiv) in [bmim]BF_4 (10 mL) and stirred at room temperature for 1 hour. The reaction progress was monitored by TLC. After completion, the mixture was washed with diethyl ether (3 × 10 mL). The combined organic layers were concentrated under vacuum, and the crude product was purified by column chromatography with ethyl acetate:n-hexane (1:9) as the eluent, yielding the pure brominated arene (**S15-1**).

2.1.9. Experimental procedure for synthesizing 6-phenylhex-5-yn-2-one oxime (S21)

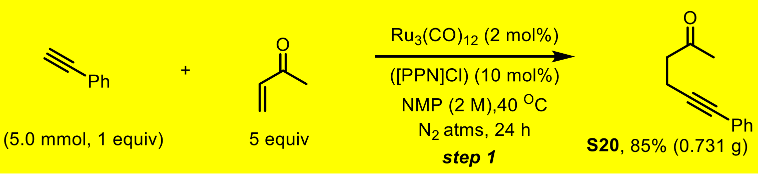

Step 1: The compound **S20** was prepared following a reported procedure.[60]
A mixture of $Ru_3(CO)_{12}$ (64 mg, 0.1 mmol, 2 mol%), Bis(triphenylphosphine)iminium chloride ([PPN]Cl) (287 mg, 0.5 mmol, 10 mol%), and 2.5 mL of *N*-Methyl-2-pyrrolidone (NMP) was stirred in a 25 mL round bottom flask at 40 °C under a nitrogen atmosphere. After 15 minutes, methyl vinyl ketone (25.0 mmol, 5 equiv) and phenylacetylene (5.0 mmol, 1 equiv) were added. The reaction mixture was then stirred at 40 °C for 24 hours. Once complete, it was allowed to cool to room temperature, filtered through a short pad of celite, and the filtrate was concentrated under reduced pressure to give an oily residue. This crude product was purified by flash column chromatography on silica gel using a 2:98 mixture of ethyl acetate and hexane as the eluent, yielding the desired compound **S20** as colorless oil (85% (0.731 g).

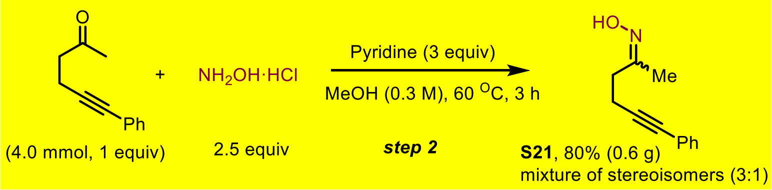

Step 2: The compound **S21** was prepared following a general procedure (GP2) In a 50 mL oven-dried round-bottom flask, the 6-phenylhex-5-yn-2-one **S20** product was combined with $NH_2OH·HCl$ (2.5 equiv) and pyridine (3.0 equiv) in dry methanol (0.3 M). The reaction mixture was stirred at 60°C in a sand bath for 3 hours, and reaction was monitored by TLC, confirming the consumption of 6-phenylhex-5-yn-2-one **S20**. After completion, the reaction mixture was cooled to room temperature, diluted with 25 mL of water, and extracted with diethyl ether. The organic layer was dried over anhydrous $MgSO_4$, filtered, and concentrated under reduced pressure. Purification by flash column chromatography over silica gel (hexanes/ethyl acetate, 1:0 to 9:1) yielded the desired 6-phenylhex-5-yn-2-one oxime **S21** as mixture of stereoisomers (colorless oil, 80%, 0.6 g).

2.1.10. Experimental procedure for synthesizing 1-(2-(phenylethynyl)pyridin-3-yl)ethan-1-one oxime (S25)

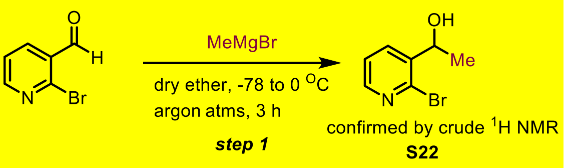

Step 1: The compound **S22** was prepared following a reported procedure.[61] To a dry solution of 2-bromopyridine-3-carboxaldehyde (2g, 11 mmol, 1 equiv) in diethyl ether (2 mL per mmol, 22 mL), a solution of methylmagnesium bromide (23.0 mL, 22 mmol) was added slowly at –78 °C under an argon atmosphere. Once the addition was complete, the reaction mixture was allowed to gradually warm to 0 °C and stirred for 3 hours at that temperature. The reaction was then quenched with a saturated aqueous solution of ammonium chloride (10–15 mL per mmol). The mixture was extracted with diethyl ether (3 portions of 20 mL per mmol). The combined organic layers were washed with brine, then dried over anhydrous magnesium sulfate. After filtration, the solvent was removed under reduced pressure. The product was confirmed by crude ^{1}H NMR.

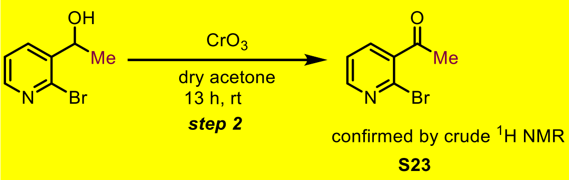

Step 2: The compound **S23** was prepared following a reported procedure.[62] To a solution of the crude 1-(2-bromopyridin-3-yl)ethanol (obtained from step 1) in acetone (30 mL), chromium trioxide (CrO_3, 2.5 g, 25 mmol) was slowly added in small portions while stirring. The reaction mixture was stirred at room temperature for 13 hours. After completion, the solvent was removed under reduced pressure. To the residue, a solution of sodium hydroxide (3.3 g, 82 mmol) in 20 mL of water was added along with 50 mL of diethyl ether. The mixture was stirred for another 30 minutes. The organic layer was then separated, dried over anhydrous sodium sulfate. Finally, the solvent was evaporated under reduced pressure to obtain the crude product and confirmed by crude ^{1}H NMR.

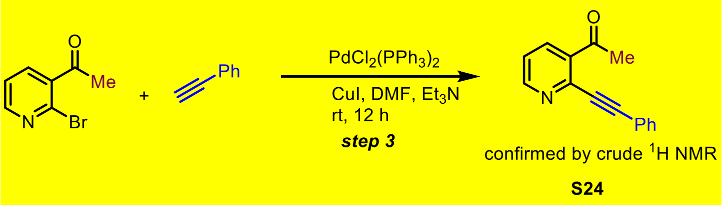

Step 3: The compound **S24** was prepared following a reported procedure.[63] In a dry flask under argon, the crude **S23** (obtained from step 2), the phenylacetylene (1.2 equiv, 13.2 mmol) and triethylamine (6.6 mL, 0.6 mL per mmol). were suspended in anhydrous DMF (11 mL, 1 mL per mmol of **S23**). To this mixture, the copper(I) iodide (2.5 mol%) and palladium(II) chloride triphenylphosphine complex (5 mol%) were added. The reaction mixture was stirred 12 h at room temperature. After completion, the reaction mixture was diluted with 50 mL of water along with ethyl acetate and diethyl ether (1:1, 30 mL). The organic layer was then separated, dried over anhydrous sodium sulfate. Finally, the solvent was evaporated under reduced pressure to obtain the crude product and confirmed by crude ^{1}H NMR.

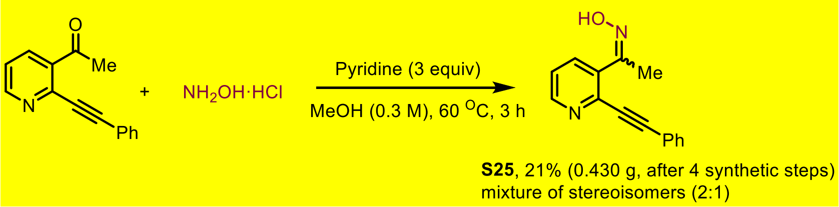

Step 4: The compound **S25** was prepared following a general procedure (GP2). In a 100 mL oven-dried round-bottom flask, the **S24** crude product (obtained from step 3) was combined with $NH_2OH·HCl$ (27.5 mmol, 2.5 equiv) and pyridine (33 mmol, 3.0 equiv) in dry methanol (0.3 M). The reaction mixture was stirred at 60°C in a sand bath for 3 hours, and reaction was monitored by TLC, confirming the consumption of **S24**. After completion, the reaction mixture was cooled to room temperature, diluted with 50 mL of water, and extracted with ethyl acetate. The organic layer was dried over anhydrous $MgSO_4$, filtered, and concentrated under reduced pressure. Purification by flash column chromatography over silica gel (hexanes/ethyl acetate, 1:0 to 8:2) yielded the desired 1-(2-(phenylethynyl)pyridin-3-yl)ethan-1-one oxime **S25** as mixture of stereoisomers (white solid, 21% yield (0.430 g) based on the starting material (11 mmol) used in step 1, after 4 synthetic steps).

2.1.11. General procedure for synthesis of diaryl diselenides (Se1 – Se5):

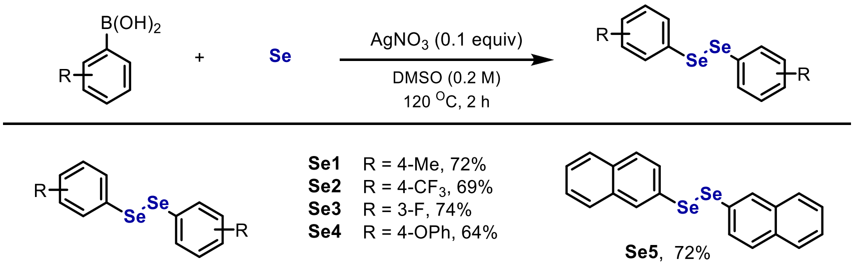

The diaryl diselenides were prepared using modified methods based on literature procedures.[64] A 50 mL oven-dried round-bottom flask was charged with arylboronic acid (4 mmol, 1 equiv), selenium (12 mmol, 3 equiv), silver nitrate ($AgNO_3$) (0.4 mmol, 0.1 equiv), and dimethyl sulfoxide (DMSO) (20 mL, 0.2 M). The reaction mixture was stirred at 120 °C for 2 hours. After cooling to room temperature, the mixture was diluted with water (20 mL) and extracted with diethyl ether (3 × 15 mL). The combined organic layers were washed sequentially with water and brine (30 mL), dried over anhydrous magnesium sulfate ($MgSO_4$), and concentrated under reduced pressure. The crude product was purified by flash column chromatography using ethyl acetate/n-hexane (1:49) as the eluent, affording the desired diaryl diselenides (**Se1 – Se5**).

2.2. Flow-electrolysis optimization details

2.2.1.General procedure for flow-electrolysis optimization studies:

A solution of oxime **S1** (0.25 mmol) in the solvent system, containing diphenyl diselenide **2** and the electrolyte, was pumped into a Vapourtec Ion Electrochemical Flow Reactor (reactor volume: 0.6 mL; spacer: 0.5 mm) using a syringe pump at a flow rate of 0.05 mL/min. Electrolysis

was carried out under a constant current (charge F/mol) using anode and cathode electrodes with an effective surface area of 12 cm². After reaching steady-state conditions (24 minutes; equivalent to two reactor volumes), the initial outflow was discarded, and the product solution was collected for 48 minutes in a glass vial. The resulting mixture was concentrated under reduced pressure, and the crude residue was diluted with an internal standard solution (100 µL, 1 M CH_2Br_2 in $CDCl_3$) and additional $CDCl_3$. The final solution was passed through a syringe filter. Conversion and yield were determined by integration of the methyl peaks (for isoquinoline *N*-oxide) and aromatic peaks (for isoindole *N*-oxide) in the ^{1}H NMR spectrum.

2.2.2. Optimization of flow electrosynthesis conditions for isoquinoline *N*-oxide (3):

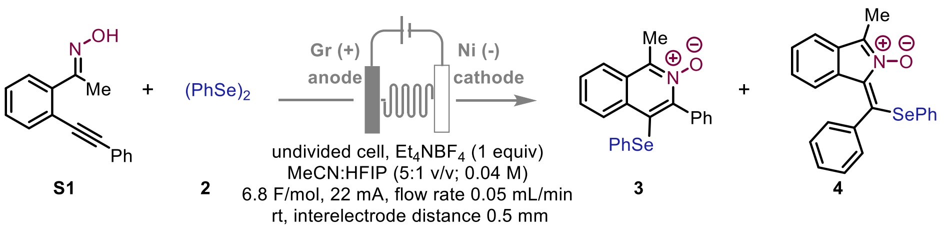

entry		variants	conversion[a] (%)	yield of **3**[a] (%)	yield of **4**[a] (%)
1		none	>99	92 (85)[b]	-
2		no electricity	<1	-	-
3		no electrolyte	89	5	34
4		30 mol%	68	28	-
5	electrolyte loading	50 mol%	84	41	-
6		80 mol%	>99	83 (77)	-
7		Pt cathode	>99	77	-
8	electrode material	Gr cathode	>99	79	-
9		Ni cathode	>99	92 (85)	-
10	diphenyl diselenide **2** loading	1.5 equiv	>99	90 (86)	-
11		10 mA (3 F/mol)	42	40	-
12	charge applying	15 mA (4.7 F/mol)	64	63	-
13		22 mA (6.8 F/mol)	>99	92 (85)	-
14		Et_4NBF_4	>99	92 (85)	-
15		LiClO4	96	68	-
16	electrolyte screening	$n$$Bu_4NPF_6$	90	60	-
17		$n$$Bu_4NHSO_4$	92	65	-
18		Bu_4NBF_4	>99	78	-
19		NaOAc	85	-	43[c]
20	solvent system	MeCN	95	65	-
21		MeCN:HFIP	>99	92 (85)	-
22		MeOH: MeCN:HFIP	>99	74[d]	-
23		batch electrolysis	84	79 (76)[e]	-

[a] Reaction conditions, oxime **S1** (0.25 mmol), $(PhSe)_2$ (1 equiv), Et_4NBF_4 (1 equiv), MeCN:HFIP (5:1 v/v; 0.04 M) 6.6 F/mol, 22 mA, flow rate 0.05 mL/min, rt, interelectrode distance 0.5 mm, graphite anode and nickel cathode, undivided cell; [b] Isolated yield in parentheses; [c] Solvent system MeCN:HFIP:H_2O (4.8:1:0.2 v/v/v; 0.04 M); [d] Solvent composition (0.5:4.5:1 v/v/v; 0.04 M) and Pt electrode as cathode used; [e] Electrolysis carried out using IKA Electrasyn 2.0, constant current 10 mA, charge 3.5 F/mol.

2.2.3. Optimization of flow electrosynthesis conditions for isoindole *N*-oxide (4):

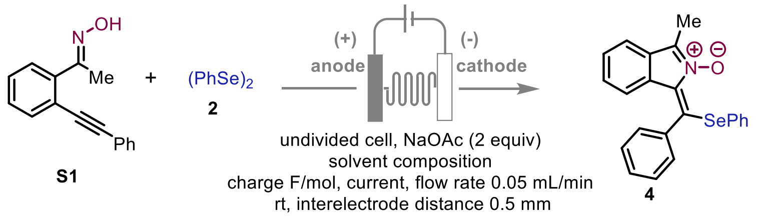

entry	electrode system	current (mA)	charge (F mol^{-1})	solvent composition (v/v/v, M)	conversion **S1**(%)[a]	yield **4** (%)[a]
1	Gr(+) I Ni(-)	10	3.5	MeCN:HFIP (5:1, 0.04 M)	>99	55[b]
2	Gr(+) I Ni(-)	22	6.8	H_2O: MeCN:HFIP (0.2:5:1, 0.04 M)	86	48
3	Gr(+) I Ni(-)	22	6.8	H_2O: MeCN:HFIP (0.4:4.8:1, 0.04 M)	78	39
4	Gr(+) I Ni(-)	16.5	6.4	H_2O: MeCN:HFIP (0.2:5:1, 0.04 M)	40	39[c]
5	Gr(+) I Ni(-)	20	6.67	MeOH: MeCN:HFIP(0.5:4.5:1, 0.038 M)	78	74(76)
6	Gr(+) I Ni(-)	22	7.2	MeCN:MeOH (4:2.5, 0.038 M)	68	56
7	Gr(+) I Ni(-)	27	8.85	MeCN:MeOH (4:2.5, 0.038 M)	>99	70
8	Gr(+) I Pt(-)	27	8.85	MeCN:MeOH (1:1, 0.039 M)	>99	88(87)
9	Gr(+) I Pt(-)	32	10.5	MeCN:MeOH (1:1, 0.039 M)	>99	62
10	Gr(+) I Pt(-)	27	8.85	MeCN:MeOH (1:1, 0.039 M)	94	58[d]
11	Gr(+) I Pt(-)	32	10.5	MeCN:MeOH (1:1, 0.039 M)	>99	67[d]
12	Pt(+) I Pt(-)	27	8.85	MeCN:MeOH (1:1, 0.039 M)	66	33
13	Gr(+) I GC(-)	27	8.85	MeCN:MeOH (1:1, 0.039 M)	40	5

[a] Reaction conditions, oxime **S1** (0.25 mmol), $(PhSe)_2$ (1 equiv), NaOAc (2 equiv), MeCN:MeOH (1:1, 0.039 M), 8.85 F/mol, 27 mA, flow rate 0.05 mL/min, rt, interelectrode distance 0.5 mm, graphite anode and platinum cathode, undivided cell; [b] Reaction performed under batch electrolysis conditions; [c] Flow rate 0.04 mL/min; [d] Bu_4NOAc used as electrolyte instead of NaOAc.

2.3. General electrolysis procedures for isoquinoline/isoindole derivatives synthesis

2.3.1.Batch electrolysis procedure for synthesis of isoquinoline N-oxide (3):

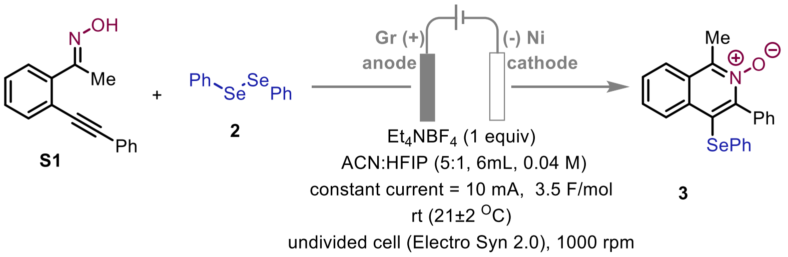

Batch electrolysis was performed using the IKA Electrasyn 2.0. A solution of 0.25 mmol oxime **S1** in 5 mL acetonitrile (MeCN) and 1 mL hexafluoroisopropanol (HFIP), was added diphenyl diselenide **2** (78 mg, 0.25 mmol, 1 equiv) and Et_4NBF_4 (54 mg, 0.25 mmol, 1 equiv) employing a graphite anode and a nickel cathode, was electrolyzed. The electrolysis was performed under constant current (i = 10 mA) conditions (j = 8.3 mA·cm^{-2}, active surface area 1.2 cm^2, 3.5 F). After completion of electrolysis, both electrodes were thoroughly rinsed with ethyl acetate (10

mL), and diluted with 25 mL of water, the aqueous layer was extracted three times with 10 mL of ethyl acetate, dried over $MgSO_4$ and filtered. The crude product was purified by flash column chromatography using methanol/ethyl acetate/n-hexane (1.5:13.5:85) as the eluent, affording the desired isoquinoline *N*-oxide (**3**).

2.3.2.Batch electrolysis procedure for synthesis of isoindole *N*-oxide (4):

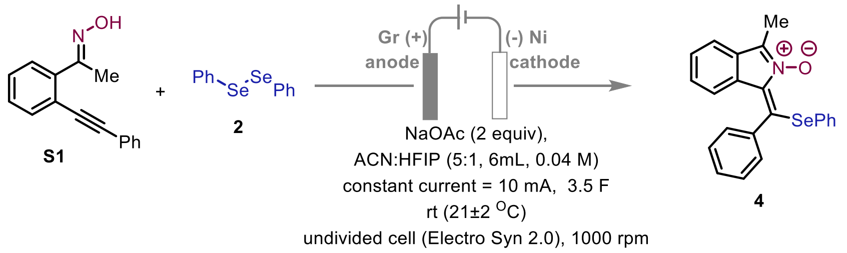

Batch electrolysis was performed using the IKA Electrasyn 2.0. A solution of 0.25 mmol oxime **S1** in 5 mL acetonitrile (MeCN) and 1 mL hexafluoroisopropanol (HFIP), was added diphenyl diselenide **2** (78 mg, 0.25 mmol, 1 equiv) and NaOAc (41 mg, 0.5 mmol, 2 equiv) employing a graphite anode and a nickel cathode, was electrolyzed. The electrolysis was performed under constant current (i = 10 mA) conditions (j = 8.3 mA·cm^{-2}, active surface area 1.2 cm^2, 3.5 F). After completion of electrolysis, both electrodes were thoroughly rinsed with ethyl acetate (10 mL), and diluted with 25 mL of water, the aqueous layer was extracted three times with 10 mL of ethyl acetate, dried over $MgSO_4$ and filtered. The crude product was purified by flash column chromatography using ethyl acetate/n-hexane (30:70) as the eluent, affording the desired isoindole *N*-oxide (**4**).

2.3.3.General flow-electrolysis protocol for the substrate-scope investigation of isoquinoline *N*-oxides (GP5)

A solution of oximes/N-tosylhydrazones (0.4 mmol) in solvent system acetonitrile and hexafluoroisopropanol (ACN:HFIP, 5:1 v/v, 0.04 M), containing diselenide (1 equiv) and Et_4NBF_4 (87 mg, 0.4 mmol, 1 equiv), was introduced into a Vapourtec Ion Electrochemical Flow Reactor (reactor volume: 0.6 mL; spacer: 0.5 mm) using a syringe pump at a flow rate of 0.05 mL/min. Electrolysis was carried out under a constant current of 22 mA employing a graphite anode and a nickel cathode (effective electrode surface area: 12 cm²). After reaching steady-state conditions (24 min; equivalent to two reactor volumes, were discarded), the product stream was collected in a glass vial. After electrolysis, both electrodes were thoroughly rinsed with ethyl acetate (15 mL), and diluted with 30 mL of water, the aqueous layer was extracted three times with 15 mL of ethyl acetate, dried over $MgSO_4$ and filtered. The crude product was purified by flash column chromatography using methanol/ethyl acetate/n-hexane (v/v/v 1.5:13.5:85) as the eluent, affording the desired isoquinolinium *N*-oxide and isoquinolinium imide derivatives.

Note: A modified solvent system of MeCN:HFIP:DCE (4:1:1 v/v/v; 0.04 M) was employed for reactions of *N*-tosylhydrazones (**S16-S19, 34a, 34b**) as substrates, and for oximes (**S1**, **S5**, **S8**, **S9**, **S11**) in combination with dibenzyl diselenide, di-2-naphthyldiselane, or diphenyl ditelluride.

This composition was chosen to ensure complete solubility of all reaction components and afforded the corresponding isoquinolinium *N*-oxide and isoquinolinium imide derivatives.

General procedure for scaling up flow electrochemical reactions

For scaling up the flow electrochemical reactions, we followed the general procedure **GP5** but used a solvent mixture of ACN, HFIP, and DCE in a 1:1:1 ratio (0.04 M) and electrolyte loading 0.8 equiv. The reaction was carried out on a 3 mmol scale using substrate **S9**, and on a 5 mmol scale using substrate **S18**, with the electrochemical reactor connected to a peristaltic pump.

2.3.4. General flow-electrolysis protocol for the substrate-scope investigation of isoindole N-oxides (GP6)

A solution of oximes/N-tosylhydrazones (0.4 mmol) in solvent system acetonitrile and methanol (ACN:MeOH, 1:1 v/v, 0.04 M), containing diselenide (1 equiv) and NaOAc (66 mg, 0.8 mmol, 2 equiv), was introduced into a Vapourtec Ion Electrochemical Flow Reactor (reactor volume: 0.6 mL; spacer: 0.5 mm) using a syringe pump at a flow rate of 0.05 mL/min. Electrolysis was carried out under a constant current of 27 mA employing a graphite anode and a platinum cathode (effective electrode surface area: 12 cm^2). After reaching steady-state conditions (24 min; equivalent to two reactor volumes, were discarded), the product stream was collected in a glass vial. After electrolysis, both electrodes were thoroughly rinsed with ethyl acetate/diethyl ether (15 mL), and diluted with 30 mL of water, the aqueous layer was extracted three times with 15 mL of ethyl acetate or diethyl ether, dried over $MgSO_4$ and filtered. The crude product was purified by flash column chromatography using ethyl acetate/n-hexane (30:70) as the eluent, affording the desired isoindole *N*-oxides.

Note:

1. Flow electrolysis of oximes (**S1**, **S2**) with dibenzyl diselenide was carried out at a low constant current (10 mA) to improve yields of **37** and **40**. Under optimized constant current conditions, we observed poor yields (< 20 %) and unidentifiable byproducts.
2. A solvent mixture of MeCN:MeOH:HFIP (5:0.5:1 v/v/v; 0.04 M substrate) was used to further boost yields of **39**, **40** and **41**. Reactions run in the presence of HFIP gave clean reaction profiles.

2.3.5. Unsuccessful substrates for synthesis of isoquinoline N-oxides and isoindole N-oxides under flow-electrolysis

unsuccessful substrates for synthesis of isoquinoline N-oxides

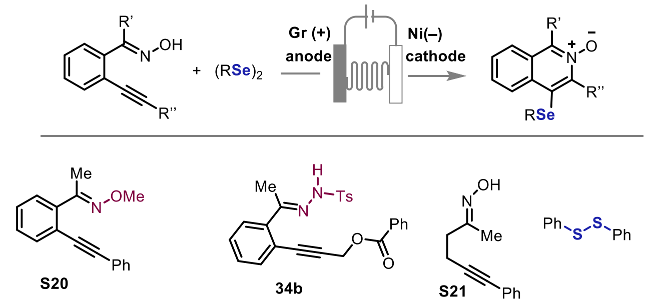

unsuccessful substrates for synthesis of isoindole N-oxides:

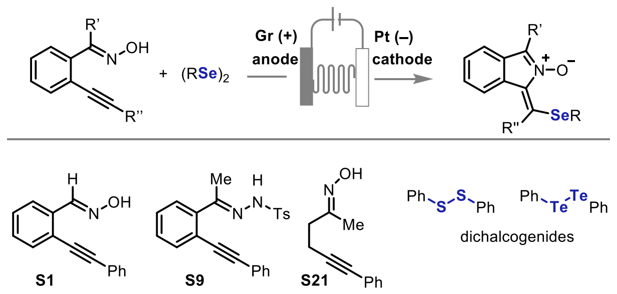

2.4. Mechanistic experiments

2.4.1. Reaction with radical scavenger 2,2,6,6-tetramethylpiperidin-1-yl)oxyl (TEMPO) under standard conditions B

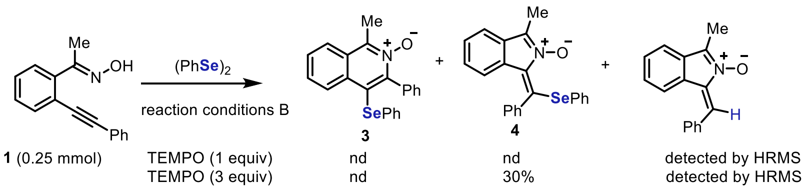

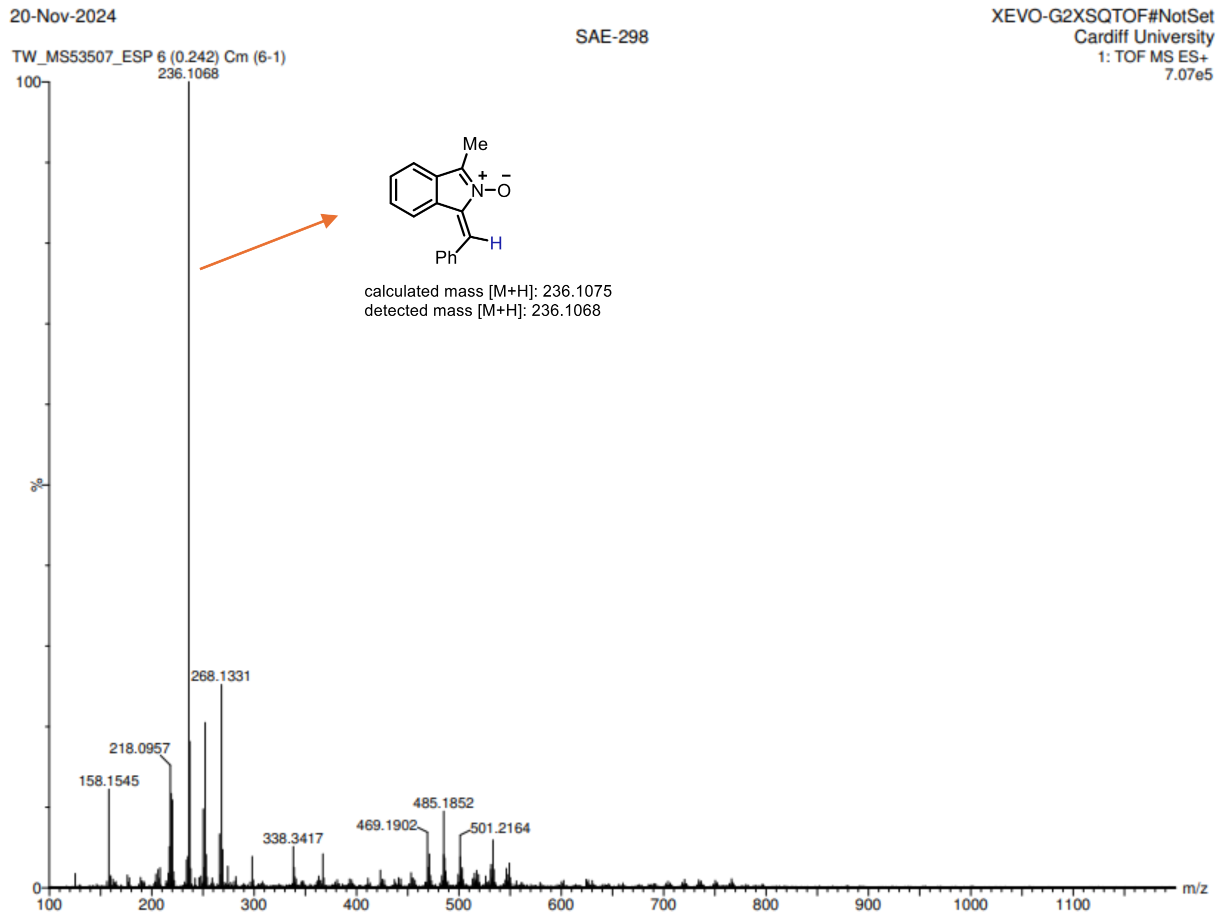

Experimental procedure: The above experiments are carried out according to general procedure **GP6**.

2.4.2. Reaction with radical scavenger butylated hydroxytoluene (BHT) under standard conditions B

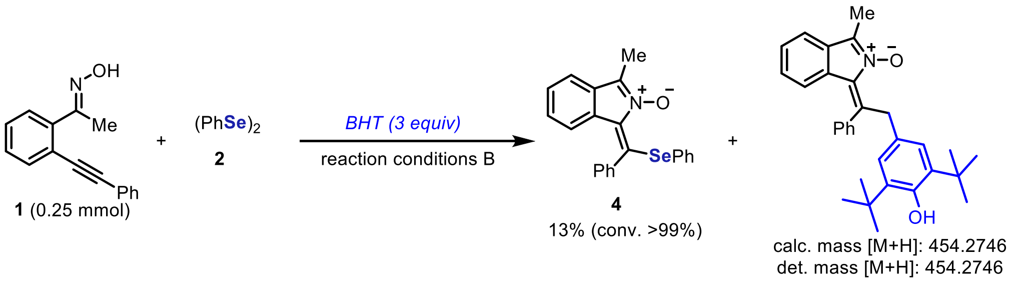

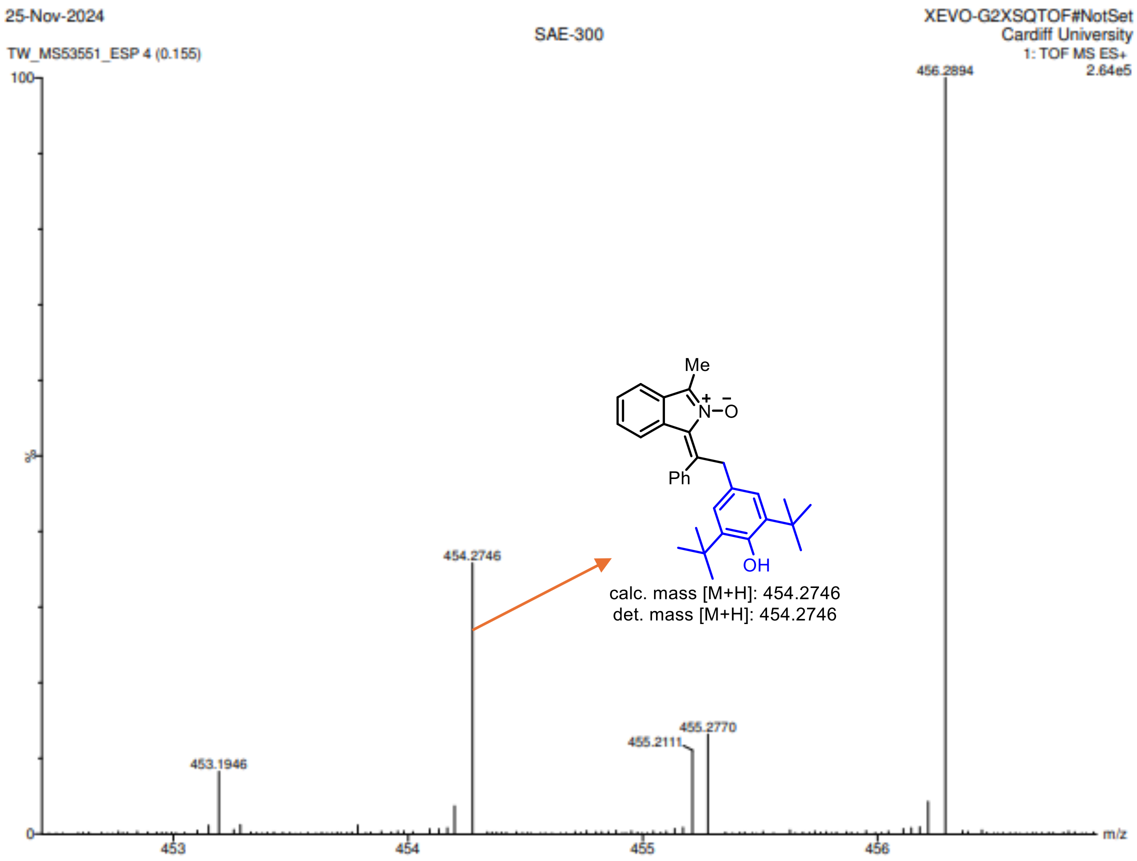

Experimental procedure: The above experiment is carried out according to general procedure **GP6**.

2.5. Cyclic voltammetry experiments and rotating disk electrode (RDE) studies

Cyclic voltammetry (CV) experiments were conducted in a 10 mL glass vial fitted with a glassy carbon working electrode (3 mm in diameter), $Ag/AgNO_3$ reference electrode, and a platinum wire counter electrode. All cyclic voltammetry studies were conducted with Orygalys OGF500 Potentiostat / Galvanostat with OGFPWR power supply. Scan rate is 100 mV/s unless specified.

Supporting electrolytes: NaOAc, Bu_4NOAc and Et_4NBF_4 were used as such received from a commercial source.

Solvents: The argon-sparged CH_3CN, HFIP and MeOH solvents were used.

Working electrode: The working electrode is a glassy carbon electrode (3 mm). Polished with 0.3 µm aluminum oxide and then sonicated in distilled water for 5 min and dried.

Reference electrode: The reference electrode consisted of a silver wire submerged in a 0.01 M $AgNO_3$ with a supporting electrolyte ($LiClO_4$).

Counter electrode: The counter electrode is a platinum wire (0.4 mm) used. Polished with 0.3 µm aluminum oxide and then sonicated in distilled water for 5 min and dried.

Cyclic voltammetry (CV) experiments were carried out under reaction conditions B but using Bu_4NOAc as the electrolyte (Figure S2), and the electrochemical behavior of the reagents was found to be similar to that under standard conditions B (Figure S4).

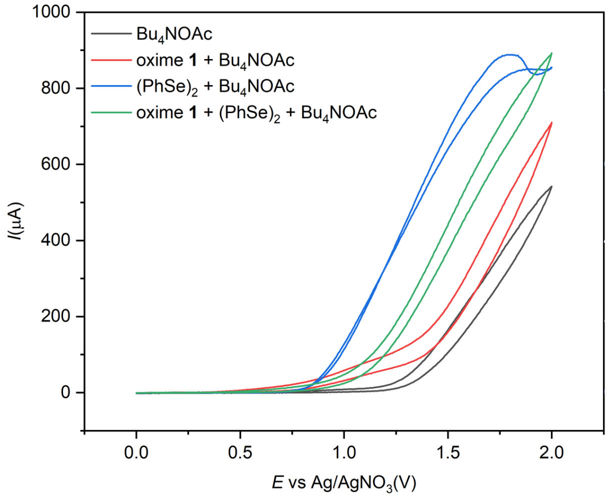

Figure S2: Cyclic voltammograms (100 mV/s; working electrode: GC; reference electrode: Ag/Ag+ (10 mM $AgNO_3$); counter electrode: Pt wire) obtained under reaction conditions (ACN: MeOH: HFIP, 4.5:0.5:1 v/v/v, 0.04 M) with 80 mM Bu_4NOAc. (i) 80 mM Bu_4NOAc (Block), (ii) 40 mM oxime **S1** (Red), (iii) 40 mM diphenyl diselenide (Blue) (iv) 40 mM oxime **S1** and 40 mM diphenyldiselenide (Green).

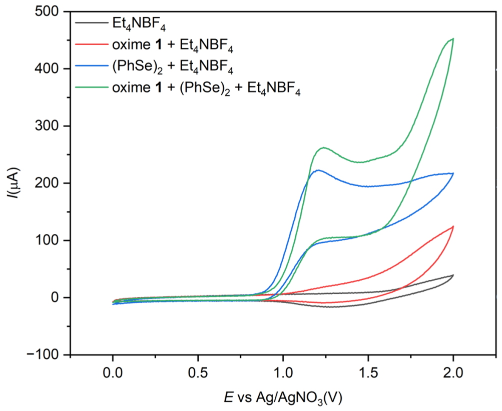

Figure S3: Cyclic voltammograms (100 mV/s; working electrode: GC; reference electrode: Ag/Ag+ (10 mM $AgNO_3$); counter electrode: Pt wire) obtained under reaction conditions A (ACN: HFIP, 5:1 v/v, 0.04 M) with 80 mM Et_4NBF_4. (i) 80 mM Et_4NBF_4 (Block), (ii) 40 mM oxime **S1** (Red), (iii) 40 mM diphenyl diselenide (Blue) (iv) 40 mM oxime **S1** and 40 mM diphenyldiselenide (Green).

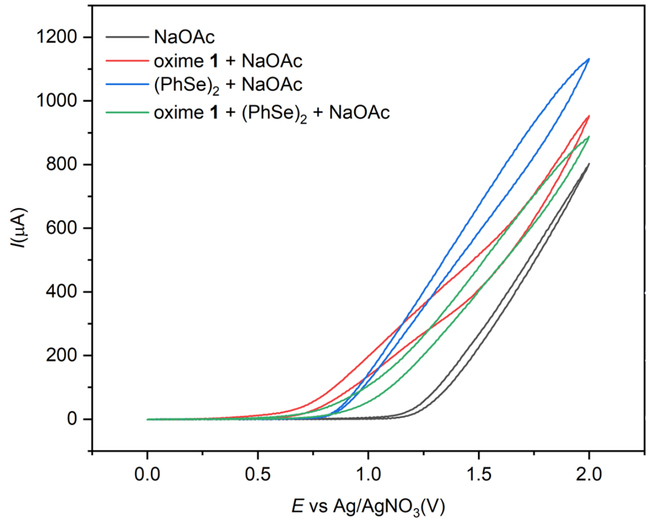

Figure S4: Cyclic voltammograms (100 mV/s; working electrode: GC; reference electrode: Ag/Ag+ (10 mM $AgNO_3$); counter electrode: Pt wire) obtained under reaction conditions B (ACN: MeOH, 1:1 v/v, 0.04 M) with 80 mM NaOAc. (i) 80 mM NaOAc (Block), (ii) 40 mM oxime **S1** (Red), (iii) 40 mM diphenyldiselenide (Blue) (iv) 40 mM oxime **S1** and 40 mM diphenyldiselenide (Green).

Rotating disk electrode (RDE) studies

All electrochemical experiments were performed using a three-electrode undivided electrochemical cell consisting of i) an Ivium glassy carbon rotating disk electrode (GC RDE) constituting as the working electrode, with an area of 0.196 cm^2; ii) Ag/$AgNO_3$ reference electrode; iii) a basi platinum wire as the counter electrode. All voltammetry's were conducted with an Ivium CompactStat.h potentiostat, with cyclic voltammetry's scanned from 0 V to 2 V vs. Ag/$AgNO_3$ at a scan rate of 100 mV/s, with the GC RDE rotated at multiple scan rates of 500, 1000, 1500, and 2000 RPM.

Rotating disk electrode (RDE) studies under conditions A

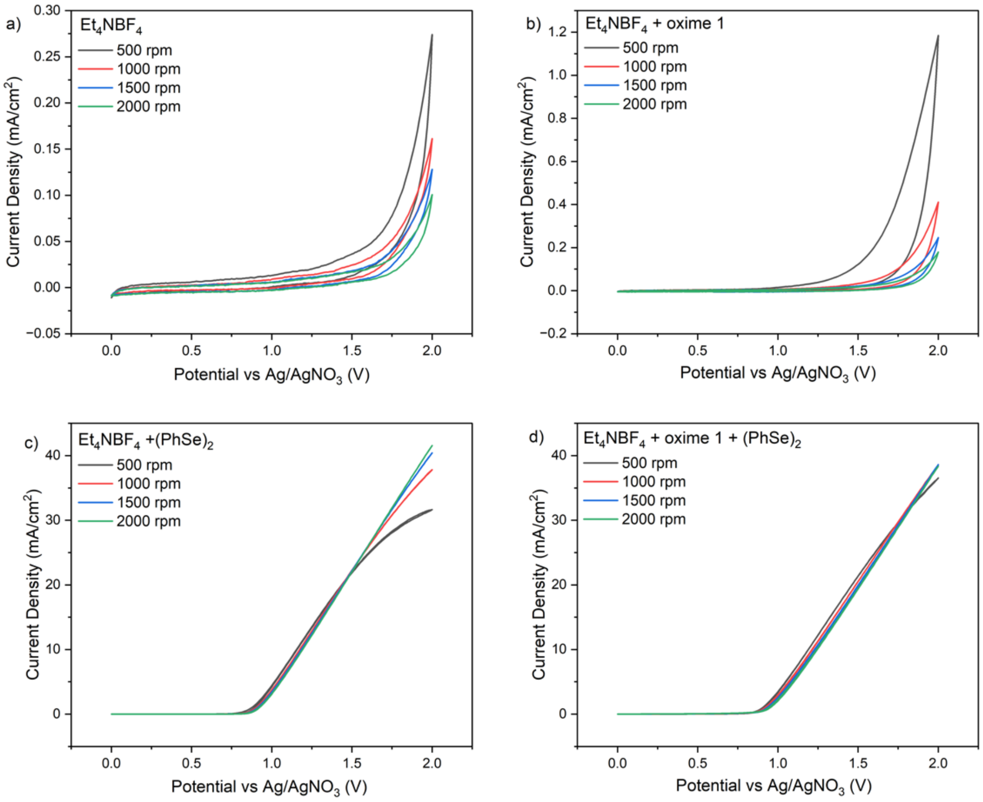

Rotating disk electrode (RDE) studies under conditions B

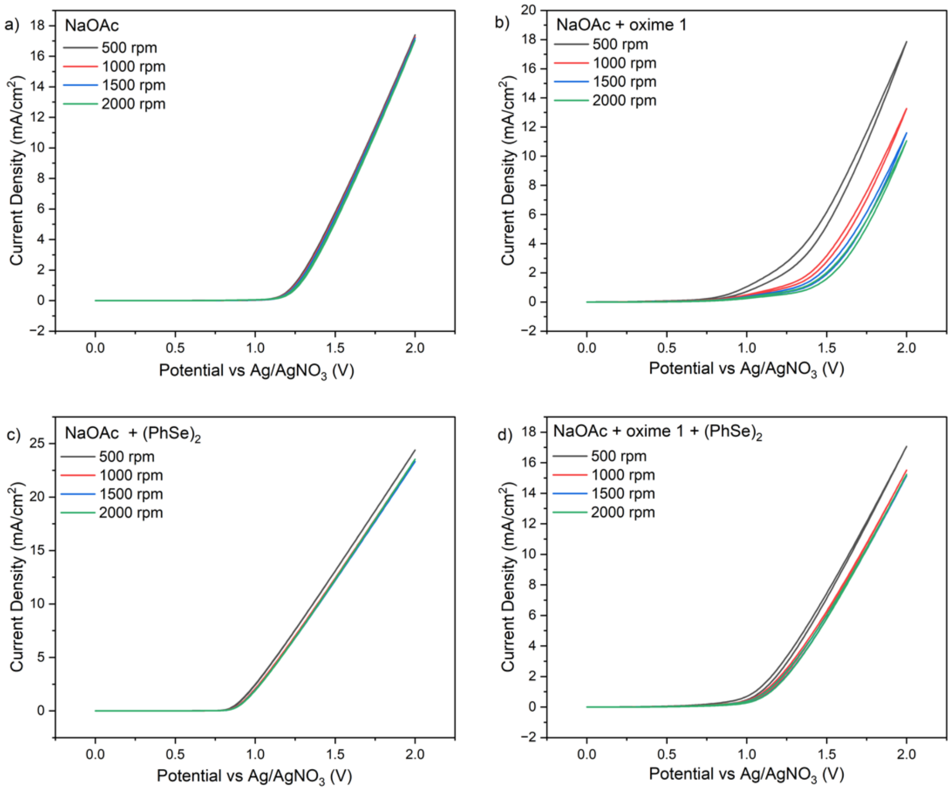

2.6. Characterization data for starting materials

1-(2-(Phenylethynyl)phenyl)ethan-1-one oxime (S1):

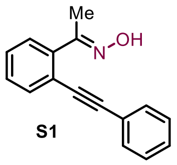

S1

^{1}H NMR (400 MHz, $CDCl_3$): δ 7.61 – 7.59 (m, 1H), 7.53 – 7.50 (m, 2H), 7.43 – 7.41 (m, 1H), 7.37 – 7.31 (m, 5H), 2.40 (s, 3H).

^{13}C NMR (101 MHz, $CDCl_3$): δ 157.6, 139.9, 133.2, 131.7, 128.7, 128.6, 128.5, 128.5, 128.4, 123.2, 121.8, 93.8, 88.1, 15.6.

The analytical data are in accordance with reported literature.[55]

1-(2-((4-Methoxyphenyl)ethynyl)phenyl)ethan-1-one (S2-1):

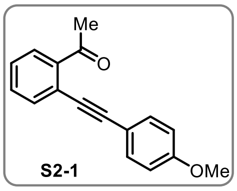

S2-1

^{1}H NMR (400 MHz, $CDCl_3$): δ 7.67 (dd, *J* = 7.5, 1.2 Hz, 1H), 7.52 (dd, *J* = 7.7, 1.4 Hz, 1H), 7.42 (d, *J* = 9.0 Hz, 2H), 7.35 (td, *J* = 7.5, 1.5 Hz, 1H), 7.29 – 7.24 (m, 1H), 6.80 (d, *J* = 9.0 Hz, 2H), 3.69 (s, 3H), 2.70 (s, 3H).

^{13}C NMR (101 MHz, $CDCl_3$): δ 199.7, 159.8, 140.1, 133.4, 132.8, 131.0, 128.5, 127.6, 121.8, 114.7, 113.9, 95.1, 87.3, 54.9, 29.6.

The analytical data are in accordance with reported literature.[56]

1-(2-((4-Methoxyphenyl)ethynyl)phenyl)ethan-1-one oxime (S2):

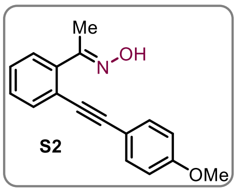

S2

^{1}H NMR (400 MHz, $CDCl_3$): δ 7.41 – 7.31 (m, 1H), 7.24 (d, *J* = 8.9 Hz, 2H), 7.20 – 7.10 (m, 3H), 6.64 (d, *J* = 9.0 Hz, 2H), 3.58 (s, 5H), 2.19 (s, 3H).

^{13}C NMR (101 MHz, $CDCl_3$): δ 159.9, 157.6, 139.8, 133.0, 132.9, 128.6, 128.4, 128.1, 122.1, 115.4, 114.1, 93.9, 87.0, 55.4, 15.6.

The analytical data are in accordance with reported literature.[57]

1-(2-(*p*-Tolylethynyl)phenyl)ethan-1-one (S3-1):

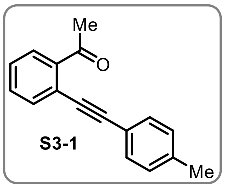

S3-1

^{1}H NMR (400 MHz, $CDCl_3$): δ 7.75 (dd, *J* = 7.8, 1.1 Hz, 1H), 7.62 (dd, *J* = 7.7, 0.9 Hz, 1H), 7.46 (ddd, *J* = 8.1, 6.9, 3.1 Hz, 3H), 7.39 (td, *J* = 7.6, 1.3 Hz, 1H), 7.18 (d, *J* = 7.9 Hz, 2H), 2.80 (s, 3H), 2.38 (s, 3H).

^{13}C NMR (101 MHz, $CDCl_3$): δ 200.7, 140.8, 139.2, 134.0, 131.5, 131.4, 129.4, 128.8, 128.2, 122.1, 119.9, 95.5, 88.0, 30.2, 21.7.

The analytical data are in accordance with reported literature.[56]

1-(2-(*p*-Tolylethynyl)phenyl)ethan-1-one oxime (S3):

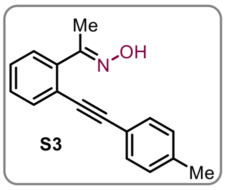

^{1}H NMR (400 MHz, $CDCl_3$): δ 9.41 (bs, 1H), 7.65 – 7.49 (m, 1H), 7.43 – 7.39 (m, 3H), 7.37 – 7.32 (m, 2H), 7.14 (d, *J* = 7.9 Hz, 2H), 2.40 (s, 3H), 2.35 (s, 3H).

^{13}C NMR (101 MHz, $CDCl_3$): δ 157.8, 139.9, 138.8, 133.1, 131.5, 129.2, 128.7, 128.4, 128.3, 122.0, 120.2, 21.7, 15.6.

The analytical data are in accordance with reported literature.[65]

1-(2-((3-Chlorophenyl)ethynyl)phenyl)ethan-1-one oxime (S4):

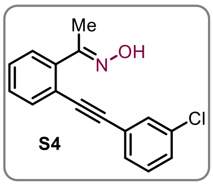

^{1}H NMR (400 MHz, $CDCl_3$): δ 9.11 (bs, 1H), 7.64 – 7.55 (m, 2H), 7.50 (dd, *J* = 2.5, 0.9 Hz, 1H), 7.43 – 7.35 (m, 3H), 7.32 – 7.28 (m, 2H), 2.38 (s, 1H). The NMR spectra show the presence of non-isolable impurities along with the desired product.

^{13}C NMR (101 MHz, $CDCl_3$): δ 157.2, 139.8, 134.3, 133.3, 133.3, 131.4, 130.0, 129.8, 129.7, 128.9, 128.4, 128.2,127.6, 92.1, 89.3, 15.6.

HRMS (ES$^+$): m/z calculated for [$C_{16}H_{13}ClNO$] [M+H]$^+$: 270.0686, measured: 270.0686.

1-(2-((4-Fluorophenyl)ethynyl)phenyl)ethan-1-one oxime (S5):

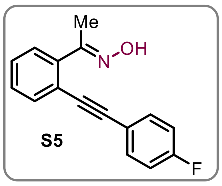

^{1}H NMR (400 MHz, $CDCl_3$): δ 8.98 (bs, 1H), 7.57 (dd, *J* = 5.8, 3.1 Hz, 1H), 7.53 – 7.44 (m, 2H), 7.38 (tdd, *J* = 7.0, 4.7, 2.4 Hz, 3H), 7.02 (t, *J* = 8.7 Hz, 2H), 2.37 (s, 3H).

^{13}C NMR (101 MHz, $CDCl_3$): δ 162.6 (d), 157.5, 139.8, 133.4 (d), 133.00, 128.6, 128.5, 128.3, 121.5, 119.3, 119.2, 115.7 (d), 92.5, 87.7, 15.4.

The analytical data are in accordance with reported literature.[65]

1-(2-((4-(Trifluoromethyl)phenyl)ethynyl)phenyl)ethan-1-one oxime (S6):

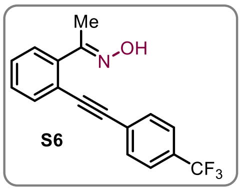

^{1}H NMR (400 MHz, $CDCl_3$): δ 9.51 (bs, 1H), 7.67 – 7.54 (m, 4H), 7.40 – 7.35 (m, 2H), 7.35 – 7.27 (m, 2H), 2.38 (s, 3H). The NMR spectra show the presence of non-isolable impurities along with the desired product.

^{13}C NMR (101 MHz, $CDCl_3$): δ 157.1, 140.1, 133.3, 132.9, 130.2, 130.0, 129.1, 128.8, 128.5, 128.2, 127.6, 125.2, 92.1, 90.5, 15.6.

HRMS (ES$^+$): m/z calculated for [$C_{17}H_{13}F_3NO$] [M+H]$^+$: 304.0949, measured: 304.0950.

Methyl 4-((2-acetylphenyl)ethynyl)benzoate (S7-1):

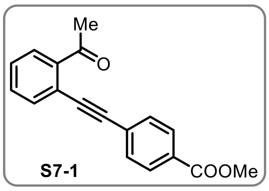

^{1}H NMR (400 MHz, CDCl$_3$): δ 8.02 (d, *J* = 8.3 Hz, 2H), 7.76 (dd, *J* = 7.7, 1.3 Hz, 1H), 7.67 – 7.57 (m, 3H), 7.49 (td, *J* = 7.5, 1.4 Hz, 1H), 7.42 (td, *J* = 7.6, 1.3 Hz, 1H), 3.92 (s, 3H), 2.76 (s, 3H).

^{13}C NMR (101 MHz, CDCl$_3$): δ 200.0, 166.5, 140.9, 134.3, 132.7, 131.7, 131.6, 130.1, 129.8, 129.8, 129.1, 128.9, 94.0, 91.5, 52.5, 30.0.

The analytical data are in accordance with reported literature.[66]

Methyl-4-((2-(1-(hydroxyimino)ethyl)phenyl)ethynyl)benzoate (S7):

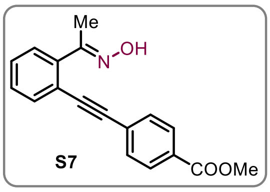

^{1}H NMR (400 MHz, CDCl$_3$): δ 8.01 (d, *J* = 8.2 Hz, 2H), 7.65 – 7.60 (m, 1H), 7.57 (d, *J* = 8.2 Hz, 2H), 7.42 (qd, *J* = 6.0, 3.7 Hz, 3H), 3.94 (s, 3H), 2.40 (s, 3H).

^{13}C NMR (101 MHz, CDCl$_3$): δ 166.7, 157.5, 139.9, 133.4, 131.6, 129.7, 129.6, 129.1, 128.9, 128.5, 127.9, 121.2, 92.9, 91.0, 52.4, 15.7.

HRMS (ES$^+$): m/z calculated for [$C_{18}H_{16}NO_3$] [M+H]$^+$: 294.1130, measured: 294.1130.

1-(2-((Trimethylsilyl)ethynyl)phenyl)ethan-1-one oxime (S8):

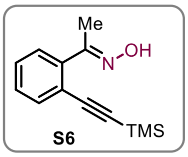

^{1}H NMR (400 MHz, CDCl$_3$): δ 7.54 – 7.49 (m, 1H), 7.39 – 7.28 (m, 3H), 2.34 (s, 9H).

^{13}C NMR (101 MHz, CDCl$_3$): δ 157.5, 140.4, 133.6, 128.7, 128.6, 128.3, 103.6, 99.3, 15.6, -0.1.

HRMS (ES$^+$): m/z calculated for [$C_{13}H_{18}NOS$] [M+H]$^+$: 232.1158, measured: 232.1158.

2-(Phenylethynyl)benzaldehyde oxime (S9):

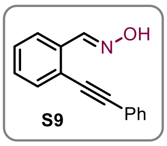

^{1}H NMR (400 MHz, CDCl$_3$): δ 8.90 (s, 1H), 8.79 (s, 1H), 7.93 – 7.83 (m, 1H), 7.63 – 7.50 (m, 4H), 7.45 – 7.31 (m, 6H).

^{13}C NMR (101 MHz, CDCl$_3$): δ 149.2, 133.0, 132.7, 131.7, 129.9, 128.83, 128.7, 128.6, 125.3, 123.3, 122.8, 95.2, 86.3.

The analytical data are in accordance with reported literature.[67]

2-((4-Methoxyphenyl)ethynyl)benzaldehyde oxime (S10):

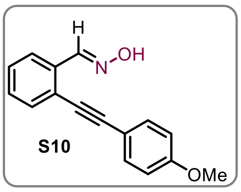

^{1}H NMR (400 MHz, CDCl$_3$): δ 8.76 (s, 1H), 8.59 (s, 1H), 7.86 (dd, *J* = 7.8, 1.3 Hz, 1H), 7.56 – 7.49 (m, 3H), 7.34 (td, *J* = 7.4, 1.5 Hz, 2H), 6.91 – 6.89 (m, 2H), 3.83 (s, 3H).

^{13}C NMR (101 MHz, CDCl$_3$): δ 160.0, 149.3, 133.2, 132.8, 132.6, 129.8, 128.4, 125.3, 123.7, 115.0, 114.2, 95.3, 85.1, 55.5.

The analytical data are in accordance with reported literature.[67]

2-(Thiophen-2-ylethynyl)benzaldehyde (S11-1):

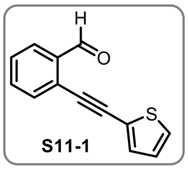
S11-1

^{1}H NMR (500 MHz, CDCl$_3$) δ 10.58 (s, 1H), 7.93 (ddd, *J* = 7.8, 1.4, 0.7 Hz, 2H), 7.61– 7.54 (m, 2H), 7.47 – 7.41 (m, 0H), 7.37 – 7.33 (m, 3H), 7.04 (dd, *J* = 5.1, 3.8 Hz, 2H).
^{13}C NMR (126 MHz, CDCl$_3$) δ 191.5, 135.7, 133.9, 133.0, 132.9, 128.8, 128.5, 127.4, 127.4, 126.5, 122.2, 89.7, 88.8.

The analytical data are in accordance with reported literature.[68]

2-(Thiophen-2-ylethynyl)benzaldehyde oxime (S11):

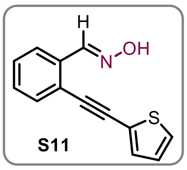
S11

^{1}H NMR (500 MHz, CDCl$_3$) δ 8.94 (s, 1H), 8.72 (s, 1H), 7.93 – 7.82 (m, 1H), 7.60 – 7.49 (m, 1H), 7.44 – 7.30 (m, 4H), 7.04 (dd, *J* = 5.2, 3.7 Hz, 1H).
^{13}C NMR (126 MHz, CDCl$_3$) δ δ 149.05, 132.85, 132.58, 132.52, 129.85, 128.86, 127.97, 127.33, 125.36, 122.94, 122.71, 89.98, 88.32.

The product is reported in the literature [30]

2-((Trimethylsilyl)ethynyl)benzaldehyde oxime (S13):

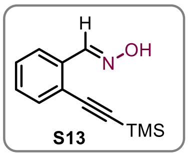
S13

^{1}H NMR (400 MHz, CDCl$_3$): δ 8.67 (s, 1H), 7.94 – 7.74 (m, 1H), 7.61 – 7.43 (m, 1H), 7.36 – 7.30 (m, 1H), 0.29 (s, 9H).

^{13}C NMR (101 MHz, CDCl$_3$): δ 149.2, 133.4, 133.2, 129.8, 129.0, 125.3, 123.3, 101.9, 100.9, -0.1.

Known but no analytical data [27]

HRMS (ES$^+$): m/z calculated for [$C_{12}H_{16}NOSi$] [M+H]$^+$: 218.1001, measured: 218.1001.

4,5-Dimethoxy-2-(phenylethynyl)benzaldehyde (S14-1):

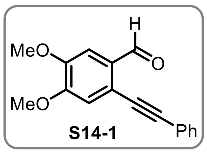
S14-1

^{1}H NMR (400 MHz, CDCl$_3$): δ 10.50 (s, 1H), 7.57 – 7.51 (m, 2H), 7.41 (s, 1H), 7.40 – 7.34 (m, 3H), 7.05 (s, 1H), 3.98 (s, 3H), 3.95 (s, 3H).

^{13}C NMR (101 MHz, CDCl$_3$): δ 190.6, 153.7, 149.8, 131.7, 130.3, 129.0, 128.6, 122.6, 121.7, 114.4, 108.3, 95.1, 84.9, 56.4, 56.2.

The analytical data are in accordance with reported literature.[68]

4,5-Dimethoxy-2-(phenylethynyl)benzaldehyde oxime (S14):

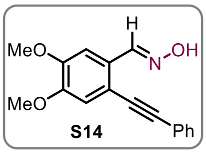

¹H NMR (400 MHz, $CDCl_3$): δ 8.67 (s, 1H), 7.63 – 7.46 (m, 2H), 7.38 – 7.35 (m, 4H), 7.00 (s, 1H), 3.94 (s, 3H), 3.93 (s, 3H).

¹³C NMR (101 MHz, $CDCl_3$): δ 150.5, 149.8, 149.2, 131.6, 128.7, 128.6, 126.6, 123.0, 116.6, 114.1, 107.0, 93.9, 86.3, 56.2, 56.1.

HRMS (ES⁺): m/z calculated for [C17H15NO3] $[M+H]^+$: 282.1130, measured: 282.1129.

Known but no analytical data[30]

1-(2-Bromo-4,5-dimethoxyphenyl)ethan-1-one (15-1):

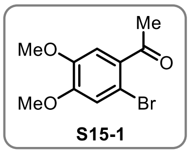

¹H NMR (400 MHz, $CDCl_3$): δ 7.11 (s, 1H), 7.01 (s, 1H), 3.88 (s, 3H), 3.86 (s, 3H), 2.64 (s, 3H).

¹³C NMR (101 MHz, $CDCl_3$): δ 199.5, 151.7, 148.1, 132.7, 116.4, 112.7, 111.8, 56.4, 56.2, 30.5.

The analytical data are in accordance with reported literature.[69]

1-(4,5-Dimethoxy-2-(phenylethynyl)phenyl)ethan-1-one (S15-2):

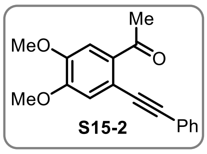

¹H NMR (400 MHz, $CDCl_3$): δ 7.54 (dd, *J* = 6.6, 3.0 Hz, 2H), 7.43 (s, 1H), 7.38 (dd, *J* = 4.9, 1.5 Hz, 3H), 7.06 (s, 1H), 3.97 (s, 3H), 3.95 (s, 3H), 2.86 (s, 3H).

¹³C NMR (101 MHz, $CDCl_3$): δ 198.6, 151.7, 149.2, 133.6, 131.4, 128.8, 128.7, 123.1, 116.2, 115.6, 111.5, 94.7, 89.3, 56.4, 56.2, 30.5.

The analytical data are in accordance with reported literature.[70]

1-(4,5-Dimethoxy-2-(phenylethynyl)phenyl)ethan-1-one oxime (S15):

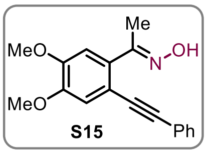

¹H NMR (400 MHz, $CDCl_3$): δ 7.50 – 7.48 (m, 2H), 7.33 (qd, *J* = 4.9, 2.4 Hz, 3H), 7.05 (s, 1H), 6.93 (s, 1H), 3.93 (s, 3H), 3.92 (s, 3H), 2.42 (s, 3H).

¹³C NMR (101 MHz, $CDCl_3$): δ 158.1, 149.3, 149.1, 133.4, 131.4, 128.5, 128.4, 123.4, 115.3, 114.1, 111.4, 92.6, 88.3, 56.2, 56.2, 15.8.

HRMS (ES⁺): m/z calculated for $[C_{18}H_{18}NO_3]$ $[M+H]^+$: 296.1287, measured: 296.1289.

4-Methyl-N'-(1-(2-(phenylethynyl)phenyl)ethylidene)benzenesulfonohydrazide (S16):

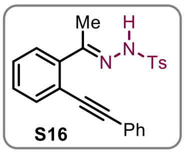

¹H NMR (400 MHz, $CDCl_3$): δ 7.83 – 7.76 (m, 2H), 7.69 – 7.62 (m, 1H), 7.42 – 7.29 (m, 8H), 7.25 (dt, *J* = 8.4, 1.7 Hz, 2H), 2.33 (s, 3H), 2.23 (s, 3H).

¹³C NMR (101 MHz, $CDCl_3$): δ 154.8, 143.8, 141.4, 136.8, 132.9, 131.7, 131.4, 129.6, 128.4, 127.8, 126.7, 122.3, 120.6, 93.9, 85.8, 24.4, 21.6.

The analytical data are in accordance with reported literature.[71]

N'-(1-(2-((4-Methoxyphenyl)ethynyl)phenyl)ethylidene)-4-methylbenzenesulfonohydrazide (S17):

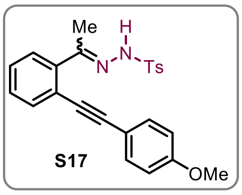

S17

^{1}H NMR (400 MHz, $CDCl_3$): (mixture of two non-isolable two stereoisomers detected) major stereoisomer: δ 8.01 (s, 1H), 7.75 (d, *J* = 8.3 Hz, 2H), 7.51 – 7.46 (m, 1H), 7.37 – 7.23 (m, 2H), 7.11 – 7.02 (m, 5H), 6.91 – 6.75 (m, 2H), 3.84 (s, 3H), 2.27 (s, 3H), 2.21 (s, 3H).

^{13}C NMR (101 MHz, $CDCl_3$): δ 160.0, 155.0, 143.7, 136.5, 135.5, 133.2, 132.9, 129.7, 129.7, 129.6, 128.1, 127.8, 121.0, 114.4, 114.0, 94.2, 84.7, 55.5, 24.4, 21.6.

The analytical data of major stereoisomer is in accordance with reported literature.[71]

4-Methyl-N'-(2-(phenylethynyl)benzylidene)benzenesulfonohydrazide (S18):

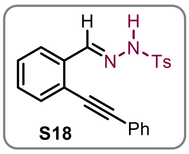

S18

^{1}H NMR (400 MHz, $CDCl_3$): δ 8.50 (s, 1H), 8.32 (s, 1H), 7.94 – 7.76 (m, 3H), 7.54 – 7.35 (m, 3H), 7.27 – 7.14 (m, 7H), 2.31 (s, 3H). Trace amount of non-isolable impurity is present alongside desired product.

^{13}C NMR (101 MHz, $CDCl_3$): δ 146.0, 144.4, 135.4, 134.2, 132.5, 131.7, 130.0, 129.8, 128.9, 128.6, 128.6, 128.1, 125.5, 123.4, 122.6, 95.4, 86.1, 21.7.

The analytical data of major stereoisomer is in accordance with reported literature.[72]

N'-(2-((4-Methoxyphenyl)ethynyl)benzylidene)-4-methylbenzenesulfonohydrazide (S19):

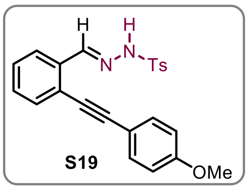

S19

^{1}H NMR (400 MHz, $CDCl_3$): δ 8.43 (bs, 1H), 8.40 (s, 1H), 7.97 – 7.85 (m, 3H), 7.46 (d, *J* = 8.5 Hz, 3H), 7.30 (dt, *J* = 16.9, 8.2 Hz, 4H), 6.84 (d, *J* = 7.9 Hz, 2H), 3.81 (s, 3H), 2.39 (s, 3H).

^{13}C NMR (101 MHz, $CDCl_3$): δ 160.1, 146.2, 144.5, 135.4, 133.9, 133.2, 132.3, 130.1, 129.9, 128.3, 128.1, 125.5, 123.8, 114.7, 114.2, 95.5, 84.8, 55.5, 21.7.

The analytical data are in accordance with reported literature.[72]

6-phenylhex-5-yn-2-one (S20)

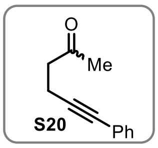

S20

^{1}H NMR (400 MHz, $CDCl_3$) δ 7.45 – 7.34 (m, 2H), 7.29 (td, *J* = 2.8, 0.6 Hz, 3H), 2.81 – 2.73 (t, 2H), 2.72 – 2.63 (t, 2H), 2.20 (s, 3H).

^{13}C NMR (101 MHz, $CDCl_3$) δ 206.63, 131.61, 128.26, 127.80, 123.65, 88.58, 81.02, 42.53, 29.96, 14.04.

The analytical data are in accordance with reported literature.[60]

6-phenylhex-5-yn-2-one oxime (S21)

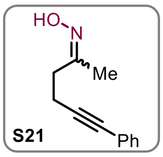

S21

^{1}H NMR (400 MHz, $CDCl_3$) Major isomer: δ 9.14 (s, 1H), 7.50 – 7.21 (m, 3H), 7.24 – 6.88 (m, 2H), 2.53 (t, *J* = 7.3 Hz, 2H), 2.41 (t, *J* = 7.3 Hz, 2H), 1.85 (s, 3H).

^{13}C NMR (101 MHz, $CDCl_3$) Major isomer: δ 156.91, 131.67, 128.23, 127.76, 123.76, 88.65, 81.58, 35.05, 16.95, 13.90.

HRMS (ES+): m/z calculated for $[C_{12}H_{14}NO]$ $[M+H]^+$: 188.1075, measured: 188.1083.

1-(2-(phenylethynyl)pyridin-3-yl)ethan-1-one oxime (S25)

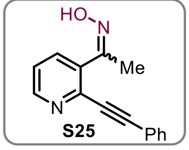

S25

^{1}H NMR (400 MHz, CDCl3): major isomer δ 10.15 (s, 1H), 8.63 (dd, *J* = 4.8, 1.7 Hz, 1H), 7.73 (dd, *J* = 7.9, 1.7 Hz, 1H), 7.62 – 7.51 (m, 2H), 7.40 – 7.22 (m, 4H), 2.45 (s, 3H).

^{13}C NMR (101 MHz, CDCl3): δ 155.54, 149.90, 141.29, 136.38, 135.74, 132.07, 129.31, 128.49, 122.73, 122.17, 93.80, 87.67, 15.34.

HRMS (ES+): m/z calculated for $[C_{15}H_{13}N_2O]$ $[M+H]^+$: 237.1027, measured: 237.1037.

4-Methyl-N'-((E)-2-((E)-styryl)benzylidene)benzenesulfonohydrazide (34a):

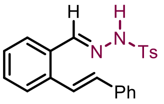

34a

^{1}H NMR (400 MHz, $CDCl_3$): δ 8.60 (s, 1H), 8.18 (s, 1H), 7.88 (t, *J* = 8.4 Hz, 2H), 7.68 (d, *J* = 7.6 Hz, 1H), 7.57 (dd, *J* = 18.8, 11.6 Hz, 4H), 7.41 – 7.34 (m, 3H), 7.29 (dt, *J* = 15.8, 6.5 Hz, 2H), 7.21 (d, *J* = 8.1 Hz, 2H), 6.91 (d, *J* = 16.1 Hz, 1H), 2.37 (s, 3H).

^{13}C NMR (101 MHz, $CDCl_3$): δ 147.1, 144.2, 137.4, 137.2, 135.3, 132.6, 130.5, 130.3, 129.7, 128.9, 128.2, 128.1, 128.1, 127.6, 127.0, 126.9, 125.8, 21.7.

The analytical data are in accordance with reported literature.[73]

1-(2-(Phenylethynyl)phenyl)ethan-1-one O-methyl oxime (S20):

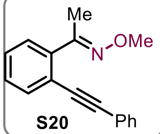

S20

^{1}H NMR (400 MHz, $CDCl_3$): δ 7.64 – 7.62 (m, 1H), 7.58 – 7.55 (m, 2H), 7.49 – 7.46 (m, 1H), 7.41 – 7.38 (m, 5H), 4.08 (s, 3H), 2.43 (s, 3H).

^{13}C NMR (101 MHz, $CDCl_3$): δ 156.8, 140.1, 133.0, 131.5, 128.5, 128.5, 128.5, 128.5, 128.5, 128.5, 123.3, 121.7, 93.7, 88.2, 62.0, 16.1.

The analytical data are in accordance with reported literature.[74]

3-(2-(1-(2-Tosylhydrazineylidene)ethyl)phenyl)prop-2-yn-1-yl benzoate (34b):

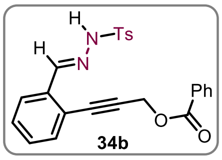

^{1}H NMR (400 MHz, $CDCl_3$): δ 8.70 (s, 1H), 8.34 (s, 1H), 8.12 – 8.05 (m, 2H), 7.91–7.87 (m, 4H), 7.63 – 7.57 (m, 1H), 7.48 (dd, *J* = 10.6, 4.8 Hz, 2H), 7.44 – 7.38 (m, 1H), 7.33 – 7.28 (m, 3H), 5.14 (s, 2H), 2.39 (s, 3H). Trace amount of non-isolable impurity is present alongside desired product.

^{13}C NMR (101 MHz, $CDCl_3$): δ 166.4, 145.7, 144.2, 135.5, 135.0, 133.7, 132.6, 129.9, 129.8, 129.8, 129.2, 128.7, 128.2, 128.0, 125.4, 122.1, 89.2, 83.5, 53.4, 21.7.

HRMS (ES$^+$): m/z calculated for $[C_{24}H_{20}N_2O_4S]$ $[M+H]^+$: 433.1222, measured: 433.1200.

1,2-Di-p-tolyldiselane (Se1):

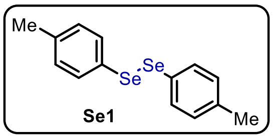

^{1}H NMR (400 MHz, $CDCl_3$): δ 7.51 (d, *J* = 8.1 Hz, 4H), 7.09 (d, *J* = 7.9 Hz, 4H), 2.35 (s, 6H).

^{13}C NMR (101 MHz, $CDCl_3$): δ 138.1, 133.5, 132.4, 130.1, 21.3.

^{77}Se NMR (76 MHz, $CDCl_3$): δ 473.44.

The analytical data are in accordance with reported literature.[75]

1,2-Bis(4-(trifluoromethyl)phenyl)diselane (Se2):

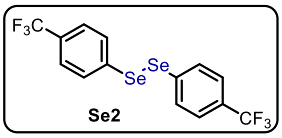

^{1}H NMR (400 MHz, $CDCl_3$): δ 7.71 (d, *J* = 8.5 Hz, 4H), 7.53 (d, *J* = 8.6 Hz, 4H).

^{13}C NMR (101 MHz, $CDCl_3$): δ 135, 132.9, 130.8, 126.3 (q, *J* = 3.7 Hz).

^{19}F NMR (376 MHz, $CDCl_3$) δ -62.64.

^{77}Se NMR (76 MHz, $CDCl_3$): δ 451.74.

The analytical data are in accordance with reported literature.[76]

1,2-Bis(3-fluorophenyl)diselane (Se3):

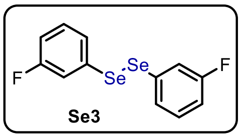

^{1}H NMR (400 MHz, $CDCl_3$): δ 7.34 (ddd, *J* = 9.6, 4.5, 1.3 Hz, 4H), 7.27 – 7.19 (m, 2H), 6.98 – 6.90 (m, 2H).

^{13}C NMR (101 MHz, $CDCl_3$): δ 162.9 (d, *J* = 250.6 Hz), 132.1 (d, *J* = 6.4 Hz), 130.6 (d, *J* = 8.0 Hz), 126.6 (d, *J* = 3.1 Hz), 118.2 (d, *J* = 23.1 Hz), 115.0 (d, *J* = 21.3 Hz).

^{19}F NMR (376 MHz, $CDCl_3$) δ -111.14.

^{77}Se NMR (76 MHz, $CDCl_3$): δ 464.06.

HRMS (ES+): m/z calculated for $[C_{12}H_8F_2Se_2]$ $[M^+]$: 349.8925, measured: 349.8929.

1,2-Di(naphthalen-2-yl)diselane (Se4):

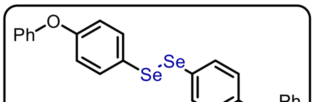

Se4

There are non-isolable impurities alongside desired product. **^{1}H NMR (400 MHz, $CDCl_3$)** δ 7.58 – 7.53 (m, 2H), 7.47 – 7.41 (m, 2H), 7.39 – 7.31 (m, 4H), 7.18 – 7.09 (m, 2H), 7.06 – 6.99 (m, 4H), 6.95 – 6.87 (m, 4H).

^{13}C NMR (101 MHz, $CDCl_3$) δ 158.1, 156.6, 134.9, 130.0, 123.8, 123.4, 119.5, 119.2.

^{77}Se NMR (76 MHz, $CDCl_3$): δ 491.67.

HRMS (ES+): m/z calculated for $[C_{24}H_{18}NO_2Se_2]$ $[M^+]$: 497.9637, measured: 497.9633.

1,2-Di(naphthalen-2-yl)diselane (Se5):

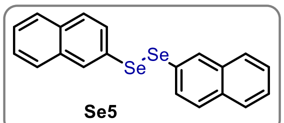

Se5

There are non-isolable impurities alongside desired product. **^{1}H NMR (400 MHz, $CDCl_3$)** δ 8.24 (d, *J* = 8.4 Hz, 1H), 7.89 – 7.77 (m, 3H), 7.54 – 7.49 (m, 1H), 7.45 (t, *J* = 7.7 Hz, 1H), 7.34 – 7.27 (m, 1H).

^{13}C NMR (101 MHz, $CDCl_3$) δ 134.2, 132.3, 129.9, 128.7, 128.1, 127.2, 126.8, 126.4, 126.2, 125.8.

^{77}Se NMR (76 MHz, $CDCl_3$): δ 427.87.

HRMS (ES+): m/z calculated for $[C_{20}H_{14}Se_2]$ $[M^+]$: 413.9426, measured: 413.9433.

2.7. Characterization data for isoquinoline *N*-oxide/imide derivatives

1-Methyl-3-phenyl-4-(phenylselanyl)isoquinoline 2-oxide (3):

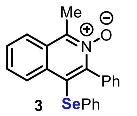

3

Following the **GP5** with substrate **S1**, a 7 mL reaction mixture (0.28 mmol) was collected after flow electrolysis. After the standard work-up, the volatile solvent was removed under reduced pressure, and the residue was purified by flash column chromatography to afford the product as a white solid (93 mg, 85% yield).

Melting point: 213-215 °C.

^{1}H NMR (400 MHz, $CDCl_3$): δ 8.35 (d, *J* = 8.2 Hz, 1H), 7.91 (d, *J* = 8.1 Hz, 1H), 7.50 (dt, *J* = 14.5, 7.0 Hz, 2H), 7.32 (m, 3H), 7.19 (m, 2H), 7.11 – 6.86 (m, 5H), 2.87 (s, 3H).

^{13}C NMR (101 MHz, $CDCl_3$): δ 152.30, 147.2, 135.6, 132.1, 130.7, 130.1, 129.6, 129.43, 129.3, 129.2, 129.1, 128.7, 128.5, 128.2, 126.7, 124.9, 124.4, 13.8.

^{77}Se NMR (76 MHz, $CDCl_3$): δ 333.38.

HRMS (ESI): m/z calculated for $[C_{22}H_{18}NOSe]$ $[M+H^+]$: 392.0554, measured: 392.0561.

FTIR (Neat): 3049, 2976, 1784, 1570, 1440, 1223, 1124, 1067, 928, 770, 754 cm^{-1}.

3-(4-Methoxyphenyl)-1-methyl-4-(phenylselanyl)isoquinoline 2-oxide (5):

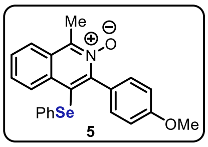

Following the **GP5** with substrate **S2**, a 7 mL reaction mixture (0.28 mmol) was collected after flow electrolysis. After the standard work-up, the volatile solvent was removed under reduced pressure, and the residue was purified by flash column chromatography to afford the product as a white solid (86 mg, 73% yield).

Melting point: 170-173 °C.

^{1}H NMR (500 MHz, CDCl$_3$): δ 8.43 (d, *J* = 9.2 Hz, 1H), 7.99 (d, *J* = 10.4 Hz, 1H), 7.62 (ddd, *J* = 8.4, 6.9, 1.3 Hz, 1H), 7.54 (ddd, *J* = 8.2, 6.9, 1.2 Hz, 1H), 7.22 (d, *J* = 8.9 Hz, 2H), 7.17 – 7.06 (m, 3H), 7.05 – 7.02 (m, 2H), 6.93 (d, *J* = 8.9 Hz, 2H), 3.83 (s, 3H), 2.96 (s, 3H).

^{13}C NMR (126 MHz, CDCl$_3$): δ 159.8, 152.2, 147.2, 132.3, 131.0, 130.8, 130.1, 129.7, 129.3, 129.1, 129.1, 128.4, 127.8, 126.7, 125.2, 124.5, 113.8, 55.3, 13.9.

^{77}Se NMR (76 MHz, CDCl$_3$): δ 335.80.

HRMS (ESI): m/z calculated for [$C_{23}H_{20}NO_2Se$] [M+H$^+$]: 422.0667, measured: 422.0659.

FTIR (Neat): 3000, 1716, 1474, 1337, 1277, 1138, 1019, 818, 743 cm^{-1}.

1-Methyl-4-(phenylselanyl)-3-(p-tolyl)isoquinoline 2-oxide (6):

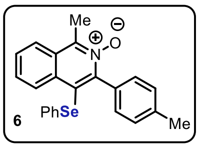

Following the **GP5** with substrate **S3**, a 7 mL reaction mixture (0.28 mmol) was collected after flow electrolysis. After the standard work-up, the volatile solvent was removed under reduced pressure, and the residue was purified by flash column chromatography to afford the product as a semi block solid (87 mg, 77% yield).

^{1}H NMR (400 MHz, CDCl$_3$): δ 8.44 (d, *J* = 8.3 Hz, 1H), 8.02 (d, *J* = 8.4 Hz, 1H), 7.65 (dd, *J* = 11.2, 4.0 Hz, 1H), 7.56 (t, *J* = 7.6 Hz, 1H), 7.32 – 7.18 (m, 4H), 7.17 – 7.02 (m, 4H), 2.99 (s, 3H), 2.44 (s, 3H).

^{13}C NMR (101 MHz, CDCl$_3$): δ 152.5, 147.3, 138.6, 132.7, 132.3, 130.7, 130.0, 129.7, 129.4, 129.3, 129.1, 129.1, 128.9, 128.5, 21.6, 13.9.

^{77}Se NMR (76 MHz, CDCl$_3$): δ 303.00.

HRMS (ESI): m/z calculated for [$C_{23}H_{20}NOSe$] [M+H$^+$]: 406.0710, measured: 406.0712.

3-(3-Chlorophenyl)-1-methyl-4-(phenylselanyl)isoquinoline 2-oxide (7):

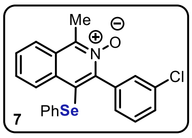

Following the **GP5** with substrate **S4**, a 7 mL reaction mixture (0.28 mmol) was collected after flow electrolysis. After the standard work-up, the volatile solvent was removed under reduced pressure, and the residue was purified by flash column chromatography to afford the product as ash grey solid (96 mg, 81% yield).

Melting point: 172-175 °C.

^{1}H NMR (400 MHz, CDCl$_3$): ^{1}H NMR (400 MHz, CDCl$_3$) δ 8.52 – 8.50 (m, 1H), 8.01 (d, *J* = 8.3 Hz, 1H), 7.69 – 7.65 (m, 1H), 7.62 – 7.58 (m, 1H), 7.39 – 7.30 (m, 2H), 7.18 – 7.09 (m, 5H), 7.02 – 7.00 (m, 2H), 2.96 (s, 3H).

^{13}C NMR (101 MHz, CDCl$_3$): δ 150.7, 147.3, 137.0, 134.0, 131.7, 130.8, 130.6, 129.8, 129.7, 129.5, 129.4, 129.3, 128.8, 128.5, 127.8, 127.1, 125.5, 13.8.

^{77}Se NMR (76 MHz, CDCl$_3$): δ 334.12.

HRMS (ESI): m/z calculated for [$C_{22}H_{17}ClNOSe$] [M+H$^+$]: 426.0164, measured: 426.0158.

FTIR (Neat): 2950, 1745, 1560, 1474, 1392, 1283, 1140, 912, 764 cm^{-1}.

3-(4-Fluorophenyl)-1-methyl-4-(phenylselanyl)isoquinoline 2-oxide (8):

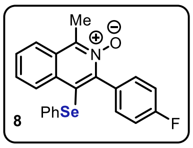

Following the **GP5** with substrate **S5**, a 7 mL reaction mixture (0.28 mmol) was collected after flow electrolysis. After the standard work-up, the volatile solvent was removed under reduced pressure, and the residue was purified by flash column chromatography to afford the product as white ash solid (71 mg, 62% yield).

Melting point: 213-215 °C.

^{1}H NMR (400 MHz, CDCl$_3$): δ 8.51 (d, *J* = 8.3 Hz, 1H), 8.04 (d, *J* = 8.3 Hz, 1H), 7.65 (dt, *J* = 15.0, 7.2 Hz, 2H), 7.26 (dd, *J* = 14.6, 6.5 Hz, 2H), 7.18 – 7.06 (m, 5H), 7.03 (d, *J* = 6.7 Hz, 2H), 2.98 (s, 3H).

^{13}C NMR (101 MHz, CDCl$_3$): δ 162.8 (d), 151.3, 147.3, 130.0, 131.6 (d), 131.4, 131.4, 130.9, 130.3, 129.7, 129.4, 129.4, 129.3, 128.6, 126.9, 125.4, 124.5, 115.3 (d), 13.9.

^{19}F NMR (376 MHz, CDCl$_3$): δ -112.50.

^{77}Se NMR (76 MHz, CDCl$_3$): δ 334.79.

HRMS (ESI): m/z calculated for [$C_{22}H_{17}NOSe$] [M+H$^+$]: 410.0459, measured: 410.0461.

FTIR (Neat): 2980, 2385, 1750, 1593, 1468, 1335, 1132, 856, 767 cm^{-1}.

1-Methyl-4-(phenylselanyl)-3-(4-(trifluoromethyl)phenyl)isoquinoline 2-oxide (9):

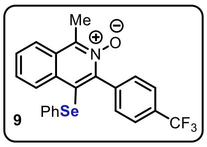

Following the **GP5** with substrate **S6**, a 7 mL reaction mixture (0.28 mmol) was collected after flow electrolysis. After the standard work-up, the volatile solvent was removed under reduced pressure, and the residue was purified by flash column chromatography to afford the product as brown sticky solid (85 mg, 66% yield).

^{1}H NMR (400 MHz, CDCl$_3$): δ 8.57 – 8.44 (m, 1H), 8.03 (d, *J* = 8.3 Hz, 1H), 7.69 (ddd, *J* = 8.4, 7.0, 1.2 Hz, 1H), 7.65 – 7.59 (m, 3H), 7.34 (d, *J* = 8.0 Hz, 2H), 7.18 – 7.06 (m, 3H), 7.02 – 6.95 (m, 2H), 2.96 (s, 3H).

^{13}C NMR (101 MHz, CDCl$_3$): δ 150.7, 147.5, 138.9, 131.7, 130.9, 130.7, 130.6, 130.4, 129.6 (q), 129.5, 128.7, 127.2, 125.5, 125.4, 125.2 (q), 124.6, 122.8, 13.8.

^{19}F NMR (376 MHz, $CDCl_3$): δ -62.69.

^{77}Se NMR (76 MHz, $CDCl_3$): δ 333.78.

HRMS (ESI): m/z calculated for [$C_{23}H_{17}F_3NOSe$] [M+H$^+$]: 460.0427, measured: 460.0425.

FTIR (Neat): 2963, 2349, 1824, 1358, 1321, 1063, 758 cm^{-1}.

3-(4-(Methoxycarbonyl)phenyl)-1-methyl-4-(phenylselanyl)isoquinoline 2-oxide (10):

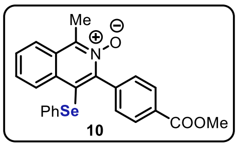

Following the **GP5** with substrate **S7**, a 7 mL reaction mixture (0.28 mmol) was collected after flow electrolysis. After the standard work-up, the volatile solvent was removed under reduced pressure, and the residue was purified by flash column chromatography to afford the product as light yellow sticky solid (89 mg, 71% yield).

^{1}H NMR (500 MHz, $CDCl_3$): δ 8.49 (d, *J* = 7.2 Hz, 1H), 8.04 (dd, *J* = 22.3, 7.0 Hz, 3H), 7.60 (dd, *J* = 41.7, 35.0 Hz, 2H), 7.36 – 7.20 (m, 2H), 7.19 – 6.89 (m, 5H), 3.93 (s, 3H), 2.96 (s, 3H).

^{13}C NMR (101 MHz, $CDCl_3$) δ 166.9, 151.3, 147.4, 140.1, 131.9, 130.9, 130.3, 130.2, 129.8, 129.7, 129.6, 129.5, 129.5, 129.4, 128.7, 127.0, 125.0, 124.5, 52.3, 13.8.

^{77}Se NMR (76 MHz, $CDCl_3$): δ 331.37.

HRMS (ESI): m/z calculated for [$C_{24}H_{20}NOSe$] [M+H$^+$]: 450.0608, measured: 450.0613.

1-Methyl-4-(phenylselanyl)isoquinoline 2-oxide (11):

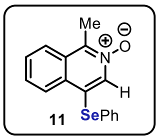

Following the **GP5** with substrate **S8**, a 7 mL reaction mixture (0.28 mmol) was collected after flow electrolysis. After the standard work-up, the volatile solvent was removed under reduced pressure, and the residue was purified by flash column chromatography to afford the product as colorless sticky solid (39 mg, 44% yield).

^{1}H NMR (400 MHz, $CDCl_3$): δ 8.24 (s, 1H), 8.17 (d, *J* = 7.9 Hz, 1H), 7.95 (d, *J* = 8.4 Hz, 1H), 7.70 – 7.56 (m, 2H), 7.49 (dd, *J* = 7.5, 1.8 Hz, 2H), 7.35 – 7.26 (m, 3H), 2.87 (s, 3H).

^{13}C NMR (101 MHz, $CDCl_3$): δ 145.3, 139.7, 133.8, 130.0, 129.7, 129.5, 129.0, 128.8, 128.7, 128.1, 127.2, 126.4, 124.7, 13.1.

^{77}Se NMR (76 MHz, $CDCl_3$): δ 339.91.

HRMS (ESI): m/z calculated for [$C_{16}H_{14}NOSe$] [M+H$^+$]: 316.0241, measured: 316.0240.

FTIR (Neat): 2992, 2932, 1606, 1512, 1336, 1286, 1011, 763 cm-1.

4-(Benzylselanyl)-1-methyl-3-phenylisoquinoline 2-oxide (12):

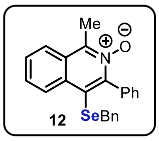

Following the **GP5** with substrate **S1**, a 7 mL reaction mixture (0.28 mmol) was collected after flow electrolysis. After the standard work-up, the volatile solvent

was removed under reduced pressure, and the residue was purified by flash column chromatography to afford the product as white sticky solid (88 mg, 78% yield).

^{1}H NMR (400 MHz, $CDCl_3$): δ 8.59 – 8.38 (m, 1H), 8.07 – 7.89 (m, 1H), 7.75 – 7.56 (m, 2H), 7.53 – 7.37 (m, 3H), 7.16 – 6.99 (m, 5H), 6.89 – 6.71 (m, 2H), 3.82 (s, 2H), 2.93 (s, 3H).

^{13}C NMR (101 MHz, $CDCl_3$) δ 152.2, 146.6, 137.6, 135.6, 131.0, 129.7, 129.4, 129.0, 128.9, 128.7 , 128.5, 128.4, 128.1, 128.0, 127.1, 124.6, 124.5, 33.3, 13.7.

^{77}Se NMR (76 MHz, $CDCl_3$): δ 300.89.

HRMS (ESI): m/z calculated for [$C_{23}H_{20}NOSe$] [M+H$^+$]: 406.0710, measured 406.0713.

FTIR (Neat): 2910, 2850, 1641, 1493, 1339, 1285, 1204, 1090, 837, 756 cm-1.

4-(Benzylselanyl)-1-methyl-3-(p-tolyl)isoquinoline 2-oxide (13):

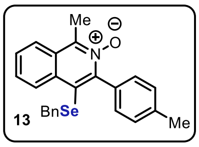

Following the **GP5** with substrate **S3**, a 7 mL reaction mixture (0.28 mmol) was collected after flow electrolysis. After the standard work-up, the volatile solvent was removed under reduced pressure, and the residue was purified by flash column chromatography to afford the product as white ash solid (84 mg, 72% yield).

Melting point: 173-175 °C.

^{1}H NMR (400 MHz, $CDCl_3$): δ 8.48 (d, J = 7.9 Hz, 1H), 7.98 (d, J = 8.0 Hz, 1H), 7.63 (dd, J = 16.6, 7.7 Hz, 2H), 7.30 – 7.18 (m, 2H), 7.10 (d, J = 5.7 Hz, 3H), 6.98 (d, J = 7.8 Hz, 2H), 6.79 (d, J = 5.4 Hz, 2H), 3.83 (s, 2H), 2.92 (s, 3H), 2.44 (s, 3H).

^{13}C NMR (101 MHz, $CDCl_3$): δ 152.5, 146.4, 138.6, 137.7, 132.8, 131.0, 129.6, 129.5, 128.9, 128.9, 128.8, 128.4, 128.2, 127.1, 124.6, 124.5, 33.3, 21.6, 13.7.

^{77}Se NMR (76 MHz, $CDCl_3$): δ 302.11.

HRMS (ESI): m/z calculated for [$C_{24}H_{22}NOSe$] [M+H$^+$]: 420.0867, measured: 420.0870.

FTIR (Neat): 2900, 1748, 1491, 1337, 1290, 1109, 912, 785, 752 cm-1.

4-(Benzylselanyl)-3-(4-fluorophenyl)-1-methylisoquinoline 2-oxide (14):

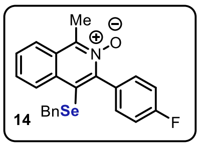

Following the **GP5** with substrate **S5**, a 7 mL reaction mixture (0.28 mmol) was collected after flow electrolysis. After the standard work-up, the volatile solvent was removed under reduced pressure, and the residue was purified by flash column chromatography to afford the product as white ash solid (82 mg, 69% yield).

Melting point: 162-164 °C.

^{1}H NMR (400 MHz, $CDCl_3$): δ 8.53 – 8.51 (m, 1H), 8.02 – 8.00 (m, 1H), 7.71 – 7.63 (m, 2H), 7.14 – 7.05 (m, 5H), 6.97 (ddd, J = 6.5, 5.2, 2.5 Hz, 2H), 6.76 – 6.74 (m, 2H), 3.83 (s, 2H), 2.93 (s, 3H).

^{13}C NMR (101 MHz, CDCl$_3$): δ 162.8 (d), 151.4, 146.8, 137.7, 131.7 (d), 131.6, 131.6, 131.0, 129.6, 129.2, 129.2, 128.7, 128.6, 128.3, 127.3, 124.9, 124.7, 115.2 (d), 33.3, 13.8.

^{19}F NMR (376 MHz, CDCl$_3$): δ -112.73.

^{77}Se NMR (76 MHz, CDCl$_3$): δ 304.73.

HRMS (ESI): m/z calculated for [$C_{23}H_{19}NOFSe$] [M+H$^+$]: 424.0616, measured: 424.0616.

4-(Benzylselanyl)-1-methylisoquinoline 2-oxide (15):

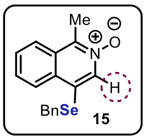

Following the **GP5** with substrate **S8**, a 7 mL reaction mixture (0.28 mmol) was collected after flow electrolysis. After the standard work-up, the volatile solvent was removed under reduced pressure, and the residue was purified by flash column chromatography to afford the product as white ash sticky solid (36 mg, 39% yield).

^{1}H NMR (400 MHz, CDCl$_3$): δ 8.33 (s, 1H), 8.13 – 8.10 (m, 1H), 7.91 (dd, *J* = 8.4, 0.6 Hz, 1H), 7.65 – 7.60 (m, 1H), 7.59 – 7.52 (m, 1H), 7.18 (ddd, *J* = 8.1, 4.9, 2.8 Hz, 3H), 7.13 (dd, *J* = 7.4, 1.8 Hz, 2H), 4.13 (s, 2H), 2.86 (s, 3H).

^{13}C NMR (101 MHz, CDCl$_3$): δ 145.3, 140.2, 137.1, 130.5, 129.3, 128.9, 128.8, 128.7, 128.5, 127.5, 127.5, 125.2, 124.6. 32.7, 13.0.

^{77}Se NMR (76 MHz, CDCl$_3$): δ 290.80.

HRMS (ESI): m/z calculated for [$C_{17}H_{16}NOSe$] [M+H$^+$]: 330.0397, measured: 330.0403.

1-Methyl-3-(p-tolyl)-4-((4-(trifluoromethyl)phenyl)selanyl)isoquinoline 2-oxide (16):

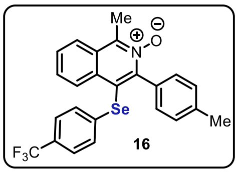

Following the **GP5** with substrate **S3**, a 7 mL reaction mixture (0.28 mmol) was collected after flow electrolysis. After the standard work-up, the volatile solvent was removed under reduced pressure, and the residue was purified by flash column chromatography to afford the product as white ash solid (83 mg, 63% yield).

Melting point: 162-165 °C.

^{1}H NMR (400 MHz, CDCl$_3$): δ 8.32 (d, *J* = 8.4 Hz, 1H), 8.03 (d, *J* = 8.4 Hz, 1H), 7.66 (t, *J* = 7.4 Hz, 1H), 7.56 (t, *J* = 7.6 Hz, 1H), 7.34 (d, *J* = 8.2 Hz, 2H), 7.22 (d, *J* = 7.9 Hz, 2H), 7.16 (d, *J* = 8.0 Hz, 2H), 7.10 (d, *J* = 8.1 Hz, 2H), 2.99 (s, 3H), 2.40 (s, 3H).

^{13}C NMR (101 MHz, CDCl$_3$): δ 152.9, 147.9, 138.9, 137.5, 133.1, 132.4, 130.7, 130.4, 129.5, 129.4, 129.3, 129.2, 129.1, 128.8, 128.6, 126.1 (q), 125.4, 124.7, 123.6, 122.7, 21.6, 14.0.

^{19}F NMR (376 MHz, CDCl$_3$): δ -62.61.

^{77}Se NMR (76 MHz, CDCl$_3$): δ 343.45.

HRMS (ESI): m/z calculated for [$C_{24}H_{19}NOFSe$] [M+H$^+$]: 474.0584, measured: 474.0590.

FTIR (Neat): 2992, 1609, 1398, 1323, 1294, 1188, 862, 760 cm^{-1}.

4-((3-Fluorophenyl)selanyl)-1-methyl-3-phenylisoquinoline 2-oxide (17):

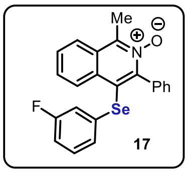

Following the **GP5** with substrate **S3**, a 7 mL reaction mixture (0.28 mmol) was collected after flow electrolysis. After the standard work-up, the volatile solvent was removed under reduced pressure, and the residue was purified by flash column chromatography to afford the product as white ash solid (70 mg, 61% yield).

Melting point: 160-162 °C.

^{1}H NMR (400 MHz, $CDCl_3$): δ 8.40 (d, *J* = 8.3 Hz, 1H), 8.02 (d, *J* = 8.5 Hz, 1H), 7.66 (ddd, *J* = 8.4, 7.0, 1.2 Hz, 1H), 7.58 (ddd, *J* = 8.2, 7.0, 1.1 Hz, 1H), 7.46 – 7.37 (m, 3H), 7.27 (dt, *J* = 3.4, 2.3 Hz, 2H), 7.07 (td, *J* = 8.1, 5.9 Hz, 1H), 6.86 – 6.76 (m, 2H), 6.74 – 6.66 (m, 1H), 2.98 (s, 3H).

^{13}C NMR (101 MHz, $CDCl_3$): δ 162.9 (d), 152.5, 147.6, 135.4, 133.9, 133.8, 130.6, 130.6, 130.5, 129.4, 129.4, 129.4, 129.3, 128.8, 128.6, 128.2, 125.6 (d), 124.6, 124.3, 117.0 (d), 113.9, 113.7, 13.9.

^{19}F NMR (376 MHz, $CDCl_3$): δ -111.40.

^{77}Se NMR (76 MHz, $CDCl_3$): δ 342.88.

HRMS (ESI): m/z calculated for [$C_{22}H_{17}NOFSSe$] [M+H$^+$]: 410.0459, measured: 410.0465.

1-Methyl-4-(naphthalen-2-ylselanyl)-3-phenylisoquinoline 2-oxide (18):

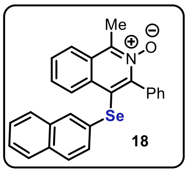

Following the **GP5** with substrate **S1**, a 7 mL reaction mixture (0.28 mmol) was collected after flow electrolysis. After the standard work-up, the volatile solvent was removed under reduced pressure, and the residue was purified by flash column chromatography to afford the product as light white sticky solid (75 mg, 61% yield).

^{1}H NMR (400 MHz, $CDCl_3$): δ 8.40 (d, *J* = 8.4 Hz, 1H), 8.01 (d, *J* = 8.5 Hz, 1H), 7.97 – 7.91 (m, 1H), 7.82 – 7.77 (m, 1H), 7.67 – 7.59 (m, 2H), 7.52 – 7.41 (m, 3H), 7.34 – 7.23 (m, 5H), 7.15 – 7.08 (m, 1H), 6.99 (dd, *J* = 7.3, 1.0 Hz, 1H), 2.97 (s, 3H).

^{13}C NMR (101 MHz, $CDCl_3$): δ 152.3, 147.2, 135.4, 134.0, 132.4, 131.4, 130.7, 129.5, 129.4, 129.3, 129.2, 129.2, 128.9, 128.7, 128.6, 128.2, 127.7, 126.6, 126.4, 126.1, 126.0, 125.0, 124.5, 13.9.

^{77}Se NMR (76 MHz, $CDCl_3$): δ 292.38.

HRMS (ESI): m/z calculated for [$C_{26}H_{20}NOSe$] [M+H$^+$]: 442.0710, measured: 442.0718.

FTIR (Neat): 2994, 2344, 1740, 1440, 1339, 1286, 1134, 957, 791, 739 cm^{-1}.

3-Phenyl-4-(phenylselanyl)isoquinoline 2-oxide (19):

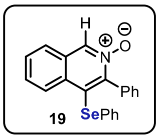

Following the **GP5** with substrate **S9**, a 7 mL reaction mixture (0.28 mmol) was collected after flow electrolysis. After the standard work-up, the volatile solvent was removed under reduced pressure, and the residue was purified by flash column chromatography to afford the product as white solid (82 mg, 78% yield).

^{1}H NMR (400 MHz, CDCl$_3$): δ 8.98 (s, 1H), 8.38 (d, *J* = 7.9 Hz, 1H), 7.75 (d, *J* = 7.5 Hz, 1H), 7.68 – 7.53 (m, 2H), 7.50 – 7.40 (m, 3H), 7.32 – 7.29 (m, 2H), 7.14 – 7.04 (m, 5H).

^{13}C NMR (101 MHz, CDCl$_3$): δ 152.5, 137.7, 134.4, 131.8, 131.4, 130.5, 129.8, 129.7, 129.5, 129.4, 129.1, 129.0, 128.9, 128.2, 128.0, 127.0, 125.3.

The analytical data are in accordance with reported literature.[31]

3-(4-Methoxyphenyl)-4-(phenylselanyl)isoquinoline 2-oxide(20):

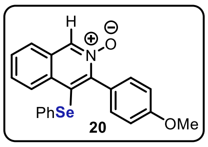

Following the **GP5** with substrate **S10**, a 7 mL reaction mixture (0.28 mmol) was collected after flow electrolysis. After the standard work-up, the volatile solvent was removed under reduced pressure, and the residue was purified by flash column chromatography to afford the product as white sticky solid (56 mg, 49% yield).

^{1}H NMR (400 MHz, CDCl$_3$): δ 8.94 (s, 1H), 8.34 (d, *J* = 8.3 Hz, 1H), 7.71 (d, *J* = 7.8 Hz, 1H), 7.56 (dt, *J* = 15.0, 6.9 Hz, 2H), 7.32 – 7.18 (m, 2H), 7.16 – 7.07 (m, 3H), 7.06 – 7.00 (m, 2H), 6.94 (d, *J* = 8.5 Hz, 2H), 3.84 (s, 3H).

^{13}C NMR (101 MHz, CDCl$_3$): δ 160.0, 152.4, 137.8, 131.9, 131.5, 131.3, 130.5, 129.8, 129.4, 129.0, 128.9, 128.2, 126.9, 126.6, 125.3, 113.6, 55.3.

^{77}Se NMR (76 MHz, CDCl$_3$): δ 341.38.

HRMS (ESI): m/z calculated for [$C_{22}H_{18}NO_2Se$] [M+H$^+$]: 408.0503, measured: 408.0500.

4-(Phenylselanyl)-3-(thiophen-2-yl)isoquinoline 2-oxide (21):

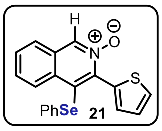

Following the **GP5** with substrate **S11**, a 7 mL reaction mixture (0.28 mmol) was collected after flow electrolysis. After the standard work-up, the volatile solvent was removed under reduced pressure, and the residue was purified by flash column chromatography to afford the product as block solid (73 mg, 68% yield).

Melting point: 221-223 °C.

^{1}H NMR (400 MHz, CDCl$_3$): δ 8.93 (s, 1H), 8.31 (d, *J* = 8.4 Hz, 1H), 7.70 (d, *J* = 8.0 Hz, 1H), 7.61 – 7.47 (m, 3H), 7.19 (dd, *J* = 3.6, 1.1 Hz, 1H), 7.14 – 7.09 (m, 5H).

^{13}C NMR (101 MHz, CDCl$_3$): δ 146.2, 137.6, 133.6, 131.9, 131.7, 131.3, 130.6, 130.1, 129.9, 129.8, 129.5, 129.5, 129.1, 129.0.

^{77}Se NMR (76 MHz, CDCl$_3$): δ 352.51.

HRMS (ESI): m/z calculated for [$C_{19}H_{14}NOSSe$] [M+H$^+$]: 383.9961, measured: 383.9964.

FTIR (Neat): 2987, 2315, 1750, 1573, 1423, 1305, 1211, 1121, 928, 791, 748 cm^{-1}.

4-(Benzylselanyl)-3-(thiophen-2-yl)isoquinoline 2-oxide (22):

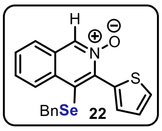

Following the **GP5** with substrate **S11**, a 7 mL reaction mixture (0.28 mmol) was collected after flow electrolysis. After the standard work-up, the volatile solvent was removed under reduced pressure, and the residue was purified by flash column chromatography to afford the product as white ash semi solid (63 mg, 57% yield).

^{1}H NMR (400 MHz, CDCl$_3$) δ 8.83 (s, 1H), 8.36 (d, *J* = 9.6 Hz, 0H), 7.69 – 7.64 (m, 1H), 7.61 – 7.53 (m, 2H), 7.14 – 7.05 (m, 6H), 6.85 – 6.80 (m, 1H), 3.85 (s, 2H).
^{13}C NMR (101 MHz, CDCl$_3$): δ 145.9, 137.1, 133.7, 131.5, 130.1, 129.6, 129.5, 129.2, 128.7, 128.4, 127.2, 126.2, 125.3, 33.8.

HRMS (ESI): m/z calculated for [$C_{20}H_{16}NOSSe$] [M+H$^+$]: 398.0118, measured: 398.0118.

FTIR (Neat): 3015, 2990, 2934, 1730, 1492,1358,1211, 1118,791cm^{-1}.

3-Phenyl-4-(p-tolylselanyl)isoquinoline 2-oxide (23):

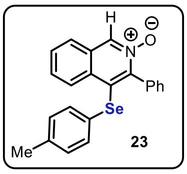

Following the **GP5** with substrate **S9**, a 7 mL reaction mixture (0.28 mmol) was collected after flow electrolysis. After the standard work-up, the volatile solvent was removed under reduced pressure, and the residue was purified by flash column chromatography to afford the product as colorless solid (85 mg, 78% yield).

^{1}H NMR (400 MHz, CDCl$_3$): δ 8.94 (s, 1H), 8.38 (dd, *J* = 7.9, 1.0 Hz, 1H), 7.72 (dd, *J* = 7.4, 1.9 Hz, 1H), 7.63 – 7.52 (m, 2H), 7.48 – 7.41 (m, 3H), 7.34 – 7.28 (m, 2H), 6.98 – 6.89 (m, 4H), 2.24 (s, 3H).

^{13}C NMR (101 MHz, CDCl$_3$): δ 152.3, 137.6, 137.1, 134.5, 131.4, 130.9, 130.2, 129.8, 129.7, 129.5, 129.1, 129.0, 128.3, 128.2, 127.9, 125.3, 21.1.

The analytical data are in accordance with reported literature.[31]

4-(Phenylselanyl)isoquinoline 2-oxide (24):

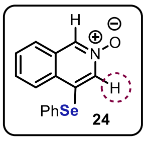

Following the **GP5** with substrate **S9**, a 7 mL reaction mixture (0.28 mmol) was collected after flow electrolysis. After the standard work-up, the volatile solvent was removed under reduced pressure, and the residue was purified by flash column chromatography to afford the product as colorless semi solid (45 mg, 54% yield).

^{1}H NMR (400 MHz, CDCl$_3$) δ 8.62 (s, 1H), 8.06 (dd, *J* = 5.9, 3.4 Hz, 1H), 7.91 (s, 1H), 7.69 (dt, *J* = 6.8, 3.4 Hz, 1H), 7.66 – 7.58 (m, 2H), 7.55 (dd, *J* = 7.8, 1.5 Hz, 2H), 7.40 – 7.27 (m, 3H).

^{13}C NMR (101 MHz, CDCl$_3$): δ 137.9, 135.4, 134.9, 130.9, 130.2, 129.9, 129.8, 129.4, 129.3, 128.0, 126.5, 125.9, 125.8.

^{77}Se NMR (76 MHz, CDCl$_3$): δ 353.56.

The analytical data are in accordance with reported literature.[31]

6,7-Dimethoxy-3-phenyl-4-(phenylselanyl)isoquinoline 2-oxide (25):

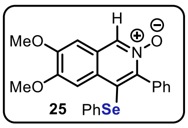

Following the **GP5** with substrate **S14**, a 7 mL reaction mixture (0.28 mmol) was collected after flow electrolysis. After the standard work-up, the volatile solvent was removed under reduced pressure, and the residue was purified by flash column chromatography to afford the product as colorless solid (70 mg, 57 % yield).

Melting point: 199-201 °C.

^{1}H NMR (400 MHz, CDCl$_3$): δ 8.86 (s, 1H), 7.54 (s, 1H), 7.45 – 7.40 (m, 3H), 7.35 – 7.29 (m, 2H), 7.15 – 7.05 (m, 5H), 7.00 (s, 1H), 4.01 (s, 3H), 3.76 (s, 3H).

^{13}C NMR (101 MHz, CDCl$_3$): δ 152.6, 152.0, 150.0, 136.7, 134.8, 131.8, 130.6, 129.9, 129.5, 129.0, 128.3, 128.2, 127.1, 126.1, 125.0, 108.0, 103.5, 56.4, 56.2.

^{77}Se NMR (76 MHz, CDCl$_3$): δ 345.18.

HRMS (ESI): m/z calculated for [$C_{23}H_{20}NOSe$] [M+H$^+$]: 438.0608, measured: 438.0611.

FTIR (Neat): 3000, 2370, 1575, 1468, 1333, 1254, 1153, 1005, 777 cm^{-1}.

3-Phenyl-4-(phenyltellanyl)isoquinoline 2-oxide (26):

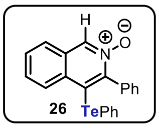

Following the **GP5** with substrate **S9**, a 7 mL reaction mixture (0.28 mmol) was collected after flow electrolysis. After the standard work-up, the volatile solvent was removed under reduced pressure, and the residue was purified by flash column chromatography to afford the product as white solid (57 mg, 48 % yield).

^{1}H NMR (400 MHz, CDCl$_3$): major product **26**; δ 8.88 (s, 1H), 8.26 (d, *J* = 8.3 Hz, 1H), 7.84 – 7.75 (m, 1H), 7.74 – 7.63 (m, 1H), 7.61 – 7.41 (m, 4H), 7.37 – 7.24 (m, 4H), 7.17 (ddd, *J* = 6.8, 3.9, 1.1 Hz, 1H), 7.12 – 7.02 (m, 2H). We detected a non-isolable N-oxide product without a tellurium group, along with the desired product **26**.

^{13}C NMR (101 MHz, CDCl$_3$): major product **26**; δ 154.0, 147.1, 137.6, 137.5, 137.1, 136.6, 133.5, 132.9, 132.7, 130.0, 129.7, 129.6, 129.5, 129.4,129.3, 129.2, 129.1, 129.1, 129.00, 128.9, 128.8, 128.4, 128.3, 127.9, 126.7, 125.5, 124.9, 124.5, 118.6, 116.1.

HRMS (ESI): m/z calculated for [$C_{15}H_{12}NO$] [M+H$^+$]: 222.0919, measured: 222.0921. HRMS data has been given for the non-isolable N-oxide product without a tellurium group.

The analytical data are in accordance with reported literature.[31]

1-Methyl-3-phenyl-4-(phenyltellanyl)isoquinoline 2-oxide (27):

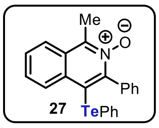

Following the **GP5** with substrate **S1**, a 7 mL reaction mixture (0.28 mmol) was collected after flow electrolysis. After the standard work-up, the volatile solvent was removed under reduced pressure, and the residue was purified by flash column chromatography to afford the product as white solid (102 mg, 83 % yield).

^{1}H NMR (400 MHz, CDCl$_3$): δ 8.41 (d, *J* = 8.1 Hz, 1H), 7.97 (d, *J* = 8.2 Hz, 1H), 7.62 (dd, *J* = 11.4, 4.1 Hz, 1H), 7.56 – 7.43 (m, 4H), 7.41 – 7.25 (m, 4H), 7.23 – 7.15 (m, 1H), 7.10 (t, *J* = 7.4 Hz, 2H), 2.99 (s, 3H). We detected a trace amount of non-isolable N-oxide product without a tellurium substituent along with the desired product **27**.

^{13}C NMR (101 MHz, CDCl$_3$): δ 154.0, 147.0, 138.8, 136.3, 134.5, 131.9, 129.9, 129.6, 129.6, 129.0, 128.8, 128.4, 128.1, 127.8, 124.6, 116.4, 115.6, 13.7.

HRMS (ESI): m/z calculated for [$C_{22}H_{18}NOTe$] [M+H$^+$]: 442.0451, measured: 442.0460.

1-Methyl-3-phenyl-4-(phenylselanyl)isoquinolin-2-ium-2-yl)(tosyl)amide (28):

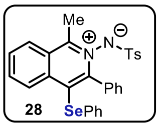

Following the **GP5** with substrate **S16**, a 7 mL reaction mixture (0.28 mmol) was collected after flow electrolysis. After the standard work-up, the volatile solvent was removed under reduced pressure, and the residue was purified by flash column chromatography to afford the product as colorless solid (93 mg, 61 % yield).

Melting point: 184-186 ^{o}C.

^{1}H NMR (400 MHz, CDCl$_3$): δ 8.68 – 8.46 (m, 1H), 8.33 (d, *J* = 8.1 Hz, 1H), 7.99 – 7.68 (m, 2H), 7.25 – 6.77 (m, 13H) aromatic protons are merged, 3.49 (s, 3H), 2.32 (s, 3H).

^{13}C NMR (101 MHz, CDCl$_3$): δ 164.2, 154.4, 142.2, 140.1, 136.5, 134.8, 134.7, 131.5, 130.6, 130.2, 130.1, 130.0, 129.9, 128.9, 128.5, 128.4, 128.1, 127.3, 127.2, 126.9, 126.1, 21.4, 19.8.

^{77}Se NMR (76 MHz, CDCl$_3$): δ 352.62.

HRMS (ESI): m/z calculated for [$C_{29}H_{25}N_2O_2SSe$] [M+H$^+$]: 545.0802, measured: 545.0807.

(3-(4-Methoxyphenyl)-1-methyl-4-(phenylselanyl)isoquinolin-2-ium-2-yl)(tosyl)amide (29):

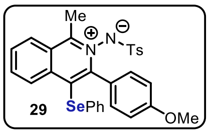

Following the **GP5** with substrate **S17**, a 7 mL reaction mixture (0.28 mmol) was collected after flow electrolysis. After the standard work-up, the volatile solvent was removed under reduced pressure, and the residue was purified by flash column chromatography to afford the product as white sticky solid (85 mg, 53 % yield).

^{1}H NMR (500 MHz, CDCl$_3$): δ 8.49 (d, *J* = 8.4 Hz, 1H), 8.32 (d, *J* = 8.5 Hz, 1H), 7.87 – 7.73 (m, 2H), 7.15 (d, *J* = 8.3 Hz, 2H), 7.11 – 7.03 (m, 2H), 6.94 (d, *J* = 8.1 Hz, 2H), 6.89 (d, *J* = 8.5 Hz, 2H), 3.78 (s, 3H), 3.50 (s, 3H), 2.32 (s, 3H).

^{13}C NMR (126 MHz, CDCl$_3$): δ 164.2, 159.6, 154.1, 142.5, 139.8, 136.5, 134.7, 131.9, 131.6, 130.6, 130.0, 129.9, 129.5, 128.7, 128.5, 128.0, 127.5, 127.2, 127.1, 126.0, 112.2, 55.1, 21.4, 19.9.

^{77}Se NMR (76 MHz, CDCl$_3$): δ 355.01.

HRMS (ES$^+$): m/z calculated for [$C_{30}H_{27}N_2O_3SSe$] [M+H]$^+$: 575.0910, measured: 575.0918.

FTIR (Neat): 2890, 2349, 1607, 1510, 1337, 1248, 1176, 829, 763 cm^{-1}.

(3-Phenyl-4-(phenylselanyl)isoquinolin-2-ium-2-yl)(tosyl)amide (30):

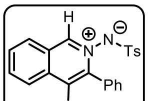

30

Following the **GP5** with substrate **S18**, a 7 mL reaction mixture (0.28 mmol) was collected after flow electrolysis. After the standard work-up, the volatile solvent was removed under reduced pressure, and the residue was purified by flash column chromatography to afford the product as white solid (96 mg, 65 % yield).

^{1}H NMR (400 MHz, CDCl$_3$): δ 9.83 (s, 1H), 8.47 (d, *J* = 8.6 Hz, 1H), 8.16 (d, *J* = 8.1 Hz, 1H), 7.92 – 7.85 (m, 1H), 7.84 – 7.75 (m, 1H), 7.34 – 7.22 (m, 1H), 7.21 – 7.03 (m, 7H), 7.01 – 6.87 (m, 4H), 6.78 – 6.60 (m, 2H), 2.33 (s, 3H).

^{13}C NMR (101 MHz, CDCl$_3$): δ 152.7, 150.5, 140.9, 140.4, 137.3, 135.3, 133.5, 131.2, 130.9, 130.4, 130.0, 129.8, 129.6, 129.5, 129.2, 129.1, 128.7, 127.4, 127.4, 126.7, 21.5.

^{77}Se NMR (76 MHz, CDCl$_3$): δ 353.10.

The analytical data are in accordance with reported literature.[29]

(3-(4-Methoxyphenyl)-4-(phenylselanyl)isoquinolin-2-ium-2-yl)(tosyl)amide (31):

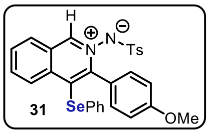

31

Following the **GP5** with substrate **S19**, a 7 mL reaction mixture (0.28 mmol) was collected after flow electrolysis. After the standard work-up, the volatile solvent was removed under reduced pressure, and the residue was purified by flash column chromatography to afford the product as colorless solid (83 mg, 53% yield).

^{1}H NMR (400 MHz, CDCl$_3$): δ 9.77 (s, 1H), 8.45 (d, *J* = 8.1 Hz, 1H), 8.13 (d, *J* = 7.8 Hz, 1H), 7.94 – 7.64 (m, 2H), 7.23 – 7.00 (m, 5H), 7.00 – 6.86 (m, 4H), 6.71 (d, *J* = 7.8 Hz, 2H), 6.60 (d, *J* = 8.0 Hz, 2H), 3.82 (s, 3H), 2.32 (s, 3H).

^{13}C NMR (101 MHz, CDCl$_3$): δ 159.8, 152.6, 150.5, 140.8, 140.6, 137.4, 135.2, 131.8, 131.3, 130.9, 130.2, 130.0, 129.5, 129.2, 129.0, 127.4, 127.3, 126.6, 125.8, 112.7, 55.3, 21.5.

The analytical data are in accordance with reported literature.[29]

(3-Phenyl-4-(p-tolylselanyl)isoquinolin-2-ium-2-yl)(tosyl)amide (32):

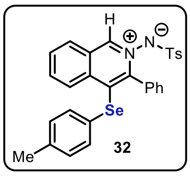

32

Following the **GP5** with substrate **S18**, a 7 mL reaction mixture (0.28 mmol) was collected after flow electrolysis. After the standard work-up, the volatile solvent was removed under reduced pressure, and the residue was purified by flash column chromatography to afford the product as colorless solid (87 mg, 57% yield).

^{1}H NMR (400 MHz, CDCl$_3$): δ 9.60 (s, 1H), 8.30 (d, *J* = 8.5 Hz, 1H), 7.96 (d, *J* = 8.1 Hz, 1H), 7.70 (t, *J* = 7.8 Hz, 1H), 7.60 (t, *J* = 7.5 Hz, 1H), 7.09 (dd, *J* = 13.5, 5.9 Hz, 1H), 7.01 – 6.87 (m, 4H), 6.66 (ddd, *J* = 42.3, 32.6, 7.8 Hz, 8H), 2.15 (s, 3H), 2.02 (s, 3H).

^{13}C NMR (101 MHz, CDCl$_3$): δ 152.5, 150.3, 140.9, 140.4, 137.6, 137.3, 135.3, 133.6, 131.2, 130.3, 130.3, 130.1, 129.6, 129.1, 128.7, 127.4, 126.7, 21.5, 21.1.

^{77}Se NMR (76 MHz, CDCl$_3$): δ 346.96.

The analytical data are in accordance with reported literature.[32]

(3-Phenyl-4-(phenyltellanyl)isoquinolin-2-ium-2-yl)(tosyl)amide (33):

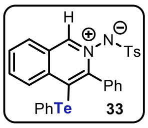

Following the **GP5** with substrate **S18**, a 7 mL reaction mixture (0.28 mmol) was collected after flow electrolysis. After the standard work-up, the volatile solvent was removed under reduced pressure, and the residue was purified by flash column chromatography to afford the product as colorless solid (84 mg, 52% yield).

^{1}H NMR (400 MHz, $CDCl_3$): δ 9.74 (s, 1H), 8.35 (d, *J* = 8.2 Hz, 1H), 8.15 – 8.08 (m, 1H), 7.86 – 7.75 (m, 2H), 7.40 – 7.27 (m, 4H), 7.26 – 7.14 (m, 5H), 7.09 (dd, *J* = 10.5, 4.5 Hz, 2H), 6.98 (d, *J* = 8.0 Hz, 2H), 6.78 – 6.73 (m, 2H), 2.37 (s, 3H).

^{13}C NMR (101 MHz, $CDCl_3$): δ 153.8, 150.1, 140.8, 140.6, 138.6, 137.1, 136.8, 135.0, 133.6, 130.2, 130.0, 129.9, 129.7, 129.1, 129.1, 128.5, 127.8, 127.2, 126.7, 120.9, 116.2, 104.6, 21.5.

The analytical data are in accordance with reported literature.[32]

5-methyl-7-phenyl-8-(phenylselanyl)-1,6-naphthyridine 6-oxide (34)

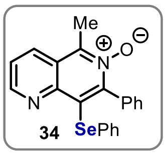

^{1}H NMR (400 MHz, CDCl3): δ 8.99 (d, *J* = 3.1 Hz, 1H), 8.29 (dd, *J* = 8.5, 1.3 Hz, 1H), 7.56 (dd, *J* = 8.5, 4.2 Hz, 1H), 7.38 – 7.29 (m, 3H), 7.21 (dd, *J* = 12.6, 6.9 Hz, 2H), 7.14 – 6.96 (m, 5H), 2.88 (s, 3H).

^{13}C NMR (101 MHz, CDCl3): δ 154.21, 151.96, 146.42, 144.45, 134.32, 132.63, 132.40, 131.51, 129.63, 129.55, 128.93, 128.79, 128.09, 127.05, 123.68, 13.64.

^{77}Se NMR (76 MHz, CDCl3): δ 355.38

HRMS (ES+): m/z calculated for [C21H17N2OSe] $[M+H]^+$: 393.0506, measured: 393.0512.

2.8. Characterization data for isoindole *N*-oxide derivatives

(*Z*)-3-Methyl-1-(phenyl(phenylselanyl)methylene)-1*H*-isoindole 2-oxide (4):

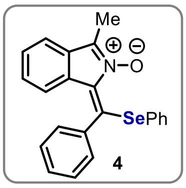

Following the **GP6** with substrate **S1**, a 7 mL reaction mixture (0.28 mmol) was collected after flow electrolysis. After the standard work-up, the volatile solvent was removed under reduced pressure, and the residue was purified by flash column chromatography to afford the product as brown solid (95 mg, 87% yield).

Melting point: 173-175 °C.

^{1}H NMR (400 MHz, $CDCl_3$): δ 7.30 (t, *J* = 8.0 Hz, 3H), 7.22 (t, *J* = 7.3 Hz, 1H), 7.18 – 7.12 (m, 3H), 7.10 (d, *J* = 7.4 Hz, 1H), 6.96 (ddd, *J* = 16.0, 15.3, 7.5 Hz, 5H), 5.91 (d, *J* = 7.9 Hz, 1H), 2.51 (s, 3H).

^{13}C NMR (101 MHz, $CDCl_3$): δ 149.2, 142.0, 137.6, 135.3, 134.2, 132.5, 129.4, 128.7, 128.5, 128.3, 128.3, 126.1, 127.5, 121.3, 118.3, 9.3.

HRMS (ES^+): m/z calculated for $[C_{22}H_{18}NOSe]$ $[M+H]^+$: 392.0554, measured: 392.0561.

FTIR (Neat): 2941, 2883, 1608, 1740, 1587, 1437, 1358, 1217, 1020, 833, 748 cm^{-1}.

(*Z*)-3-Methyl-1-((phenylselanyl)(p-tolyl)methylene)-1*H*-isoindole 2-oxide (36):

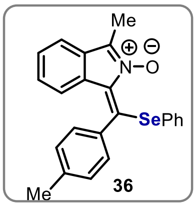

Following the **GP6** with substrate **S3**, a 7 mL reaction mixture (0.28 mmol) was collected after flow electrolysis. After the standard work-up, the volatile solvent was removed under reduced pressure, and the residue was purified by flash column chromatography to afford the product as brown solid (75 mg, 66% yield).

Melting point: 182-185 ^{o}C.

^{1}H NMR (400 MHz, CDCl$_3$): δ 7.26 – 7.18 (m, 3H), 7.14 (td, *J* = 7.6, 0.8 Hz, 1H), 7.03 (ddd, *J* = 6.9, 2.3, 1.1 Hz, 1H), 6.97 – 6.83 (m, 5H), 6.75 (d, *J* = 8.1 Hz, 2H), 5.92 (d, *J* = 7.9 Hz, 1H), 2.42 (s, 3H), 2.18 (s, 3H).

^{13}C NMR (101 MHz, CDCl$_3$): δ 150.0, 141.7, 138.2, 137.6, 134.2, 132.4, 132.3, 129.6, 128.9, 128.5, 128.3, 128.2, 127.4, 127.1, 126.1, 121.3, 118.2, 21.4, 9.3.

HRMS (ES$^+$): m/z calculated for [$C_{23}H_{20}NOSe$] [M+H]$^+$: 406.0710, measured: 406.0717.

FTIR (Neat): 3057, 2922, 1450, 1360, 1217, 1018, 762, 751 cm^{-1}.

(Z)-3-Methyl-1-(phenyl(p-tolylselanyl)methylene)-1*H*-isoindole 2-oxide (37):

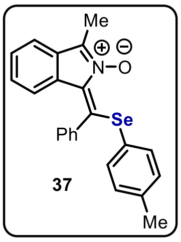

Following the **GP6** with substrate **S1**, a 7 mL reaction mixture (0.28 mmol) was collected after flow electrolysis. After the standard work-up, the volatile solvent was removed under reduced pressure, and the residue was purified by flash column chromatography to afford the product as brown solid (76 mg, 67% yield).

Melting point: 190-192 ^{o}C.

^{1}H NMR (400 MHz, CDCl$_3$): δ 7.31 (d, *J* = 7.6 Hz, 1H), 7.22 (td, *J* = 7.6, 0.9 Hz, 1H), 7.18 – 7.13 (m, 5H), 6.98 – 6.88 (m, 3H), 6.80 (d, *J* = 7.7 Hz, 2H), 5.89 (d, *J* = 7.9 Hz, 1H), 2.50 (s, 3H), 2.19 (s, 3H).

^{13}C NMR (101 MHz, CDCl$_3$): δ 149.8, 141.9, 138.5, 137.5, 135.5, 134.2, 132.5, 129.1, 128.7, 128.3, 128.2, 127.5, 127.1, 126.1, 125.8, 121.3, 118.3, 21.3, 9.4.

^{77}Se NMR (76 MHz, CDCl$_3$): δ 400.01.

HRMS (ES$^+$): m/z calculated for [$C_{23}H_{20}NOSe$] [M+H]$^+$: 406.0710, measured: 406.0719.

(Z)-1-((Benzylselanyl)(phenyl)methylene)-3-methyl-1*H*-isoindole 2-oxide (38):

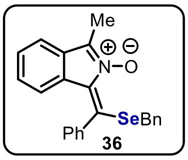

Following the **GP6** with substrate **S1**, a 7 mL reaction mixture (0.28 mmol) was collected after flow electrolysis. After the standard work-up, the volatile solvent was removed under reduced pressure, and the residue was purified by flash column chromatography to afford the product as brown solid (75 mg, 66% yield).

^{1}H NMR (400 MHz, CDCl$_3$): δ 7.62 – 7.49 (m, 3H), 7.34 – 7.27 (m, 3H), 7.25 – 7.15 (m, 4H), 7.14 – 7.08 (m, 2H), 6.98 – 6.91 (m, 1H), 5.89 (d, *J* = 7.9 Hz, 1H), 3.38 (s, 2H), 2.44 (s, 3H).

^{13}C NMR (101 MHz, CDCl$_3$): δ 149.0, 141.4, 136.8, 136.3, 135.0, 132.3, 129.6, 129.4, 129.2, 128.6, 128.0, 127.4, 127.1, 125.8, 31.4, 9.3.

^{77}Se NMR (76 MHz, CDCl$_3$): δ 510.49.

HRMS (ES$^+$): m/z calculated for [$C_{23}H_{20}NOSe$] [M+H]$^+$: 406.0710, measured: 406.0716.

FTIR (Neat): 2988, 2890, 1785, 1583, 1368, 1221, 1028, 752 cm^{-1}.

(*Z*)-1-((3-Chlorophenyl)(phenylselanyl)methylene)-3-methyl-1*H*-isoindole 2-oxide (39):

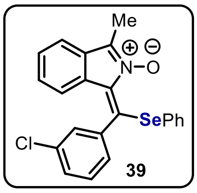

Following the **GP6** with substrate **S4**, a 7 mL reaction mixture (0.28 mmol) was collected after flow electrolysis. After the standard work-up, the volatile solvent was removed under reduced pressure, and the residue was purified by flash column chromatography to afford the product as brown sticky solid (80 mg, 67% yield).

^{1}H NMR (400 MHz, CDCl$_3$): δ 7.35 – 7.29 (m, 3H), 7.29 – 7.23 (m, 1H), 7.15 (ddd, *J* = 5.9, 5.5, 4.3 Hz, 1H), 7.08 (ddd, *J* = 14.7, 5.5, 1.6 Hz, 4H), 6.99 (td, *J* = 7.8, 1.0 Hz, 1H), 6.92 (d, *J* = 1.9 Hz, 1H), 6.87 (dt, *J* = 6.5, 1.8 Hz, 1H), 6.03 (d, *J* = 7.9 Hz, 1H), 2.51 (s, 3H).

^{13}C NMR (101 MHz, CDCl$_3$): δ 146.7, 142.4, 137.7, 136.9, 134.3, 132.7, 129.7, 129.1, 129.0, 128.9, 128.4,127.9, 127.4, 127.0, 125.8, 121.3, 118.5, 9.4.

HRMS (ES$^+$): m/z calculated for [$C_{22}H_{17}ClNOSe$] [M+H]$^+$: 426.0164, measured: 426.0171.

(*Z*)-1-((4-Methoxyphenyl)(phenylselanyl)methylene)-3-methyl-1*H*-isoindole 2-oxide (40):

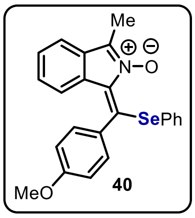

Following the **GP6** with substrate **S2**, a 7 mL reaction mixture (0.28 mmol) was collected after flow electrolysis. After the standard work-up, the volatile solvent was removed under reduced pressure, and the residue was purified by flash column chromatography to afford the product as brown solid (55 mg, 47% yield).

Melting point: 174-176 °C.

^{1}H NMR (400 MHz, CDCl$_3$): δ 7.38 – 7.18 (m, 4H), 7.12 (d, *J* = 7.4 Hz, 1H), 6.99 (ddd, *J* = 15.3, 11.6, 4.2 Hz, 3H), 6.91 – 6.81 (m, 2H), 6.76 – 6.62 (m, 2H), 6.05 (d, *J* = 7.9 Hz, 1H), 3.74 (s, 3H), 2.50 (s, 3H).

^{13}C NMR (101 MHz, CDCl$_3$): δ 159.6, 149.5, 141.8, 137.6, 134.5, 132.5, 130.1, 129.7, 128.5, 128.3, 127.7, 127.5, 127.2, 126.2, 121.3, 118.3, 113.8, 55.4, 9.4.

^{77}Se NMR (76 MHz, CDCl$_3$): δ 605.84.

HRMS (ES$^+$): m/z calculated for [$C_{23}H_{20}NO_2Se$] [M+H]$^+$: 422.0659, measured: 422.0667.

(Z)-1-((Benzylselanyl)(4-methoxyphenyl)methylene)-3-methyl-1*H*-isoindole 2-oxide (41):

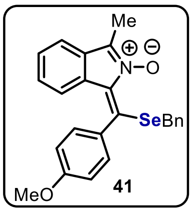

Following the **GP6** with substrate **S2**, a 7 mL reaction mixture (0.28 mmol) was collected after flow electrolysis. After the standard work-up, the volatile solvent was removed under reduced pressure, and the residue was purified by flash column chromatography to afford the product as brown sticky solid (42 mg, 35% yield).

^{1}H NMR (400 MHz, CDCl$_3$): δ 7.26 – 6.98 (m, 12H) all aromatic proton peaks merged, 6.92 (t, *J* = 7.6 Hz, 1H), 5.96 (d, *J* = 7.9 Hz, 1H), 3.86 (s, 2H), 3.34 (s, 3H), 2.36 (s, 3H). There are non-isolable impurities in the product.

^{13}C NMR (101 MHz, CDCl$_3$): δ 160.1, 149.4, 141.4, 137.00, 135.3, 132.3, 129.4, 129.3, 128.6, 127.4, 127.1, 126.9, 126.0, 121.0, 118.2, 114.9, 104.6, 55.6, 31.5, 9.3.

^{77}Se NMR (76 MHz, CDCl$_3$) δ 399.94.

HRMS (ES$^+$): m/z calculated for [$C_{24}H_{22}NO_2Se$] [M+H]$^+$: 436.0739, measured: 436.0741.

3-Methyl-1-(((4-phenoxyphenyl)selanyl)(phenyl)methylene)-1*H*-isoindole 2-oxide (42):

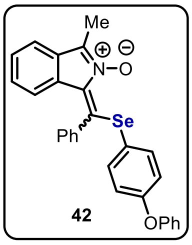

Following the **GP6** with substrate **S1**, a 7 mL reaction mixture (0.28 mmol) was collected after flow electrolysis. After the standard work-up, the volatile solvent was removed under reduced pressure, and the residue was purified by flash column chromatography to afford the product as white solid [85 mg, 63% combined yield (*Z/E* ratio 45:05)].

The compound **42** exists as a mixture of stereoisomers, which were separable. One stereoisomer was characterized by **^{1}H**, **^{13}C**, and **^{19}F** NMR spectroscopy, while the second was characterized by **^{1}H** NMR only. Further structural studies are required to determine the exact stereochemistry of each isomer.

Melting point: 177-179 °C.

^{1}H NMR (500 MHz, CDCl$_3$): δ 7.38 – 7.31 (m, 3H), 7.30 – 7.22 (m, 6H), 7.14 – 7.08 (m, 1H), 7.03 – 6.92 (m, 3H), 6.88 – 6.82 (m, 2H), 6.69 – 6.62 (m, 2H), 5.95 (dt, *J* = 7.9, 0.8 Hz, 1H), 2.52 (s, 3H).

^{13}C NMR (126 MHz, CDCl$_3$): δ 157.6, 157.2, 149.3, 142.2, 139.4, 135.5, 134.3, 132.5, 129.8, 128.9, 128.5, 128.3, 127.6, 127.3, 126.1, 123.5, 123.4, 121.3, 119.2, 118.7, 118.4, 9.3.

HRMS (ES$^+$): m/z calculated for [$C_{28}H_{22}NO_2Se$] [M+H]$^+$: 484.0816, measured: 484.0821.

FTIR (Neat): 2920, 2853, 1608, 1527, 1360, 1329, 1233, 1013, 775, 758 cm^{-1}.

^{1}H NMR (500 MHz, CDCl$_3$): (minor stereoisomer): δ 7.27 – 7.23 (m, 3H), 7.20 – 7.15 (m, 6H), 7.03 (d, *J* = 0.6 Hz, 1H), 6.98 – 6.82 (m, 3H), 6.76 (dd, *J* = 7.8, 0.8 Hz, 2H), 6.57 (d, *J* = 8.1 Hz, 2H), 5.86 (d, *J* = 7.9 Hz, 1H), 2.45 (s, 3H).

(*Z*)-3-Methyl-1-((phenylselanyl)(4-(trifluoromethyl)phenyl)methylene)-1*H*-isoindole 2-oxide (43):

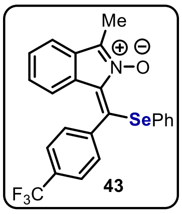

Following the **GP6** with substrate **S6**, a 7 mL reaction mixture (0.28 mmol) was collected after flow electrolysis. After the standard work-up, the volatile solvent was removed under reduced pressure, and the residue was purified by flash column chromatography to afford the product as brown solid (60 mg, 47% yield).

Melting point: 122-125 °C.

^{1}H NMR (400 MHz, CDCl$_3$): δ 7.40 (d, *J* = 8.0 Hz, 2H), 7.34 (d, *J* = 7.6 Hz, 1H), 7.29 – 7.23 (m, 3H), 7.17 – 7.06 (m, 3H), 7.02 – 6.94 (m, 3H), 5.94 (d, *J* = 7.9 Hz, 1H), 2.51 (s, 3H).

^{13}C NMR (101 MHz, CDCl$_3$): δ 146.6, 142.5, 139.0, 137.7, 134.2, 132.8, 130.5 (q) 129.3, 129.0, 128.9, 128.5, 128.0, 127.5, 125.8, 125.7, 125.3 (q), 121.1, 118.6, 9.4.

^{19}F NMR (376 MHz, CDCl$_3$): δ -62.81.

HRMS (ES$^+$): m/z calculated for [$C_{23}H_{17}NOFSe$] [M+H]$^+$: 460.0427, measured: 460.0430.

(*Z*)-1-((4-Fluorophenyl)(phenylselanyl)methylene)-3-methyl-1*H*-isoindole 2-oxide (44):

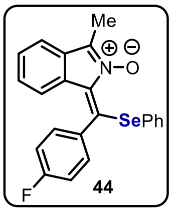

Following the **GP6** with substrate **S5**, a 7 mL reaction mixture (0.28 mmol) was collected after flow electrolysis. After the standard work-up, the volatile solvent was removed under reduced pressure, and the residue was purified by flash column chromatography to afford the product brown solid (51 mg, 45% yield).

Melting point: 135-137 °C.

^{1}H NMR (400 MHz, CDCl$_3$): δ 7.27 – 7.14 (m, 4H), 7.07 (t, *J* = 7.4 Hz, 1H), 6.95 (t, *J* = 7.6 Hz, 2H), 6.87 (ddd, *J* = 8.9, 8.5, 4.9 Hz, 3H), 6.78 (t, *J* = 8.5 Hz, 2H), 5.89 (d, *J* = 7.9 Hz, 1H), 2.42 (s, 3H).

^{13}C NMR (101 MHz, CDCl$_3$): δ 162.4 (d), 147.8, 142.1, 137.6, 134.5, 132.6, 131.3 (d), 130.7 (d), 129.3, 128.7, 128.4, 127.7, 127.2, 125.9, 121.1, 118.4, 115.5 (d), 9.3.

^{19}F NMR (376 MHz, CDCl$_3$): δ -112.28

^{77}Se NMR (95 MHz, CDCl$_3$): δ 605.46, 400.00.

HRMS (ES$^+$): m/z calculated for [$C_{24}H_{22}NO_3Se$] [M+H]$^+$: 410.0459, measured: 410.0468.

1-(((3-Fluorophenyl)selanyl)(phenyl)methylene)-3-methyl-1*H*-isoindole 2-oxide (45):

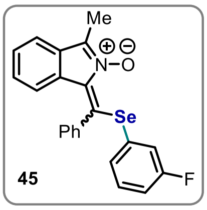

Following the **GP6** with substrate **S1**, a 7 mL reaction mixture (0.28 mmol) was collected after flow electrolysis. After the standard work-up, the volatile solvent was removed under reduced pressure, and the residue was purified by flash column chromatography to afford the product as light brown sticky solid [72 mg, 66% combined yield (*Z/E* ratio 44:06)].

The compound **45** exists as a mixture of stereoisomers, which were separable. One stereoisomer was characterized by ^{1}**H**, ^{13}**C**, and ^{19}**F** NMR spectroscopy, while the second was characterized by ^{1}**H** NMR only. Further structural studies are required to determine the exact stereochemistry of each isomer. ^{1}**H NMR (400 MHz, CDCl$_3$):** δ 7.33 (d, *J* = 7.6 Hz, 1H), 7.23 (ddd, *J* = 10.0, 6.6, 4.9 Hz, 4H), 7.11 (d, *J* = 7.6 Hz, 1H), 6.98 (qd, *J* = 8.4, 2.8 Hz, 5H), 6.87 – 6.70 (m, 1H), 5.96 (d, *J* = 7.9 Hz, 1H), 2.52 (s, 3H).

^{13}**C NMR (101 MHz, CDCl$_3$):** δ 161.5 (d), 147.8, 142.1, 134.9, 134.3, 133.3 (d), 132.6, 131.2, 131.1, 129.5, 129.4, 128.6, 128.6, 128.4, 127.7, 127.3, 125.9, 124.3 (d), 121.3, 118.4, 115.6 (d), 9.3.

^{19}**F NMR (376 MHz, CDCl$_3$):** δ -112.50.

HRMS (ES$^+$): m/z calculated for [$C_{22}H_{17}NOFSe$] [M+H]$^+$: 410.0459, measured: 410.0469.

FTIR (Neat): 2922, 2853, 1529, 1350, 1330, 1261, 1051, 779, 748 cm^{-1}.

^{1}**H NMR (400 MHz, CDCl$_3$)** (minor stereoisomer): δ 7.26 (d, *J* = 7.6 Hz, 1H), 7.21 – 7.10 (m, 4H), 7.03 (d, *J* = 7.6 Hz, 1H), 6.95 – 6.83 (m, 5H), 6.74 (ddd, *J* = 8.5, 2.5, 1.2 Hz, 1H), 5.88 (d, *J* = 7.9 Hz, 1H), 2.44 (s, 3H).

1-((4-Fluorophenyl)((3-fluorophenyl)selanyl)methylene)-3-methyl-1*H*-isoindole 2-oxide (46):

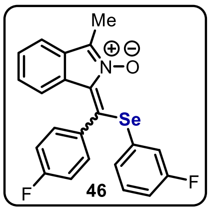

Following the **GP6** with substrate **S5**, a 7 mL reaction mixture (0.28 mmol) was collected after flow electrolysis. After the standard work-up, the volatile solvent was removed under reduced pressure, and the residue was purified by flash column chromatography to afford the product as brown sticky solid [(58 mg, 49% combined yield (*Z/E* ratio 45:05)].

The compound **46** exists as a mixture of stereoisomers, which were separable. One stereoisomer was characterized by ^{1}**H**, ^{13}**C**, and ^{19}**F** NMR spectroscopy, while the second was characterized by ^{1}**H** NMR only. Further structural studies are required to determine the exact stereochemistry of each isomer. ^{1}**H NMR (400 MHz, CDCl$_3$):** δ 7.34 (d, *J* = 7.5 Hz, 1H), 7.30 – 7.24 (m, 1H) residual $CHCl_3$ peak merged, 7.16 – 6.81 (m, 9H) aromatic C-H peaks merged, 5.99 (d, *J* = 7.9 Hz, 1H), 2.51 (s, 3H).

^{13}**C NMR (101 MHz, CDCl$_3$):** δ 163.4 (d), 160.9 (d), 146.9, 142.8, 134.7, 133.4 (d), 132.7, 131.1, 131.0, 130.7 (d), 129.8 (d), 128.0, 127.6, 126.0, 124.4 (d), 121.3, 118.7, 116.1, 115.9, 115.6, 9.4.

^{19}**F NMR (376 MHz, CDCl$_3$):** δ -111.71, -112.47.

^{77}**Se NMR (76 MHz, CDCl$_3$):** δ 608.46, 399.98.

HRMS (ES+): m/z calculated for [$C_{22}H_{16}NOF_2Se$] [M+H]+: 426.0209, measured: 426.0202.

^{1}H NMR (400 MHz, CDCl$_3$) (minor stereoisomer): δ 7.32 (dt, *J* = 15.0, 7.4 Hz, 2H), 7.18 – 7.00 (m, 6H), 7.00 – 6.91 (m, 2H), 6.89 – 6.80 (m, 1H), 6.08 (d, *J* = 7.8 Hz, 1H), 2.50 (s, 3H).

^{19}F NMR (376 MHz, CDCl$_3$): δ -111.05, -112.20.

^{77}Se NMR (76 MHz, CDCl$_3$): δ 733.84, 384.99.

(*Z*)-5,6-dimethoxy-3-methyl-1-(phenyl(phenylselanyl)methylene)-1*H*-isoindole 2-oxide (47):

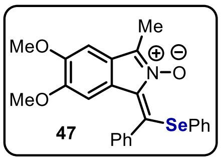

Following the **GP6** with substrate **S15**, a 7 mL reaction mixture (0.28 mmol) was collected after flow electrolysis. After the standard work-up, the volatile solvent was removed under reduced pressure, and the residue was purified by flash column chromatography to afford the product as brown solid (75 mg, 60% yield).

Melting point: 185-188 °C.

^{1}H NMR (400 MHz, CDCl$_3$): δ 7.32 (d, *J* = 7.3 Hz, 2H), 7.22 – 7.05 (m, 4H), 7.03 – 6.95 (m, 4H), 6.76 (s, 1H), 5.37 (s, 1H), 3.88 (s, 3H), 3.27 (s, 3H), 2.47 (s, 3H).

^{13}C NMR (101 MHz, CDCl$_3$): δ 149.3, 148.9, 146.9, 142.1, 137.7, 135.5, 134.2, 129.3, 128.9, 128.5, 128.4, 128.2, 128.1, 125.8, 119.4, 105.0, 101.1, 56.2, 55.4, 9.5.

^{77}Se NMR (76 MHz, CDCl$_3$): δ 592.06.

HRMS (ES+): m/z calculated for [$C_{24}H_{22}NO_3Se$] [M+H]+: 452.0765, measured: 452.0775.

FTIR (Neat): 2922, 2852, 1735, 1603, 1433, 1289, 1043, 856, 773, 736 cm^{-1}.

(*Z*)-5-methyl-7-(phenyl(phenylselanyl)methylene)-7H-pyrrolo[3,4-b]pyridine 6-oxide (48)

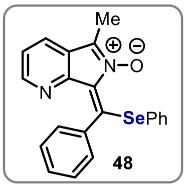

Following the **GP6** with substrate **S25**, a 7 mL reaction mixture (0.28 mmol) was collected after flow electrolysis. After the standard work-up, the volatile solvent was removed under reduced pressure, and the residue was purified by flash column chromatography to afford the product as brown solid (56 mg, 51% yield).

^{1}H NMR (400 MHz, CDCl3): δ 8.31 (dd, *J* = 4.9, 1.4 Hz, 1H), 7.73 (dd, *J* = 7.8, 1.5 Hz, 1H), 7.47 – 7.35 (m, 3H), 7.26 (s, 4H), 7.21 – 7.08 (m, 4H), 2.68 (s, 3H).

^{13}C NMR (101 MHz, CDCl3): δ 155.94, 148.11, 145.13, 139.22, 137.33, 134.50, 132.85, 129.45, 129.03, 128.51, 128.35, 128.07, 127.39, 126.85, 124.69, 121.59, 9.27.

^{77}Se NMR (76 MHz, CDCl3): δ 400.

HRMS (ES+): m/z calculated for [C21H17N2OSe] [M+H]+: 393.0506, measured: 393.0510.

2.9. Experimental procedure and characterization data for synthetic applications

Synthesis of *N*,3-diphenyl-4-(phenylselanyl)isoquinolin-1-amine (50):

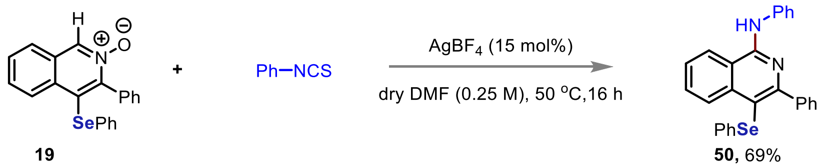

An oven-dried 7 mL reaction vial was charged with isoquinoline N-oxide **19** (38 mg, 0.1 mmol), Phenyl isothiocyanate (16 mg, 0.12 mmol) and $AgBF_4$ (4.4 mg, 0.0225 mmol). Dry DMF (0.275 mL, 0.25 M) was then added, and the mixture was stirred at 50 °C for 16 hours. After completion of the reaction, the mixture was cooled to room temperature and concentrated under reduced pressure. The crude product was purified by silica gel flash chromatography (PE:EA = 9:1) to afford 31 mg (69% yield) of the desired product **50** as a light brown solid.

Melting point: 164-166 °C.

^{1}H NMR (400 MHz, $CDCl_3$): δ 8.50 (dd, *J* = 8.4, 0.7 Hz, 1H), 7.98 (t, *J* = 7.9 Hz, 1H), 7.85 (t, *J* = 8.1 Hz, 2H), 7.73 – 7.67 (m, 3H), 7.59 (ddd, *J* = 11.1, 6.1, 2.7 Hz, 1H), 7.46 – 7.34 (m, 5H), 7.18 – 7.06 (m, 6H).

^{13}C NMR (101 MHz, $CDCl_3$): δ 152.1, 140.1, 140.0, 134.5, 131.2, 130.2, 129.9, 129.2, 129.1, 128.8, 128.1, 127.5, 126.9, 125.7,123.0, 121.6, 120.1, 118.6,110.7.

^{77}Se NMR (76 MHz, $CDCl_3$): δ 291.73.

HRMS (ES$^+$): m/z calculated for $[C_{27}H_{21}N_2Se]$ $[M+H]^+$: 453.0870, measured: 453.0877.

FTIR (Neat): 3443, 3042, 2987, 1750, 1616, 1595, 1415, 1018, 890, 768 cm^{-1}.

Synthesis of (*E*)-3-phenyl-4-(phenylselanyl)-1-styrylisoquinoline (51):

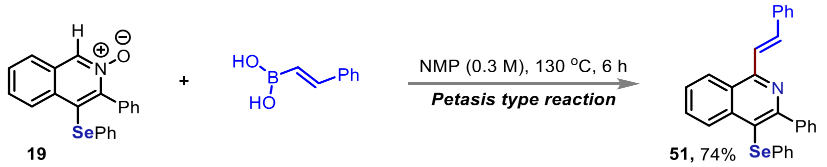

An oven-dried 7 mL reaction vial was charged with isoquinoline N-oxide **19** (38 mg, 0.1 mmol) and trans-2-phenylvinylboronic acid (22 mg, 0.15 mmol). Dry NMP (0.33 mL, 0.3 M) was then added, and the mixture was stirred at 130 °C for 6 hours. After completion of the reaction, the mixture was cooled to room temperature and concentrated under reduced pressure. The crude product was purified by silica gel flash chromatography (PE:EA = 8.5:1.5) to afford 34 mg (74% yield) of the desired product **51** as a yellow solid.

Melting point: 178-180 °C.

^{1}**H NMR (400 MHz, CDCl$_3$):** δ 8.55 (d, *J* = 8.4 Hz, 1H), 8.44 (d, *J* = 8.3 Hz, 1H), 8.23 – 8.00 (m, 2H), 7.79 – 7.60 (m, 6H), 7.52 – 7.40 (m, 5H), 7.40 – 7.32 (m, 1H), 7.14 – 6.93 (m, 5H).

^{13}**C NMR (101 MHz, CDCl$_3$):** δ 158.0, 155.0, 142.7, 139.5, 137.6, 137.0, 134.0, 131.1, 130.3, 129.6, 129.5, 129.2, 128.9, 128.9, 128.2, 127.7, 127.7, 127.4, 126.4, 126.1, 125.0, 122.6, 120.1, 104.6.

^{77}**Se NMR (76 MHz, CDCl$_3$):** δ 304.06.

HRMS (ES$^+$): m/z calculated for [$C_{29}H_{22}NSe$] [M+H]$^+$: 464.0917, measured: 464.0922.

FTIR (Neat): 3057, 2976, 2389, 1685, 1531, 1386, 1020, 788, 750 cm^{-1}.

Synthesis of diphenyl(3-phenyl-4-(phenylselanyl)isoquinolin-1-yl)phosphine oxide (52):

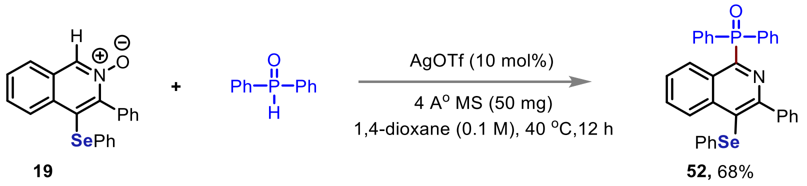

An oven-dried 7 mL reaction vial was charged with isoquinoline N-oxide **19** (38 mg, 0.1 mmol), silver triflate (2.5 mg 0.01 mmol) and diphenylphosphine oxide (24 mg, 0.12 mmol) in 1,4-dioxane (1.0 mL, 1 M). To this mixture was added 4 Å MS (50 mg) added, and the mixture was stirred at 40 °C for 12 hours. After completion of the reaction, the mixture was cooled to room temperature and concentrated under reduced pressure. The crude product was purified by silica gel flash chromatography (PE:EA = 8:2) to afford 38 mg (68% yield) of the desired product **52** as a white sticky solid.

^{1}**H NMR (400 MHz, CDCl$_3$)** δ 9.41 (d, *J* = 8.3 Hz, 1H), 8.49 (d, *J* = 8.4 Hz, 1H), 7.95 – 7.90 (m, 4H), 7.70 – 7.58 (m, 3H), 7.54 – 7.50 (m, 3H), 7.44 (td, *J* = 7.5, 3.0 Hz, 6H), 7.38 – 7.34 (m, 3H), 7.09 – 7.03 (m, 3H), 6.99 – 6.97 (m, 2H).

^{13}**C NMR (101 MHz, CDCl$_3$):** δ 156.7, 156.0, 155.8, 155.4, 141.1, 138.9 (d), 132.8, 132.6, 132.5, 132.3, 131.9 (d), 131.7, 130.8, 130.6, 130.5, 130.2, 129.6, 129.3, 128.5, 128.3, 128.2, 127.7, 127.5, 126.6, 124.9 (d), 104.7, 29.8.

^{31}**P NMR (162 MHz, CDCl$_3$):** δ 27.19.

^{77}**Se NMR (76 MHz, CDCl$_3$):** δ 316.22.

HRMS (ES$^+$): m/z calculated for [$C_{33}H_{25}NOPSe$] [M+H]$^+$: 562.0839, measured: 562.0845.

FTIR (Neat): 3057, 2228, 1813, 1575, 1475, 1342, 1117, 1068, 997, 858, 765 cm^{-1}.

Synthesis of 2-(3-phenyl-4-(phenylselanyl)isoquinolin-1-yl)phenol (53):

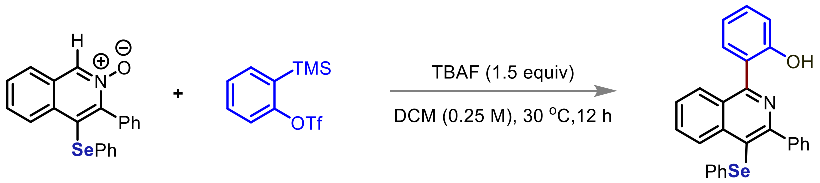

An oven-dried 7 mL reaction vial was charged with isoquinoline N-oxide **19** (38 mg, 0.1 mmol), 2-(Trimethylsilyl)phenyl trifluoromethanesulfonate (34 µL, 0.14 mmol). To this mixture was added dry DCM (0.275 mL, 0.25 M). TBAF 1.0 M in THF (150 µL, 0.15 mmol) was then added, and the mixture was stirred at 30 °C for 12 hours. After completion of the reaction, the mixture was concentrated under reduced pressure. The crude product was purified by silica gel flash chromatography (PE:EA = 9:1) to afford 31 mg (69% yield) of the desired product **53** as a light white sticky solid.

^{1}H NMR (400 MHz, $CDCl_3$): δ 8.13 (d, J = 6.0 Hz, 1H), 7.47 – 7.25 (m, 11H), 7.17 (dt, J = 14.5, 6.1 Hz, 4H), 6.95 – 6.77 (m, 2H), 5.72 (s, 1H).

^{13}C NMR (101 MHz, $CDCl_3$): δ 158.0, 155.0, 142.7, 139.5, 137.6, 136.9, 133.8, 131.1, 130.3, 129.6, 129.5, 129.2, 128.9, 128.9, 128.2, 127.7, 127.7, 127.4, 126.4, 126.1, 124.9, 122.6, 120.1, 104.6.

^{77}Se NMR (76 MHz, $CDCl_3$): δ 453.57.

HRMS (ES$^+$): m/z calculated for [$C_{27}H_{20}NOSe$] [M+H]$^+$: 454.0710, measured: 454.0710.

FTIR (Neat): 3053, 2394, 1742, 1576, 1474, 1360, 1105, 930, 735 cm^{-1}.

Synthesis of 6-phenyl-5-(phenylselanyl)indazolo[3,2-a]isoquinoline (54):

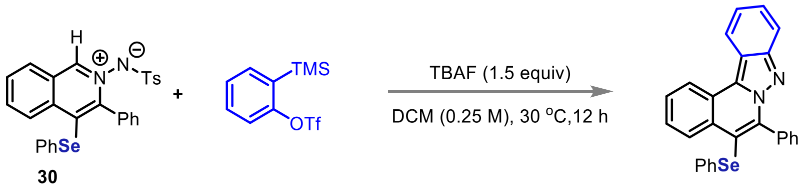

An oven-dried 7 mL reaction vial was charged with isoquinolinium imide **30** (53 mg, 0.1 mmol), 2-(Trimethylsilyl)phenyl trifluoromethanesulfonate (34 µL, 0.14 mmol). To this mixture was added dry DCM (0.275 mL, 0.25 M). TBAF 1.0 M in THF (150 µL, 0.15 mmol was then added, and the mixture was stirred at 30 °C for 12 hours. After completion of the reaction, the mixture was concentrated under reduced pressure. The crude product was purified by silica gel flash chromatography (PE:EA = 8:2) to afford 32.8 mg (73% yield) of the desired product **54** as a light yellow solid.

Melting point: 118-120 °C.

^{1}H NMR (400 MHz, $CDCl_3$): δ 8.79 (d, *J* = 8.0 Hz, 1H), 8.60 (d, *J* = 8.3 Hz, 1H), 8.51 (d, *J* = 8.6 Hz, 1H), 7.91 (d, *J* = 8.7 Hz, 1H), 7.84 – 7.73 (m, 1H), 7.65 – 7.58 (m, 1H), 7.57 – 7.44 (m, 6H), 7.40 – 7.33 (m, 1H), 7.11 (s, 5H).

^{13}C NMR (101 MHz, $CDCl_3$): δ 149.4, 144.2, 135.8, 133.1, 131.7, 130.2, 130.1, 129.9, 129.5, 129.4, 128.8, 128.5, 128.0, 127.7,126.4, 126.2, 123.2, 122.1, 121.3, 118.3, 117.9, 116.7.

^{77}Se NMR (76 MHz, $CDCl_3$): δ 305.87.

HRMS (ES$^+$): m/z calculated for [$C_{27}H_{19}N_2Se$] [M+H]$^+$: 451.0713, measured: 451.0718.

FTIR (Neat): 2997, 2391, 1612, 1575, 1471, 1377, 1244, 1113, 1020, 789, 746 cm^{-1}.

Synthesis of 2-(4-methoxyphenyl)-5-phenyl-6-(phenylselanyl)pyrazolo[5,1-a]isoquinoline (55):

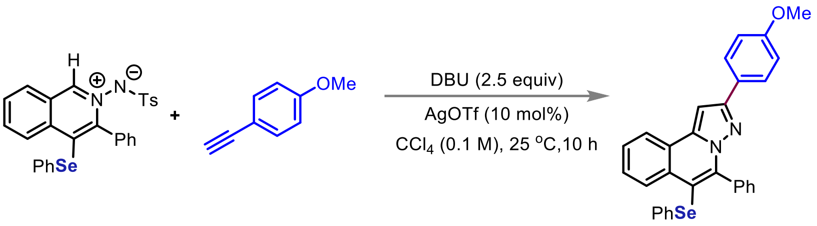

30 **55**, 74%

An oven-dried 7 mL reaction vial was charged with isoquinoline N-oxide **30** (53 mg, 0.1 mmol), 4-Ethynylanisole (20 mg, 0.15 mmol), 1,8-Diazabicyclo[5.4.0]undec-7-ene (DBU) (37.4 μL, 0.25 mmol) and $AgBF_4$ (4.4 mg, 0.0225 mmol). CCl_4 (1 mL, 0.1 M) was then added, and the mixture was stirred at 25 °C for 10 hours. After completion of the reaction, the mixture was concentrated under reduced pressure. The crude product was purified by silica gel flash chromatography (PE:EA = 8.5:1.5) to afford 37 mg (74% yield) of the desired product **55** as a light yellow solid.

Melting point: 203-205 °C.

^{1}H NMR (400 MHz, $CDCl_3$): δ 8.40 (d, *J* = 7.7 Hz, 1H), 8.18 (dd, *J* = 7.8, 0.8 Hz, 1H), 7.94 – 7.76 (m, 2H), 7.70 – 7.43 (m, 7H), 7.34 (s, 1H), 7.22 – 7.06 (m, 5H), 7.00 – 6.88 (m, 2H), 3.84 (s, 3H).

^{13}C NMR (101 MHz, $CDCl_3$): δ 160.0, 153.1, 144.6, 140.9, 135.4, 133.5, 130.5, 130.4, 129.6, 129.3, 129.1, 129.0, 128.66, 128.1, 128.0, 127.9, 126.0, 125.9, 125.8, 124.3, 123.9, 114.1, 111.9, 94.8, 55.4.

^{77}Se NMR (76 MHz, $CDCl_3$): δ 316.24.

HRMS (ES$^+$): m/z calculated for [$C_{30}H_{23}N_2OSe$] [M+H]$^+$: 507.0976, measured: 507.0977.

FTIR (Neat): 3013, 2315, 1575, 1519, 1471, 1311, 1242, 1022, 914, 756 cm^{-1}.

4. References:

[26] H.-F. Yao, D.-L. Wang, F.-H. Li, B. Wu, Z.-J. Cai, S.-J. Ji, *Org. Biomol. Chem.* **2020**, *18*, 7577–7584.
[27] J. M. Anghinoni, S. S. Ferreira, J. C. Kazmierczak, G. Perin, F. Penteado, E. J. Lenardão, *J. Org. Chem.* **2024**, *89*, 11272–11280.
[28] D. R. Araujo, H. A. Goulart, A. M. Barcellos, R. Cargnelutti, E. J. Lenardão, G. Perin, *J. Org. Chem.* **2021**, *86*, 1721–1729.
[29] H. A. Goulart, D. R. Araujo, A. M. Barcellos, R. G. Jacob, E. J. Lenardão, G. Perin, *Eur. J. Org. Chem.* **2022**, *43*, e202201027.
[30] L. Zhang, W. Xiong, B. Yao, H. Liu, M. Li, Y. Qin, Y. Yu, X. Li, M. Chen, W. Wu, J. Li, J. Wang, H. Jiang, *RSC Adv.* **2022**, *12*, 30248–30252.
[55] H.-S. Yeom, Y. Lee, J.-E. Lee, S. Shin, *Org. Biomol. Chem.* **2009**, *7*, 4744–4752.
[56] T. Miao, Z.-Y. Tian, Y.-M. He, F. Chen, Y. Chen, Z.-X. Yu, Q.-H. Fan, *Angew. Chem. Int. Ed.* **2017**, *56*, 4135–4139.
[57] H. F. von Köller, F. J. Geffers, P. Kalvani, A. Foraita, P.-E. J. Loß, B. Butschke, P. G. Jones, D. B. Werz, *Chem. Commun.* **2023**, *59*, 14697–14700.
[58] M. M. Hansmann, A. López-Andarias, E. Rettenmeier, C. Egler-Lucas, F. Rominger, A. S. K. Hashmi, C. Romero-Nieto, *Angew. Chem. Int. Ed.* **2016**, *55*, 1196–1199.
[59] J. S. Yadav, B. V. S. Reddy, P. S. R. Reddy, A. K. Basak, A. V. Narsaiah, *Adv. Synth. Catal.* **2004**, *346*, 77–82.
[60] T. Nishimura, Y. Washitake, S. Uemura, *Adv. Synth. Catal.* **2007**, *349*, 2563–2571.
[61] R. J. Faggyas, E. D. D. Calder, C. Wilson, A. Sutherland, *J. Org. Chem.* **2017**, *82*, 11585–11593.
[62] A. I. Subota, O. S. Artamonov, A. Gorlova, D. M. Volochnyuk, O. O. Grygorenko, *Tetrahedron Lett.* **2017**, *58*, 1989–1991.
[63] M. Tiano, P. Belmont, *J. Org. Chem.* **2008**, *73*, 4101–4109.
[64] Y.-T. Ma, C. Lin, X.-B. Huang, M.-C. Liu, Y.-B. Zhou, H.-Y. Wu, *Chem. Commun.* **2022**, *58*, 6550–6553.
[65] X. Zhao, W. Fan, Z. Miao, R. Chen, *Synth. Commun.* **2013**, *43*, 1714–1720.
[66] M. E. Domaradzki, Y. Long, Z. She, X. Liu, G. Zhang, Y. Chen, *J. Org. Chem.* **2015**, *80*, 11360–11368.
[67] Q. Ding, J. Wu, *Adv. Synth. Catal.* **2008**, *350*, 1850–1854.
[68] A. S. K. Hashmi, M. Wieteck, I. Braun, P. Nösel, L. Jongbloed, M. Rudolph, F. Rominger, *Adv. Synth. Catal.* **2012**, *354*, 555–562.
[69] Y. L. Choi, C.-M. Yu, B. T. Kim, J.-N. Heo, *J. Org. Chem.* **2009**, *74*, 3948–3951.
[70] K. Sekine, A. Takayanagi, S. Kikuchi, T. Yamada, *Chem. Commun.* **2013**, *49*, 11320–11322.
[71] J. Aziz, J.-D. Brion, M. Alami, A. Hamze, *RSC Adv.* **2015**, *5*, 74391–74398.
[72] J. Lv, H. Liu, L. Lu, R. Hua, *J. Org. Chem.* **2022**, *87*, 16011–16018.
[73] B. G. Das, A. Chirila, M. Tromp, J. N. H. Reek, B. de Bruin, *J. Am. Chem. Soc.* **2016**, *138*, 8968–8975.
[74] F. Xie, Z. Zhang, X. Yu, G. Tang, X. Li, *Angew. Chem. Int. Ed.* **2015**, *54*, 7405–7409.
[75] S. Ortgies, A. Breder, *Org. Lett.* **2015**, *17*, 2748–2751.
[76] M. Wilken, S. Ortgies, A. Breder, I. Siewert, *ACS Catal.* **2018**, *8*, 10901–10912.

4.NMR Spectra

1-(2-(Phenylethynyl)phenyl)ethan-1-one oxime (S1)

^{1}H NMR (400 MHz, $CDCl_3$)

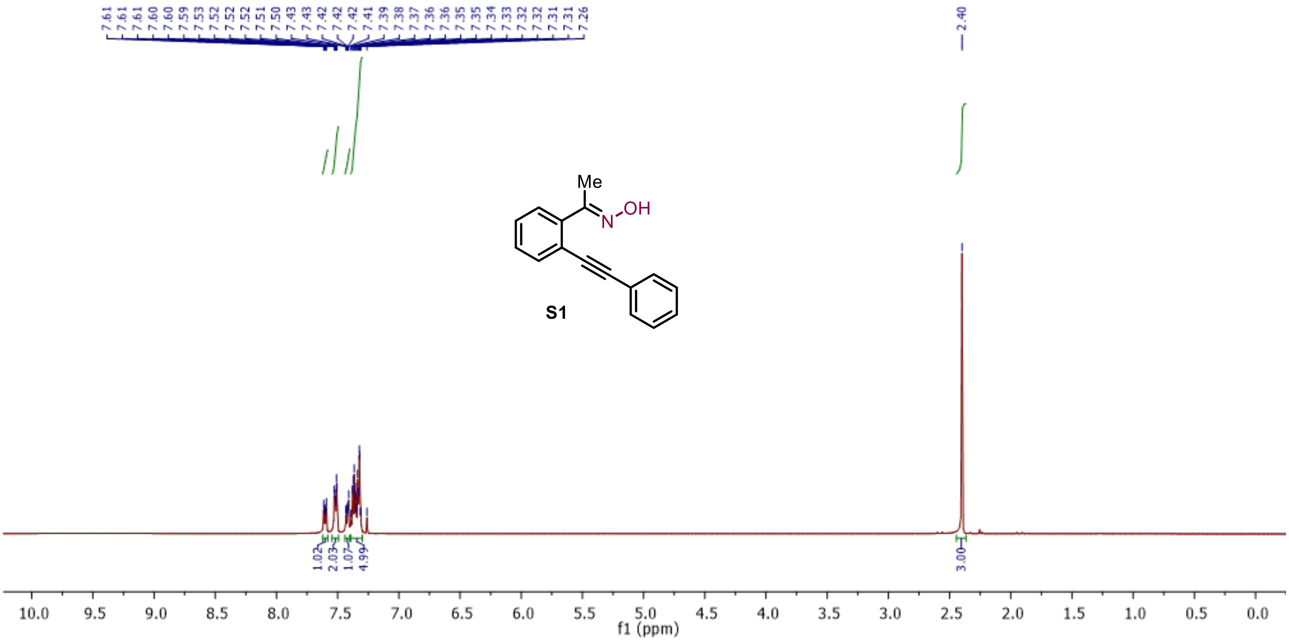

^{13}C NMR (101 MHz, $CDCl_3$)

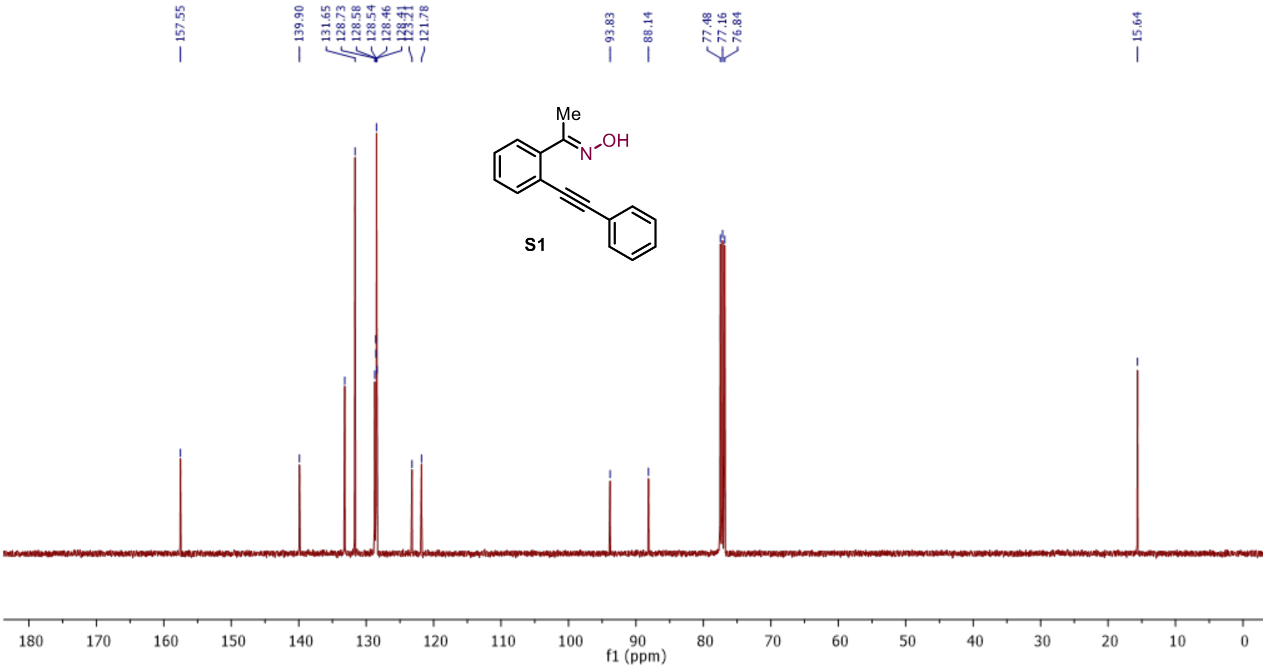

1-(2-((4-Methoxyphenyl)ethynyl)phenyl)ethan-1-one (S2-1)

^{1}H NMR (400 MHz, $CDCl_3$)

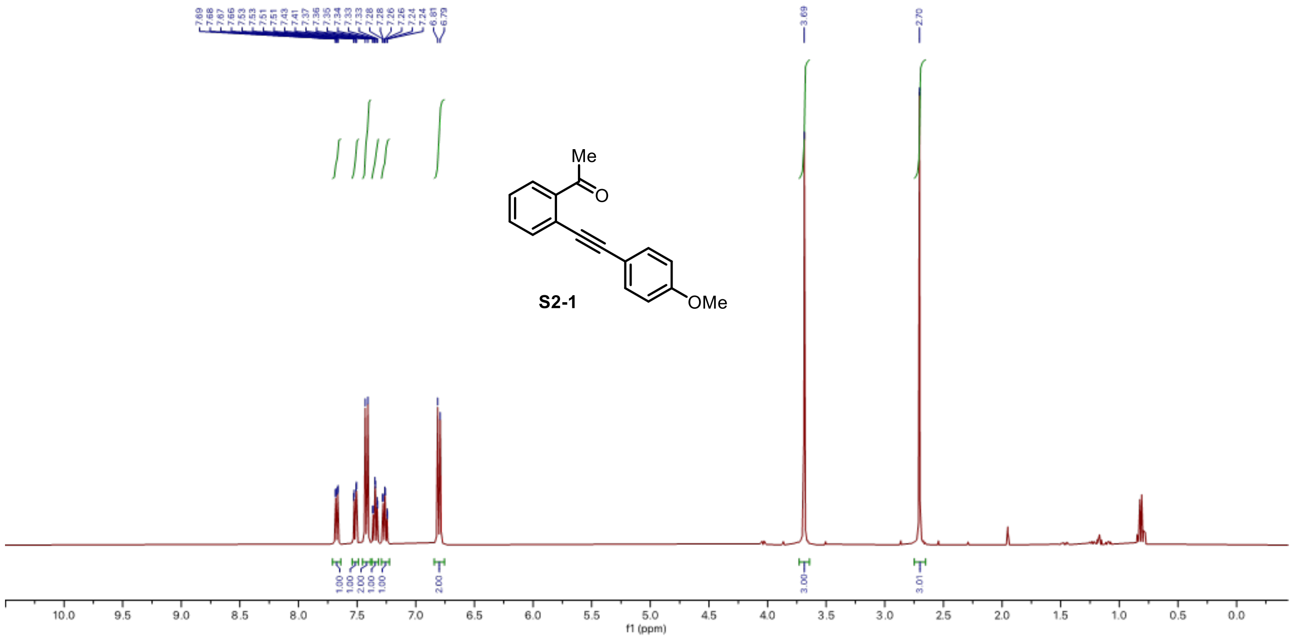

^{13}C NMR (101 MHz, $CDCl_3$)

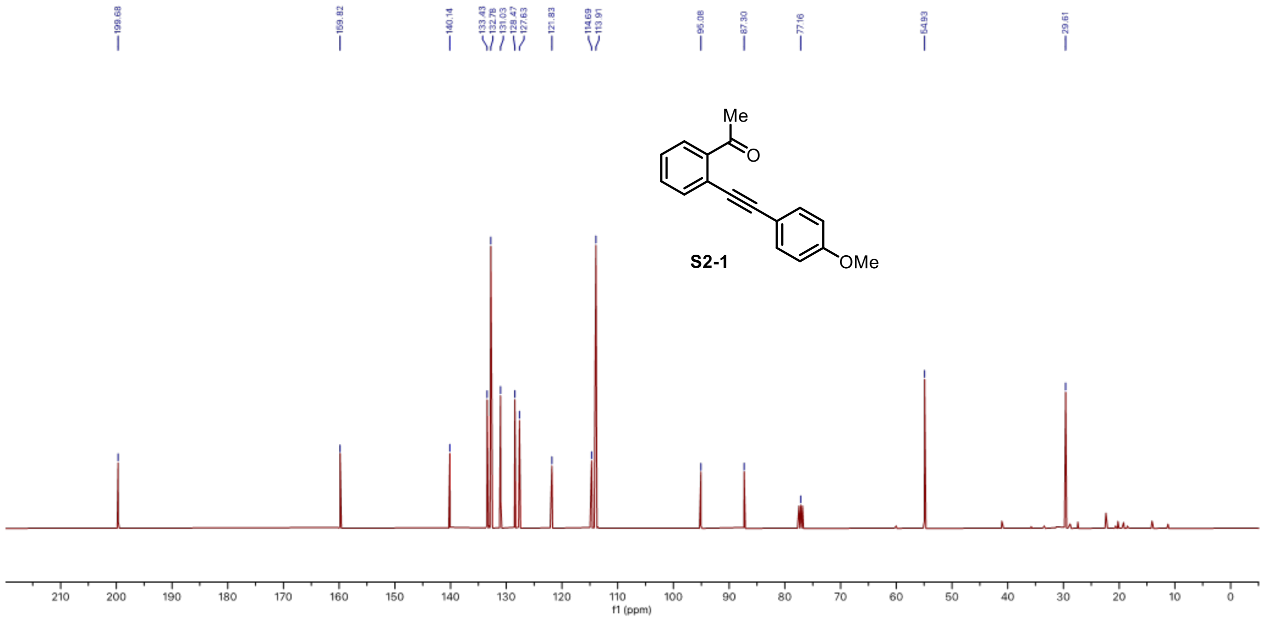

1-(2-((4-Methoxyphenyl)ethynyl)phenyl)ethan-1-one oxime(S2)

^{1}H NMR (400 MHz, $CDCl_3$)

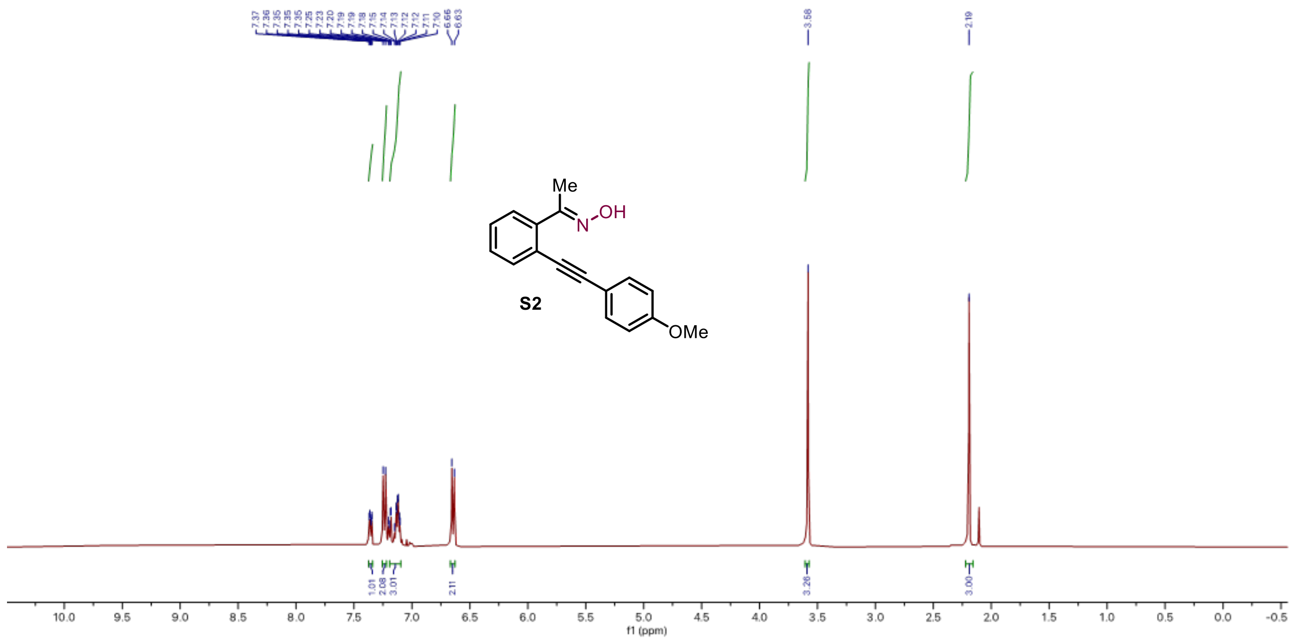

^{13}C NMR (101 MHz, $CDCl_3$)

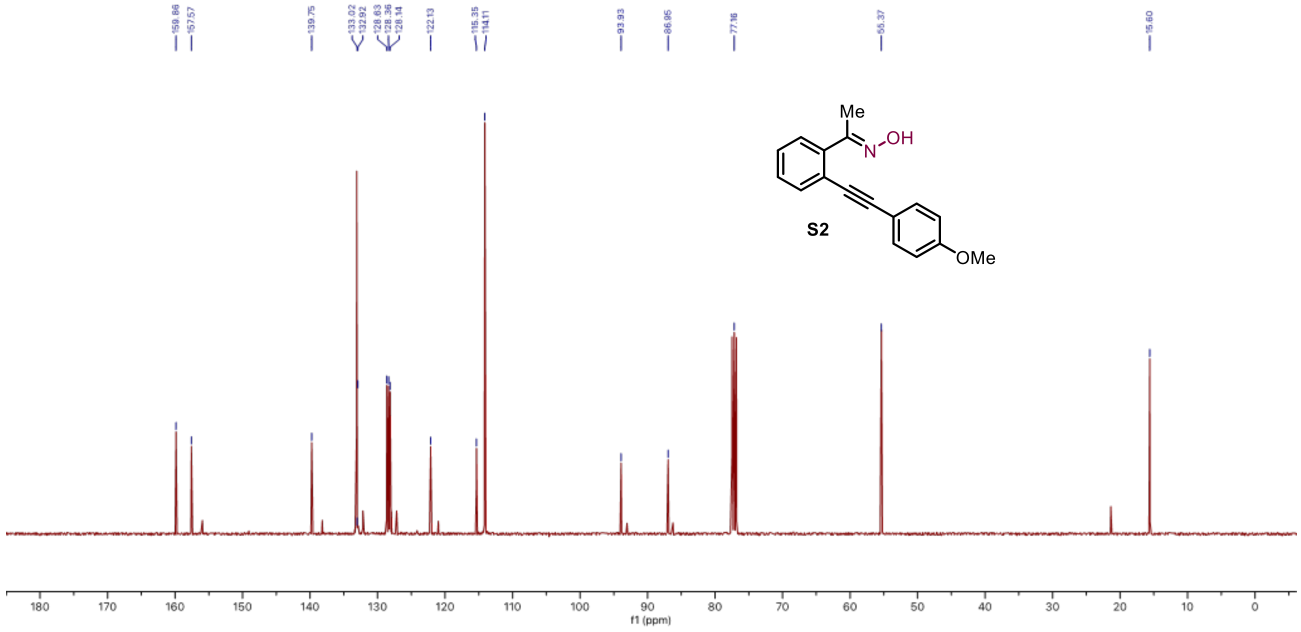

1-(2-(p-Tolylethynyl)phenyl)ethan-1-one (S3-1)

^{1}H NMR (400 MHz, $CDCl_3$)

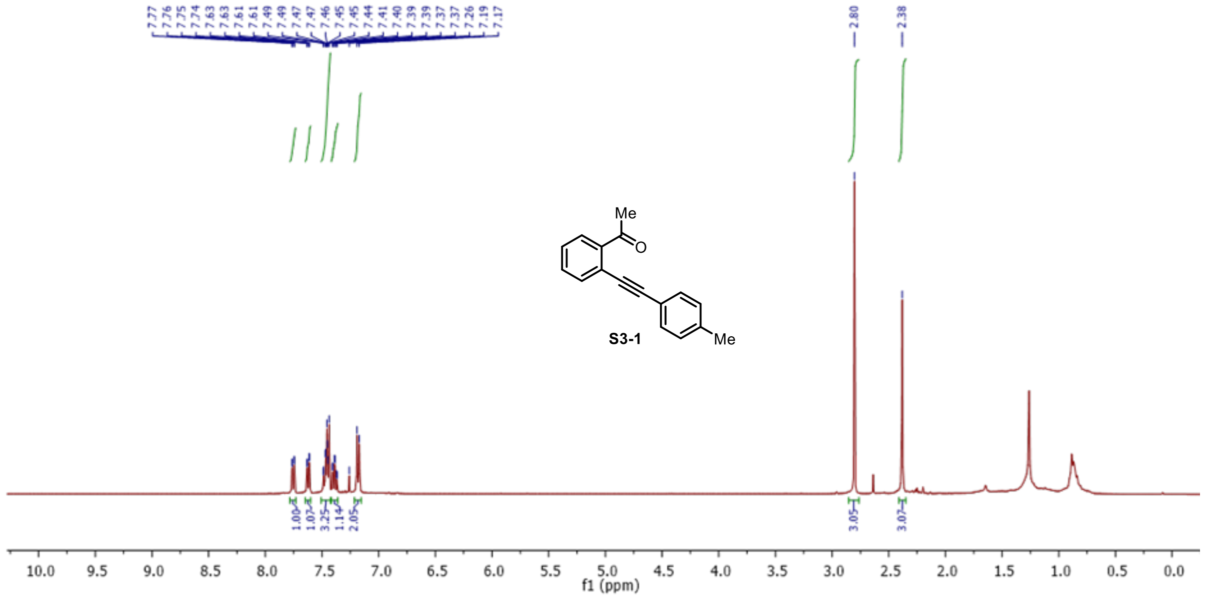

^{13}C NMR (101 MHz, $CDCl_3$)

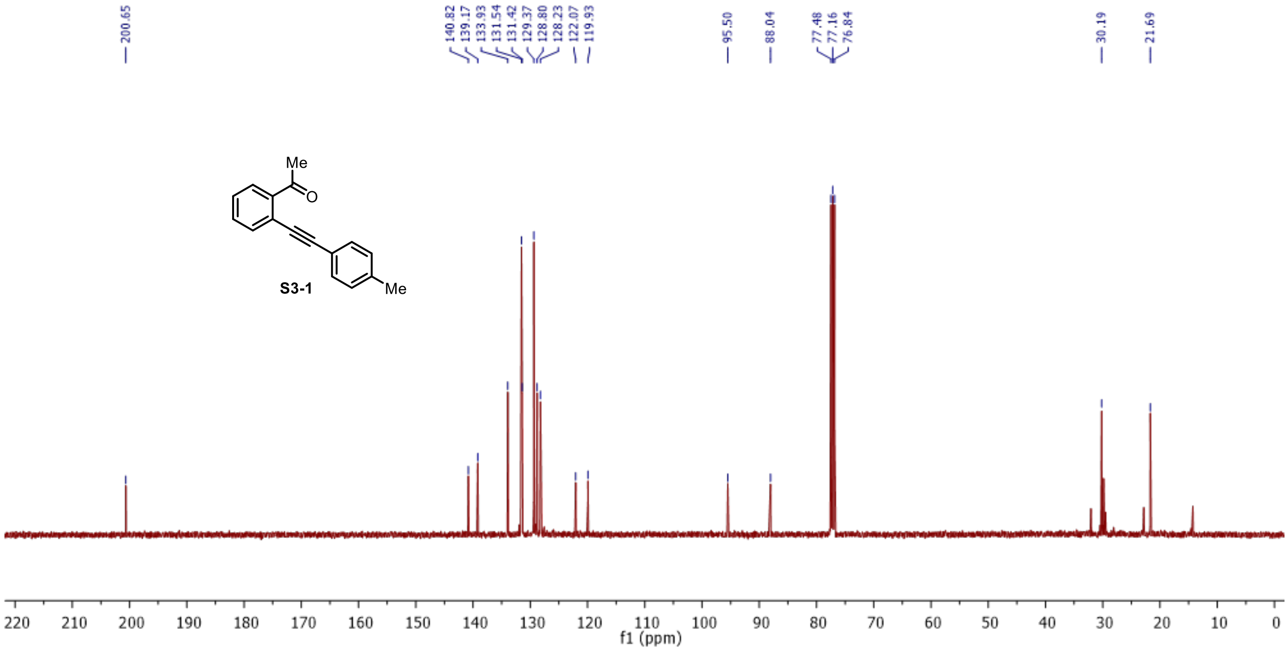

1-(2-(p-Tolylethynyl)phenyl)ethan-1-one oxime (S3)

^{1}H NMR (400 MHz, $CDCl_3$)

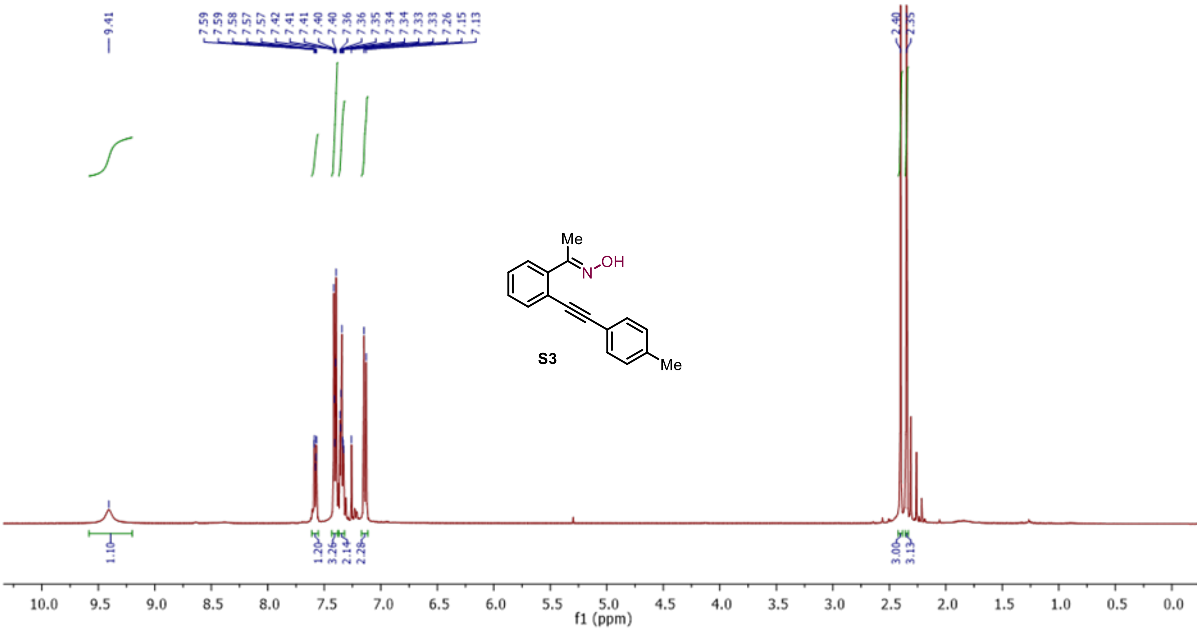

^{13}C NMR (101 MHz, $CDCl_3$)

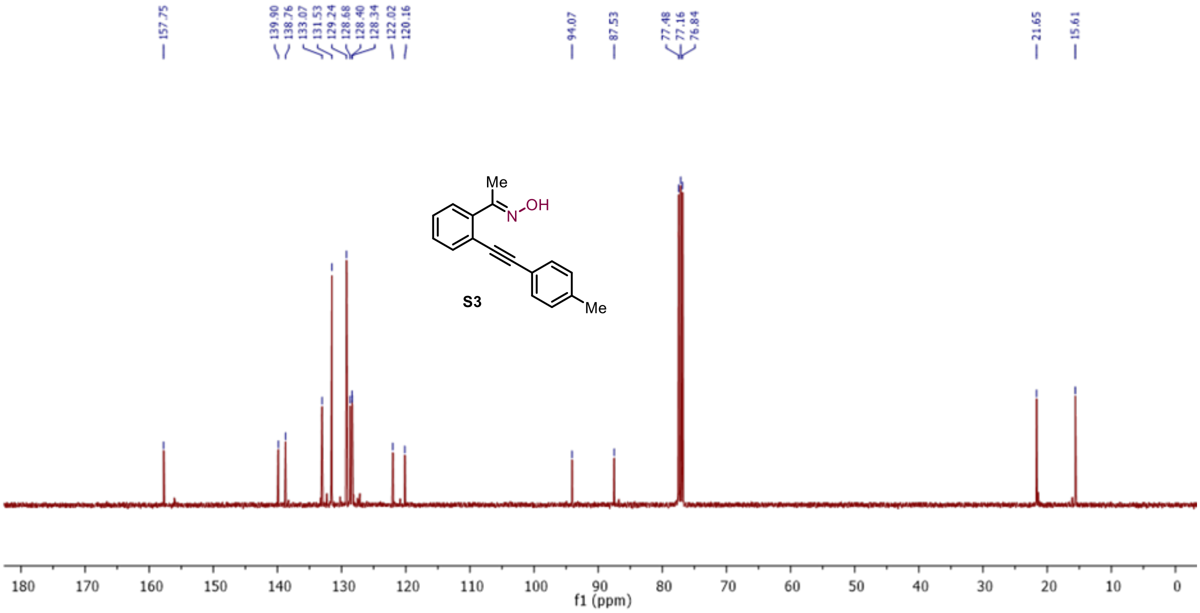

1-(2-((4-Chlorophenyl)ethynyl)phenyl)ethan-1-one oxime (S4):

The NMR spectra show the presence of non-isolable impurities along with the desired product.

^{1}H NMR (400 MHz, $CDCl_3$)

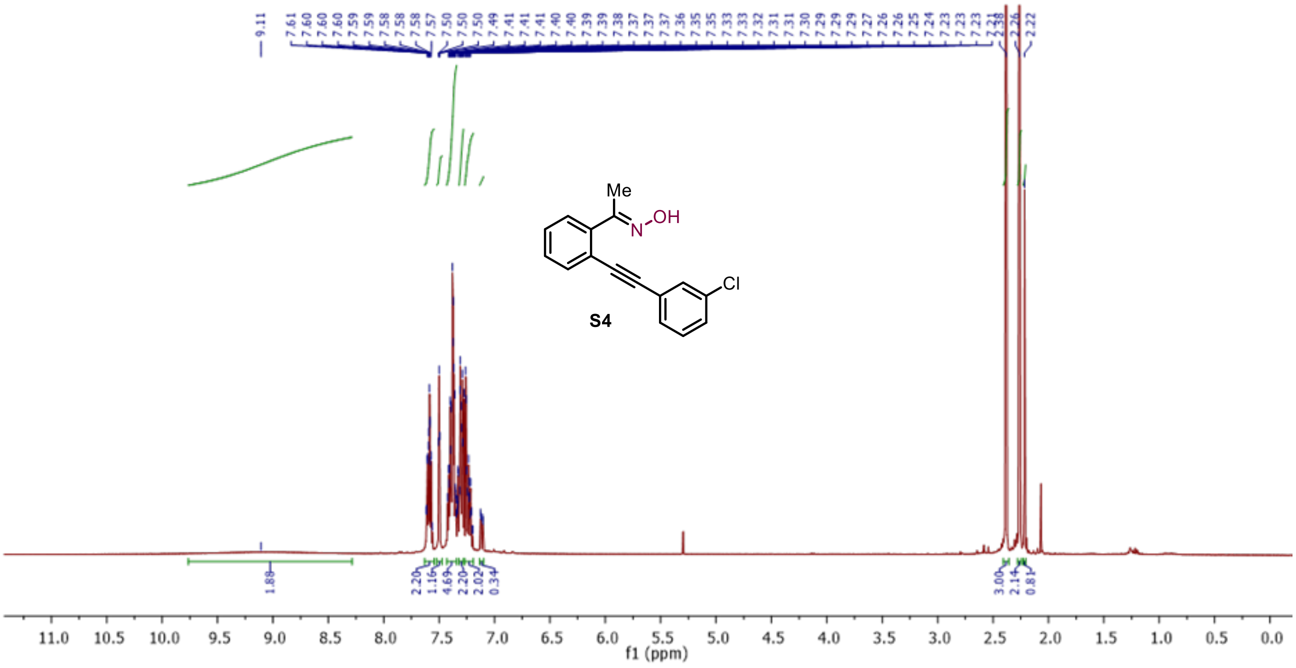

^{13}C NMR (101 MHz, $CDCl_3$)

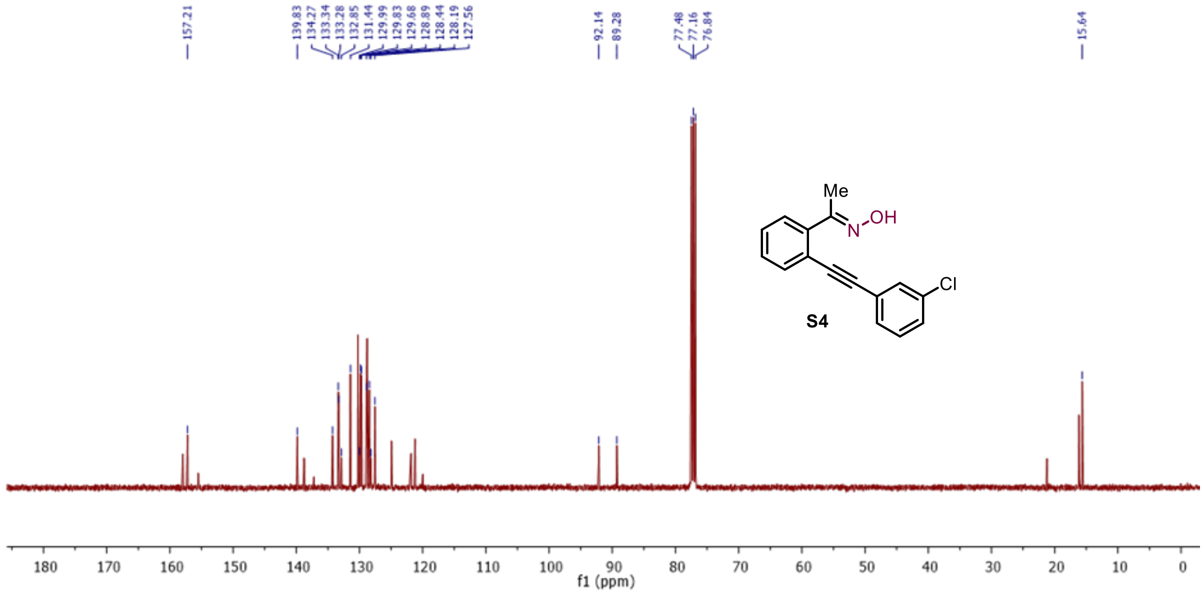

1-(2-((4-Fluorophenyl)ethynyl)phenyl)ethan-1-one oxime (S5)

^{1}H NMR (400 MHz, $CDCl_3$)

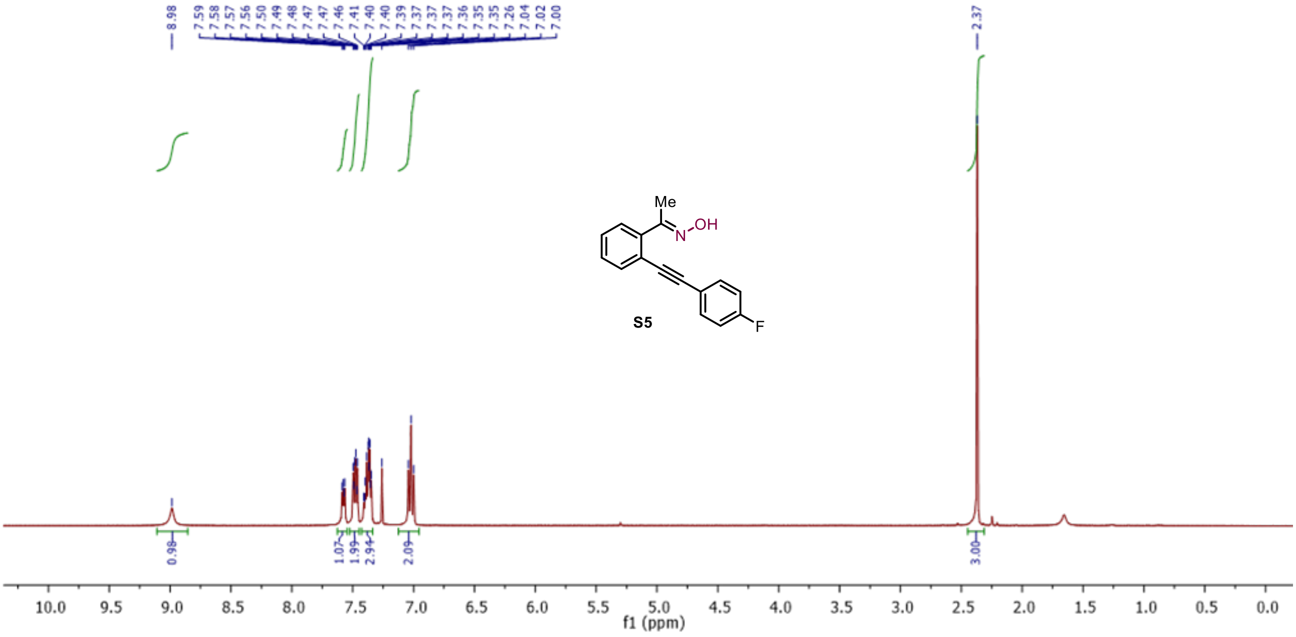

^{13}C NMR (101 MHz, $CDCl_3$)

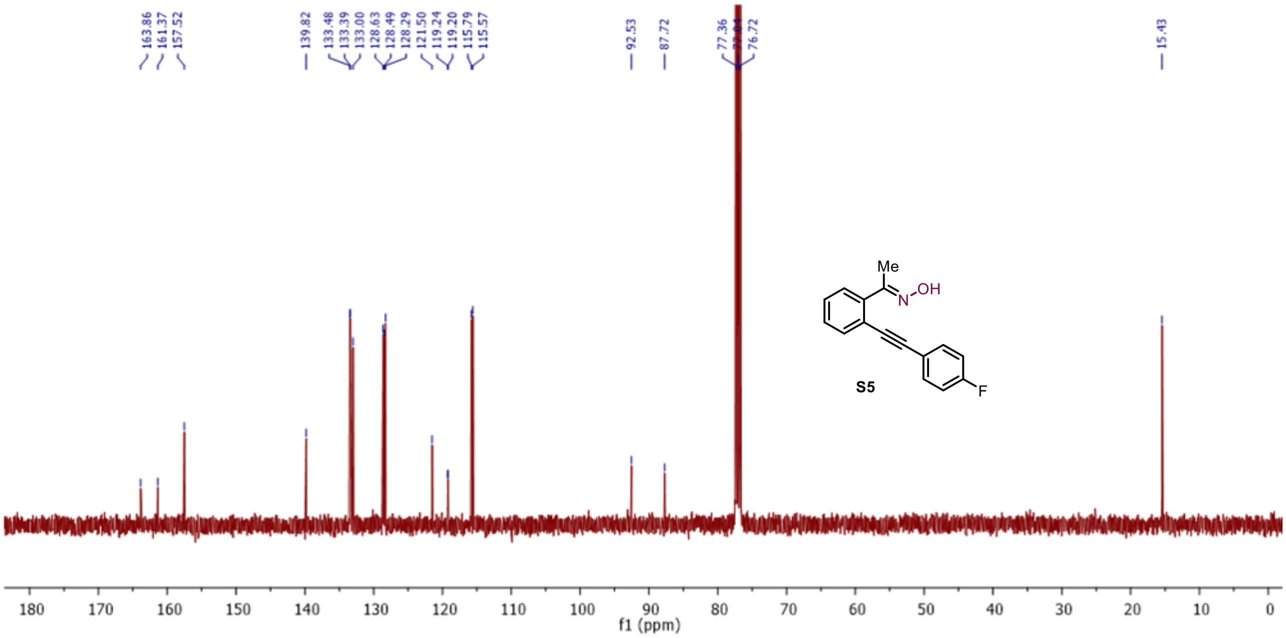

1-(2-((4-(Trifluoromethyl)phenyl)ethynyl)phenyl)ethan-1-one oxime (S6)

The NMR spectra show the presence of non-isolable impurities along with the desired product.

^{1}H NMR (400 MHz, $CDCl_3$)

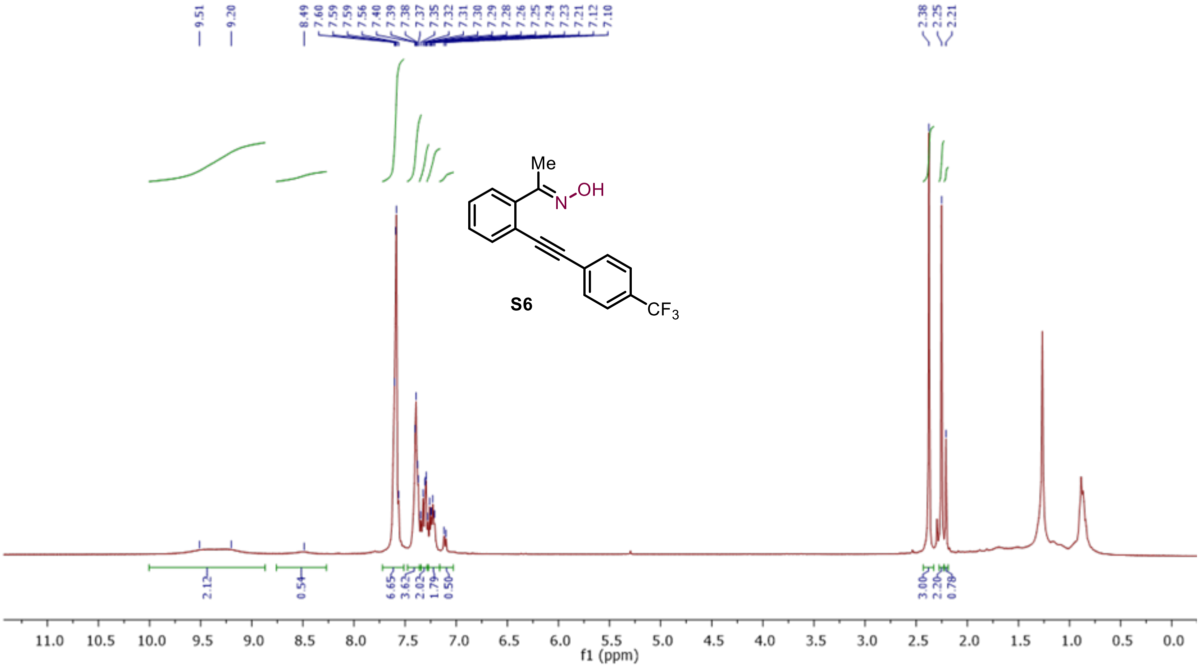

^{13}C NMR (101 MHz, $CDCl_3$)

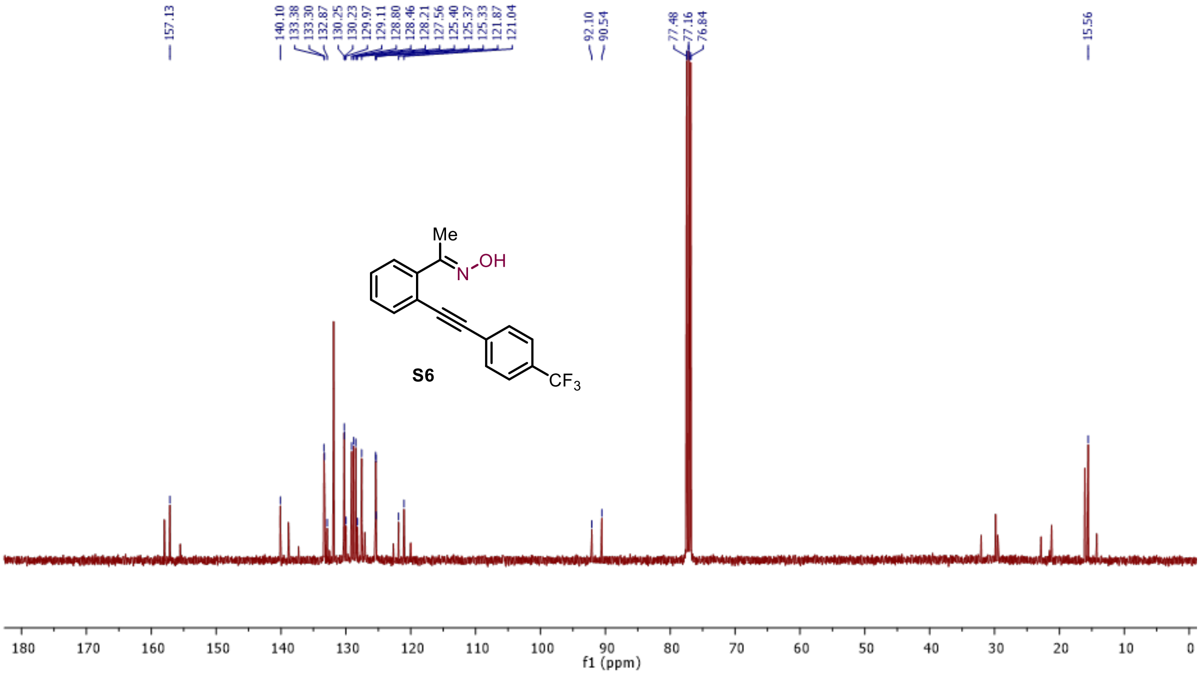

Methyl 4-((2-acetylphenyl)ethynyl)benzoate (S7-1)

^1H NMR (400 MHz, $CDCl_3$)

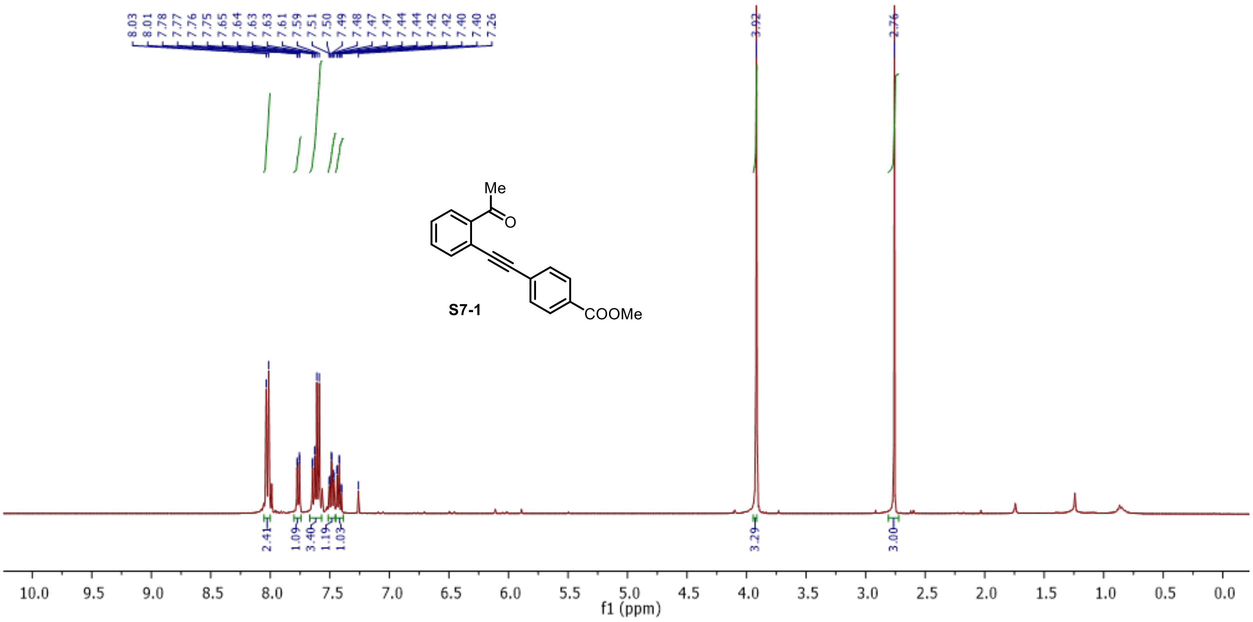

^{13}C NMR (101 MHz, $CDCl_3$)

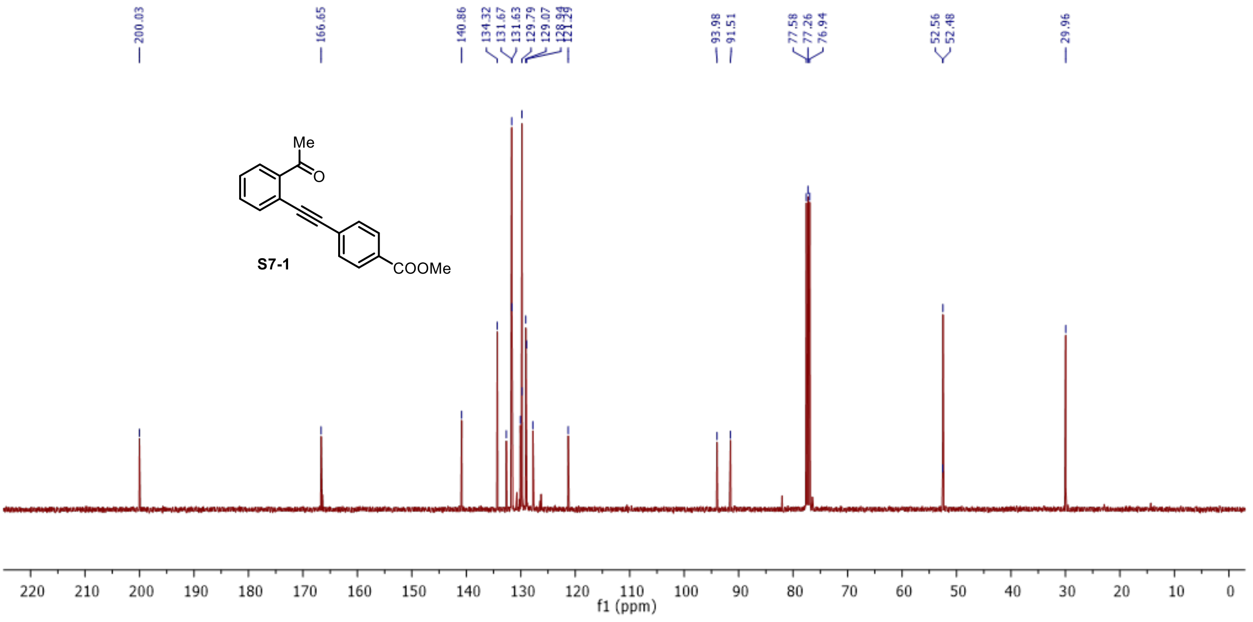

Methyl-4-((2-(1-(hydroxyimino)ethyl)phenyl)ethynyl)benzoate (S7)

^{1}H NMR (400 MHz, $CDCl_3$)

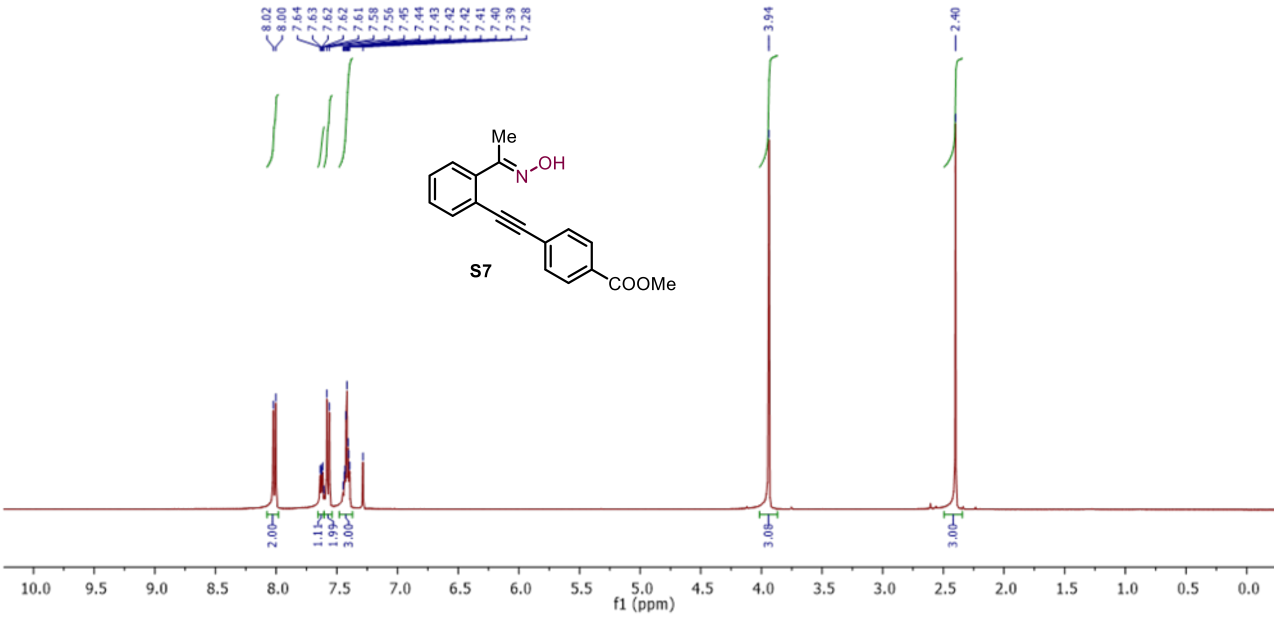

^{13}C NMR (101 MHz, $CDCl_3$)

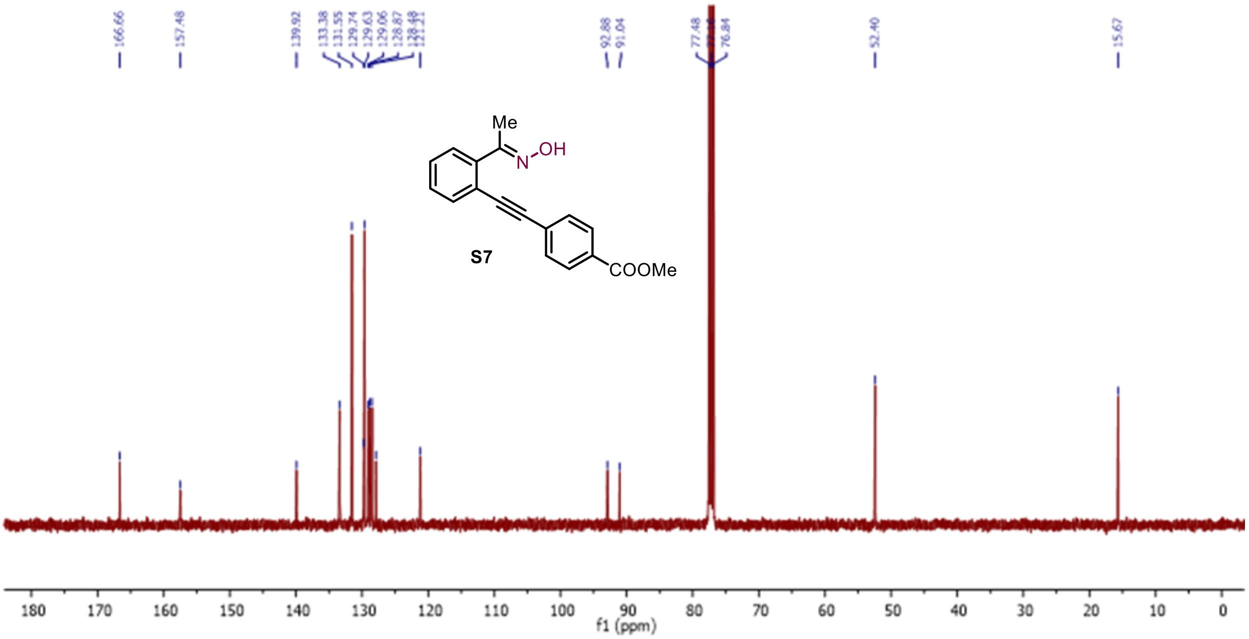

1-(2-((Trimethylsilyl)ethynyl)phenyl)ethan-1-one oxime (S8)

^{1}H NMR (400 MHz, $CDCl_3$)

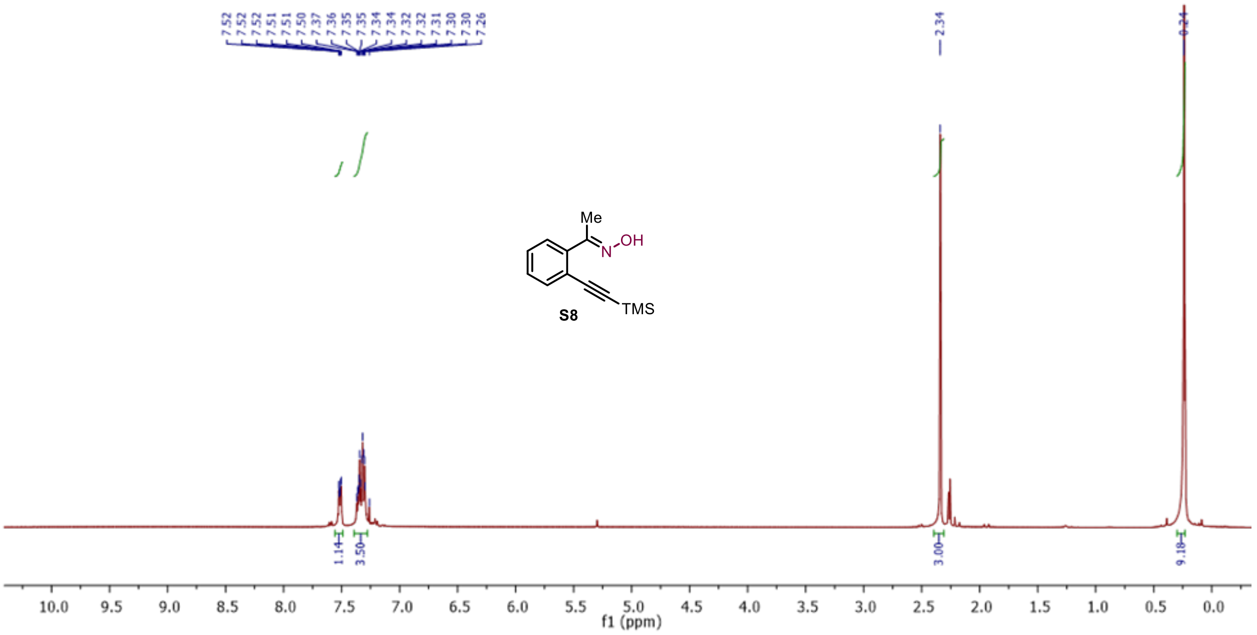

^{13}C NMR (101 MHz, $CDCl_3$)

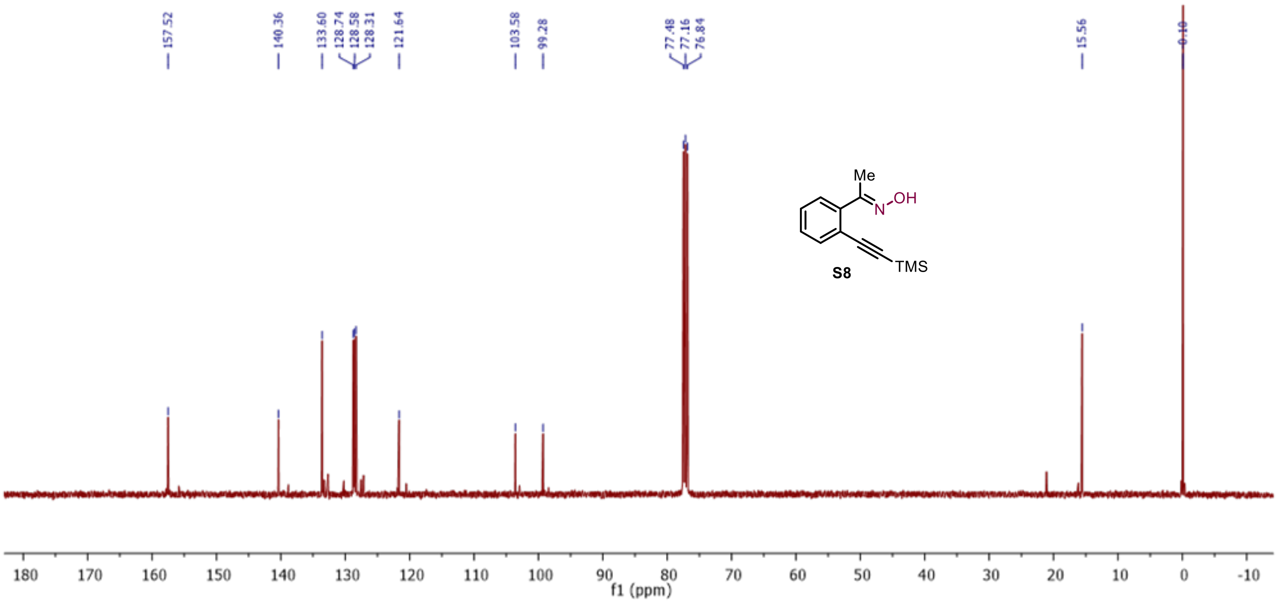

2-(Phenylethynyl)benzaldehyde oxime (S9)

^{1}H NMR (400 MHz, $CDCl_3$)

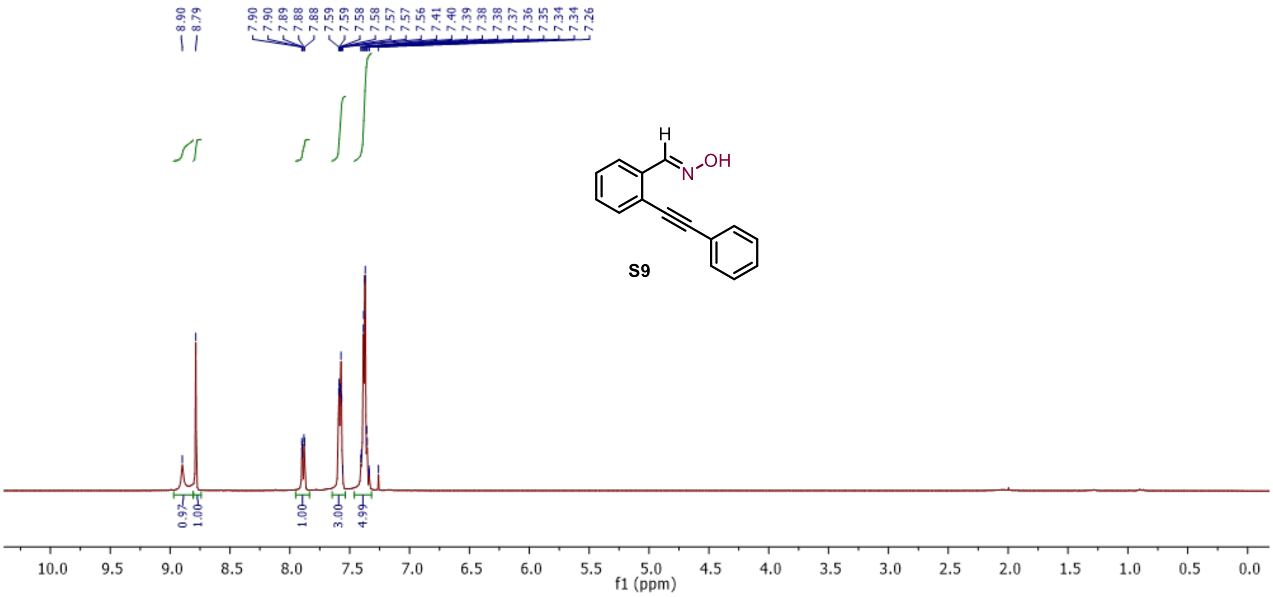

^{13}C NMR (101 MHz, $CDCl_3$)

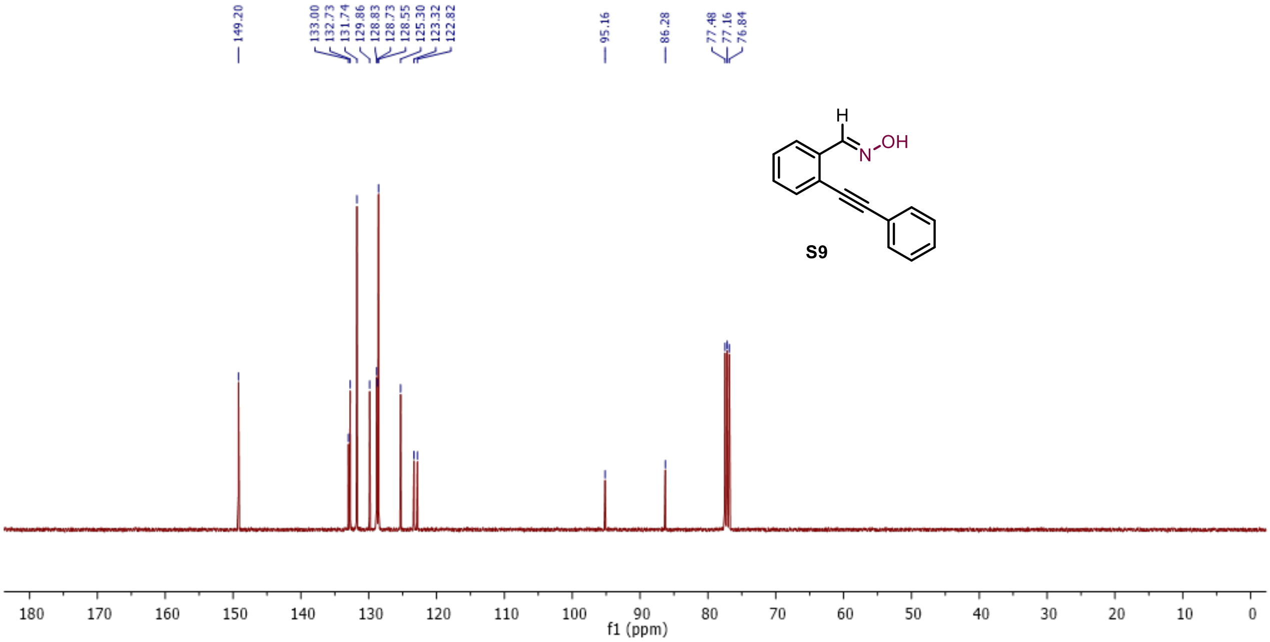

2-((4-Methoxyphenyl)ethynyl)benzaldehyde oxime (S10)

^{1}H NMR (400 MHz, $CDCl_3$)

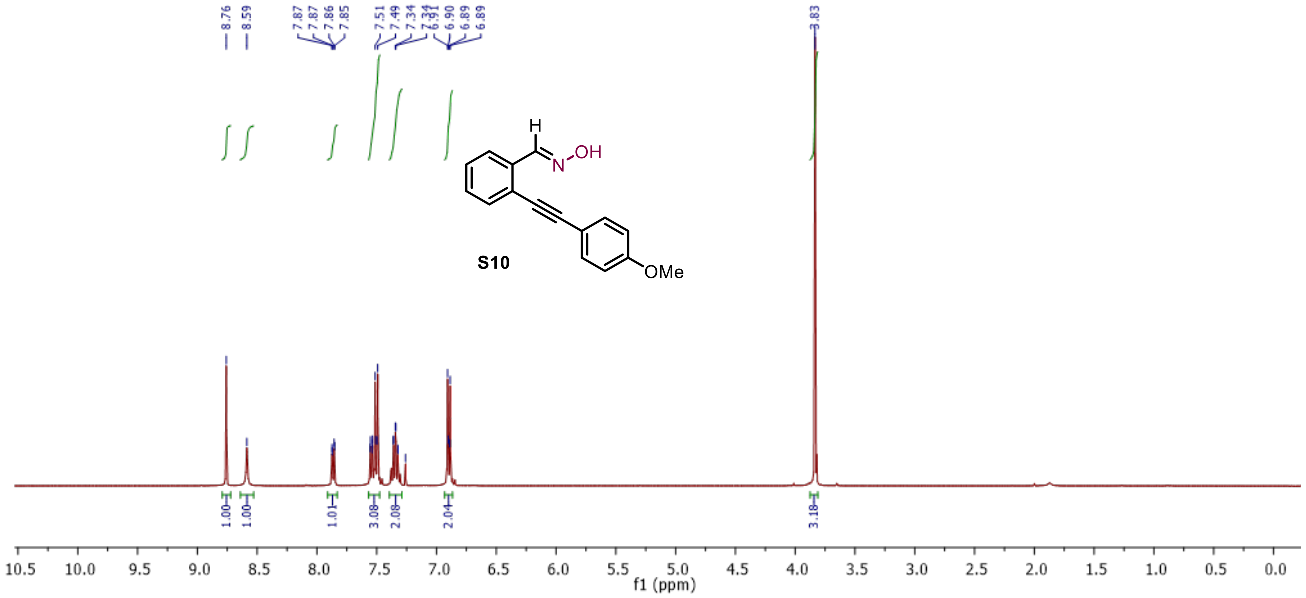

^{13}C NMR (101 MHz, $CDCl_3$)

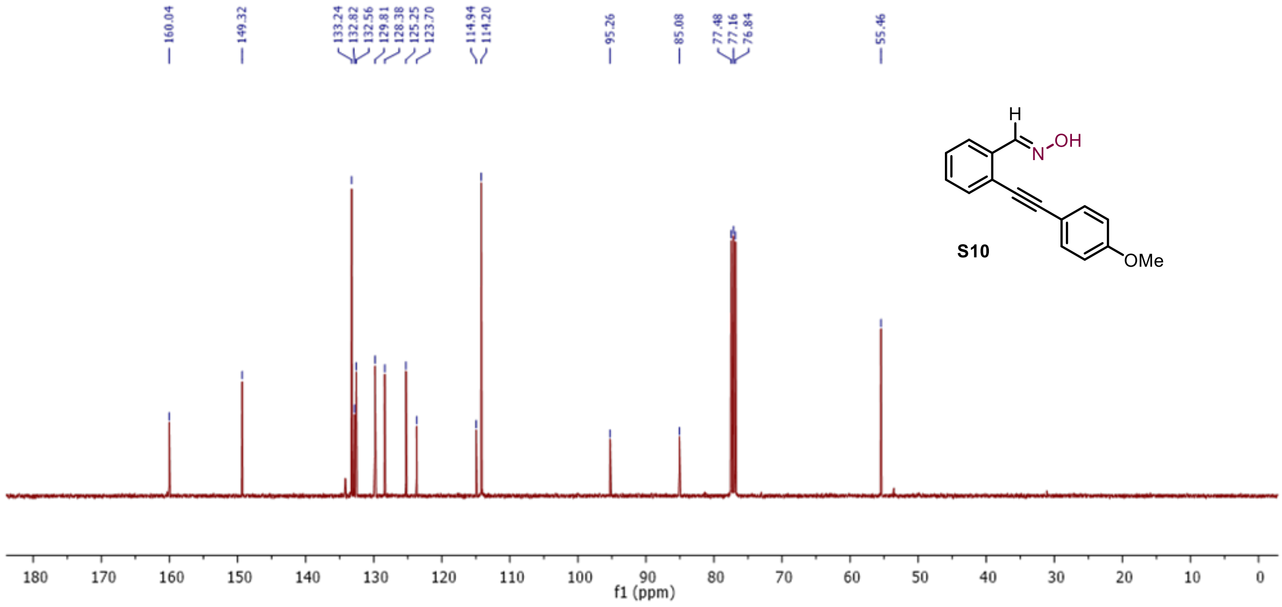

2-(Thiophen-2-ylethynyl)benzaldehyde (S11-1)

^{1}H NMR (500 MHz, $CDCl_3$)

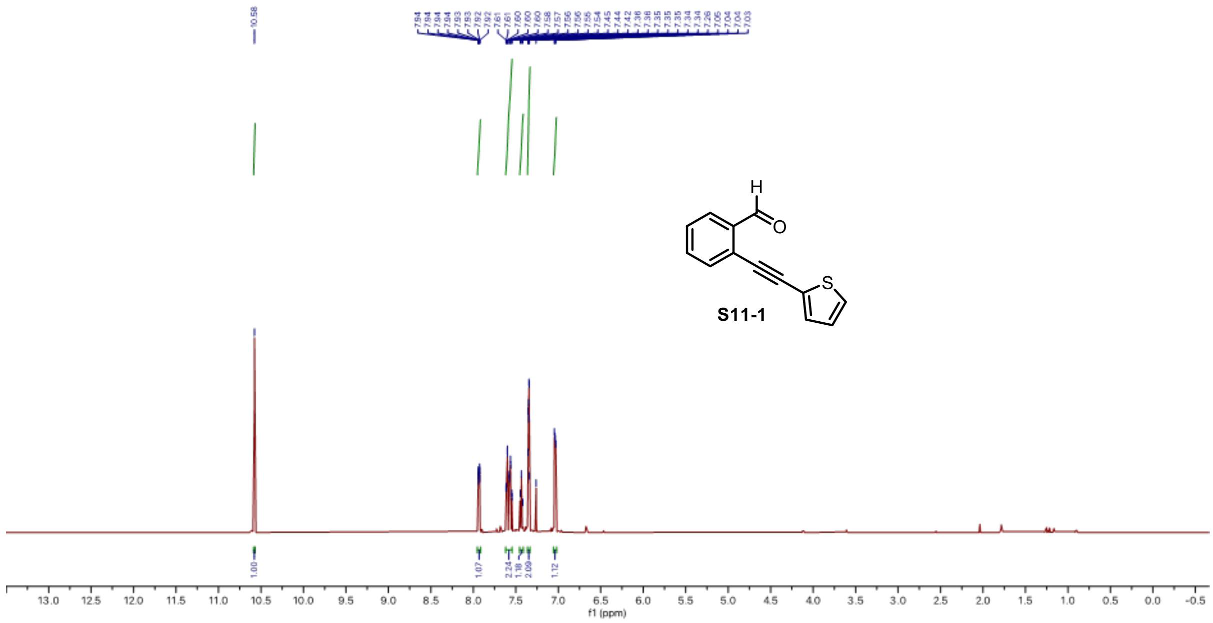

^{13}C NMR (126 MHz, $CDCl_3$)

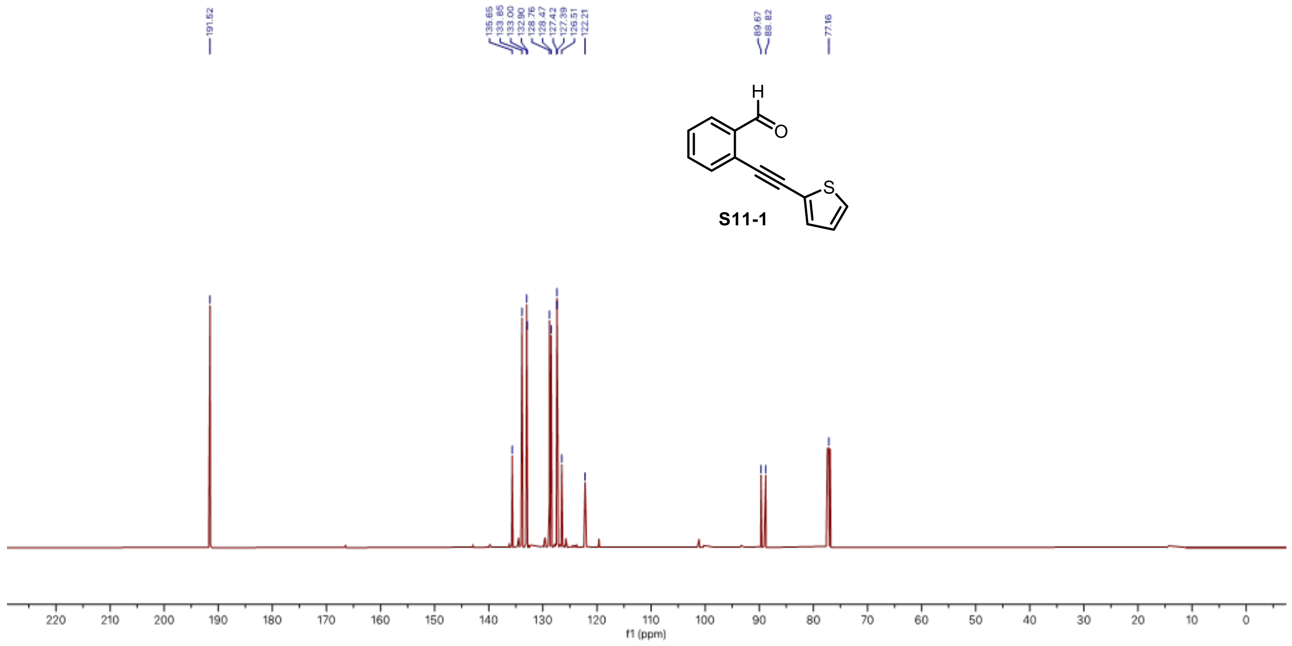

2-(Thiophen-2-ylethynyl)benzaldehyde oxime (S11)

^{1}H NMR (400 MHz, $CDCl_3$)

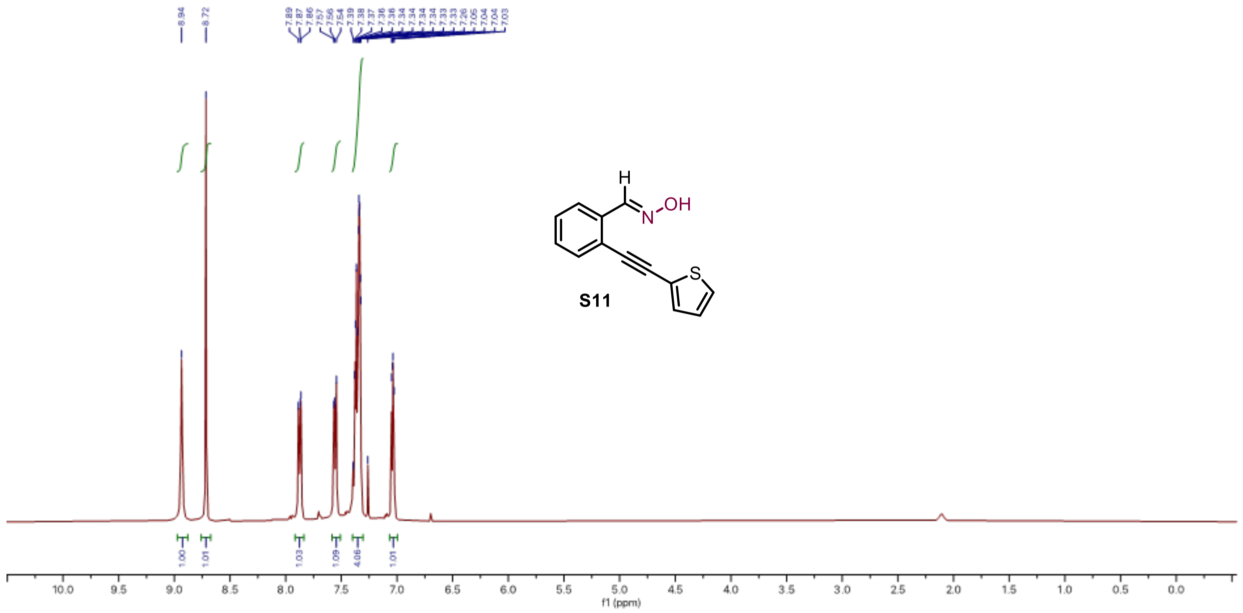

^{13}C NMR (101 MHz, $CDCl_3$)

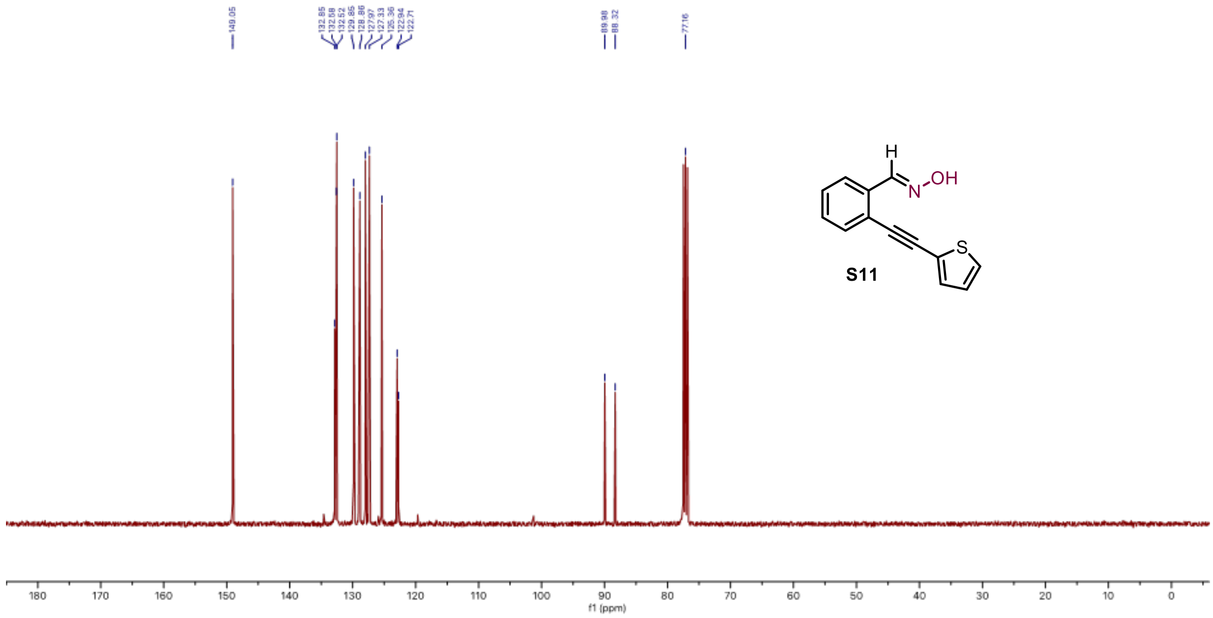

2-((Trimethylsilyl)ethynyl)benzaldehyde oxime (S13)

^{1}H NMR (400 MHz, $CDCl_3$)

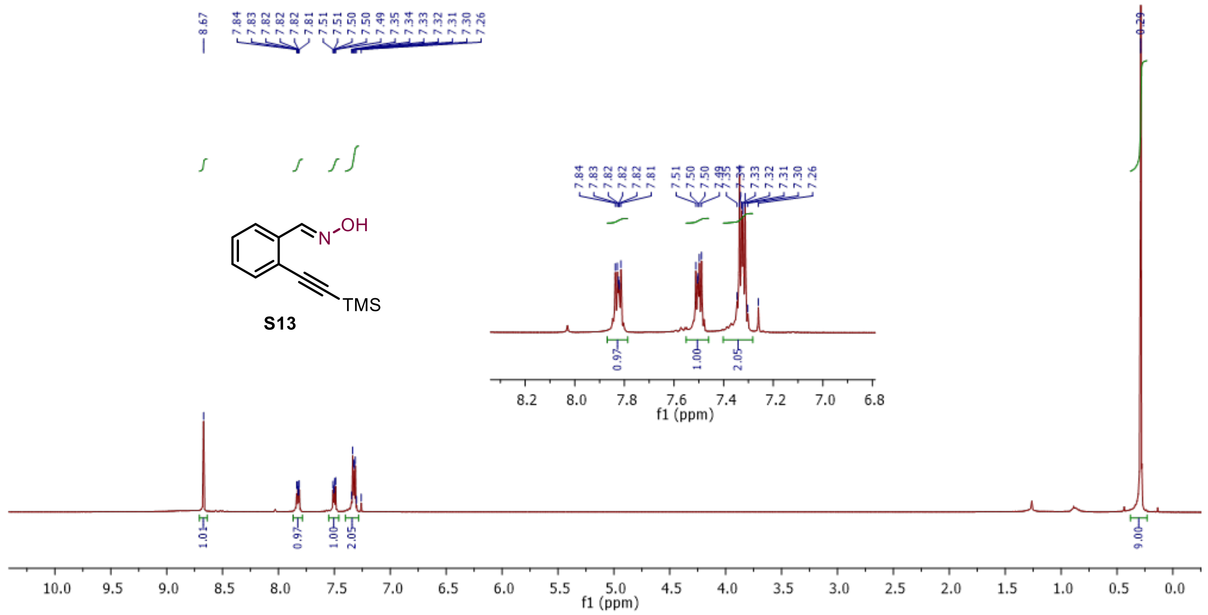

^{13}C NMR (101 MHz, $CDCl_3$)

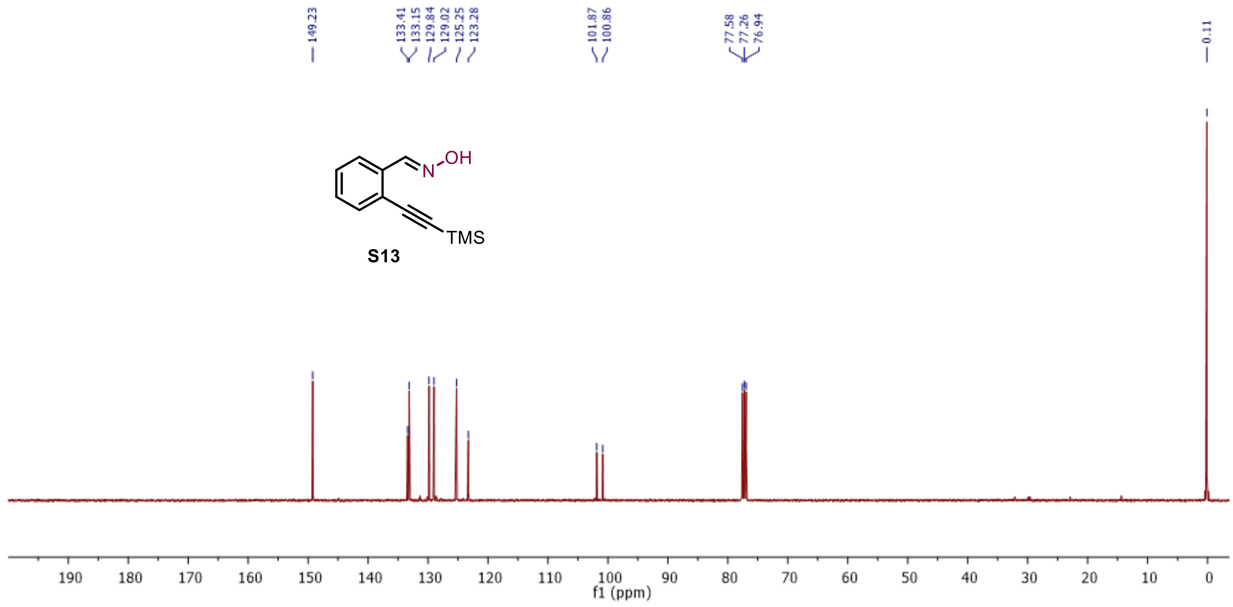

4,5-Dimethoxy-2-(phenylethynyl)benzaldehyde (S14-1)

^{1}H NMR (400 MHz, $CDCl_3$)

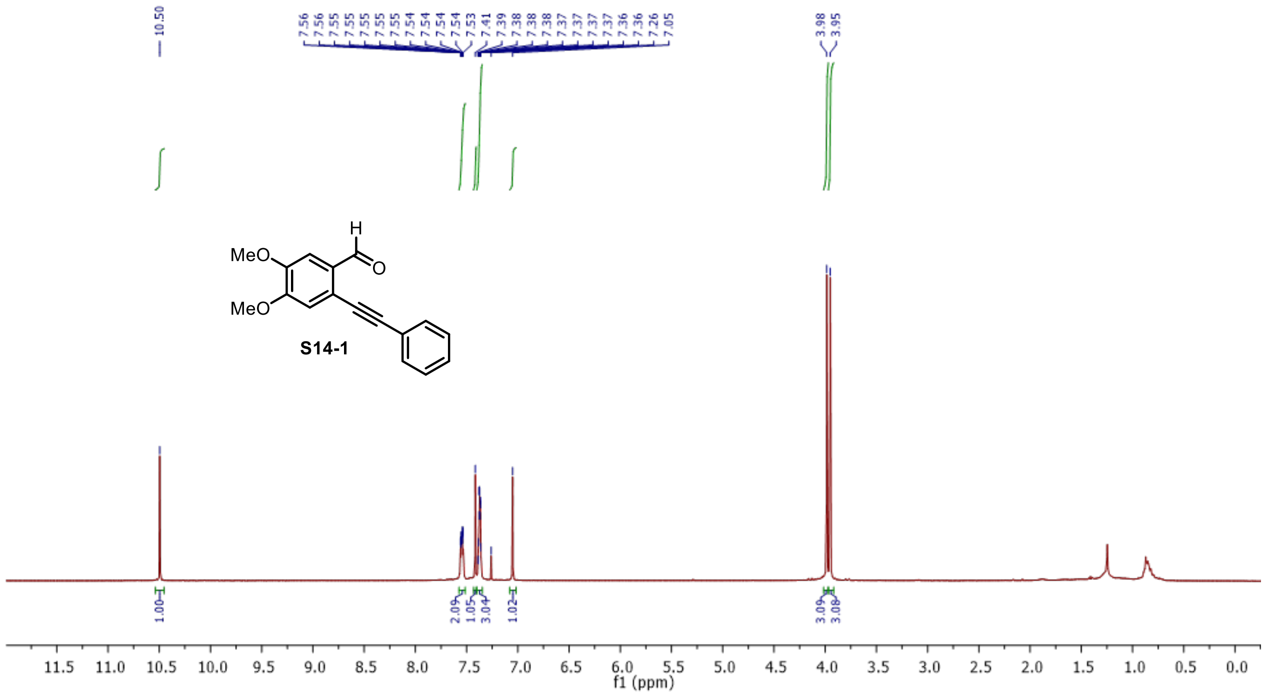

^{13}C NMR (101 MHz, $CDCl_3$)

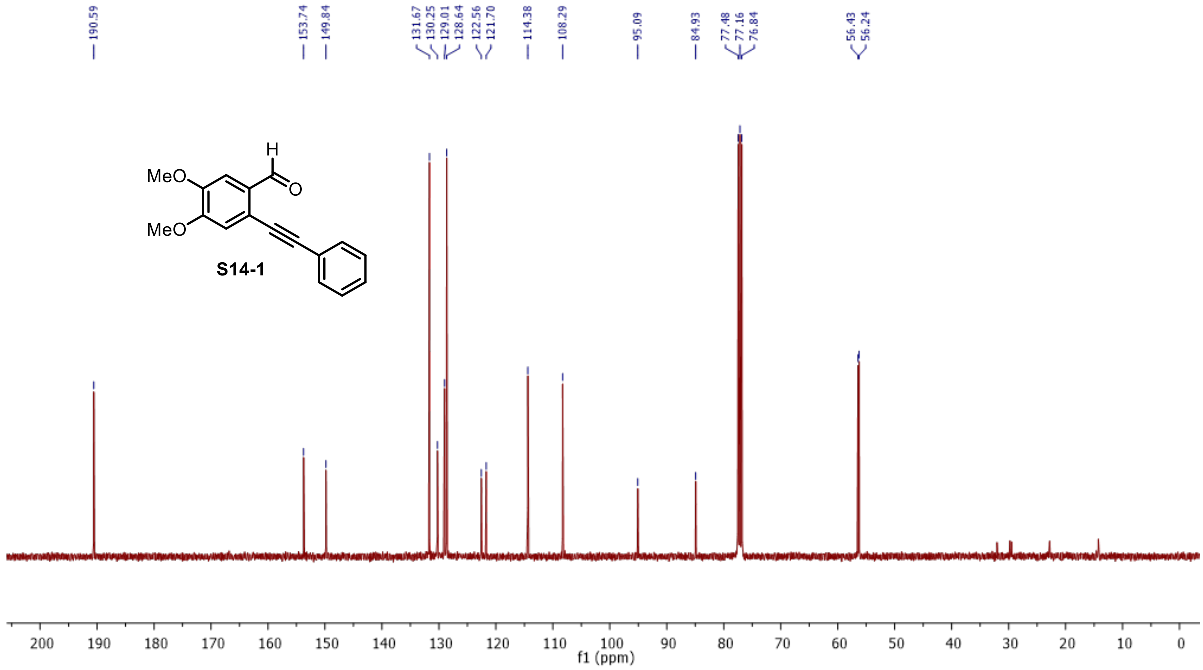

4,5-Dimethoxy-2-(phenylethynyl)benzaldehyde oxime (S14)

^{1}H NMR (400 MHz, $CDCl_3$)

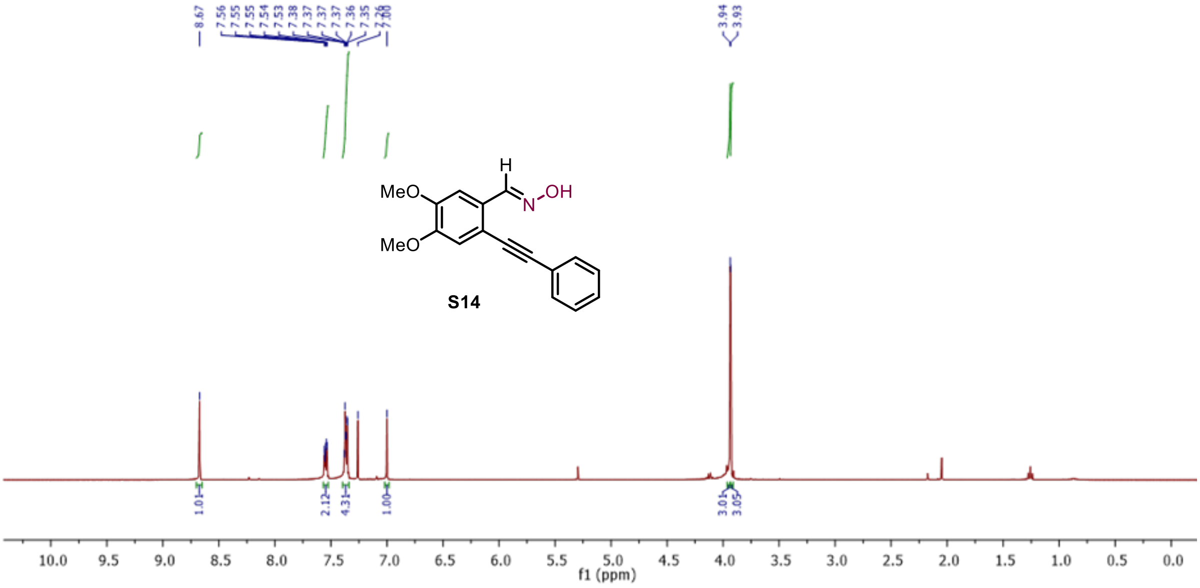

^{13}C NMR (101 MHz, $CDCl_3$)

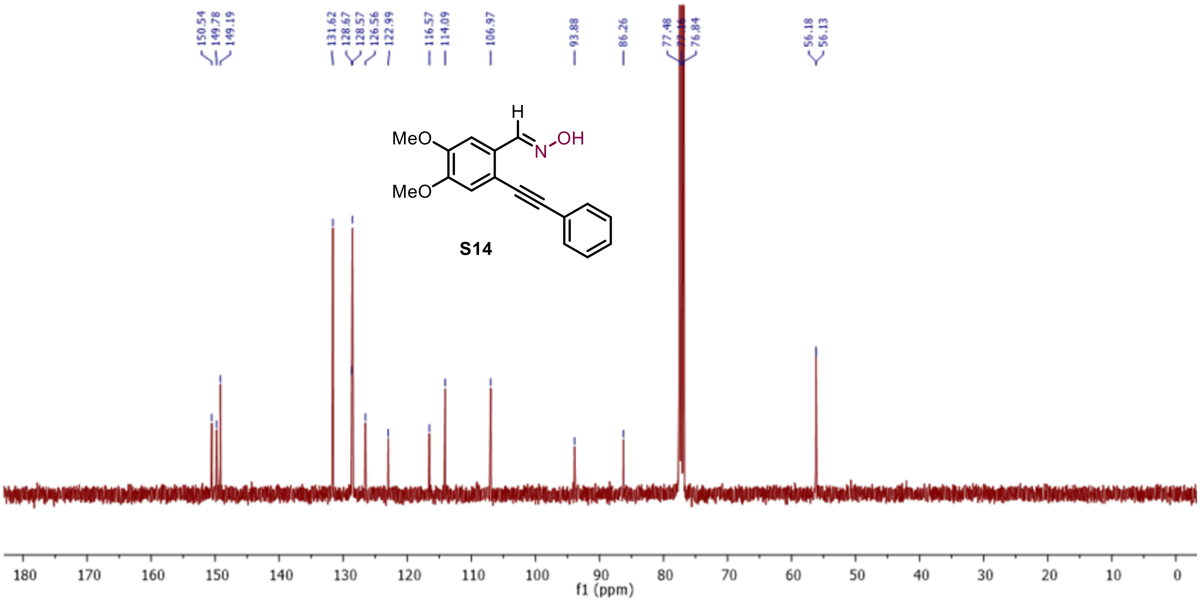

1-(2-Bromo-4,5-dimethoxyphenyl)ethan-1-one (15-1)

^{1}H NMR (400 MHz, $CDCl_3$)

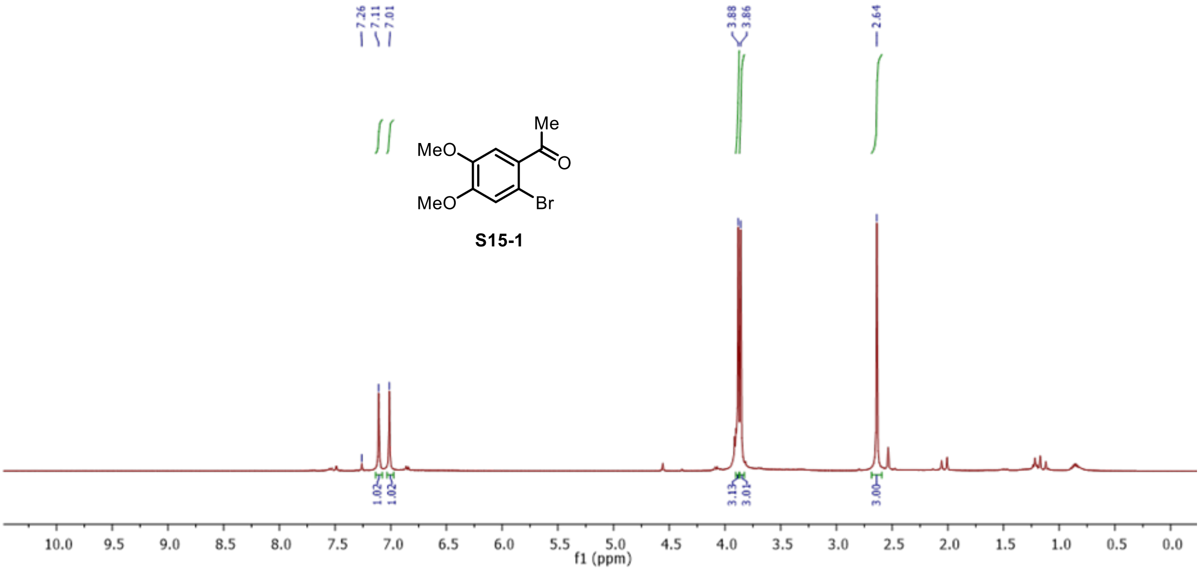

^{13}C NMR (101 MHz, $CDCl_3$)

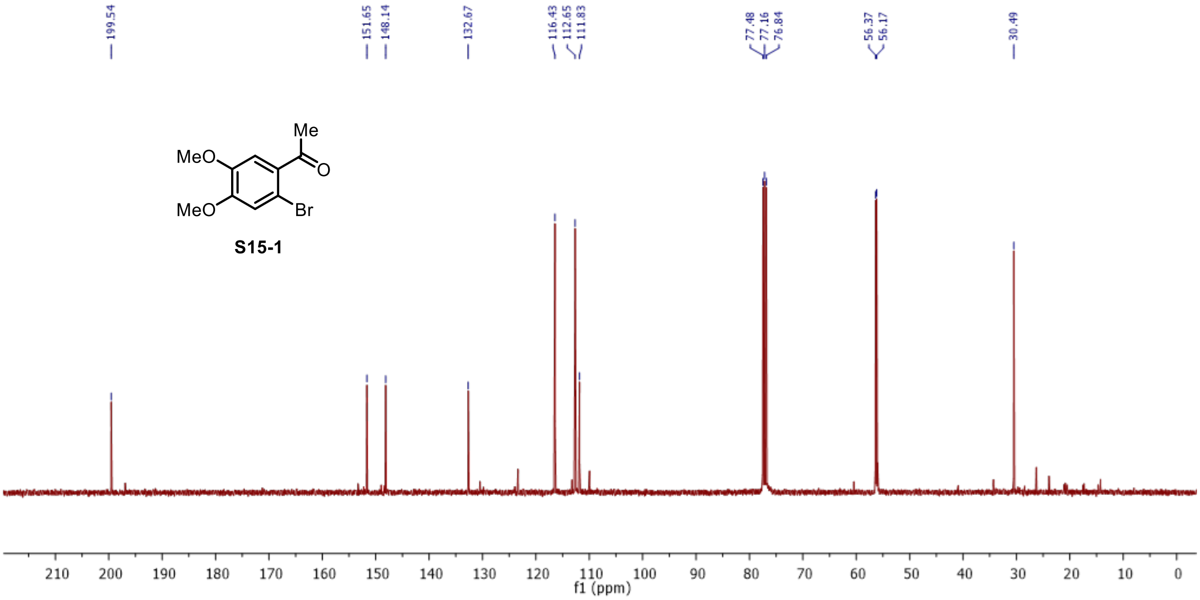

1-(4,5-Dimethoxy-2-(phenylethynyl)phenyl)ethan-1-one (S15-2)

^{1}H NMR (400 MHz, $CDCl_3$)

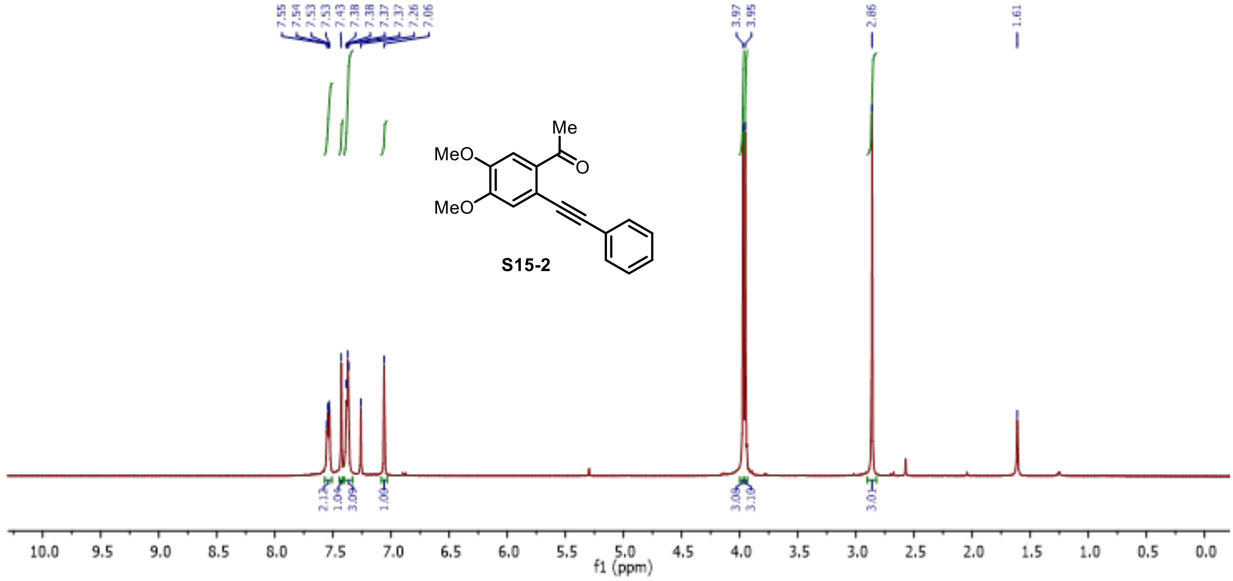

^{13}C NMR (101 MHz, $CDCl_3$)

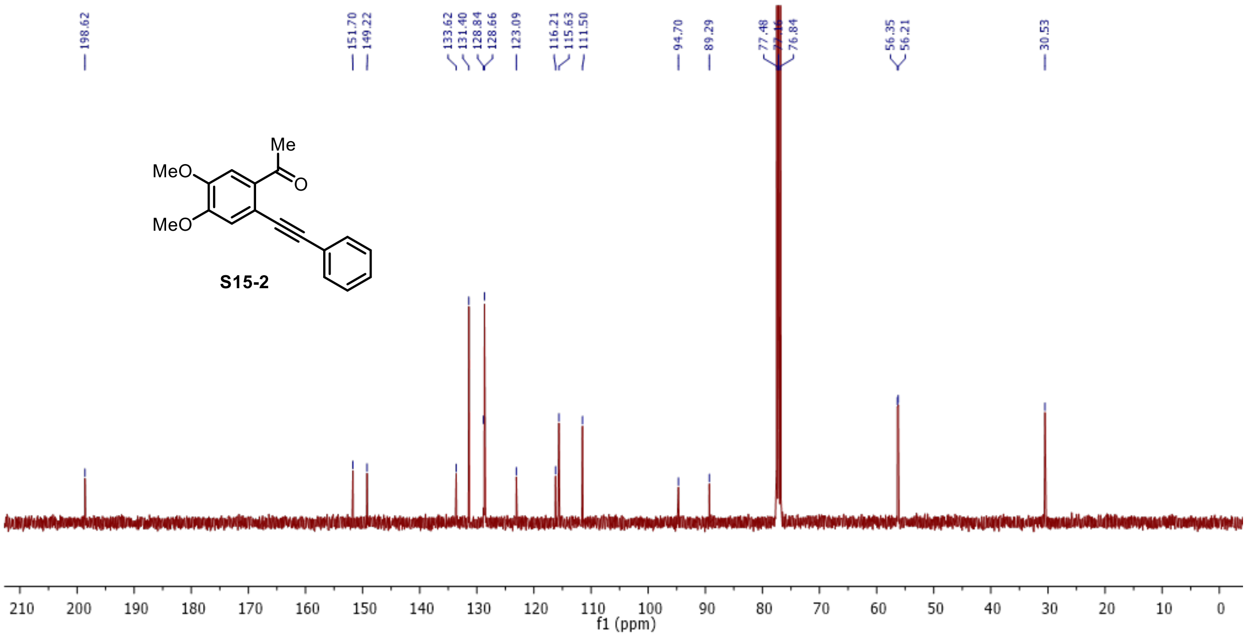

1-(4,5-Dimethoxy-2-(phenylethynyl)phenyl)ethan-1-one oxime (S15)

^{1}H NMR (400 MHz, $CDCl_3$)

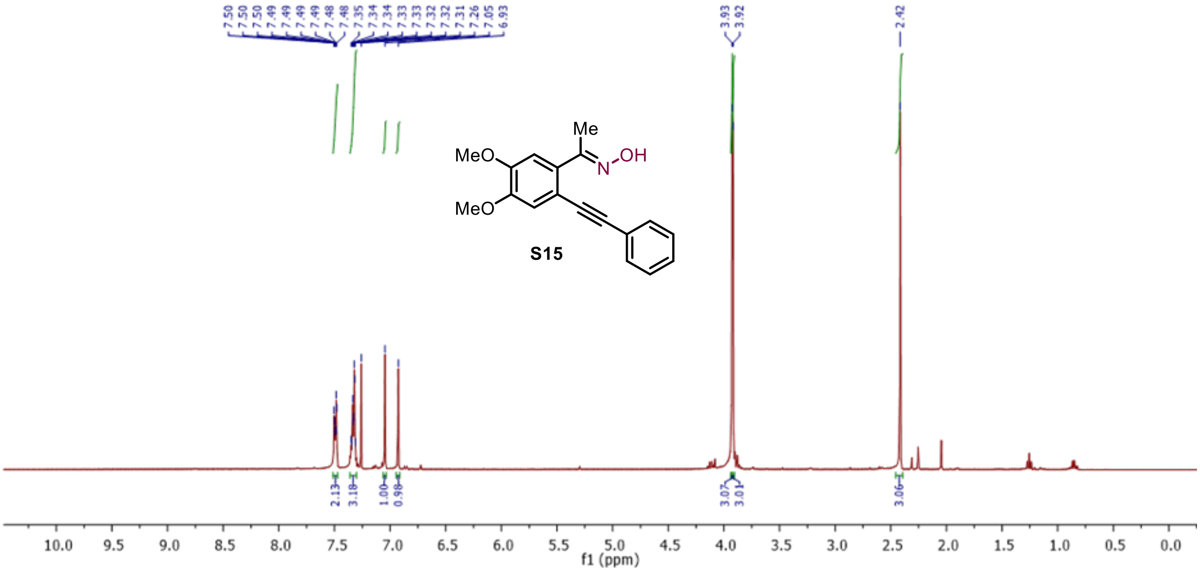

^{13}C NMR (101 MHz, $CDCl_3$)

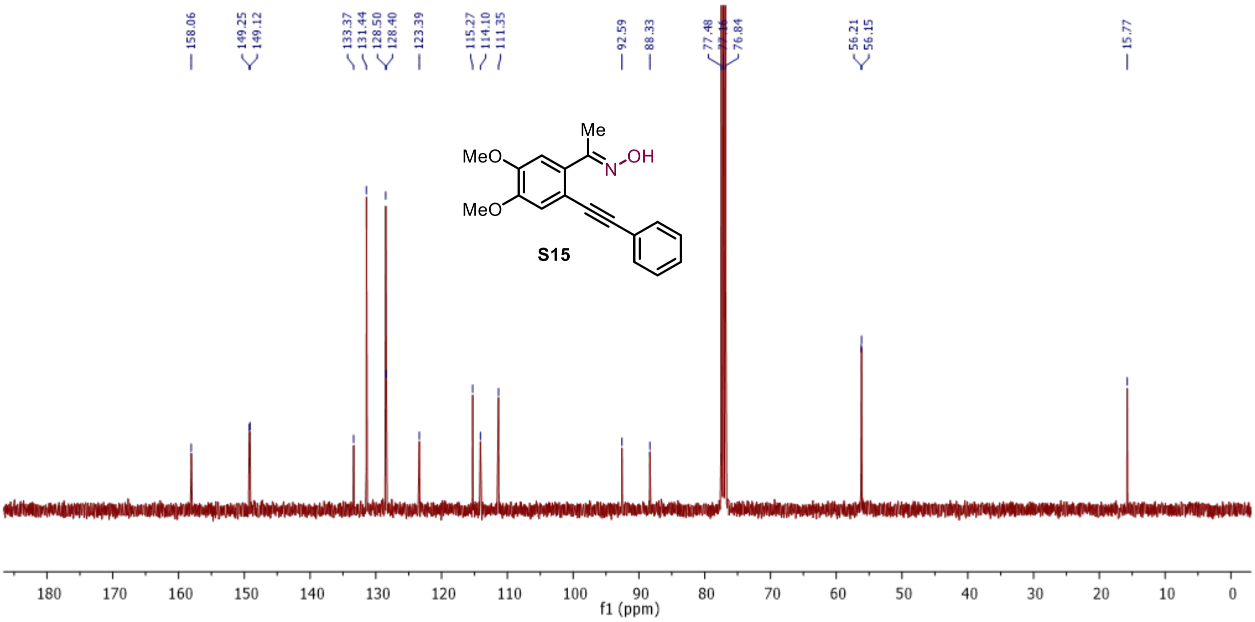

4-Methyl-N'-(1-(2-(phenylethynyl)phenyl)ethylidene)benzenesulfonohydrazide (S16)

(mixture of two non-isolable two stereoisomers detected)

^{1}H NMR (400 MHz, $CDCl_3$)

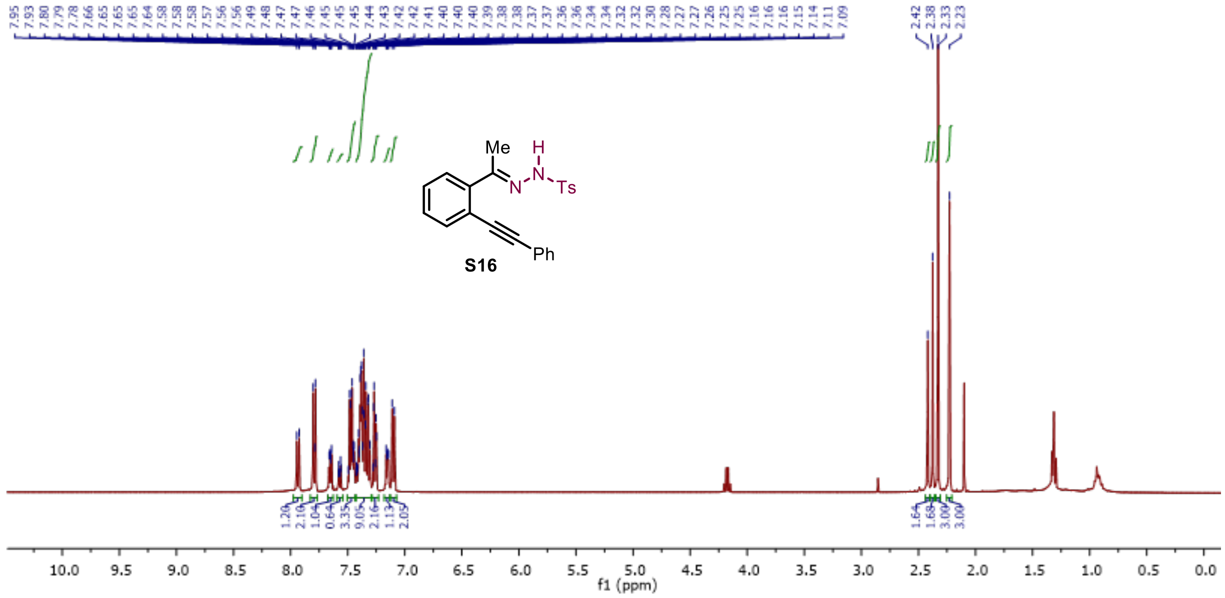

^{13}C NMR (101 MHz, $CDCl_3$

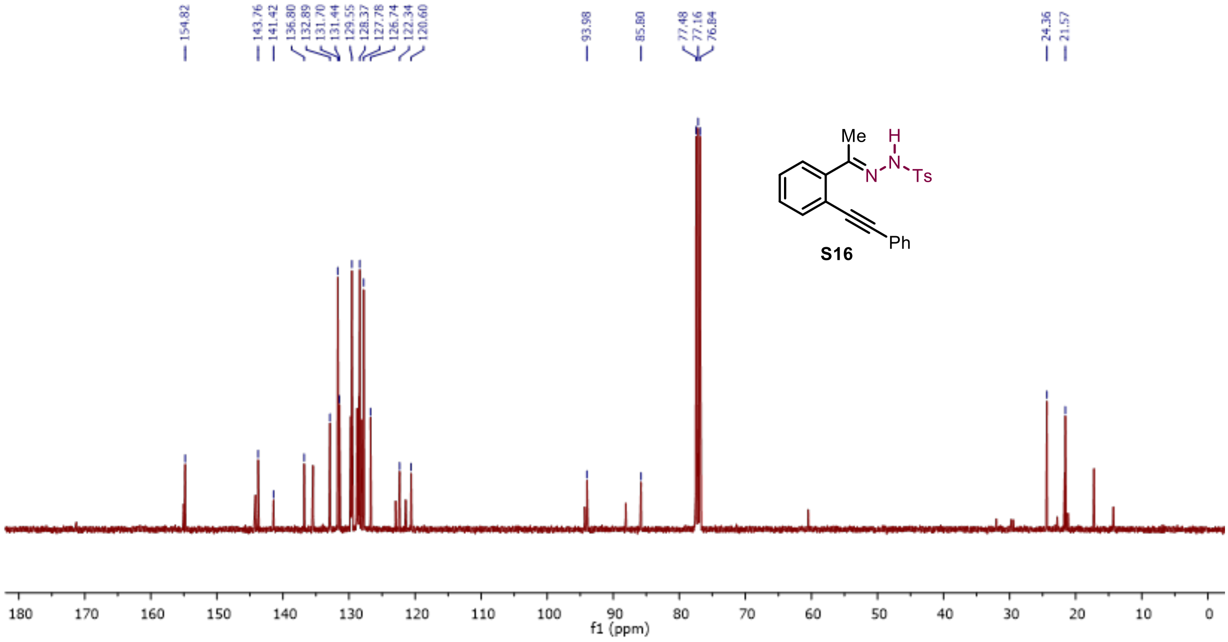

N'-(1-(2-((4-Methoxyphenyl)ethynyl)phenyl)ethylidene)-4-methylbenzenesulfonohydrazide (S17)

(mixture of two non-isolable two stereoisomers detected)

^{1}H NMR (400 MHz, $CDCl_3$):

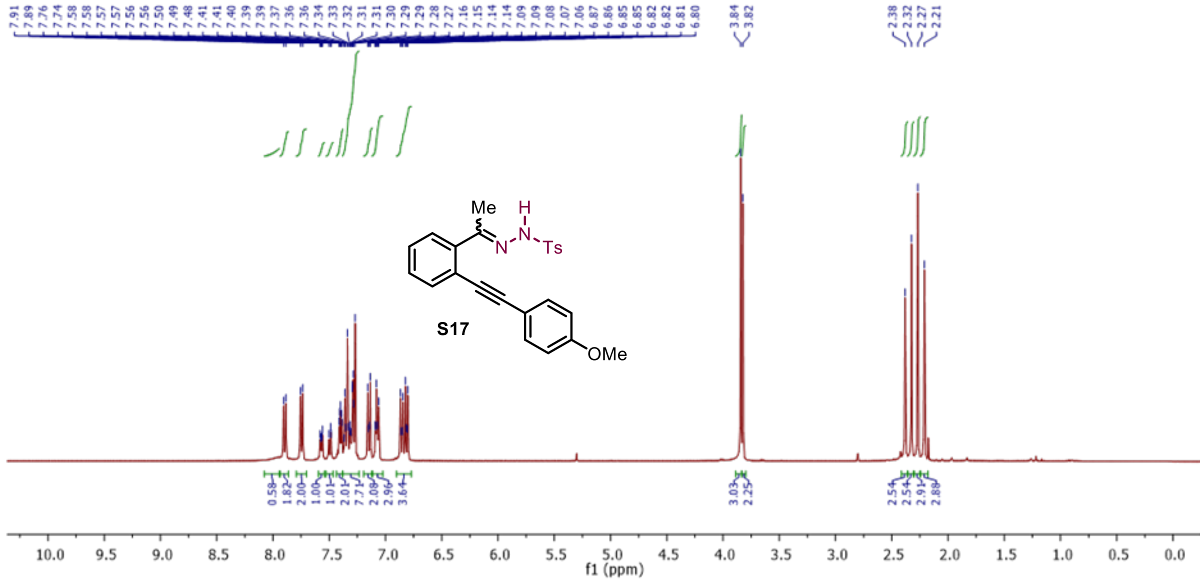

^{13}C NMR (101 MHz, $CDCl_3$)

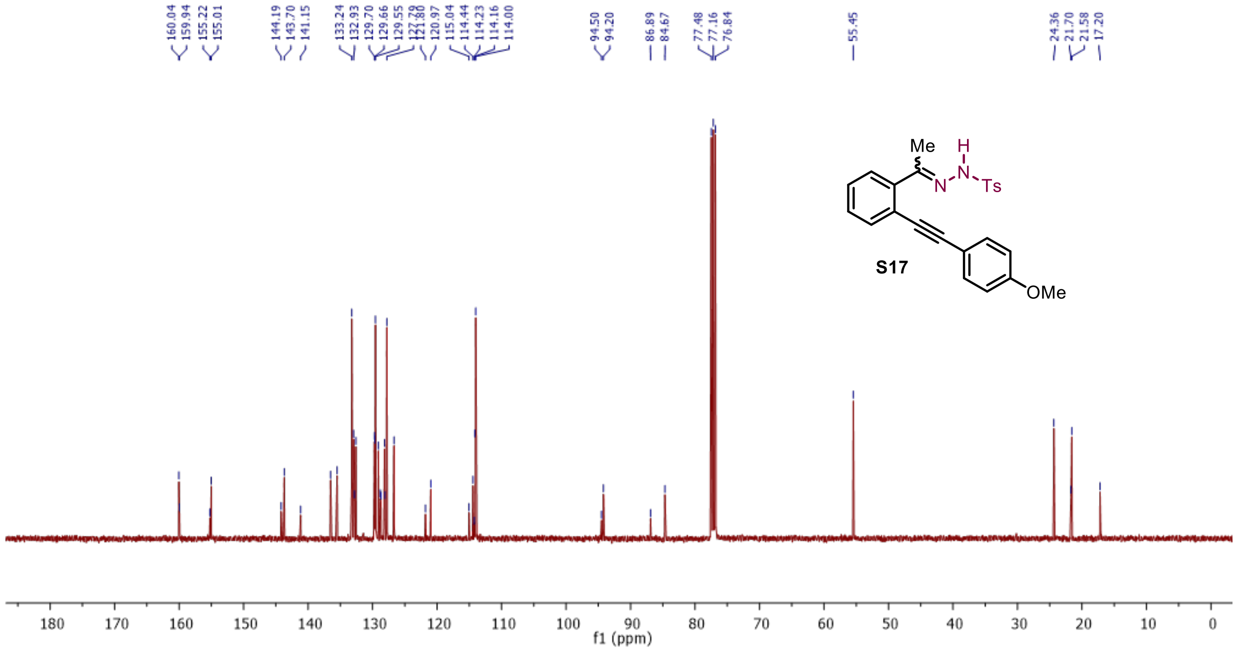

4-Methyl-N'-(2-(phenylethynyl)benzylidene)benzenesulfonohydrazide (S18)

^{1}H NMR (400 MHz, $CDCl_3$)

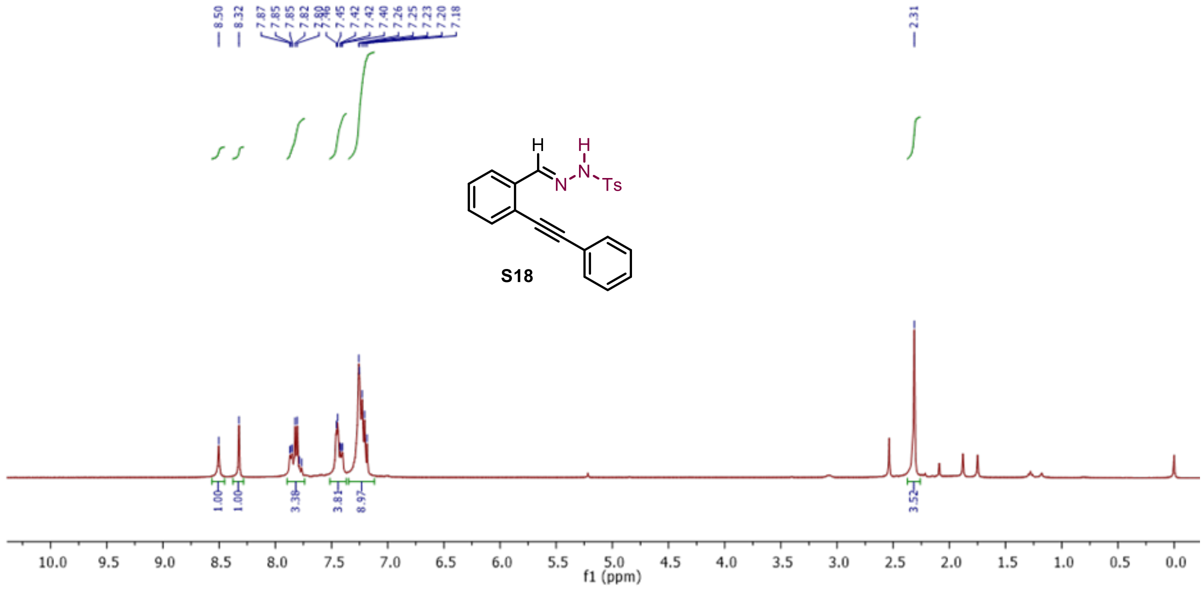

^{13}C NMR (101 MHz, $CDCl_3$)

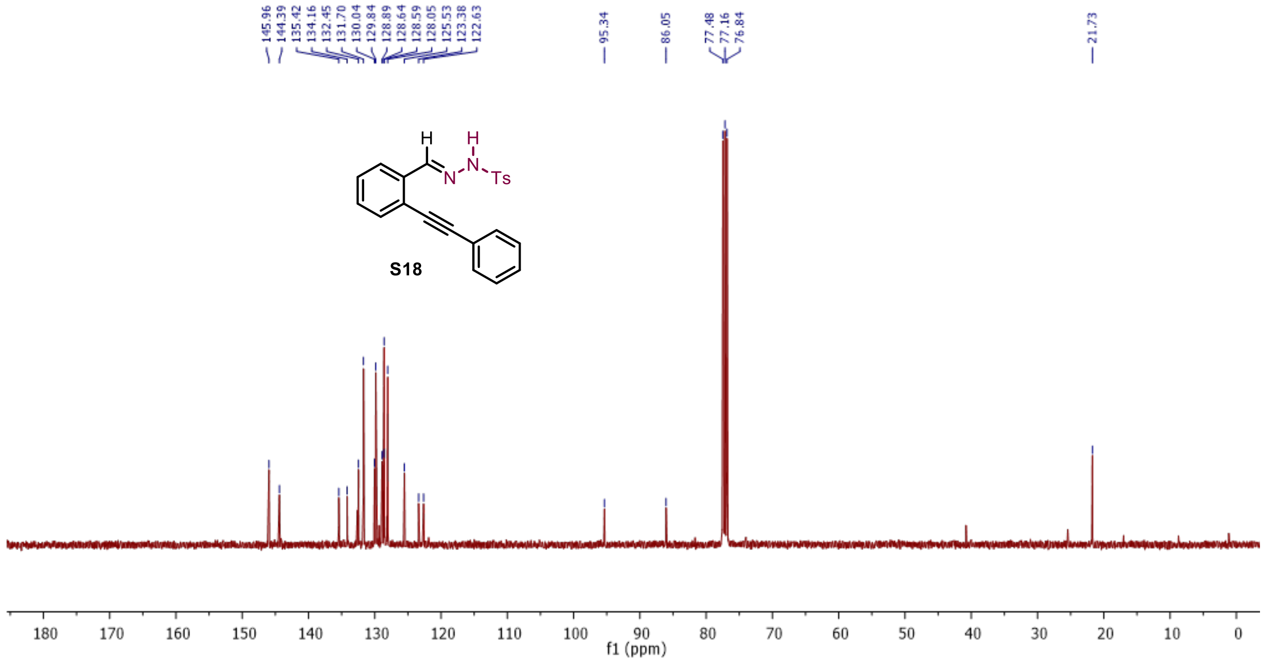

N'-(2-((4-Methoxyphenyl)ethynyl)benzylidene)-4-methylbenzenesulfonohydrazide (S19)

^{1}H NMR (400 MHz, $CDCl_3$)

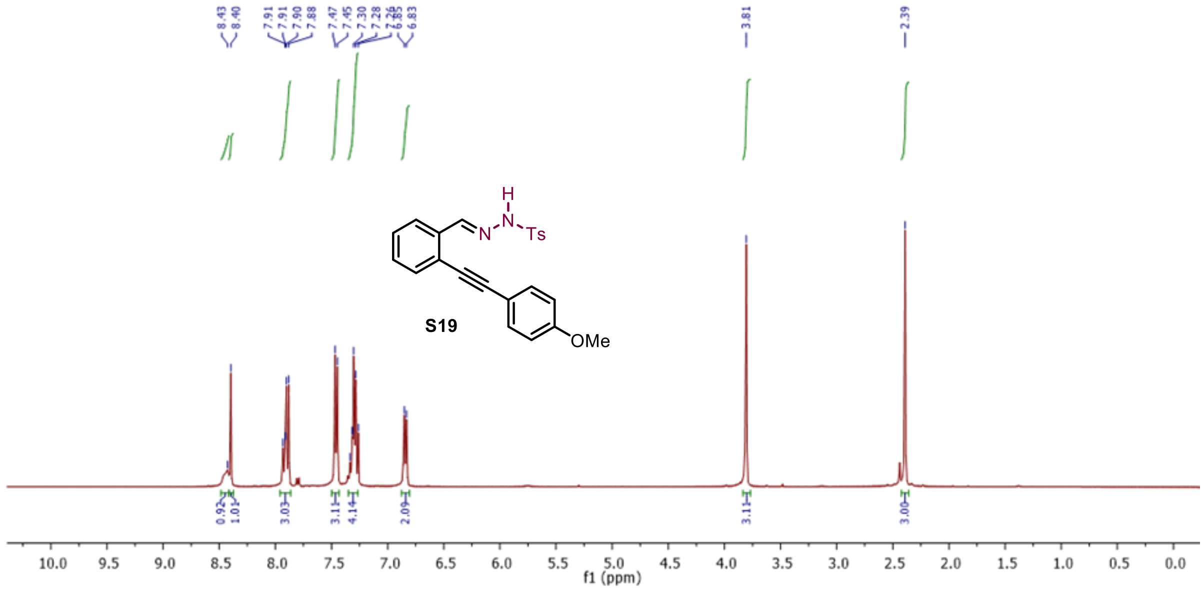

^{13}C NMR (101 MHz, $CDCl_3$)

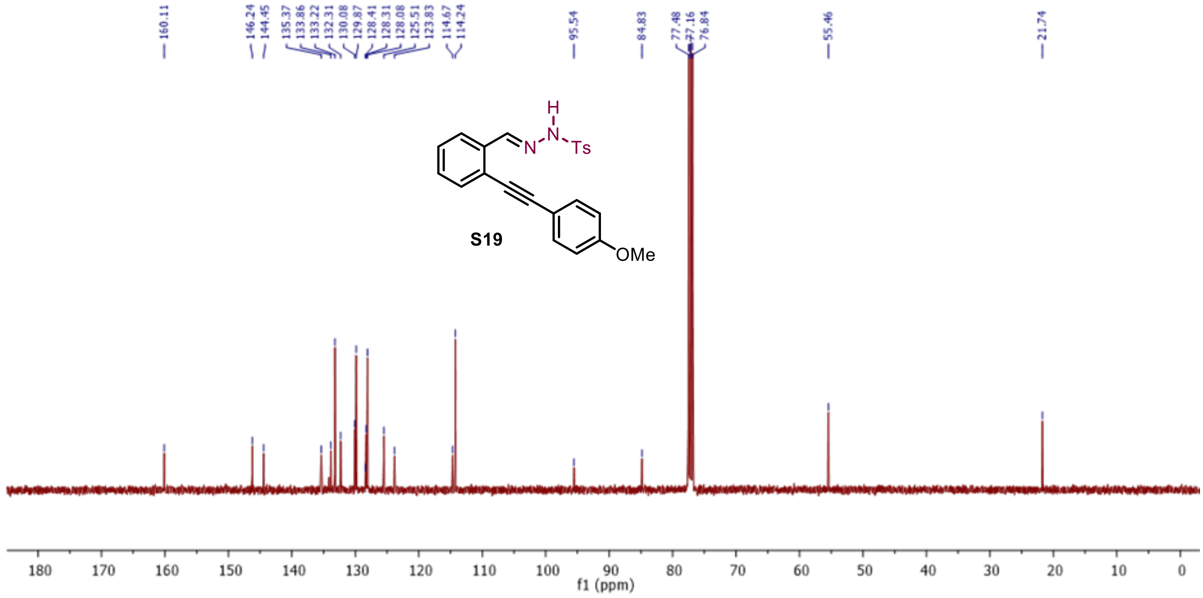

6-phenylhex-5-yn-2-one (S20)

^{1}H NMR (400 MHz, $CDCl_3$)

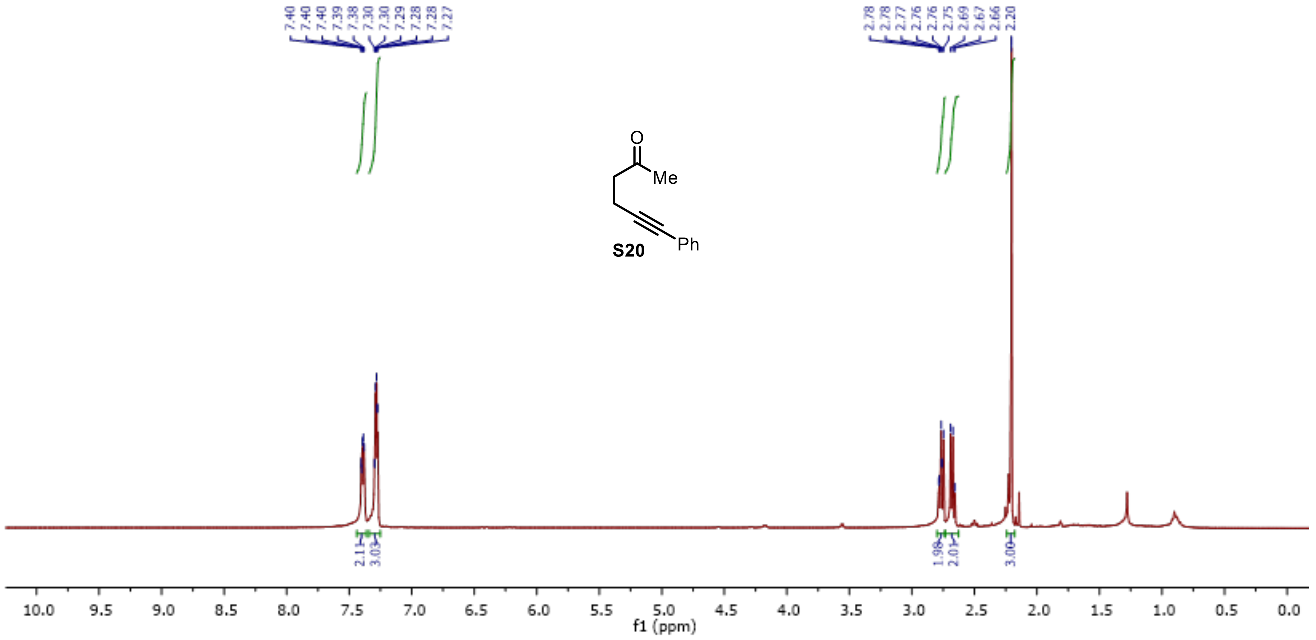

^{13}C NMR (101 MHz, $CDCl_3$)

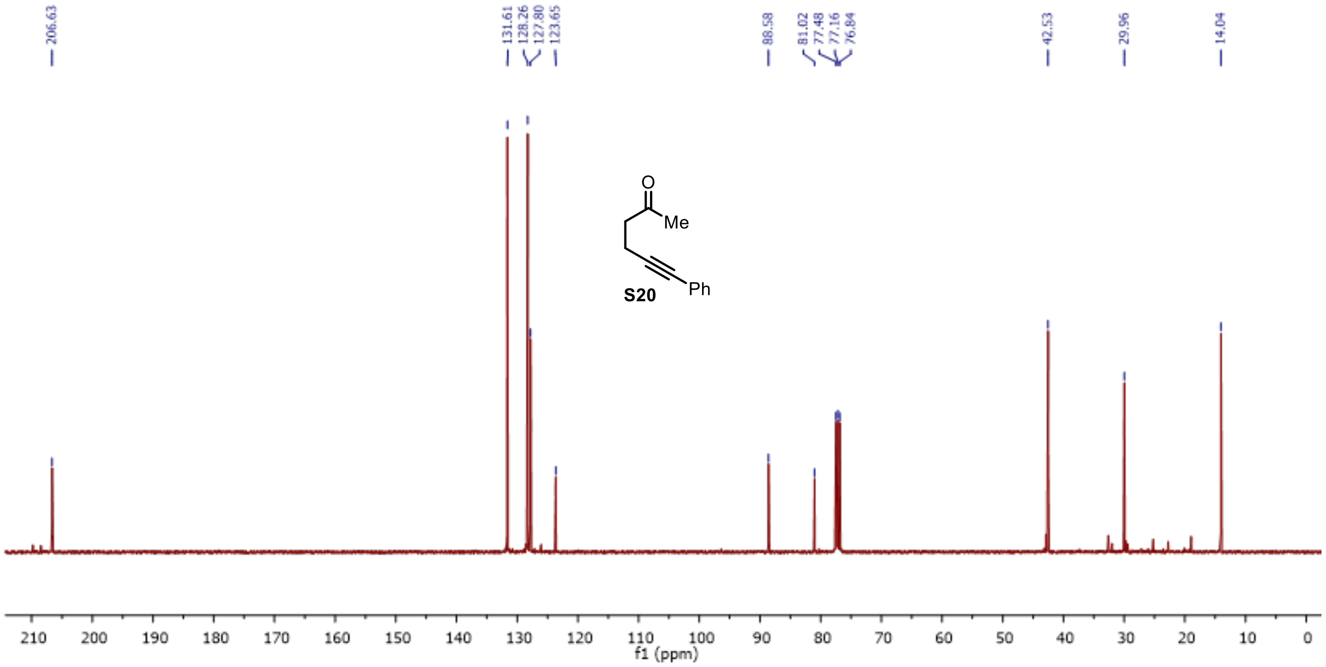

6-phenylhex-5-yn-2-one oxime (S21)

^{1}H NMR (400 MHz, $CDCl_3$)

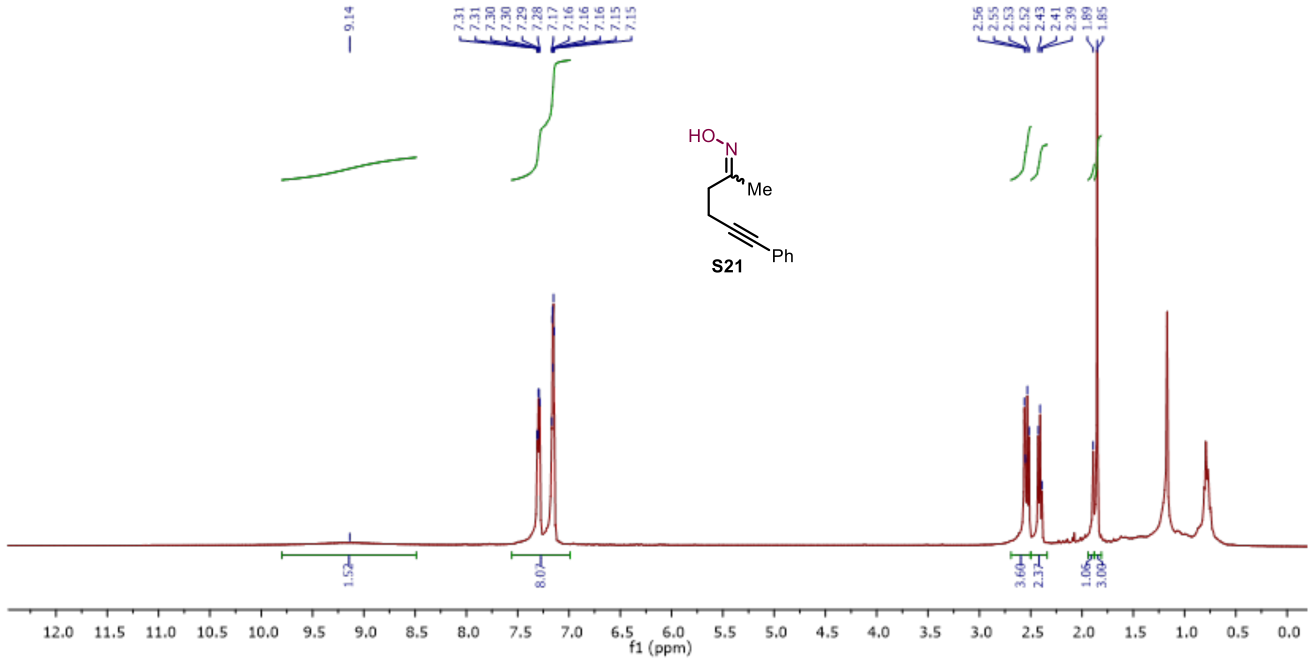

^{13}C NMR (101 MHz, $CDCl_3$)

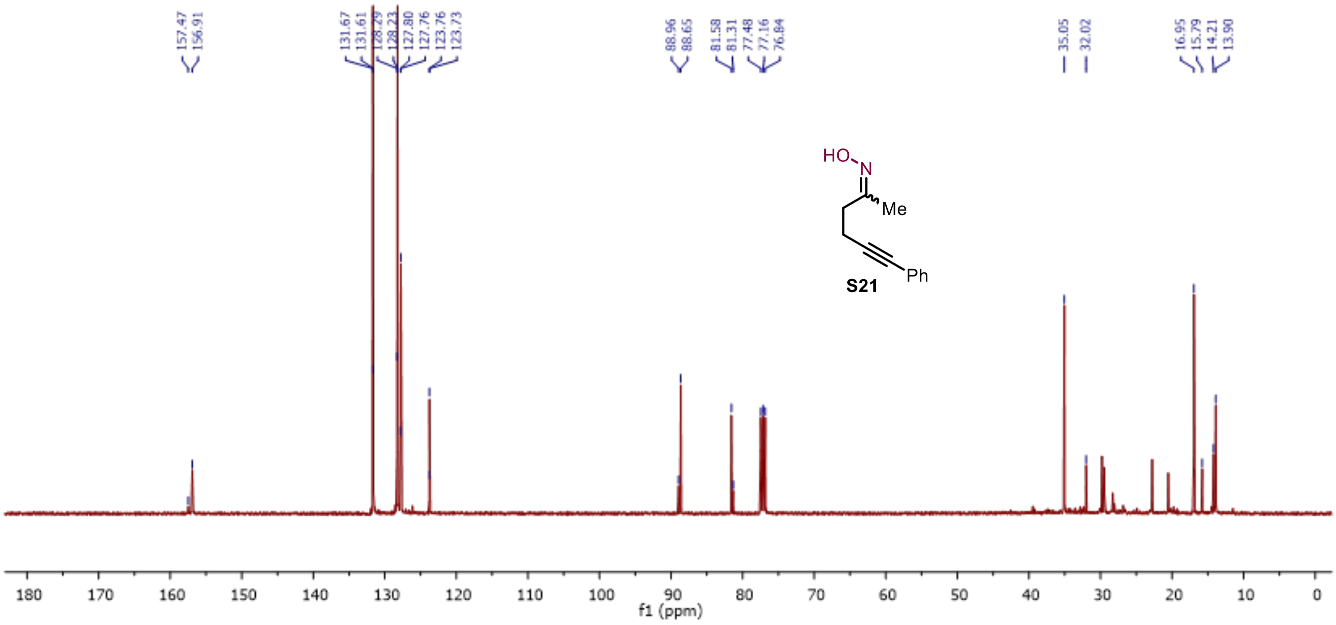

1-(2-(phenylethynyl)pyridin-3-yl)ethan-1-one oxime (S25)

^{1}H NMR (400 MHz, $CDCl_3$)

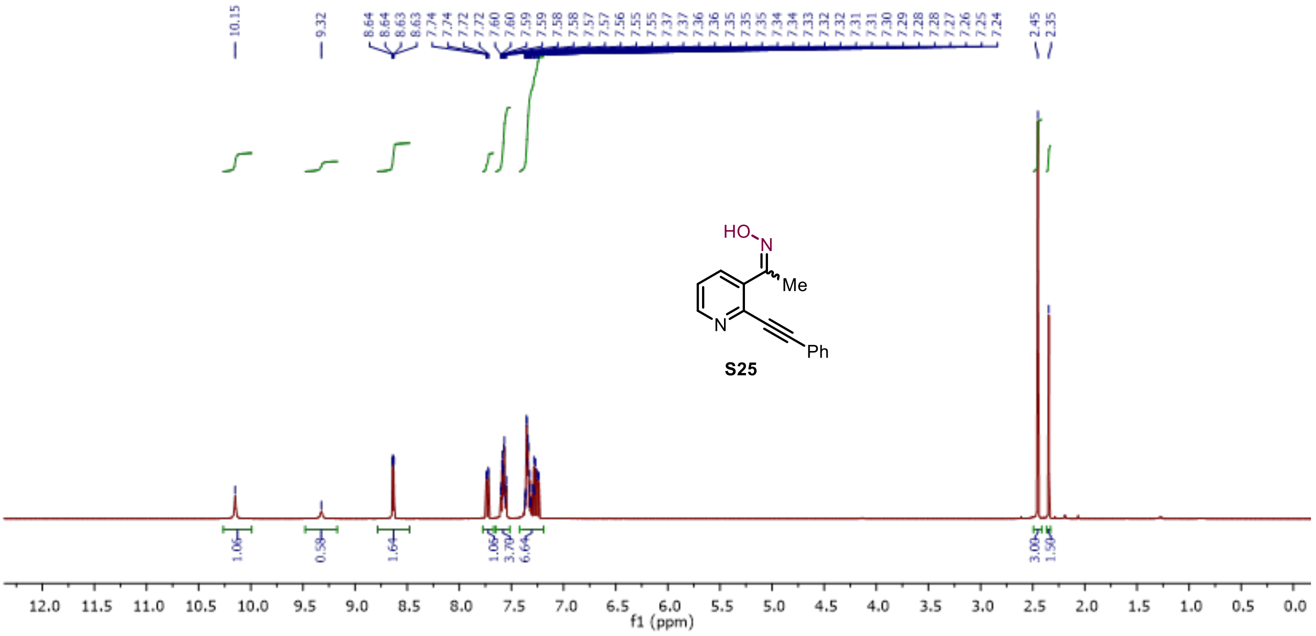

^{13}C NMR (101 MHz, $CDCl_3$)

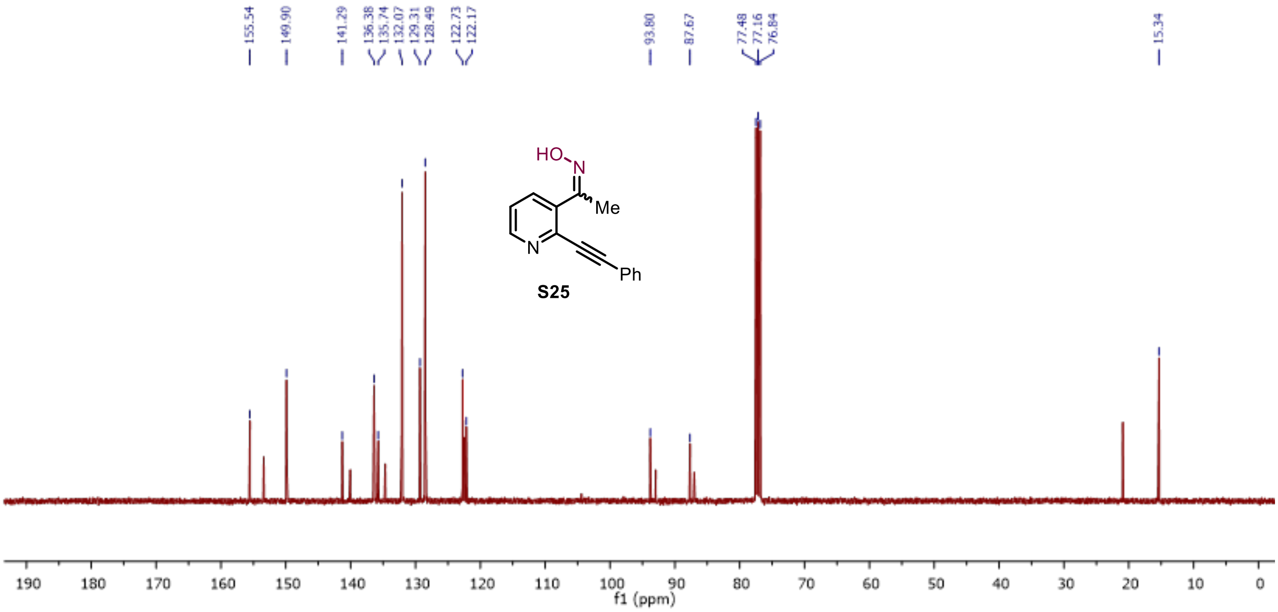

4-Methyl-N'-((E)-2-((E)-styryl)benzylidene)benzenesulfonohydrazide (34a)

^1H NMR (400 MHz, $CDCl_3$)

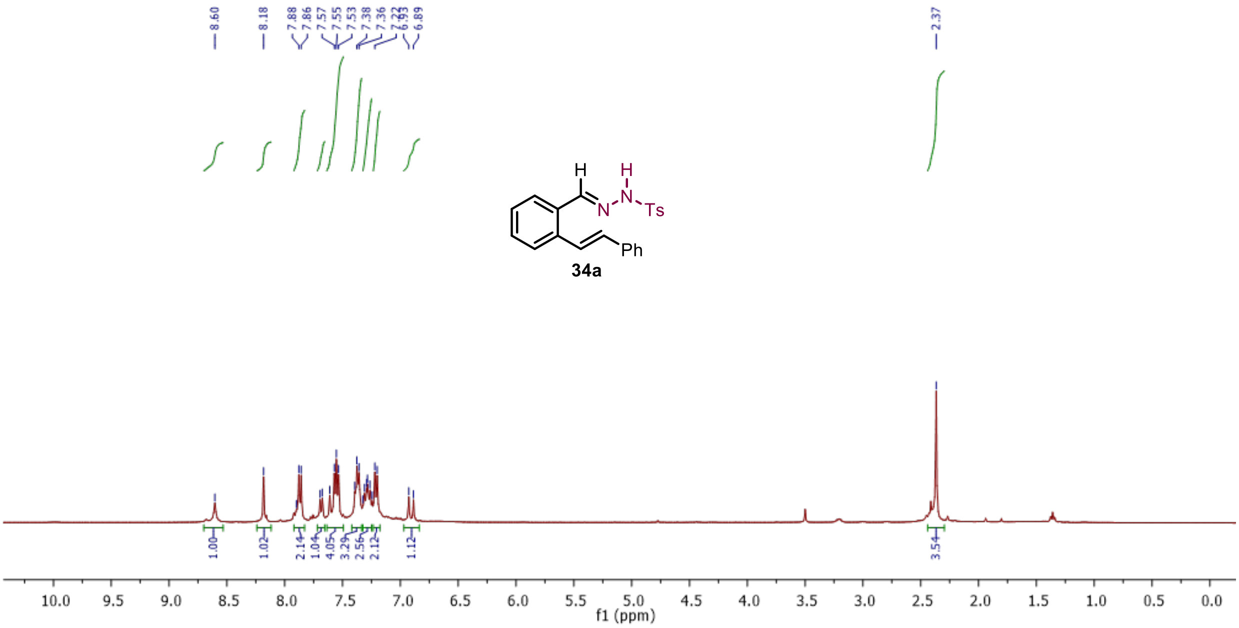

^{13}C NMR (101 MHz, $CDCl_3$)

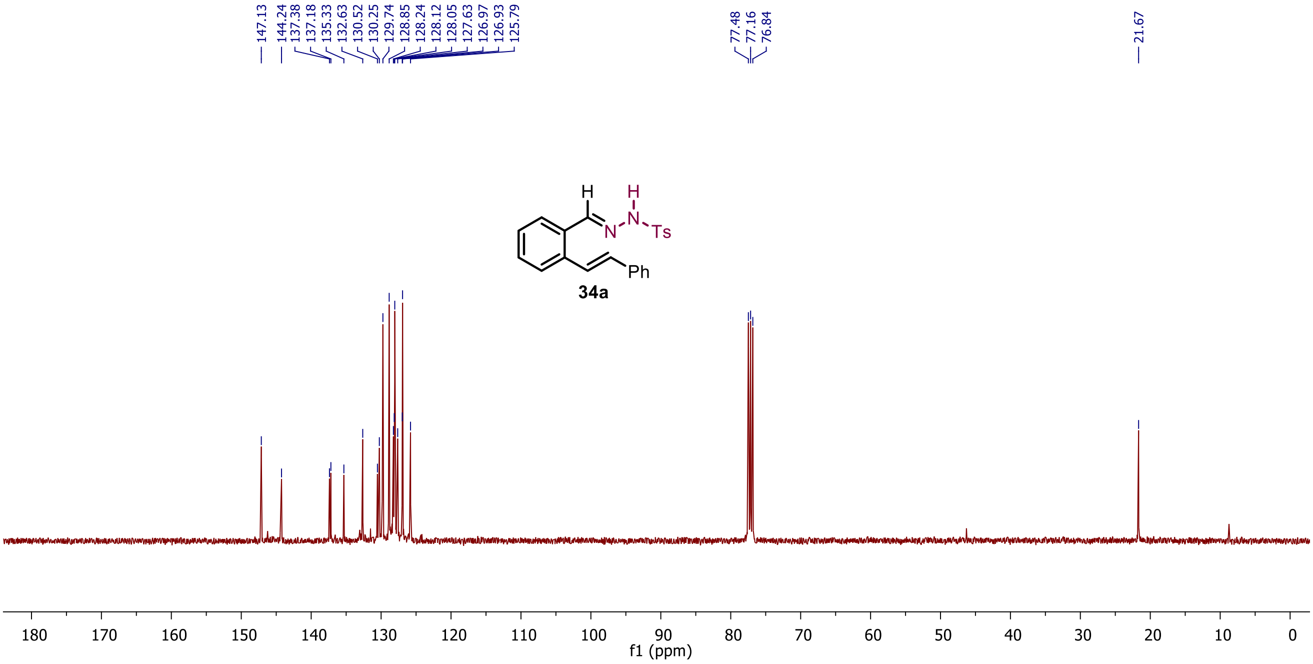

1-(2-(Phenylethynyl)phenyl)ethan-1-one O-methyl oxime (S20)

^{1}H NMR (400 MHz, CDCl$_3$)

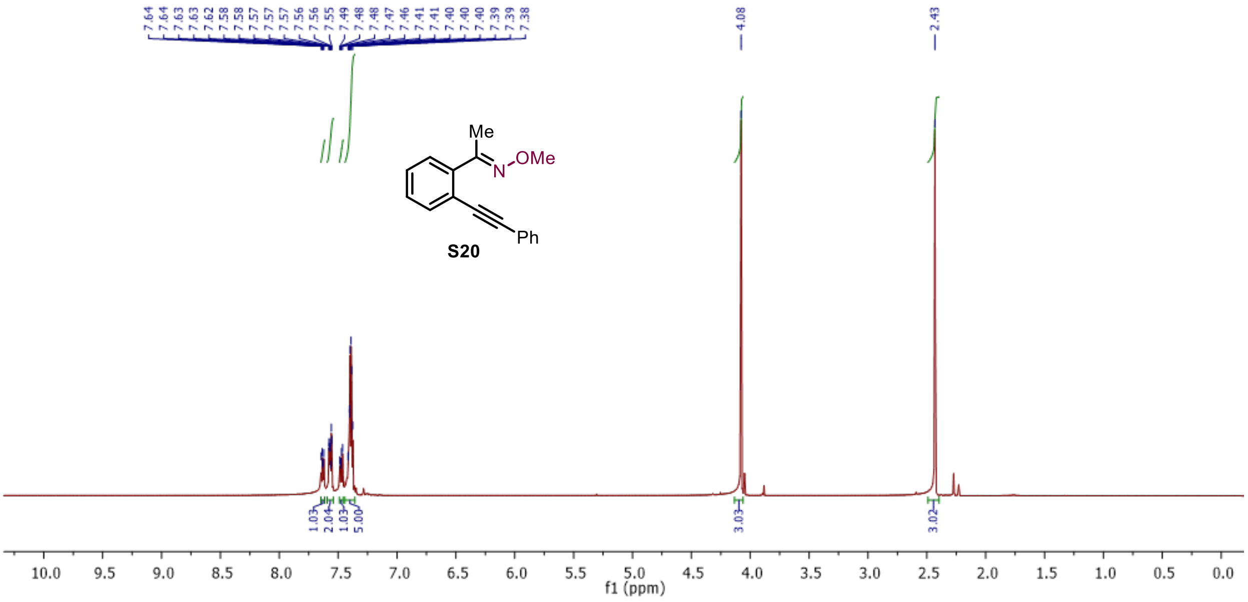

^{13}C NMR (101 MHz, CDCl$_3$)

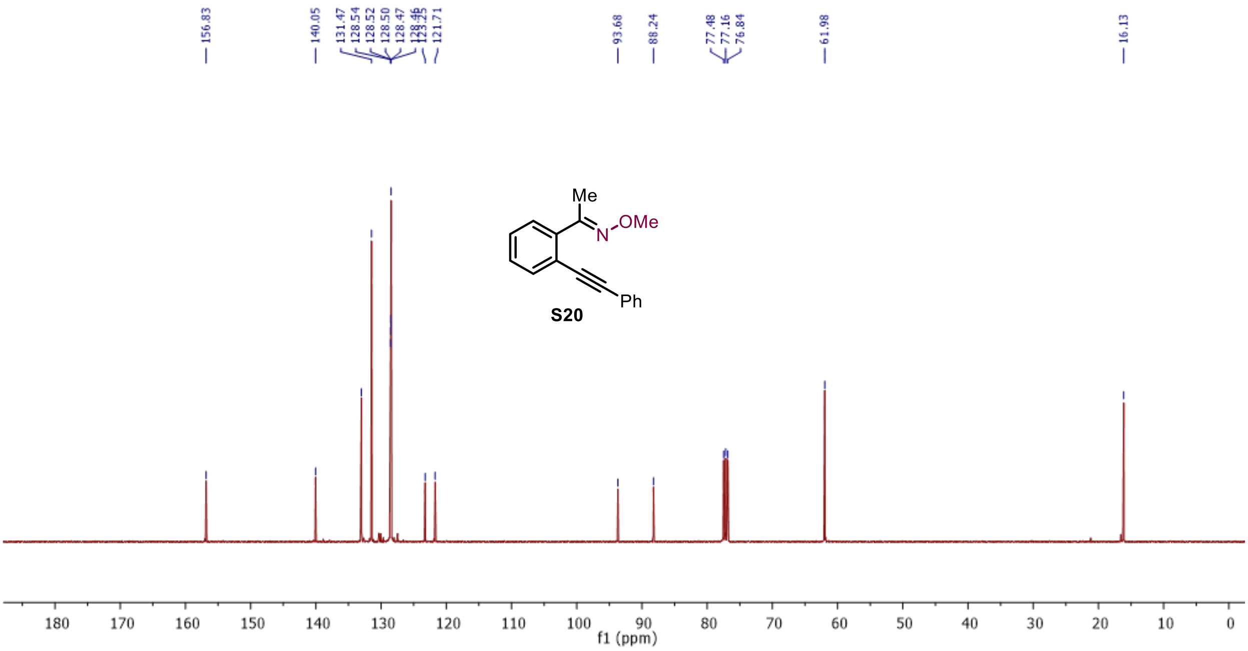

3-(2-(1-(Hydroxyimino)ethyl)phenyl)prop-2-yn-1-yl benzoate (34b)

^{1}H NMR (400 MHz, $CDCl_3$)

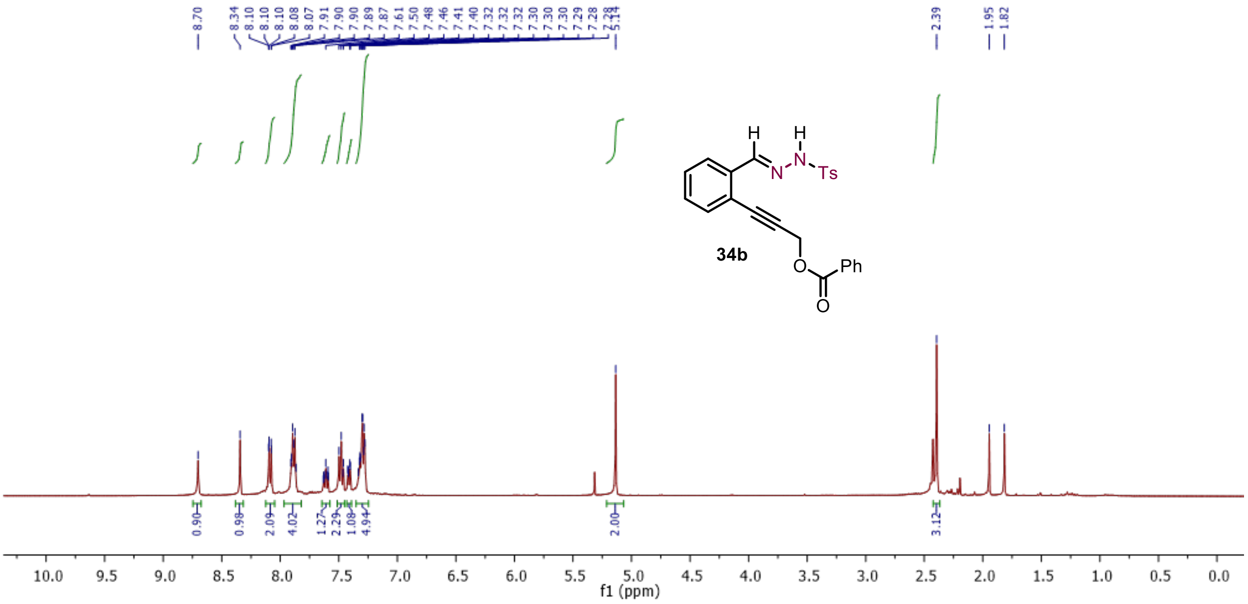

^{13}C NMR (101 MHz, $CDCl_3$)

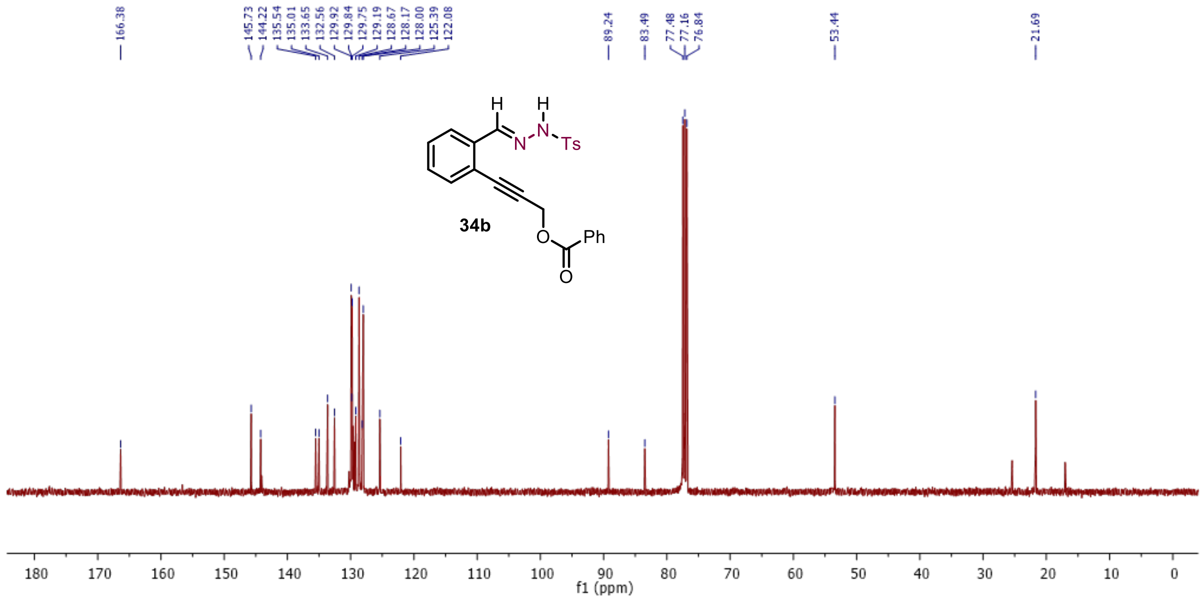

1,2-Di-p-tolyldiselane (Se1)

^{1}H NMR (400 MHz, $CDCl_3$)

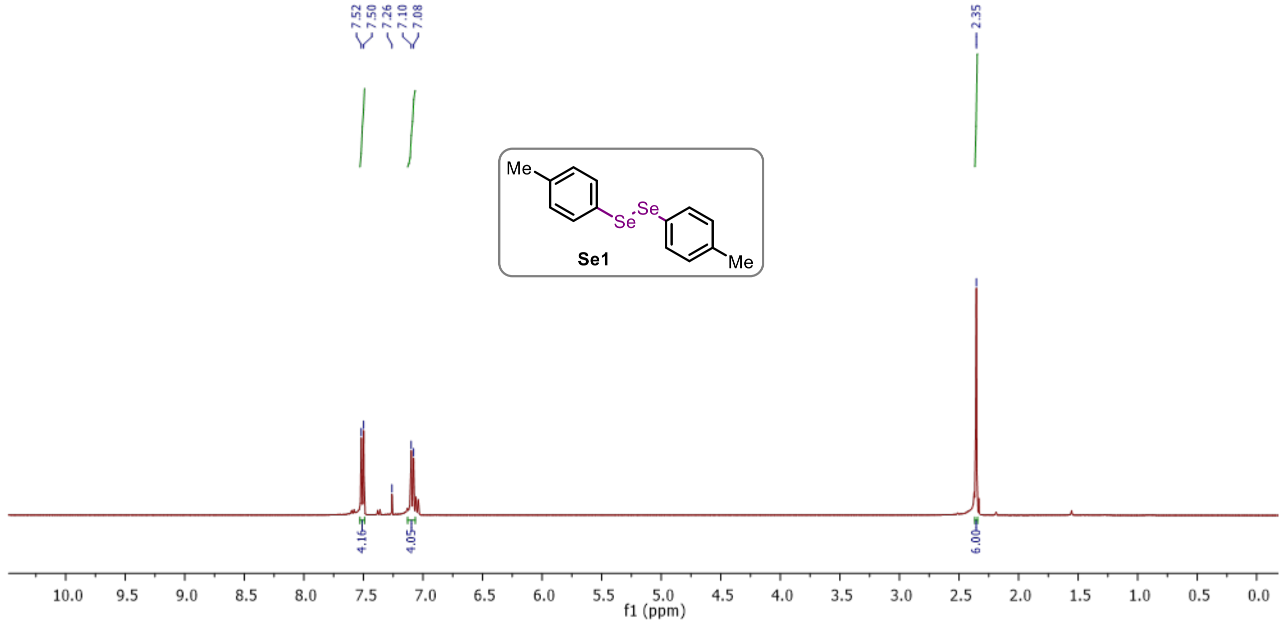

^{13}C NMR (101 MHz, $CDCl_3$)

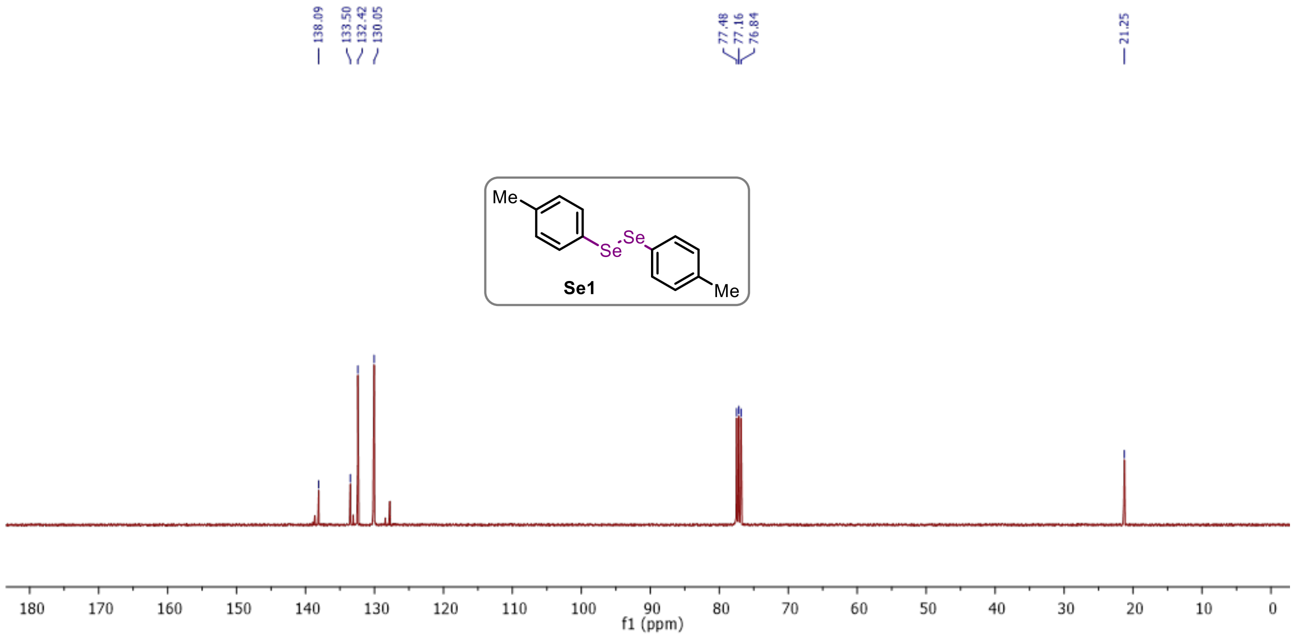

^{77}Se NMR (76 MHz, $CDCl_3$)

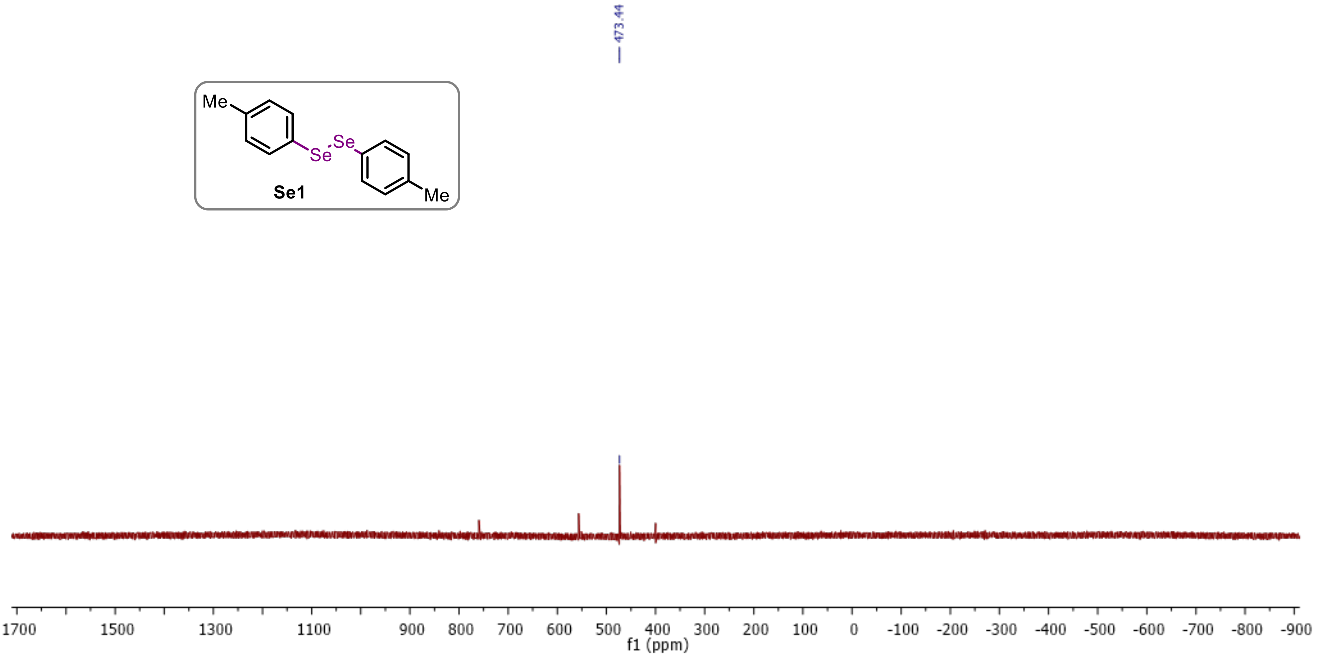

1,2-Bis(4-(trifluoromethyl)phenyl)diselane (Se2)

^{1}H NMR (400 MHz, $CDCl_3$)

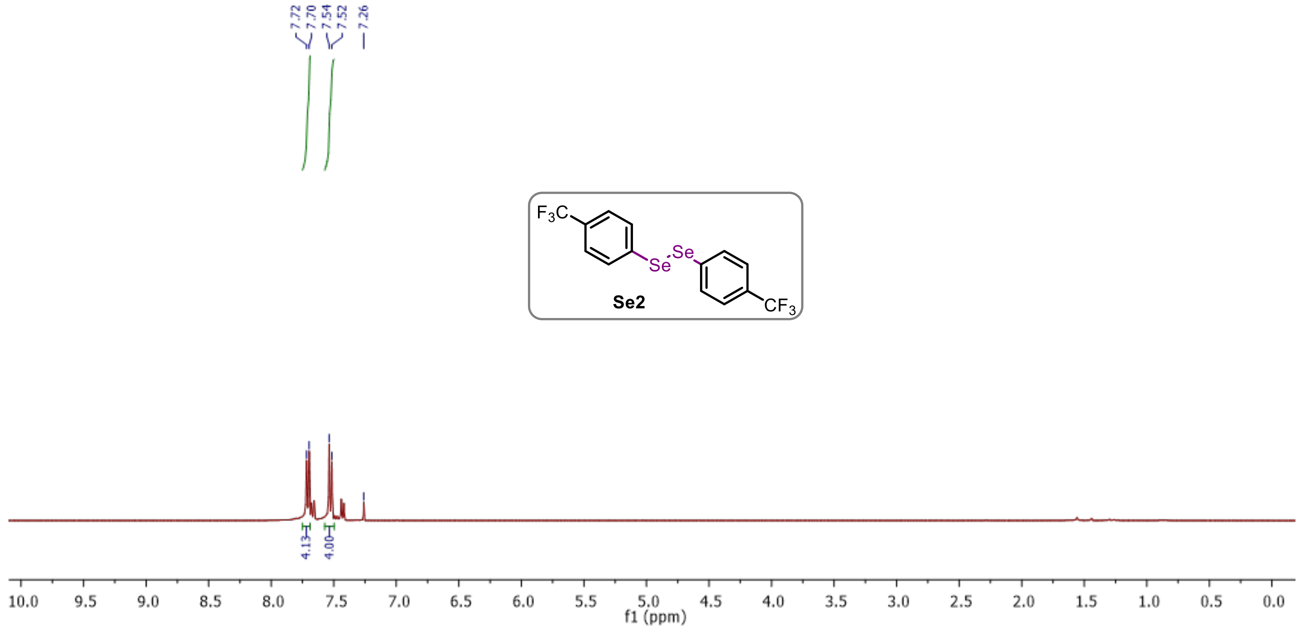

^{13}C NMR (101 MHz, $CDCl_3$)

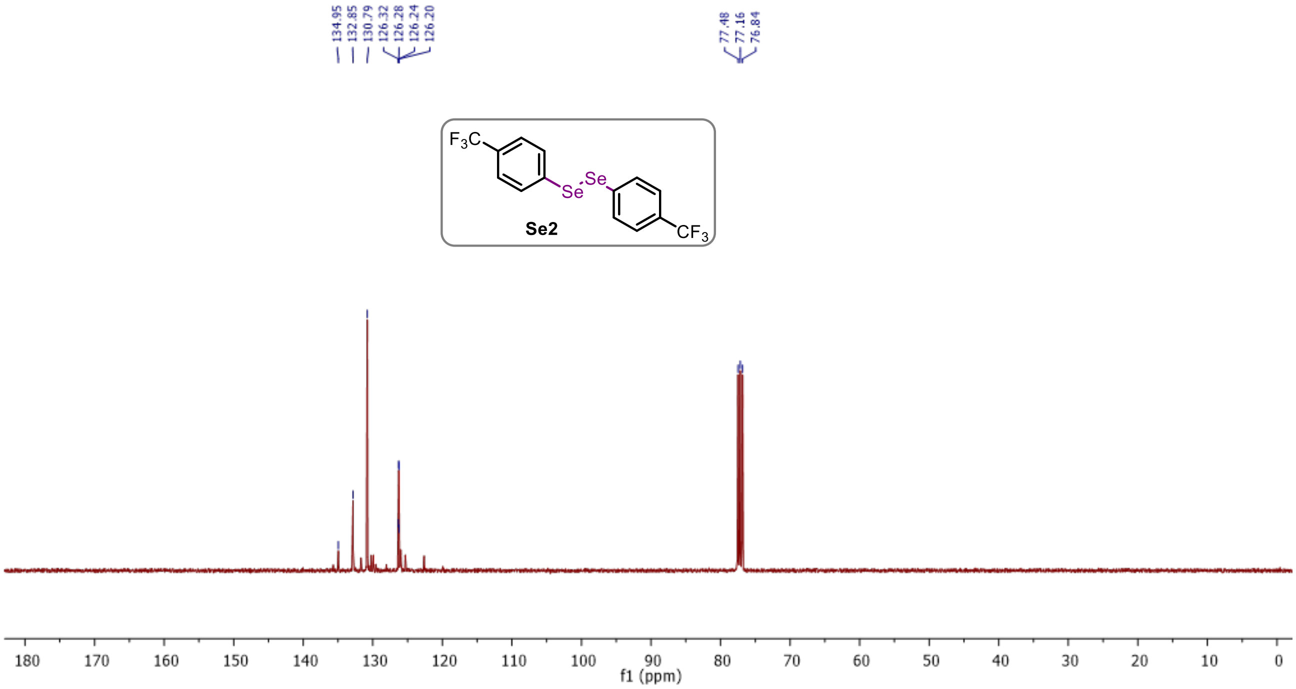

^{19}F NMR (376 MHz, $CDCl_3$)

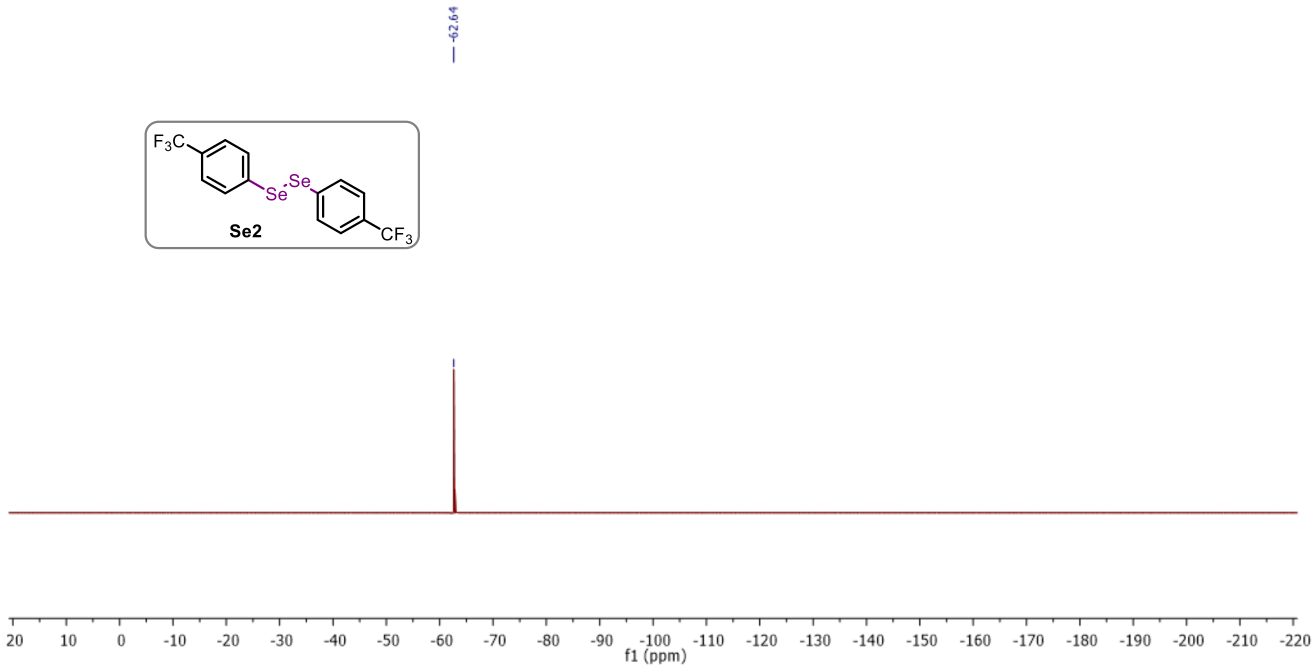

^{77}Se NMR (76 MHz, $CDCl_3$)

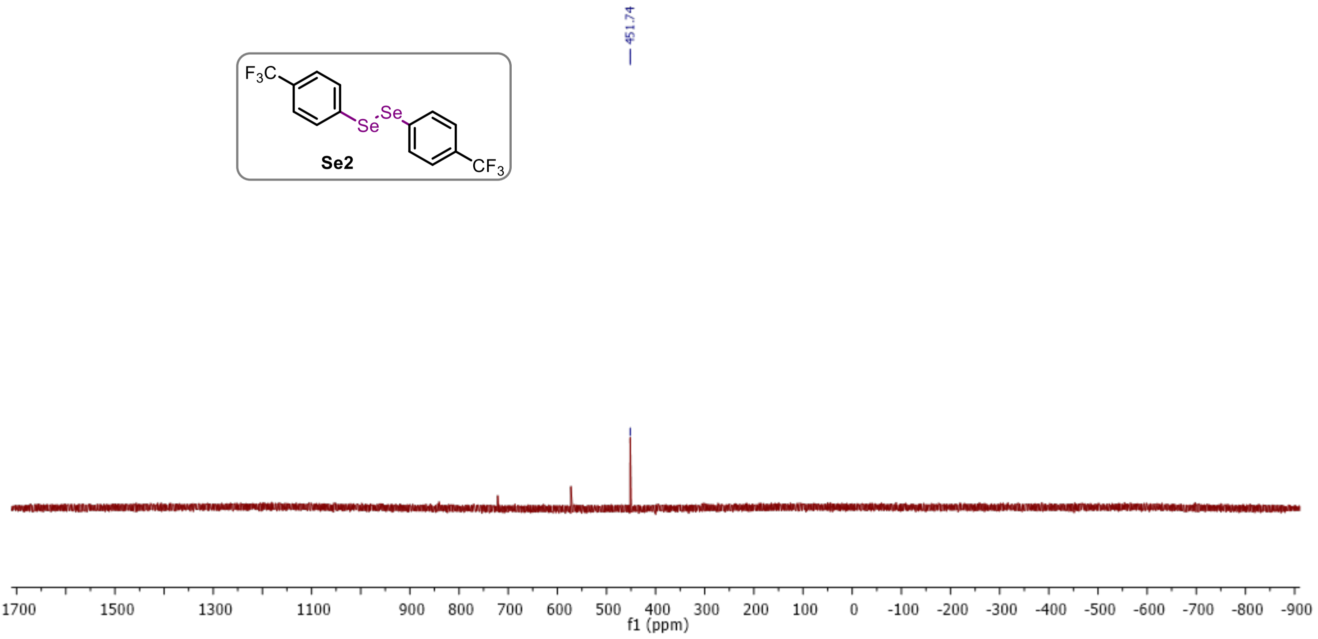

1,2-Bis(3-fluorophenyl)diselane (Se3)

^{1}H NMR (400 MHz, $CDCl_3$)

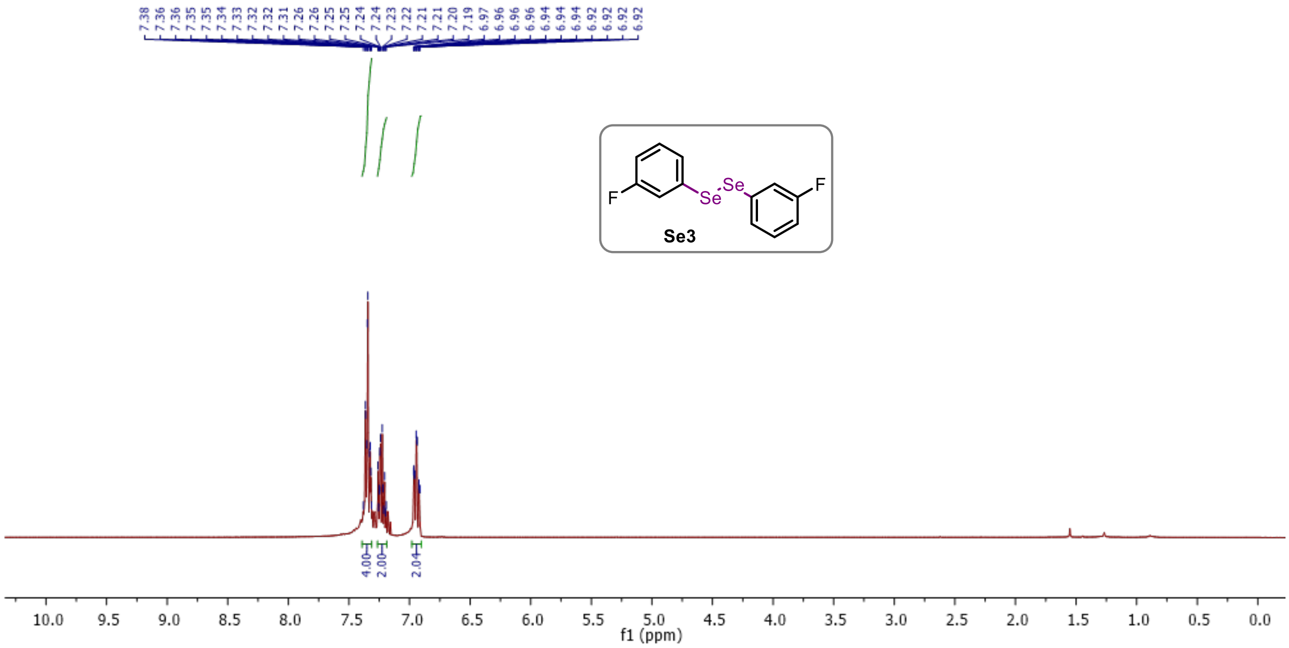

^{13}C NMR (101 MHz, $CDCl_3$)

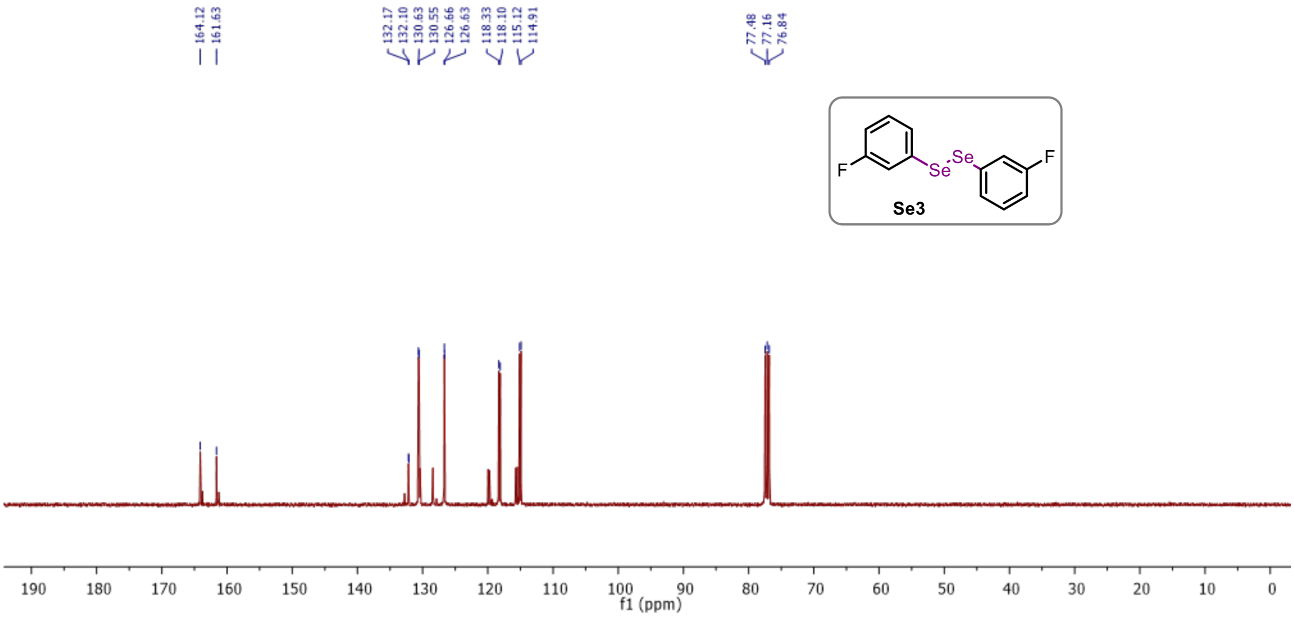

^{19}F NMR (376 MHz, $CDCl_3$)

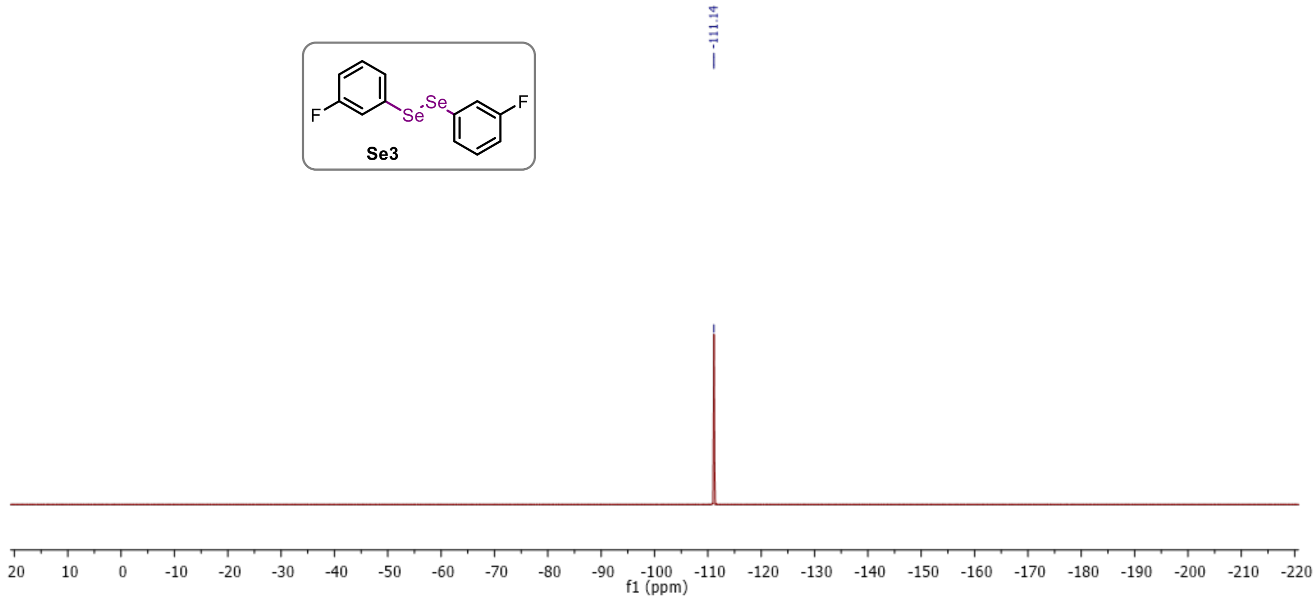

^{77}Se NMR (76 MHz, $CDCl_3$)

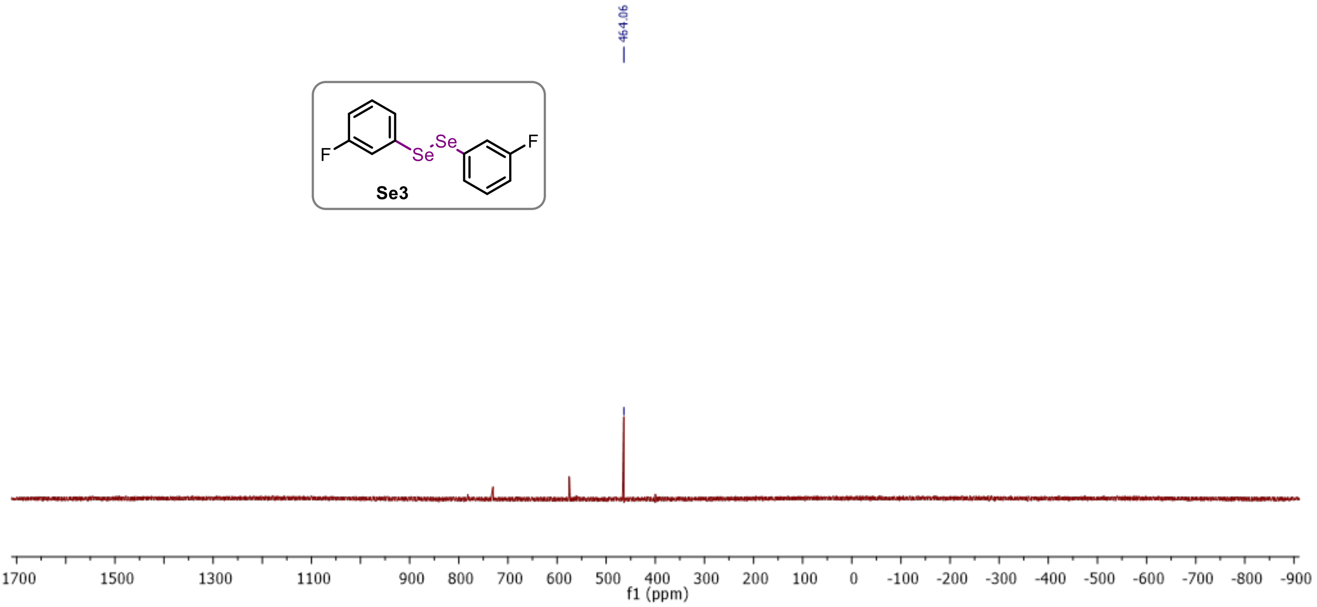

1,2-Di(naphthalen-2-yl)diselane (Se4)

^{1}H NMR (400 MHz, $CDCl_3$)

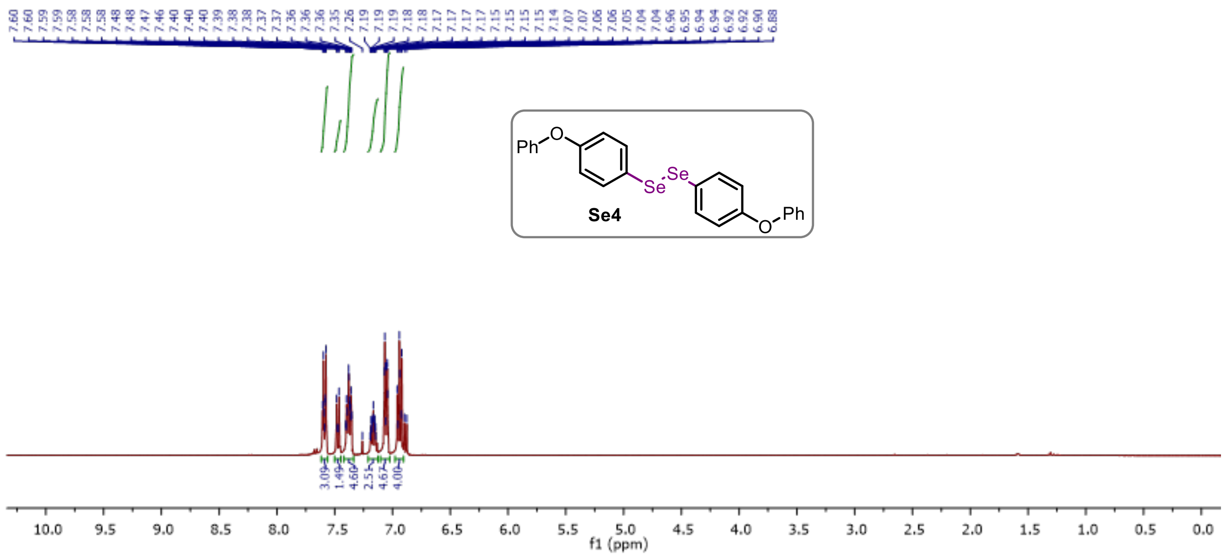

^{13}C NMR (101 MHz, $CDCl_3$)

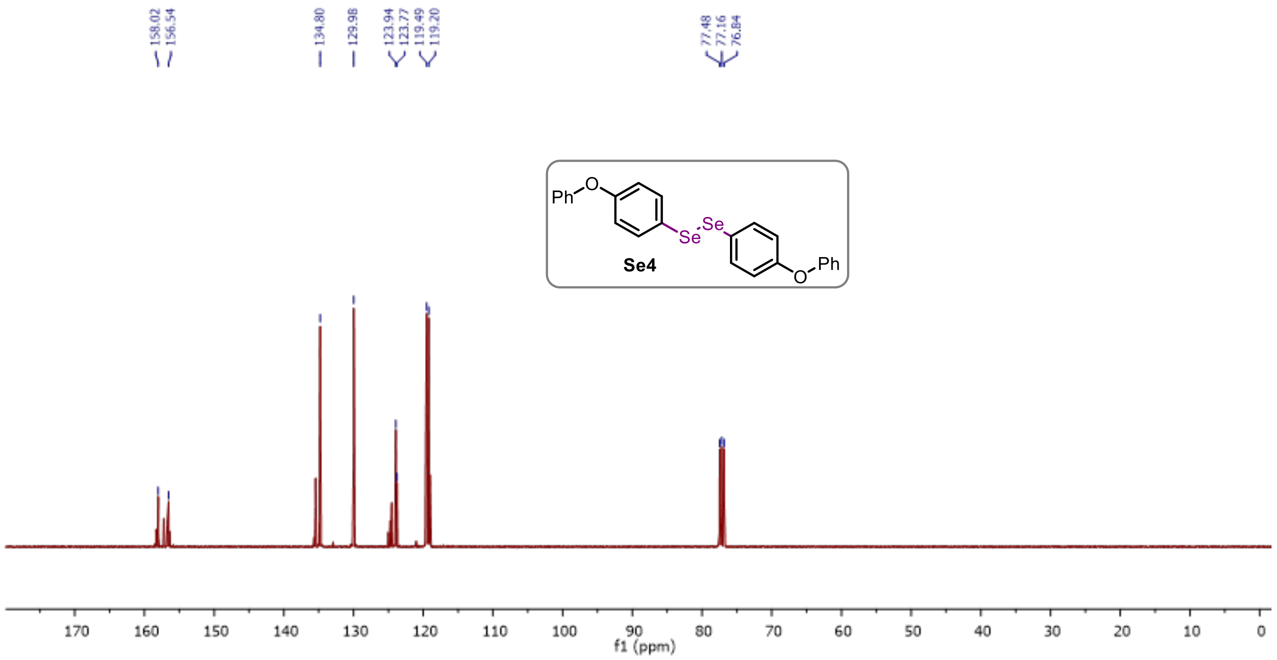

^{77}Se NMR (76 MHz, $CDCl_3$)

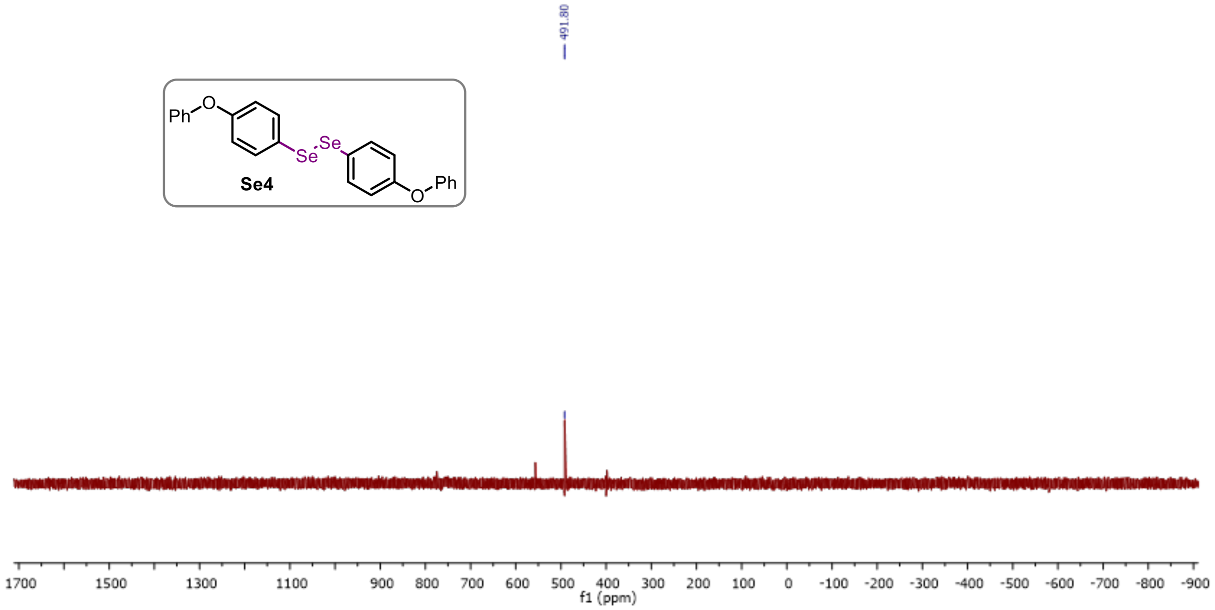

1,2-Di(naphthalen-2-yl)diselane (Se5)

^{1}H NMR (400 MHz, $CDCl_3$)

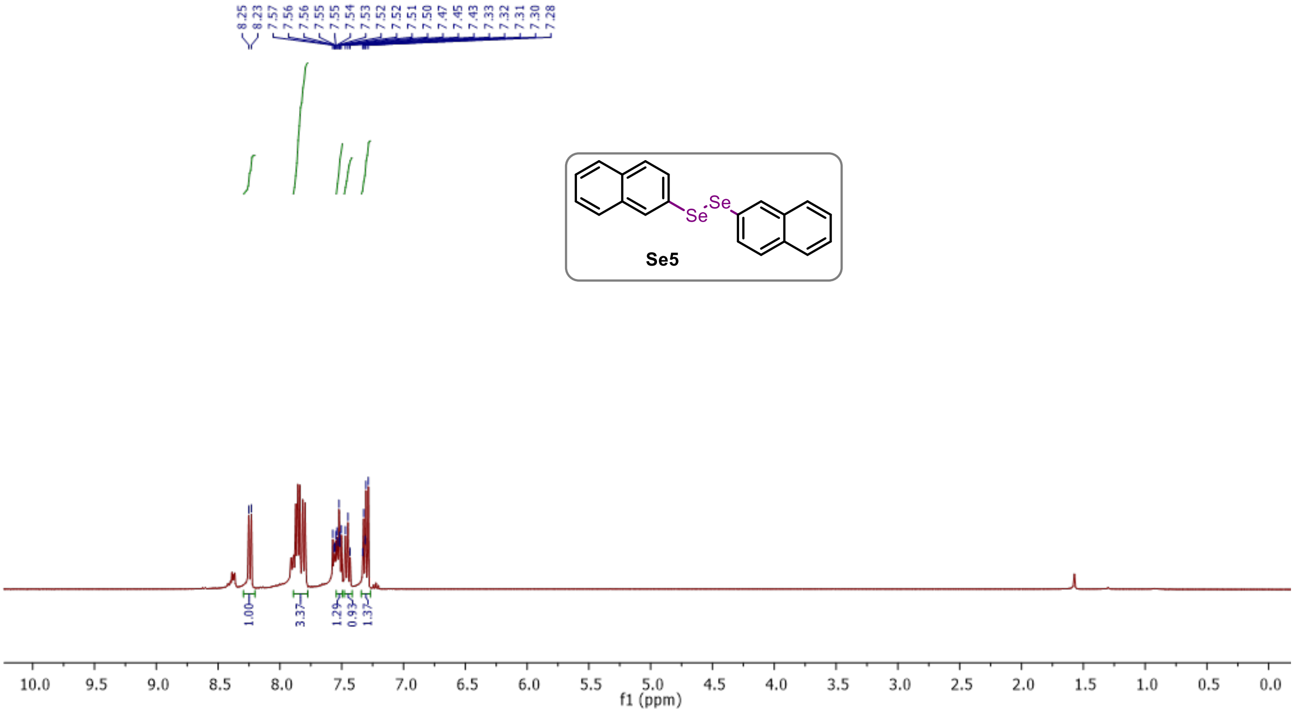

^{13}C NMR (101 MHz, $CDCl_3$)

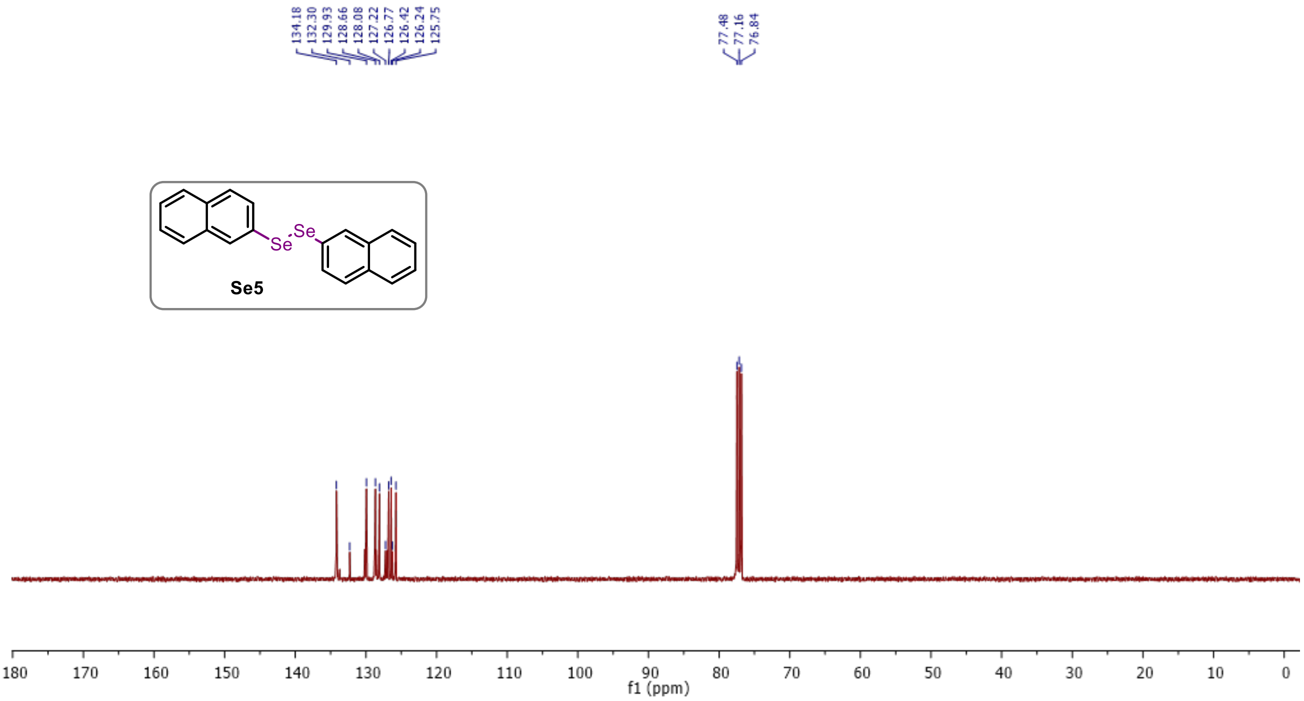

^{77}Se NMR (76 MHz, $CDCl_3$)

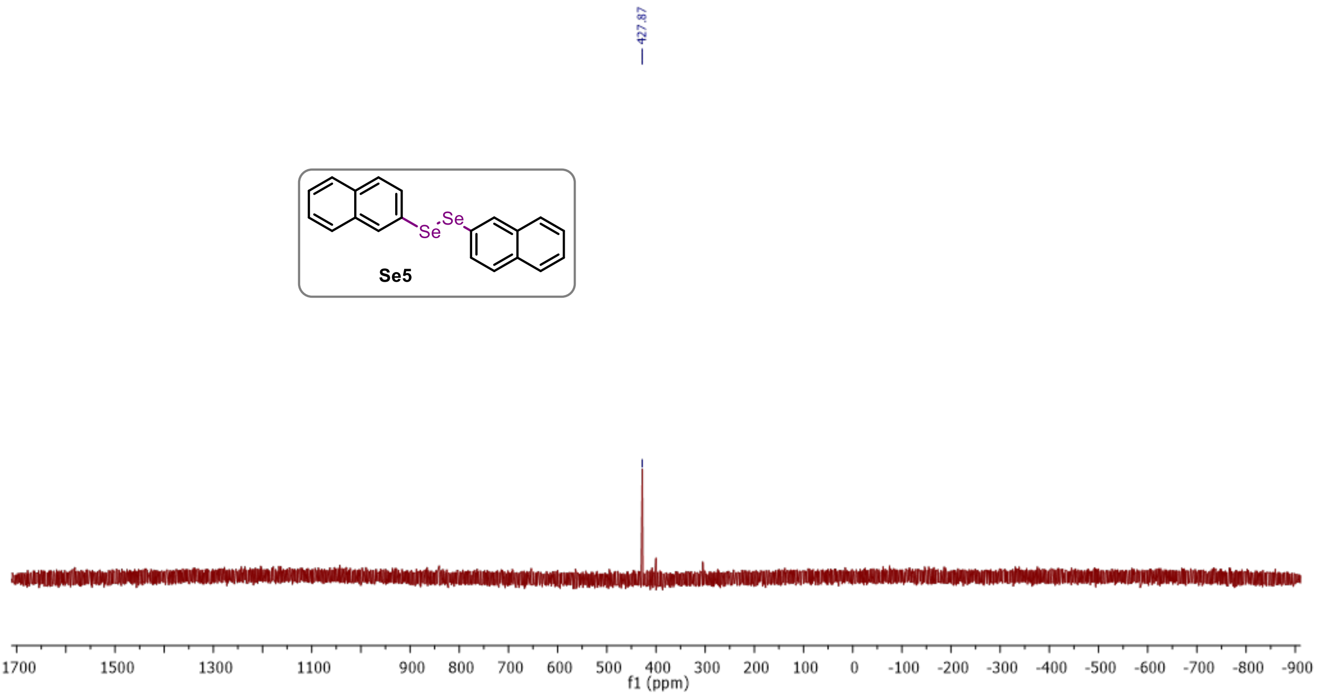

1-Methyl-3-phenyl-4-(phenylselanyl)isoquinoline 2-oxide (3)

^{1}H NMR (400 MHz, CDCl3)

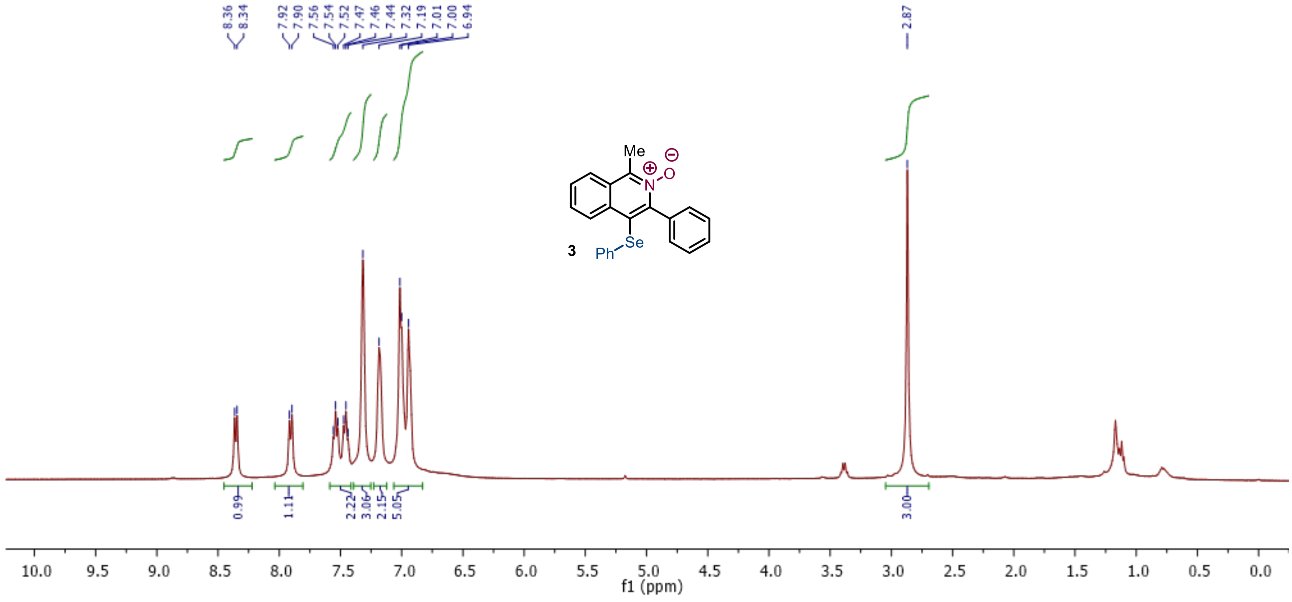

^{13}C NMR (101 MHz, $CDCl_3$)

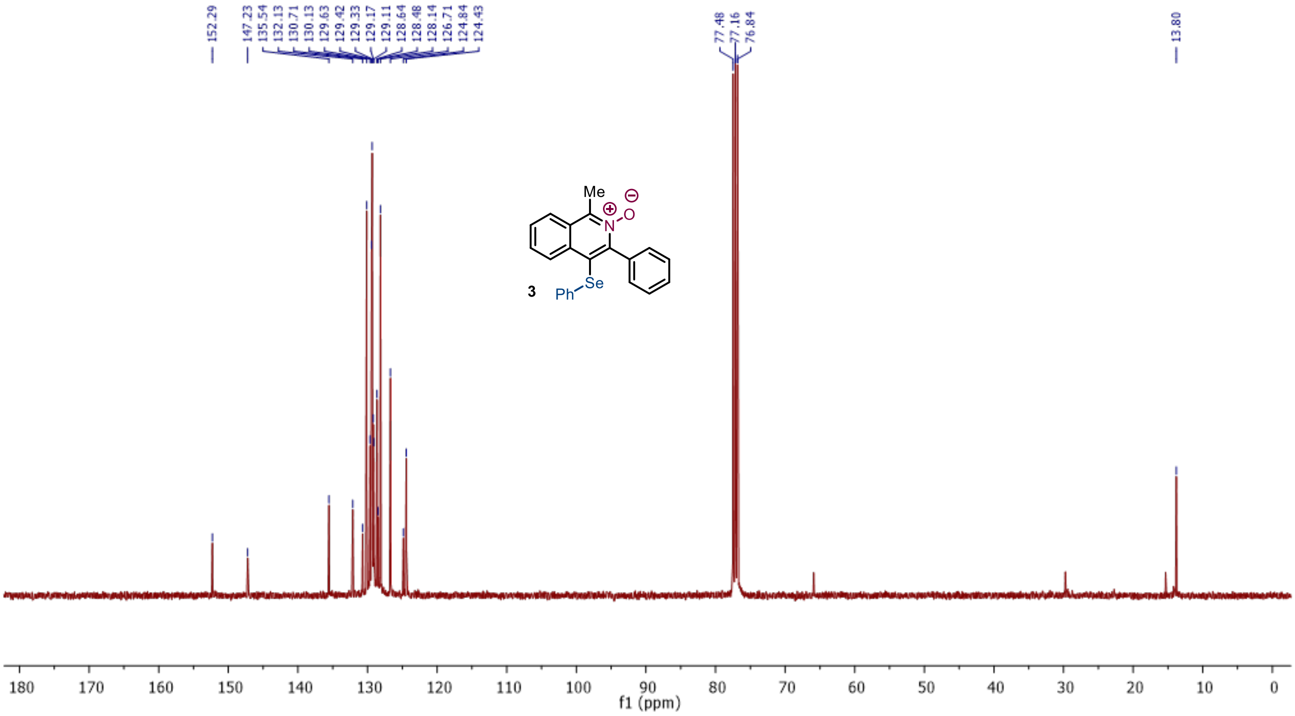

^{77}Se NMR (76 MHz, $CDCl_3$)

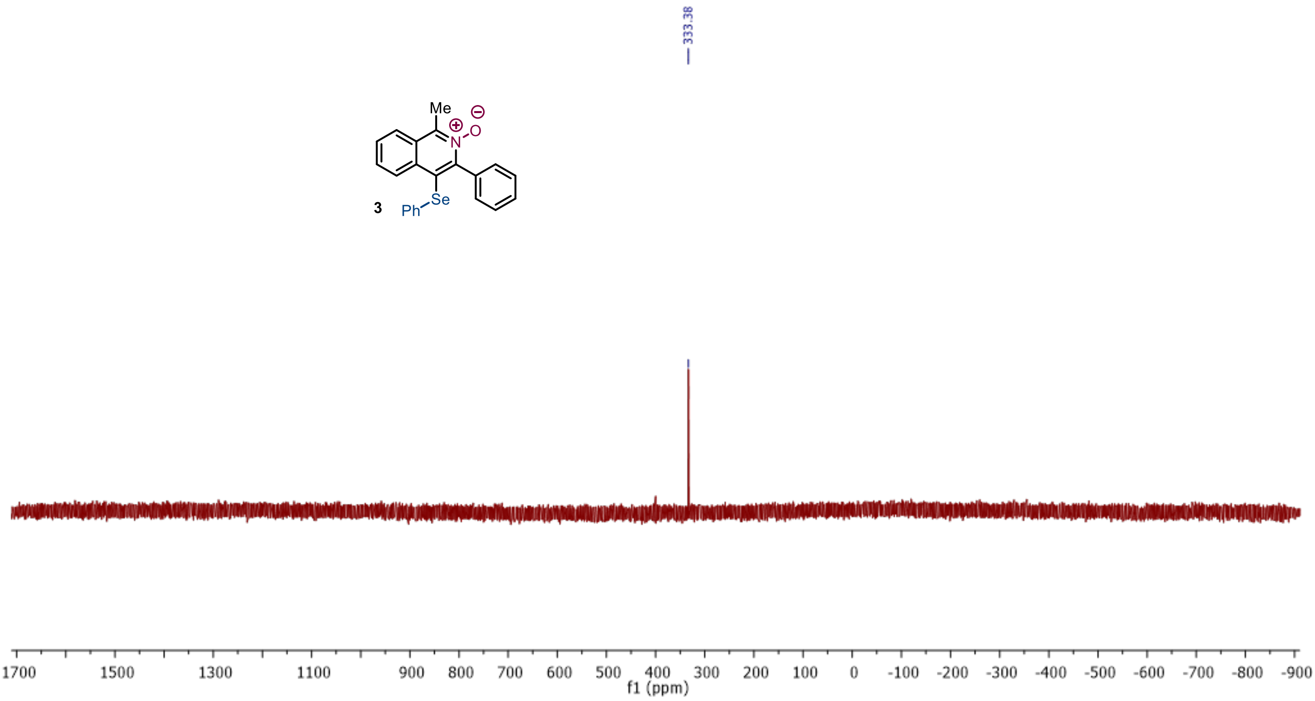

3-(4-Methoxyphenyl)-1-methyl-4-(phenylselanyl)isoquinoline 2-oxide (5)

^{1}H NMR (500 MHz, $CDCl_3$)

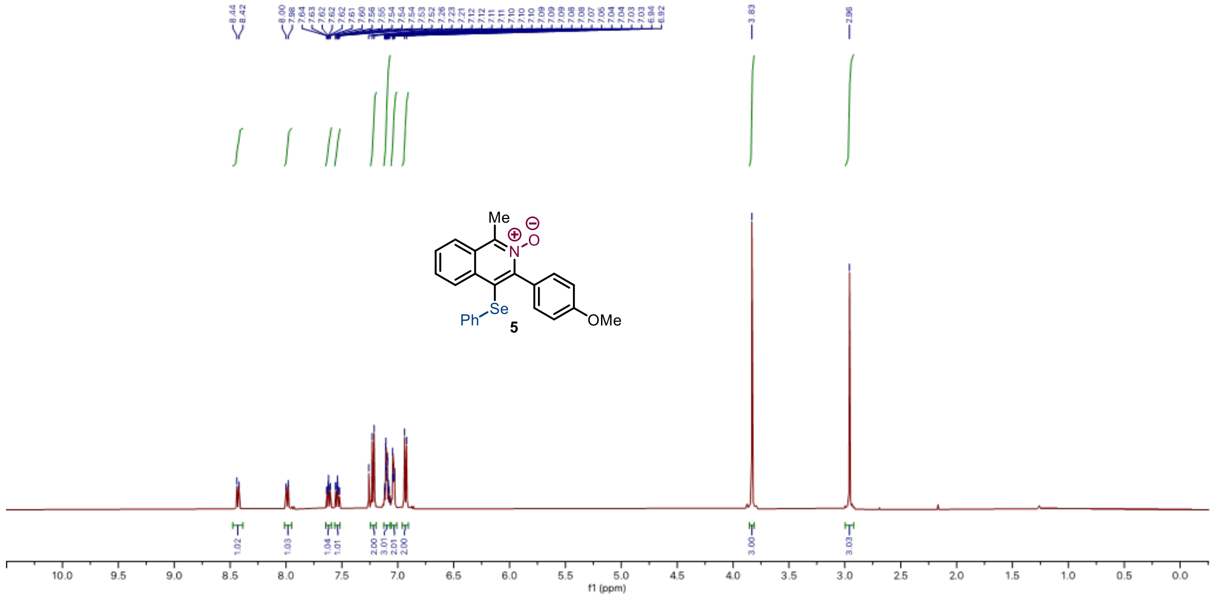

^{13}C NMR (126 MHz, $CDCl_3$)

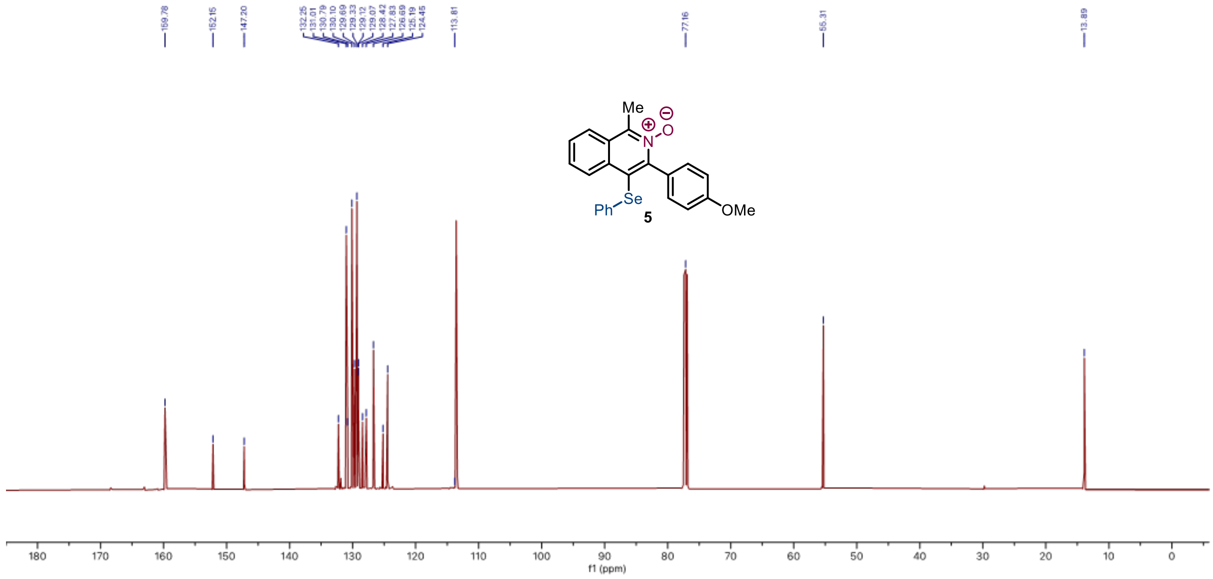

^{77}Se NMR (76 MHz, $CDCl_3$)

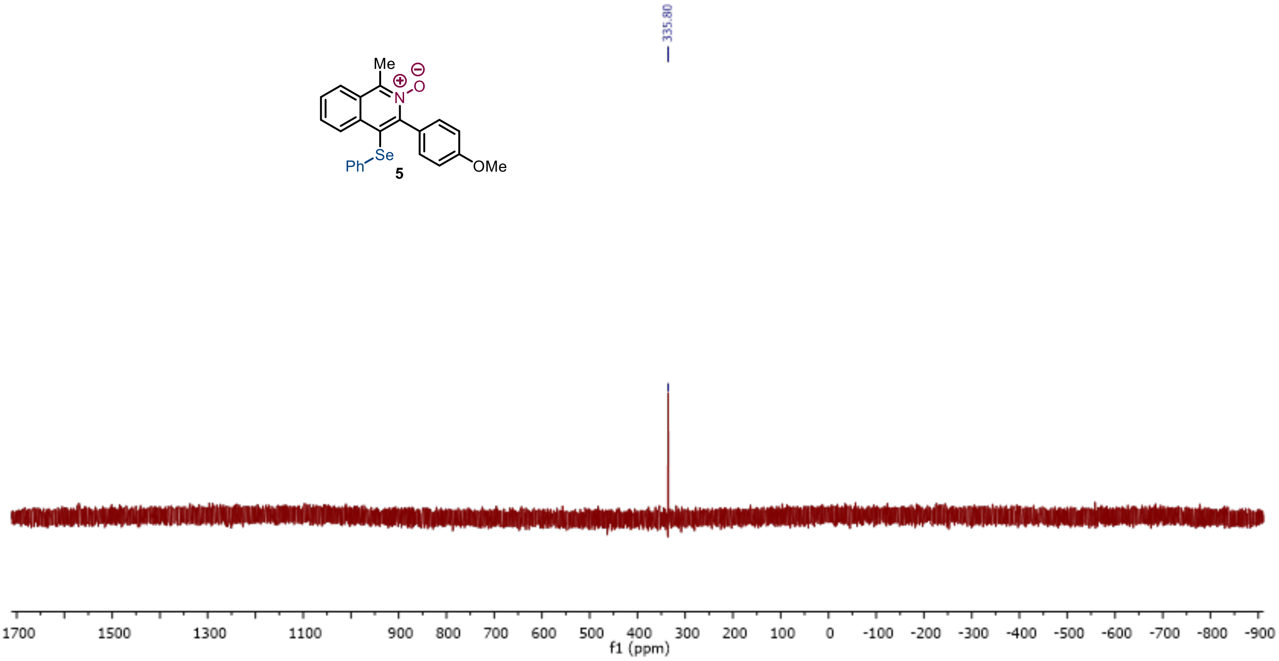

1-Methyl-4-(phenylselanyl)-3-(p-tolyl)isoquinoline 2-oxide (6)

^{1}H NMR (400 MHz, CDCl$_3$)

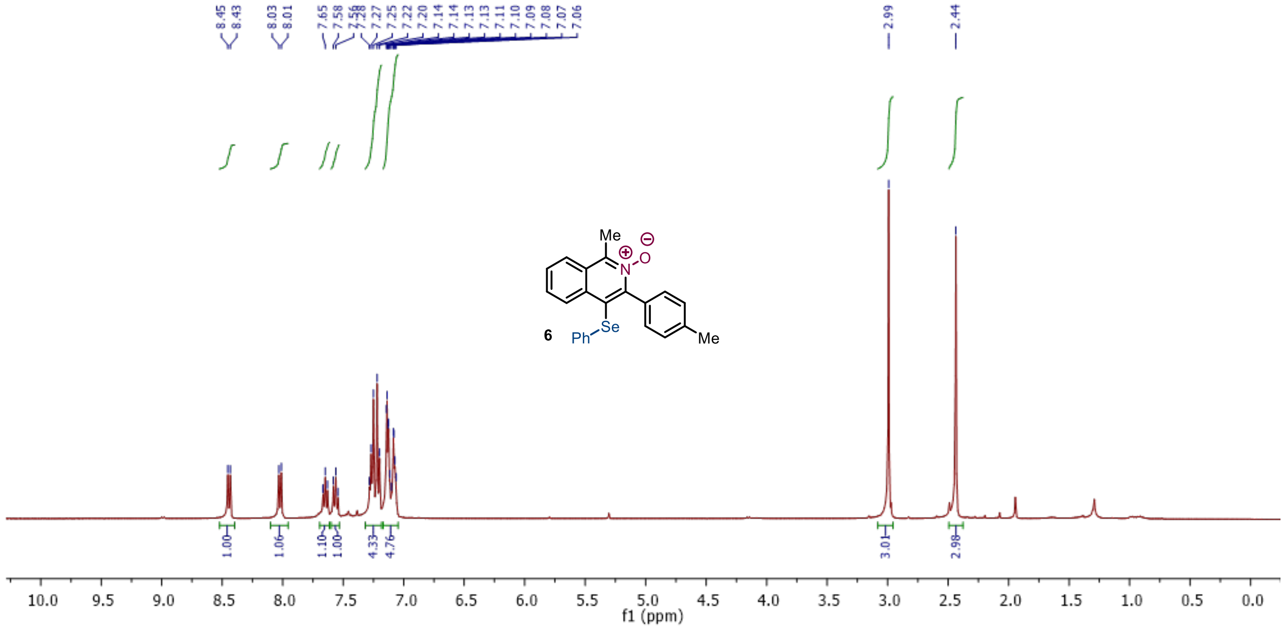

^{13}C NMR (101 MHz, CDCl$_3$)

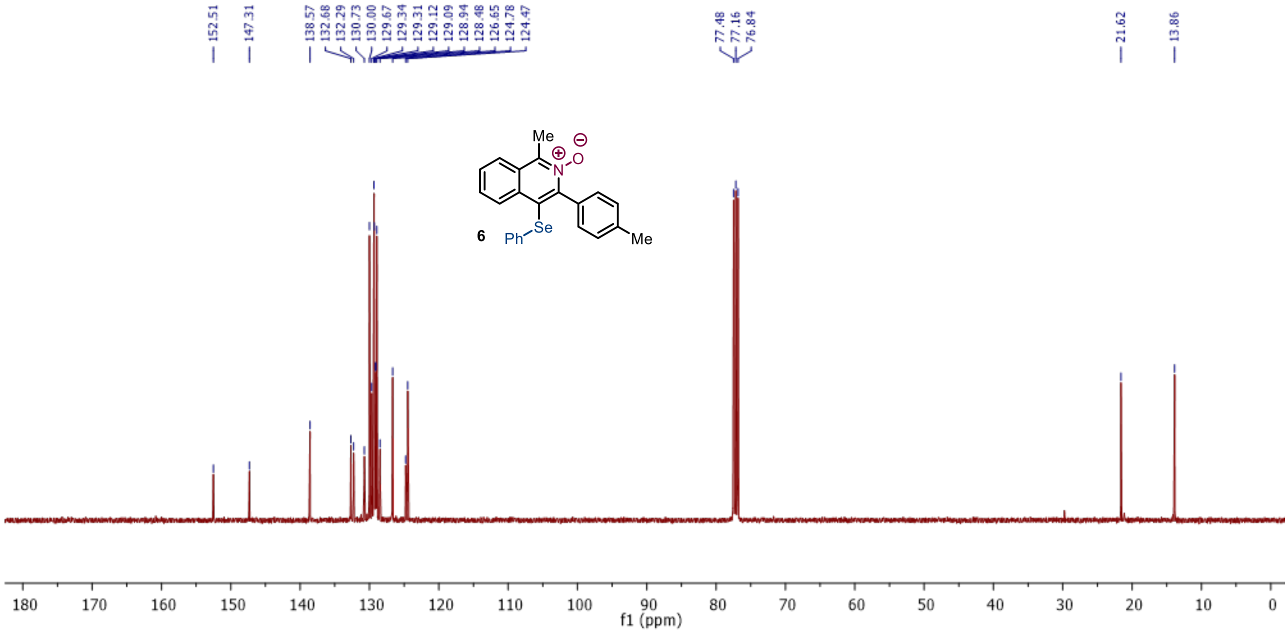

^{77}Se NMR (76 MHz, CDCl$_3$)

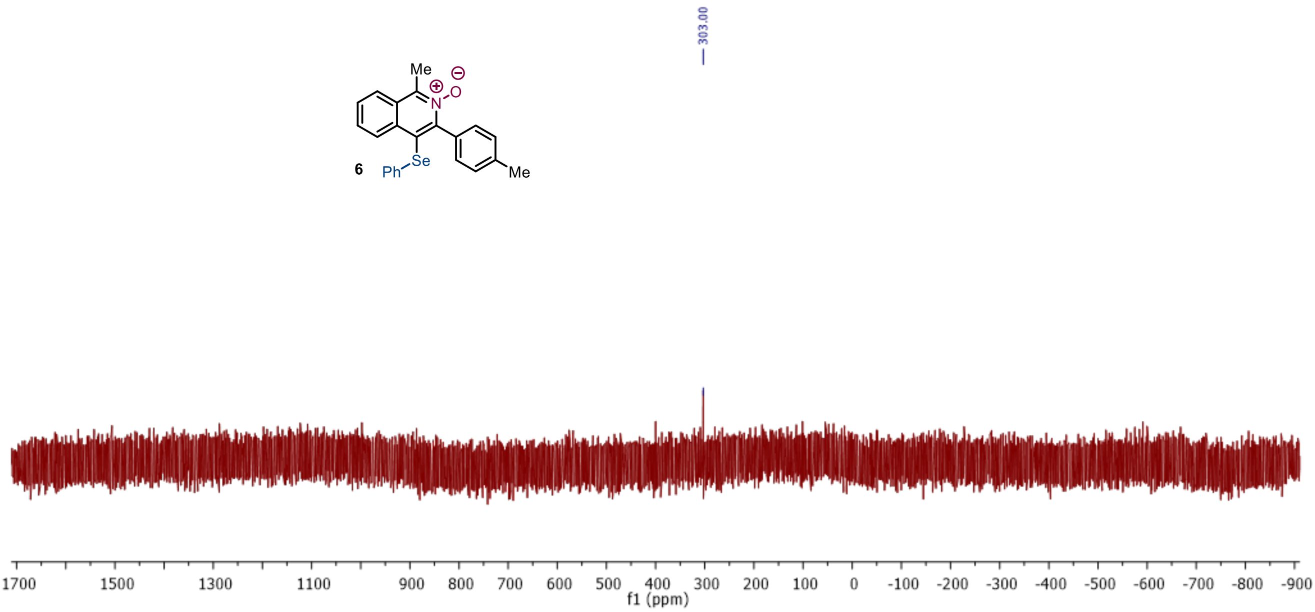

3-(4-Chlorophenyl)-1-methyl-4-(phenylselanyl)isoquinoline 2-oxide (7)

^{1}H NMR (400 MHz, CDCl$_3$)

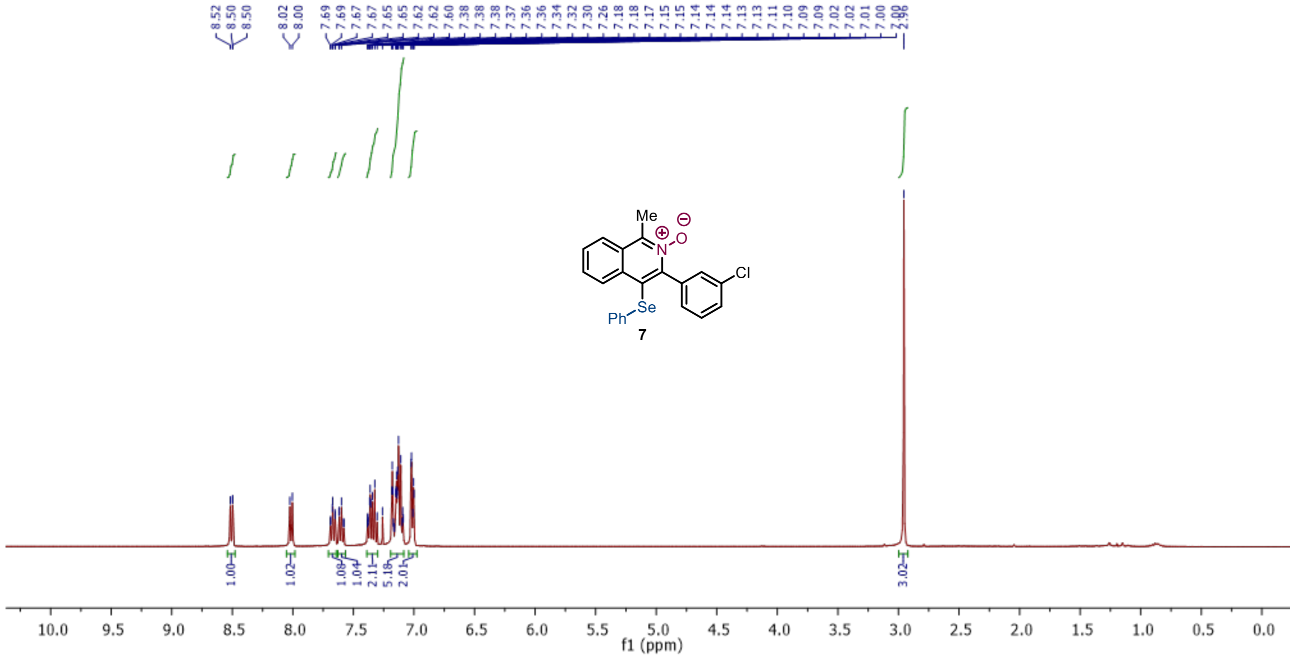

^{13}C NMR (101 MHz, $CDCl_3$)

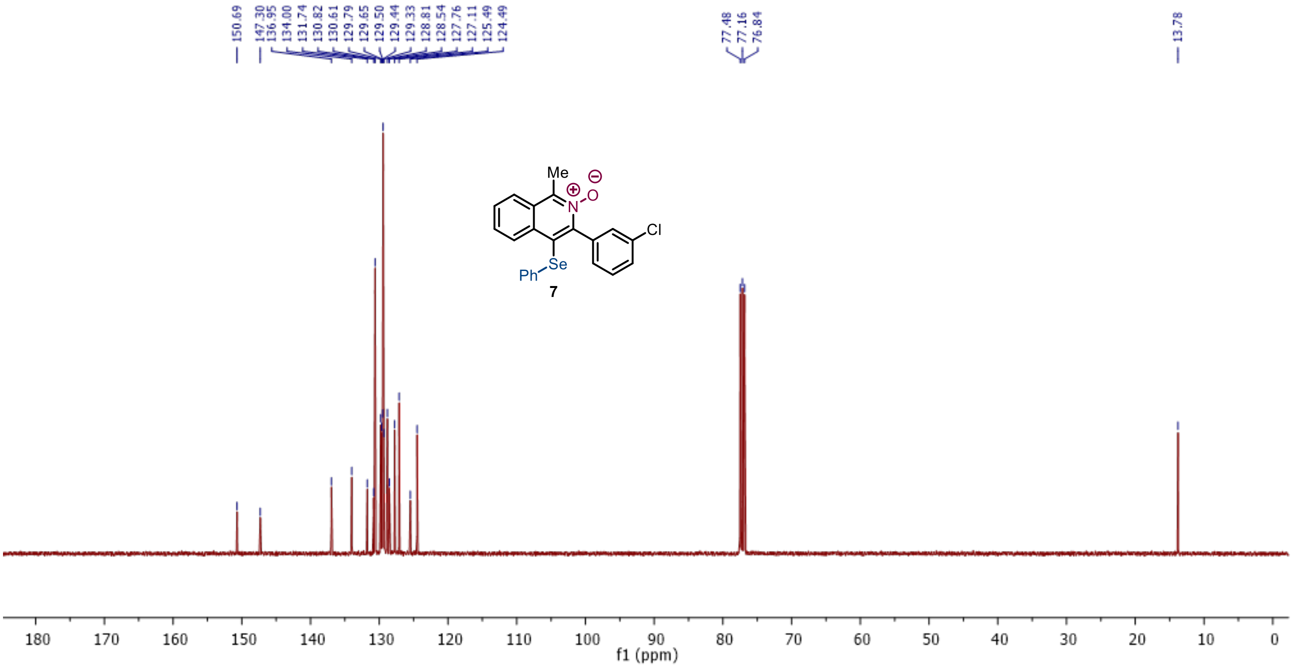

NMR (76 MHz, $CDCl_3$)

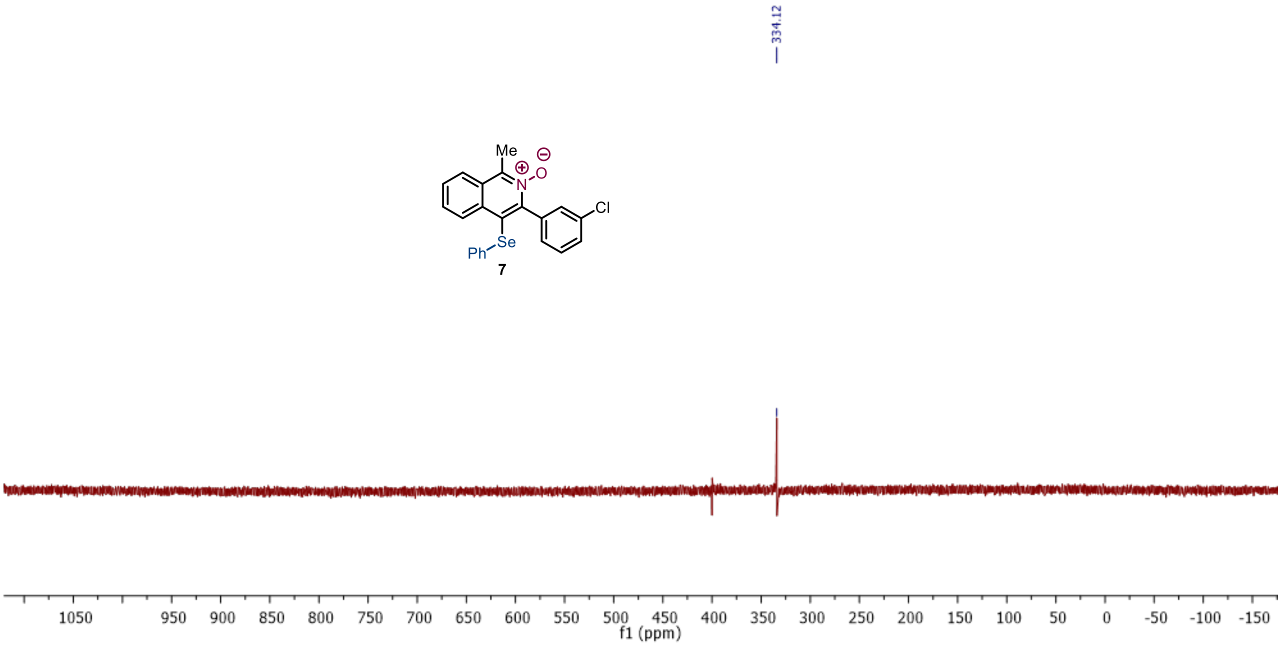

3-(4-Fluorophenyl)-1-methyl-4-(phenylselanyl)isoquinoline 2-oxide (8)

^{1}H NMR (400 MHz, $CDCl_3$)

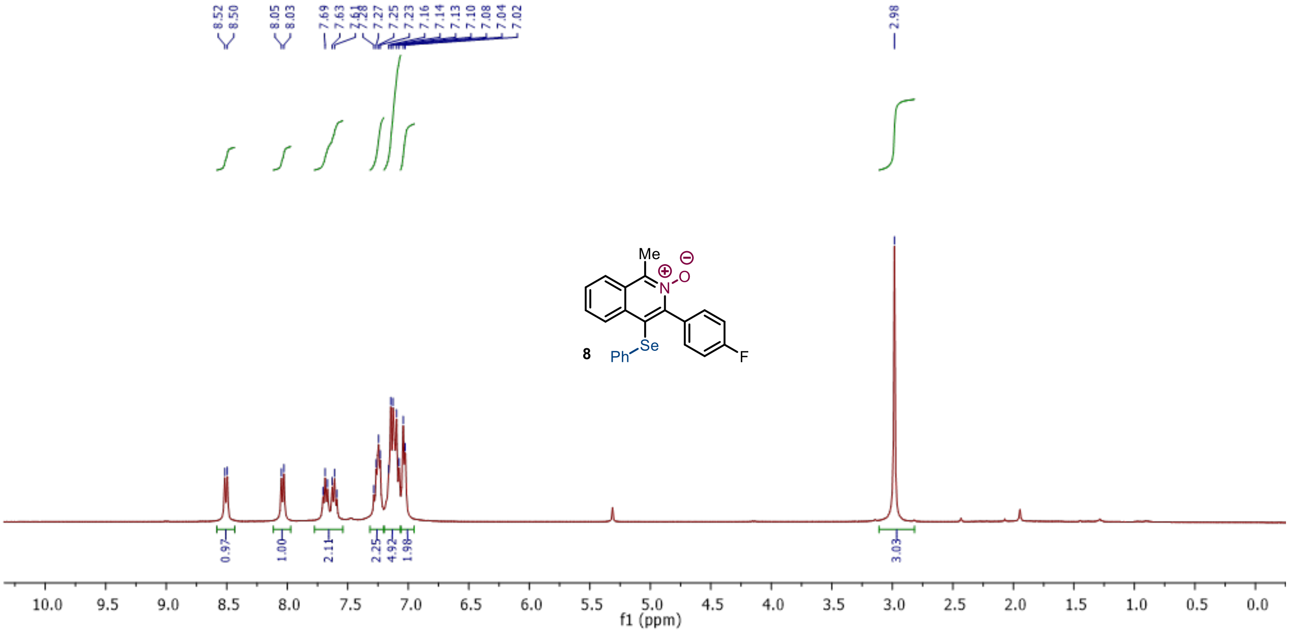

^{13}C NMR (101 MHz, $CDCl_3$)

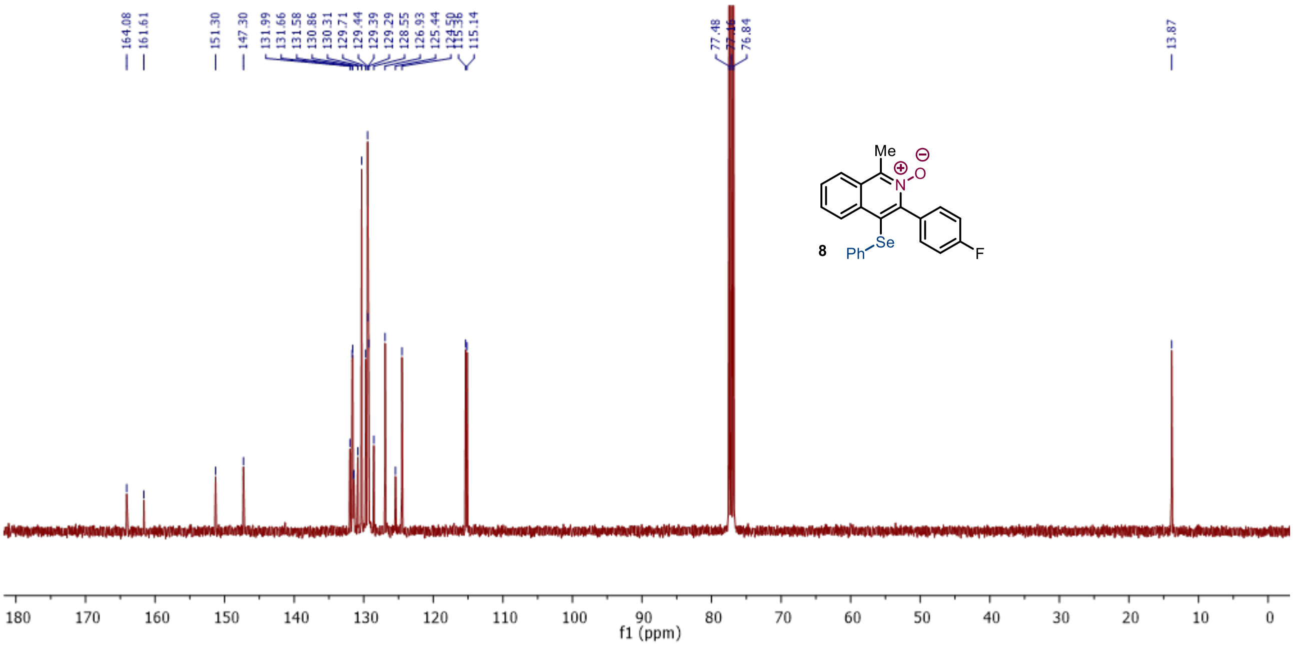

^{77}Se NMR (76 MHz, $CDCl_3$)

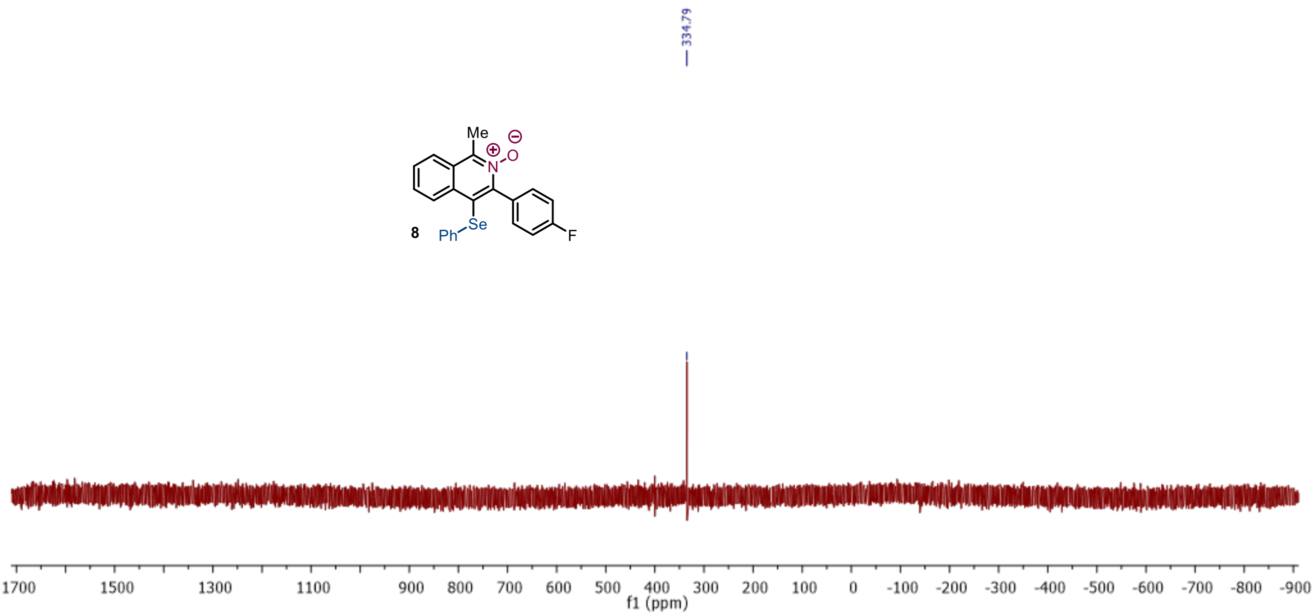

^{19}F NMR (376 MHz, $CDCl_3$)

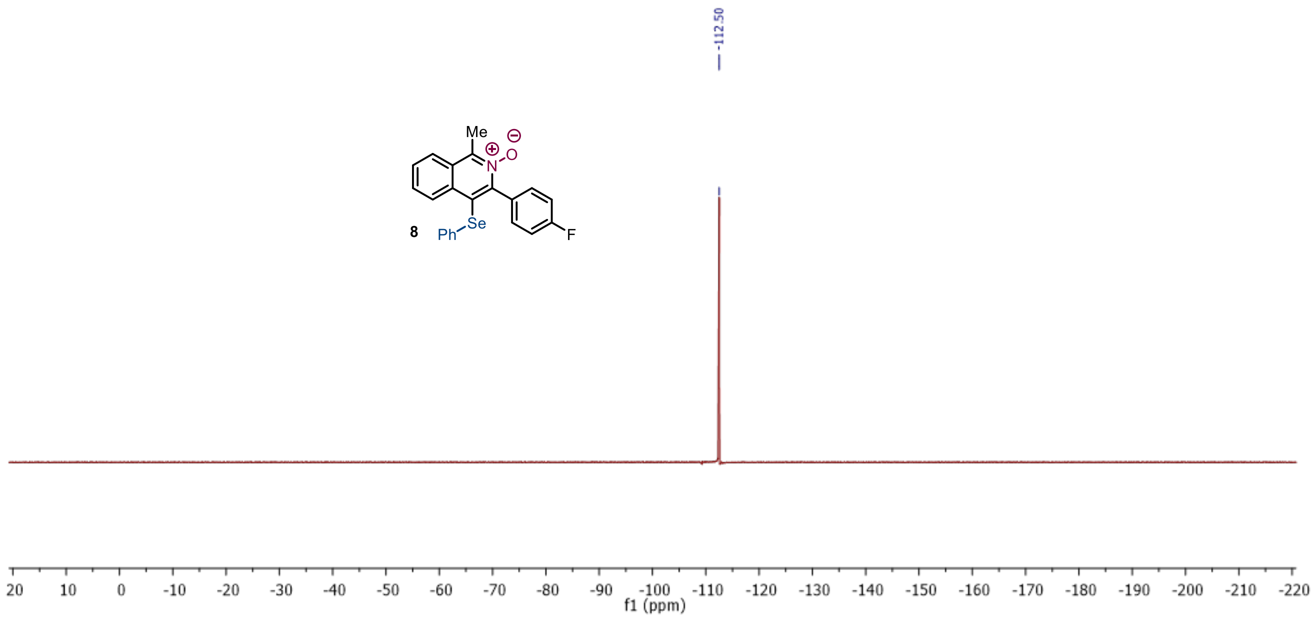

1-Methyl-4-(phenylselanyl)-3-(4-(trifluoromethyl)phenyl)isoquinoline 2-oxide (9)

^{1}H NMR (400 MHz, $CDCl_3$)

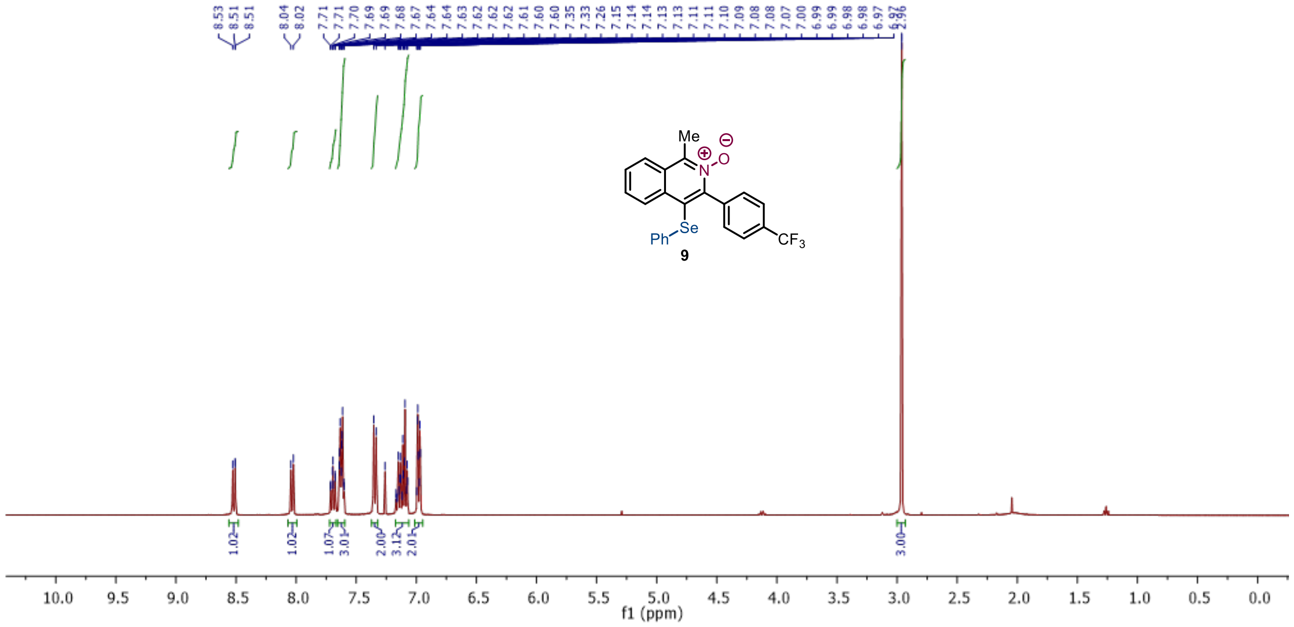

^{13}C NMR (101 MHz, $CDCl_3$)

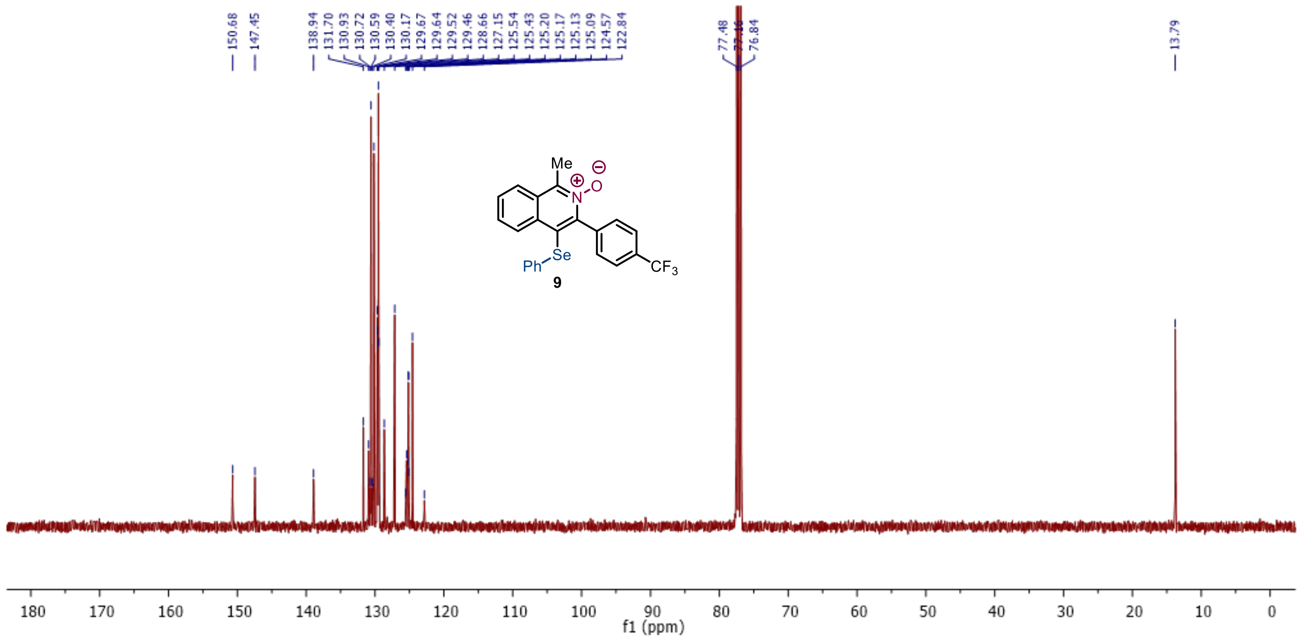

^{19}F NMR (376 MHz, $CDCl_3$)

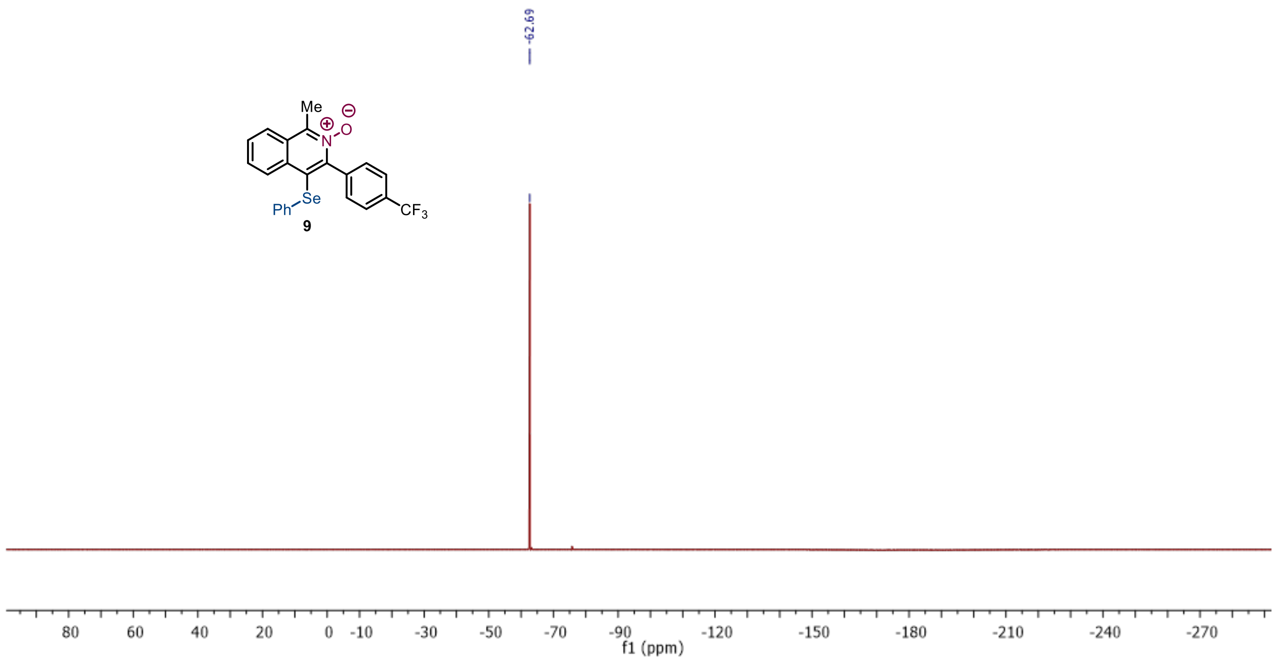

^{77}Se NMR (76 MHz, $CDCl_3$)

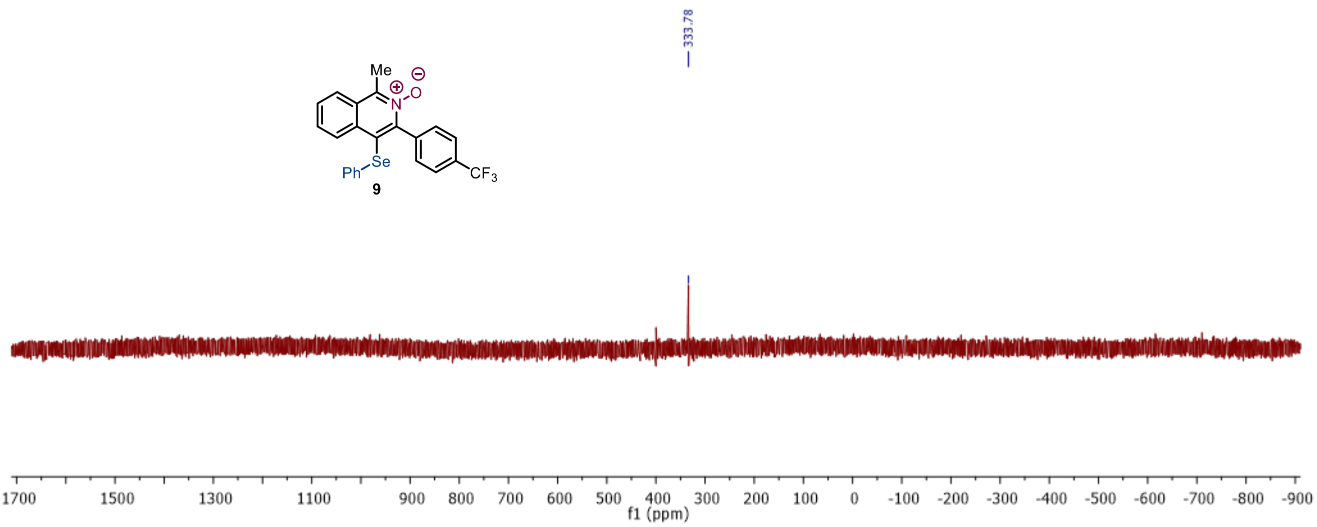

3-(4-(Methoxycarbonyl)phenyl)-1-methyl-4-(phenylselanyl)isoquinoline 2-oxide (10)

^{1}H NMR (500 MHz, $CDCl_3$)

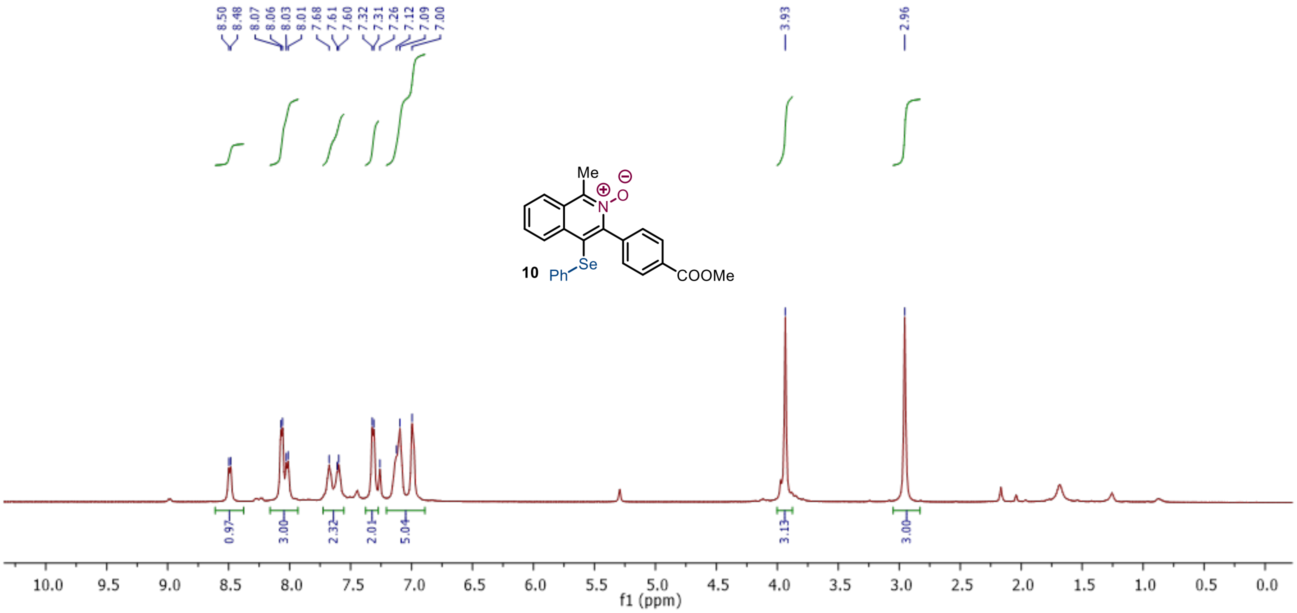

^{13}C NMR (101 MHz, $CDCl_3$)

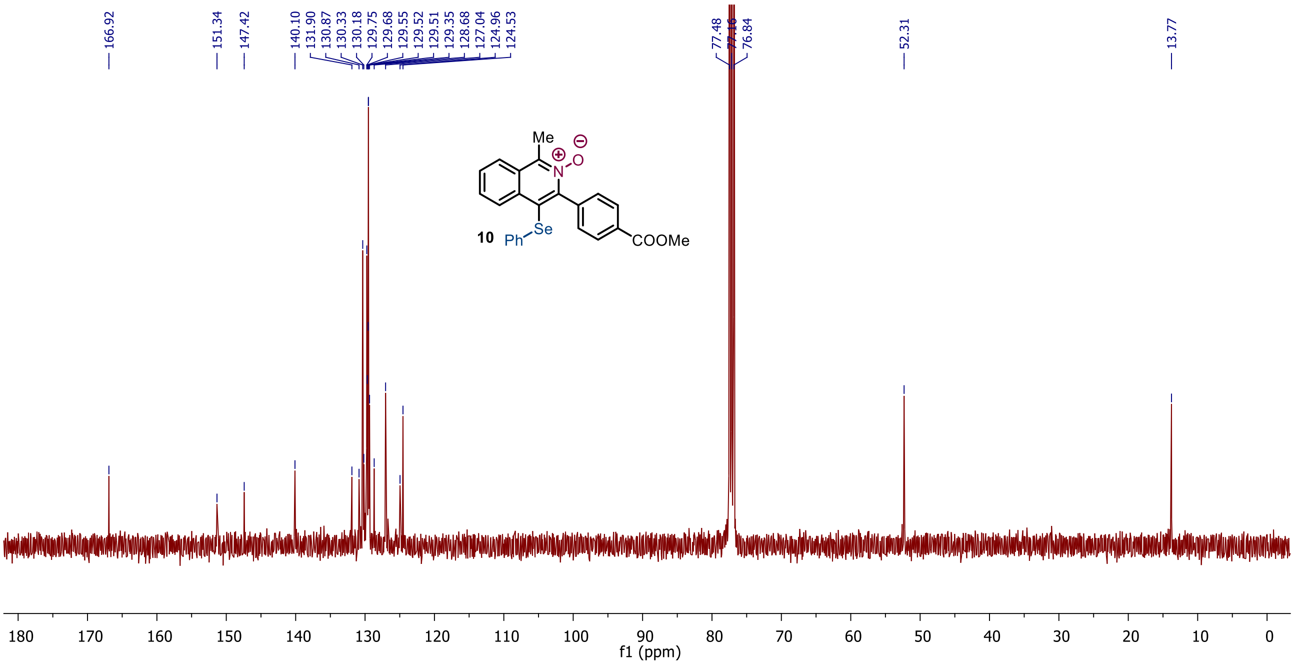

^{77}Se NMR (76 MHz, $CDCl_3$)

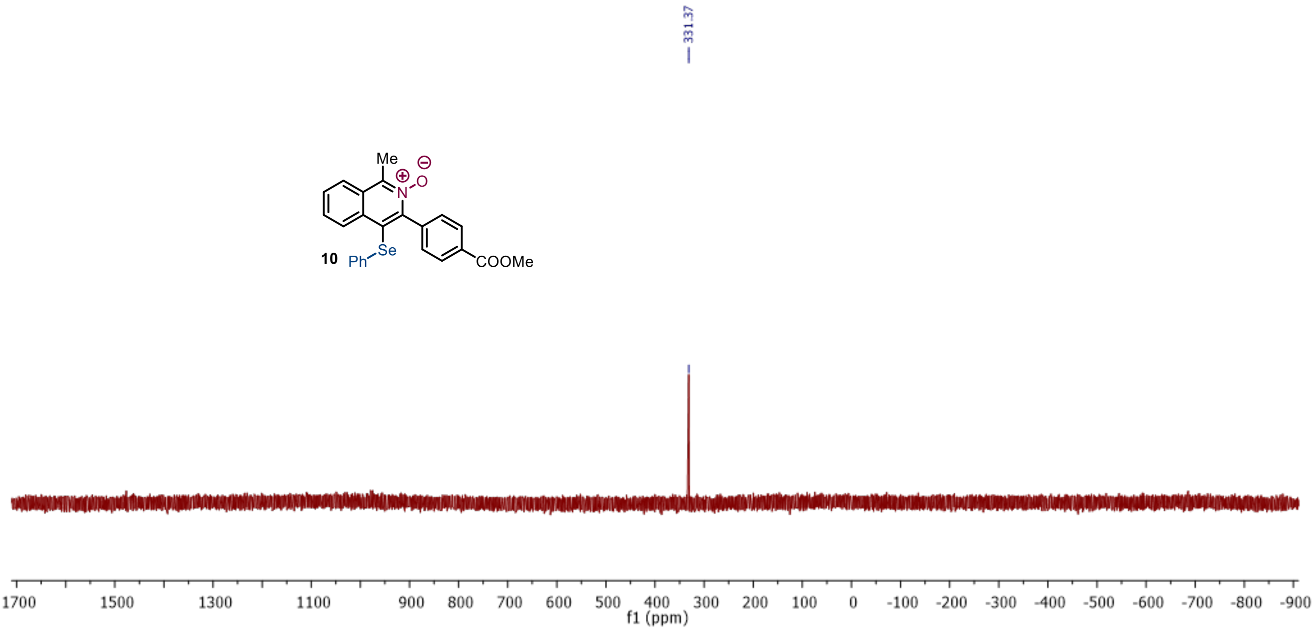

1-Methyl-4-(phenylselanyl)isoquinoline 2-oxide (11):

^{1}H NMR (400 MHz, $CDCl_3$)

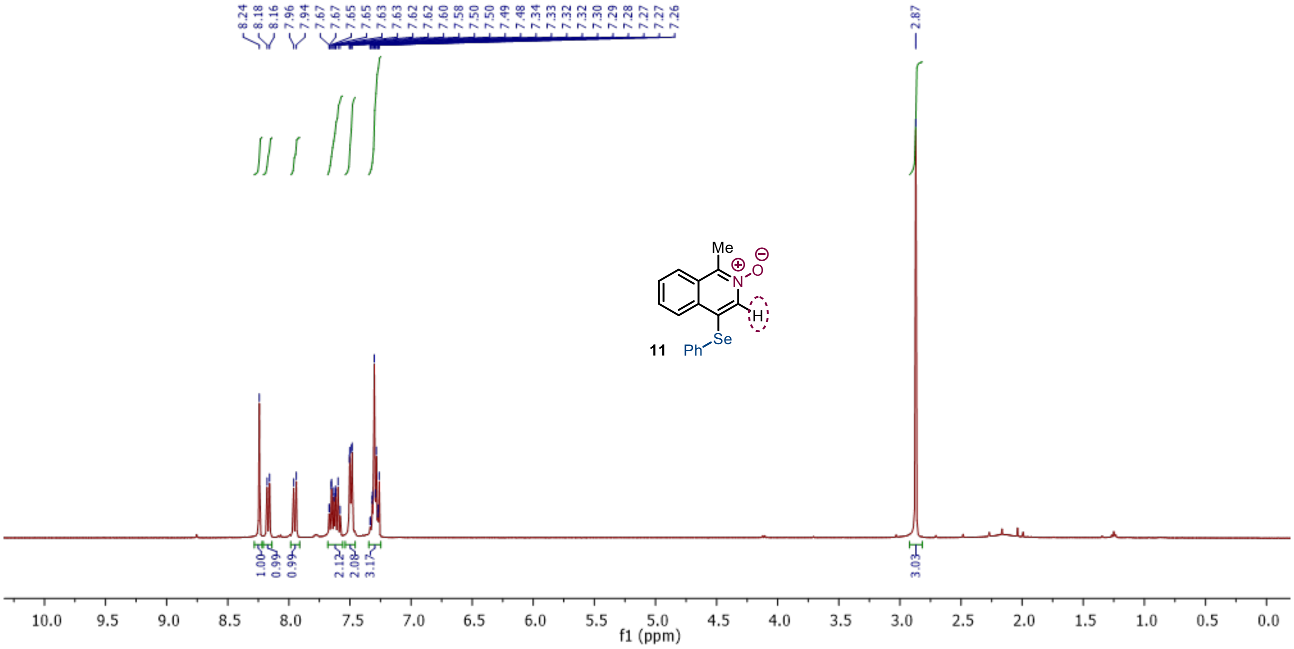

^{13}C NMR (101 MHz, $CDCl_3$)

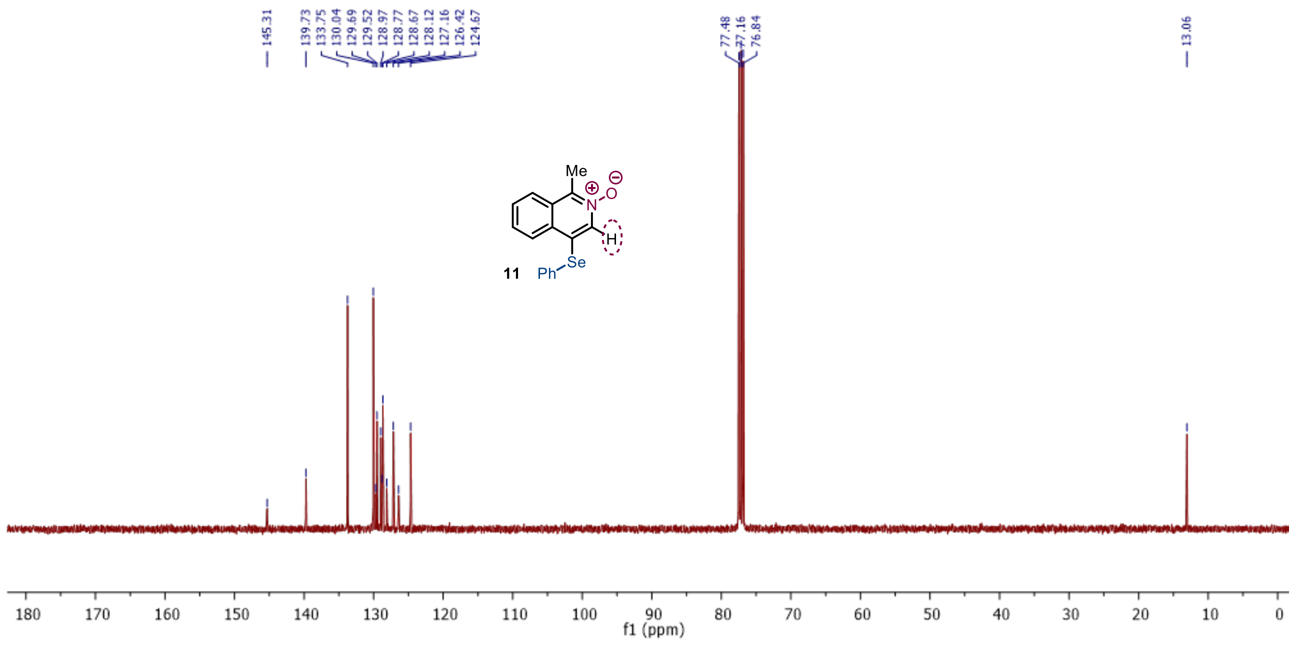

^{77}Se NMR (76 MHz, $CDCl_3$)

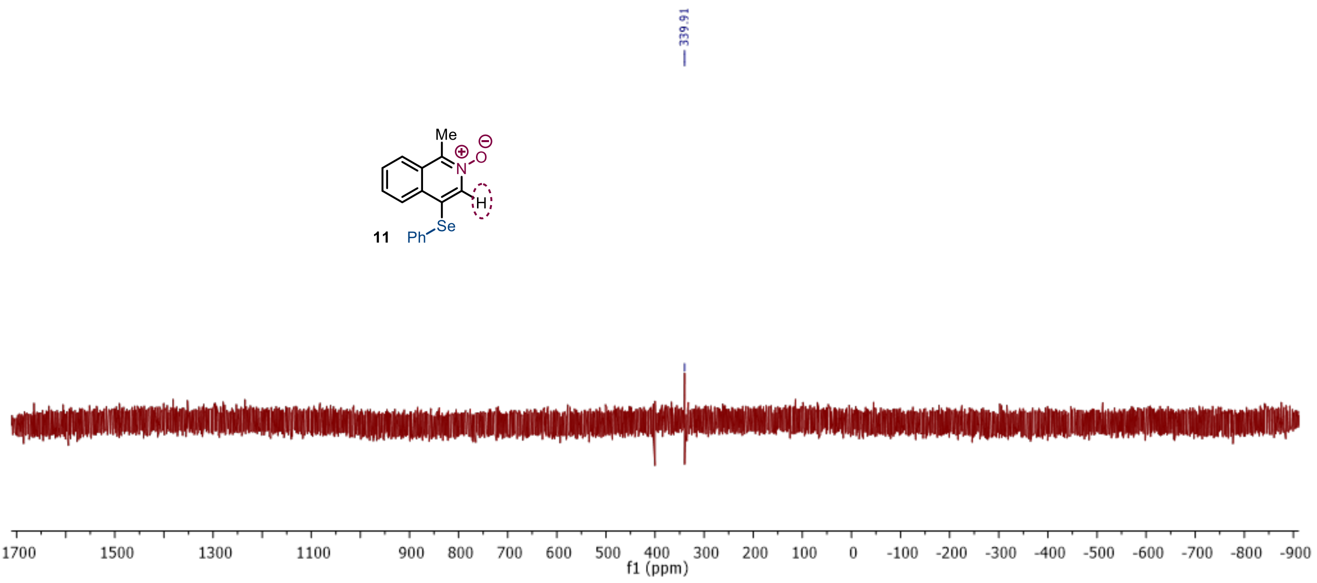

4-(Benzylselanyl)-1-methyl-3-phenylisoquinoline 2-oxide (12)

^{1}H NMR (400 MHz, $CDCl_3$)

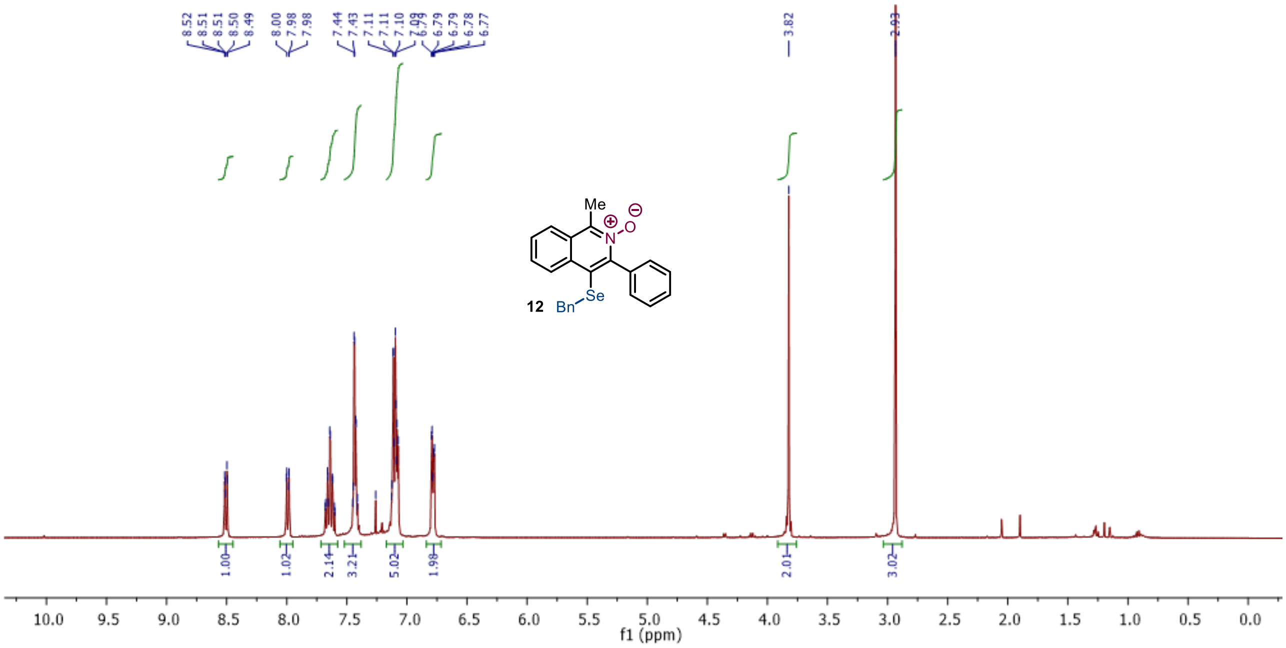

^{13}C NMR (101 MHz, $CDCl_3$)

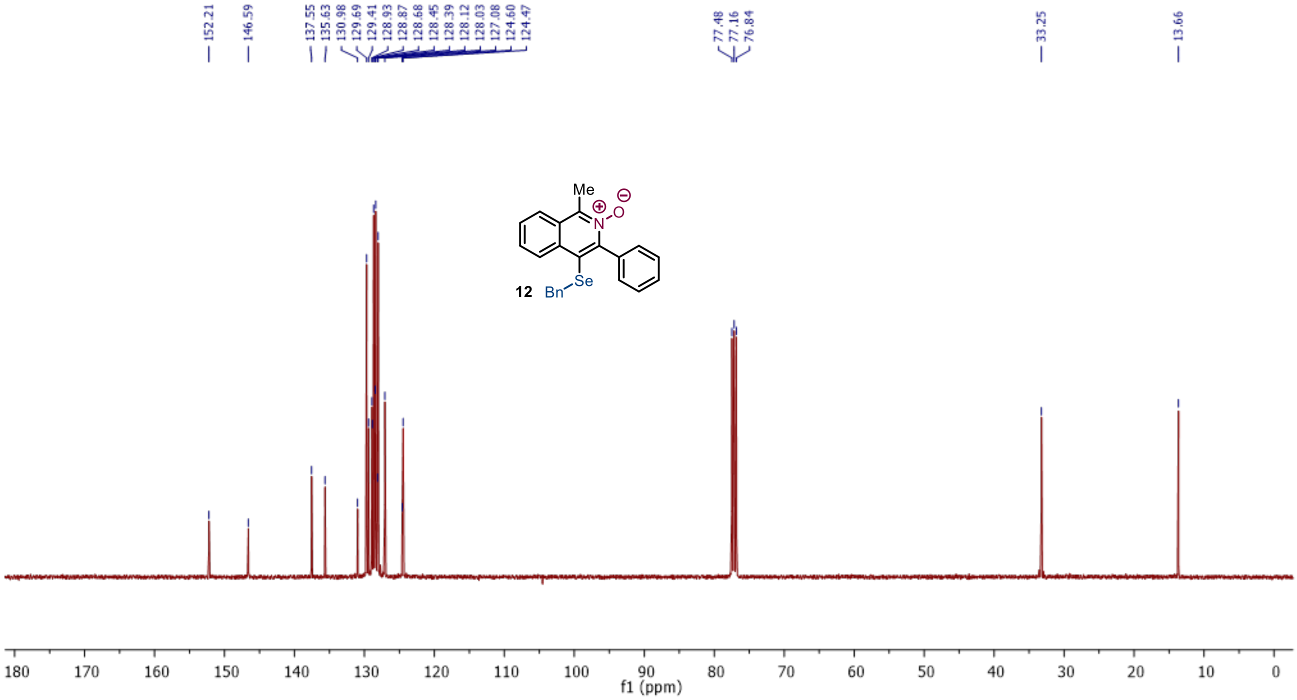

^{77}Se NMR (76 MHz, $CDCl_3$)

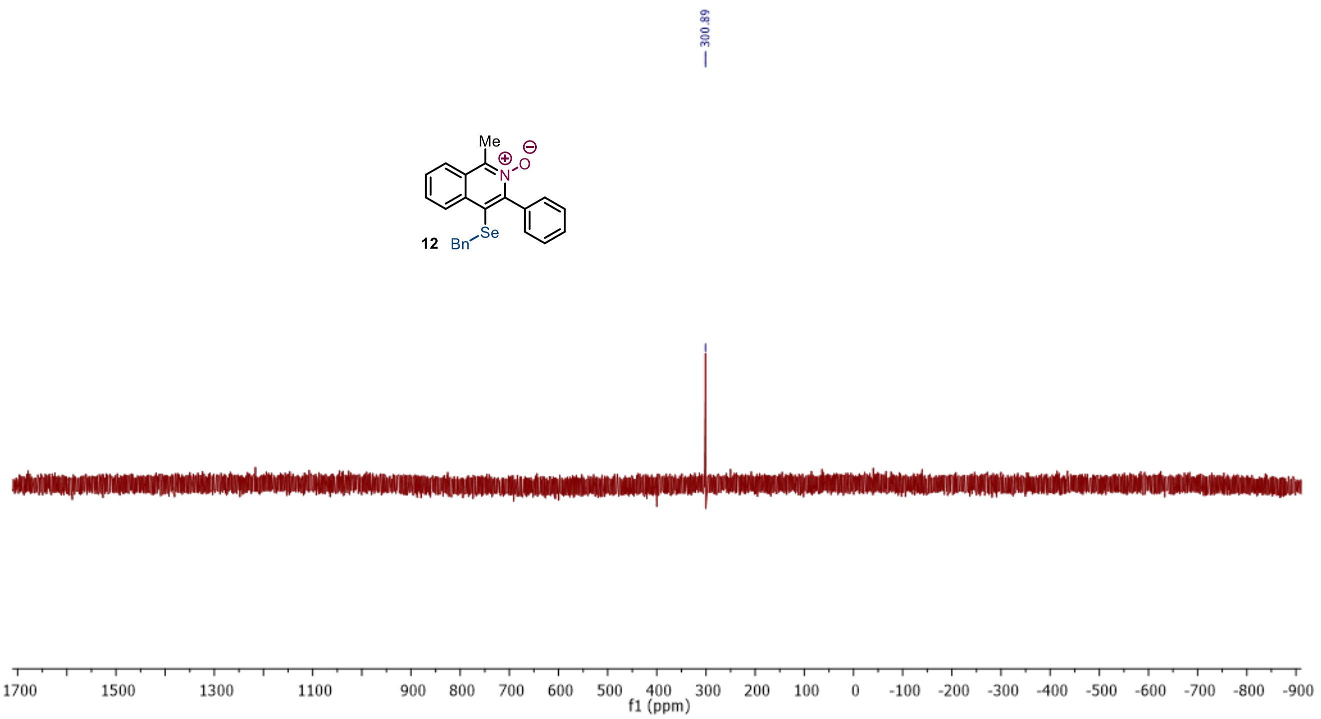

4-(Benzylselanyl)-1-methyl-3-(p-tolyl)isoquinoline 2-oxide (13):

^{1}H NMR (400 MHz, $CDCl_3$)

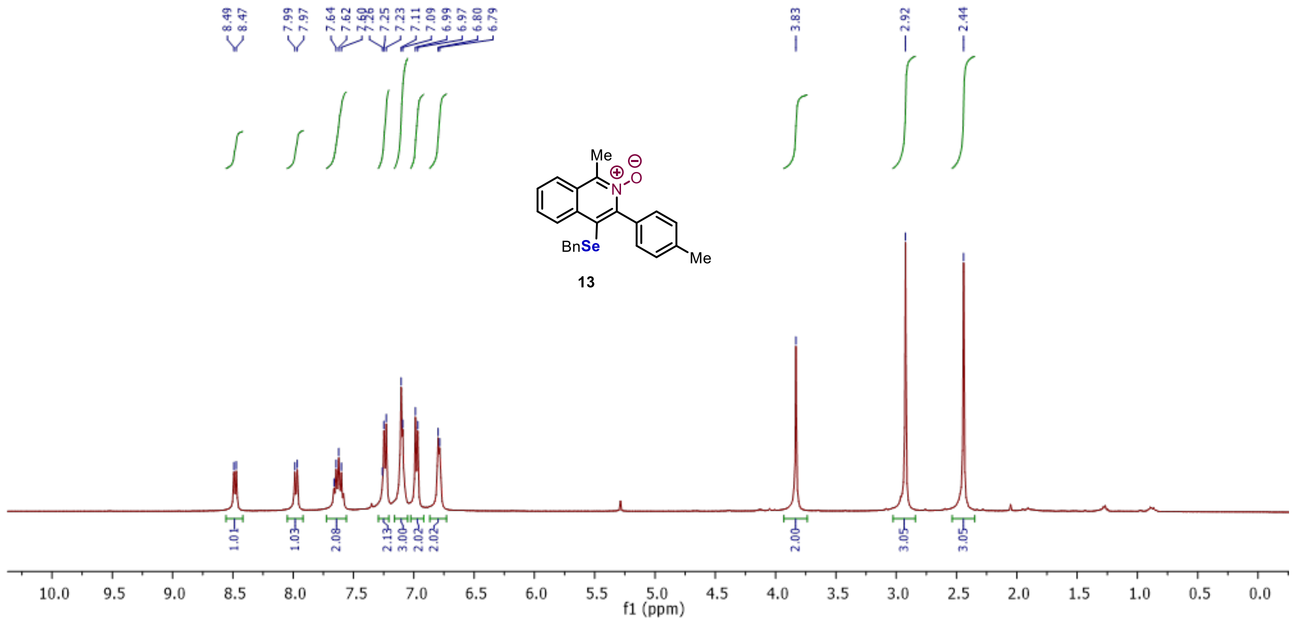

^{13}C NMR (101 MHz, $CDCl_3$)

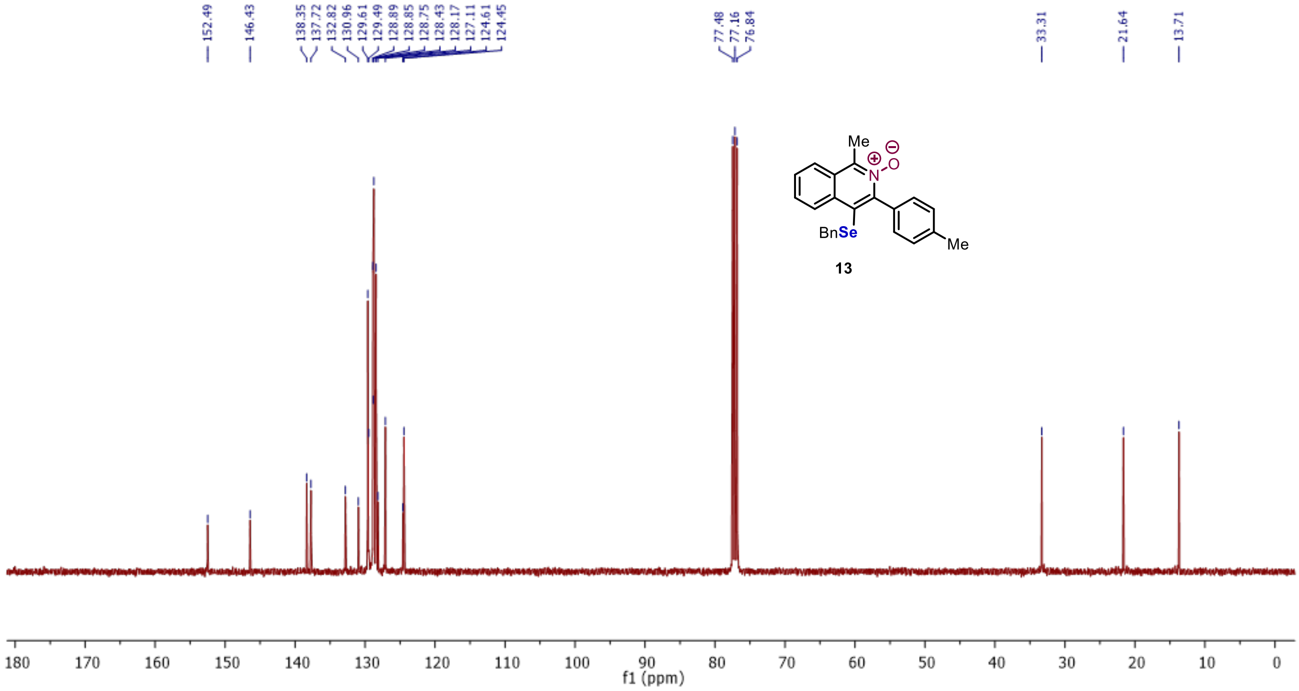

^{77}Se NMR (76 MHz, $CDCl_3$)

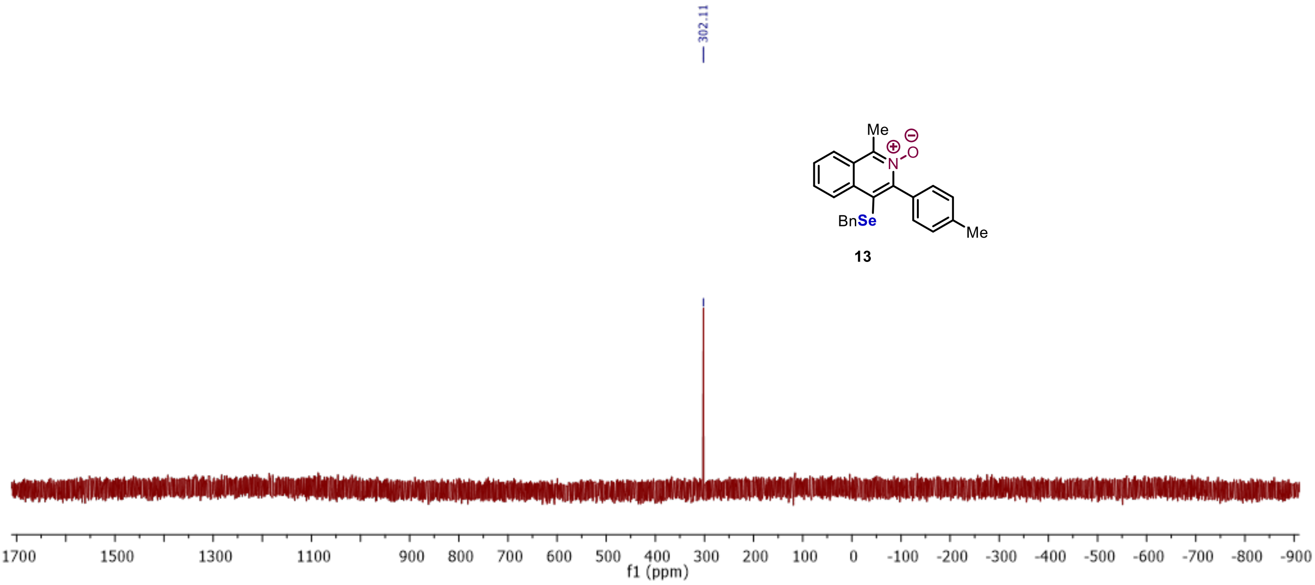

4-(Benzylselanyl)-3-(4-fluorophenyl)-1-methylisoquinoline 2-oxide (14)

^1H NMR (400 MHz, $CDCl_3$)

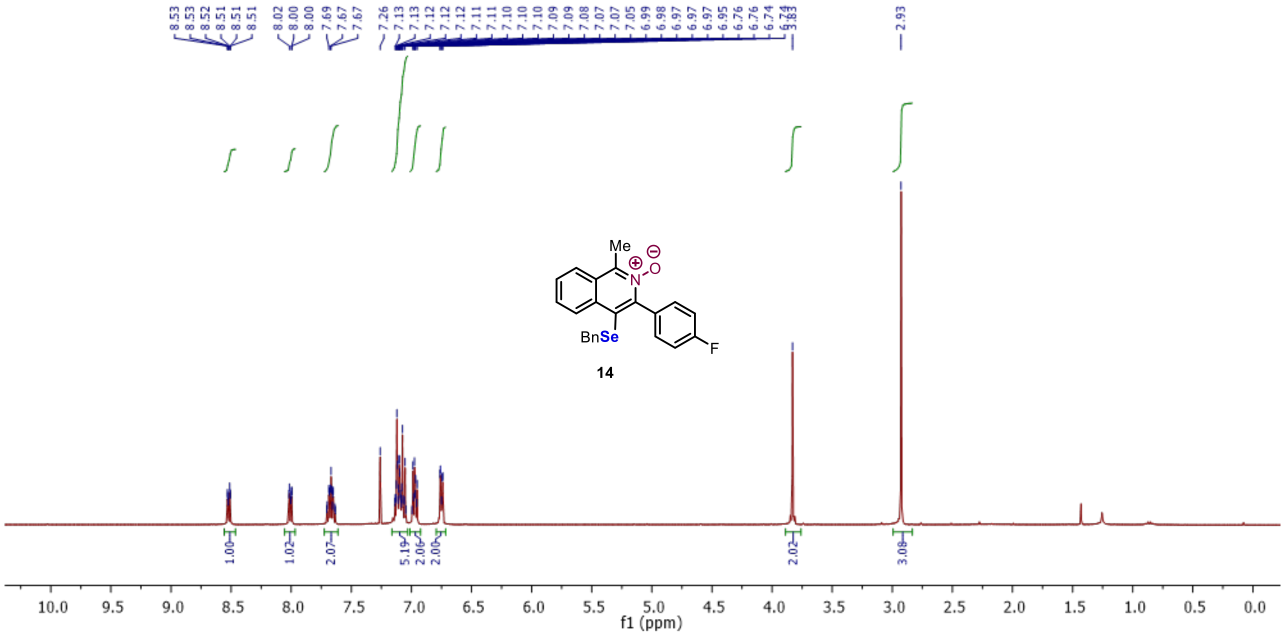

^{13}C NMR (101 MHz, $CDCl_3$)

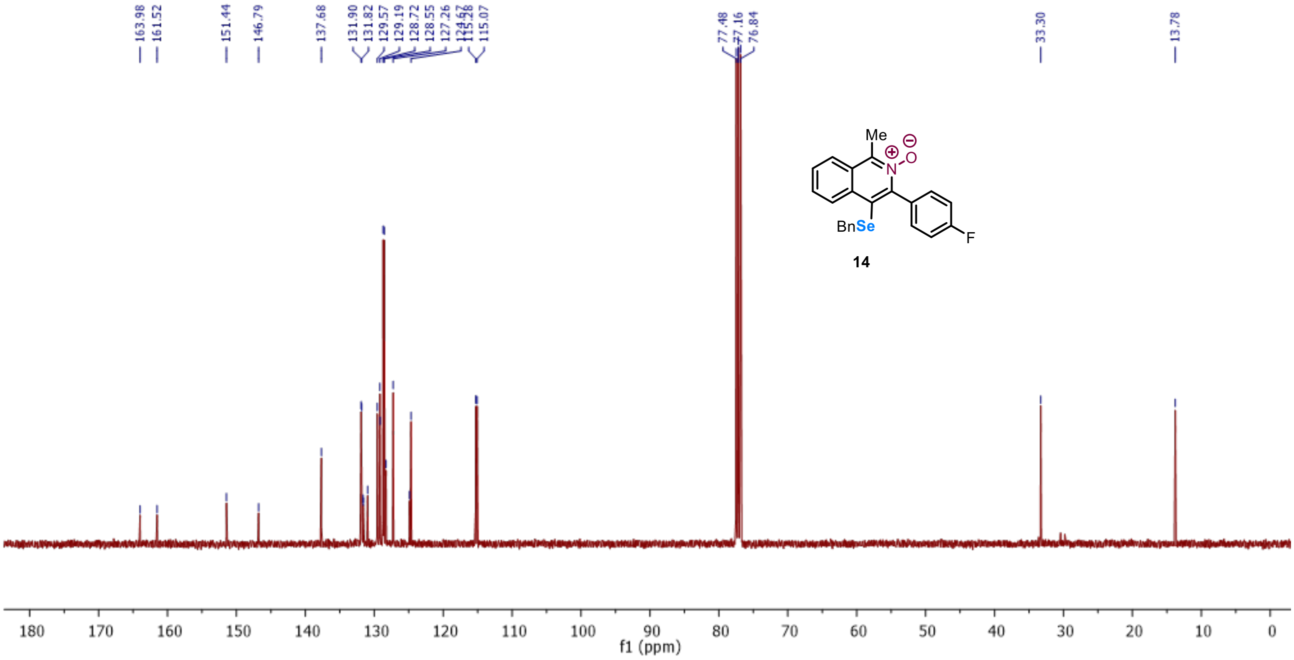

^{19}F NMR (376 MHz, $CDCl_3$)

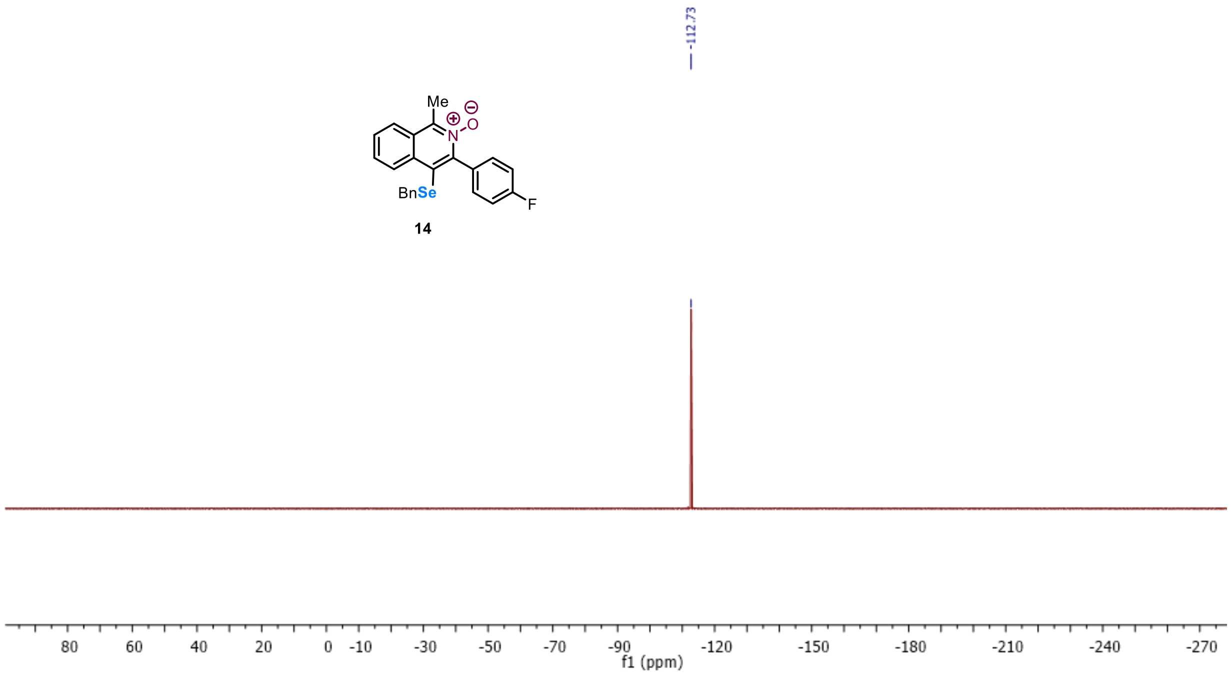

NMR (76 MHz, $CDCl_3$)

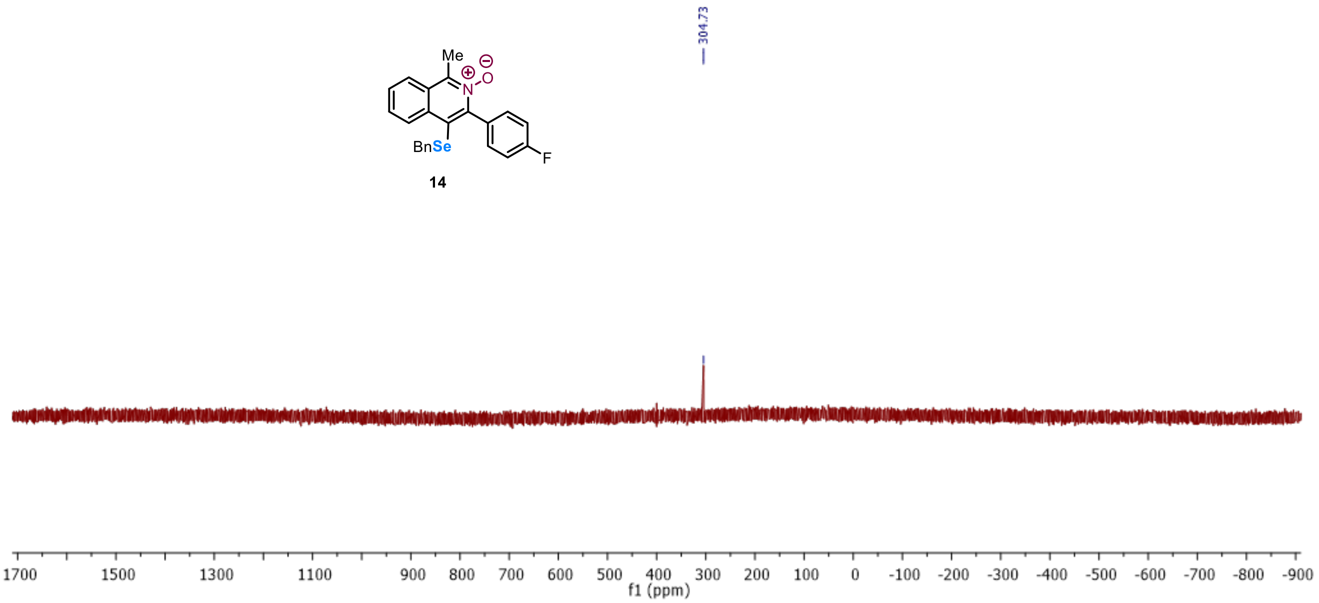

4-(Benzylselanyl)-1-methylisoquinoline 2-oxide (15)

^{1}H NMR (400 MHz, $CDCl_3$)

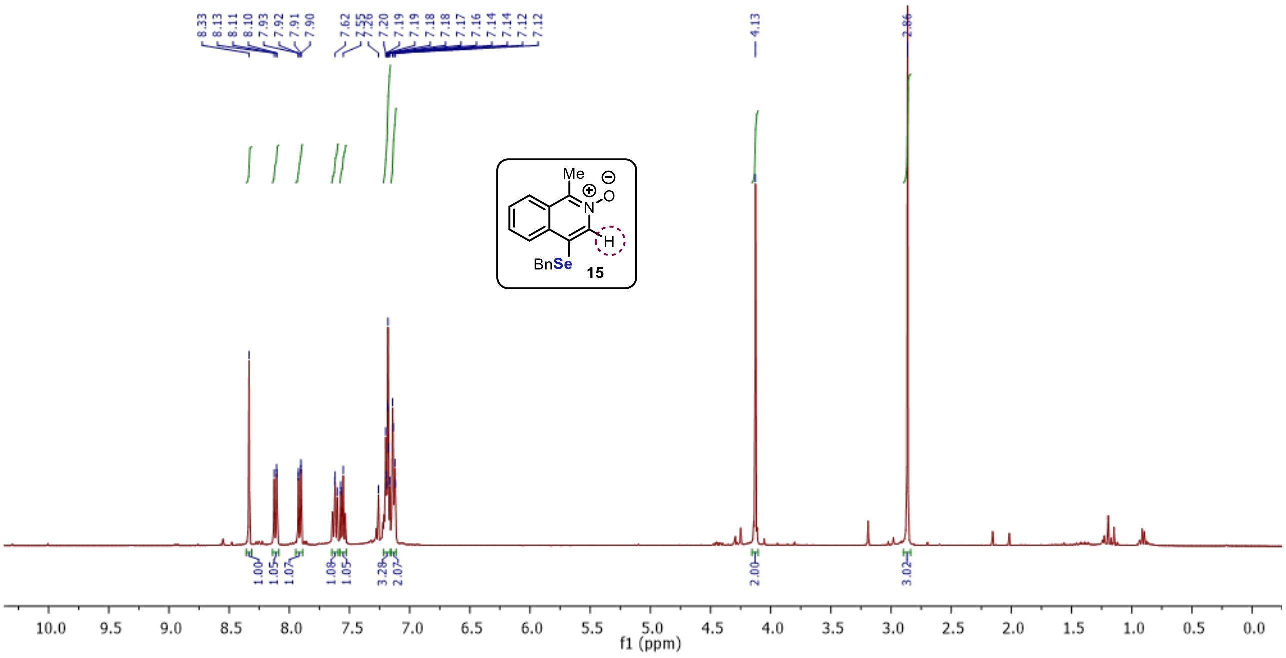

^{13}C NMR (101 MHz, $CDCl_3$)

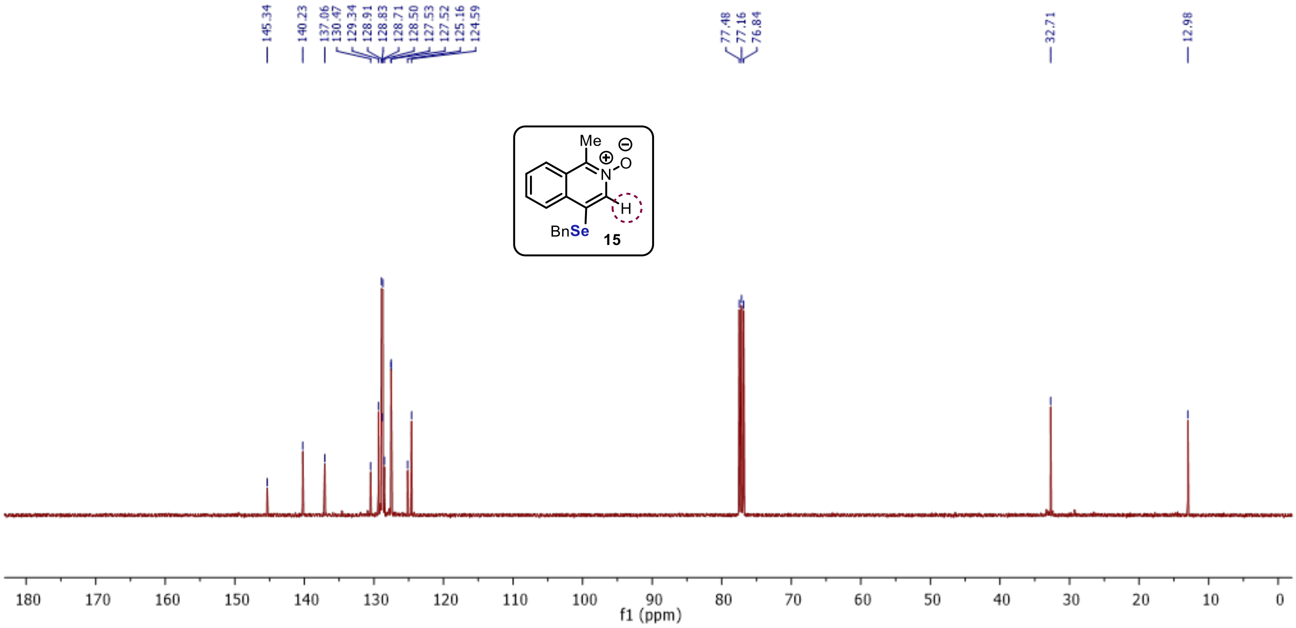

NMR (76 MHz, $CDCl_3$)

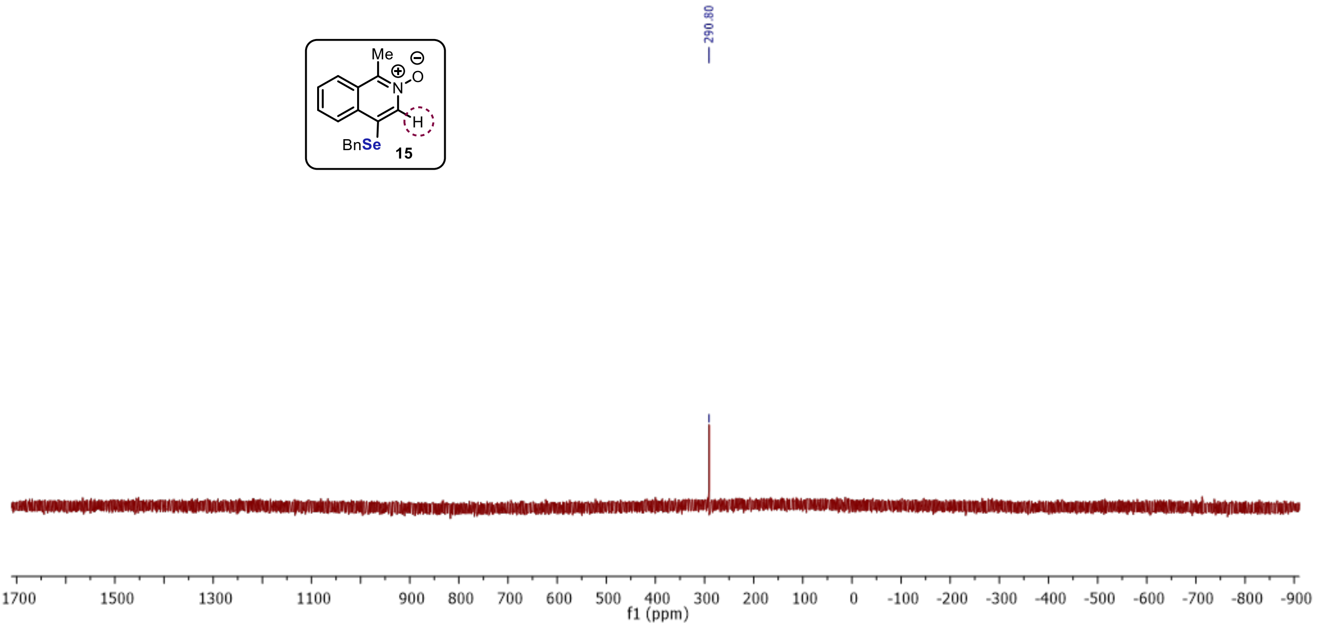

1-Methyl-3-(p-tolyl)-4-((4-(trifluoromethyl)phenyl)selanyl)isoquinoline 2-oxide (16)

^1H NMR (400 MHz, $CDCl_3$)

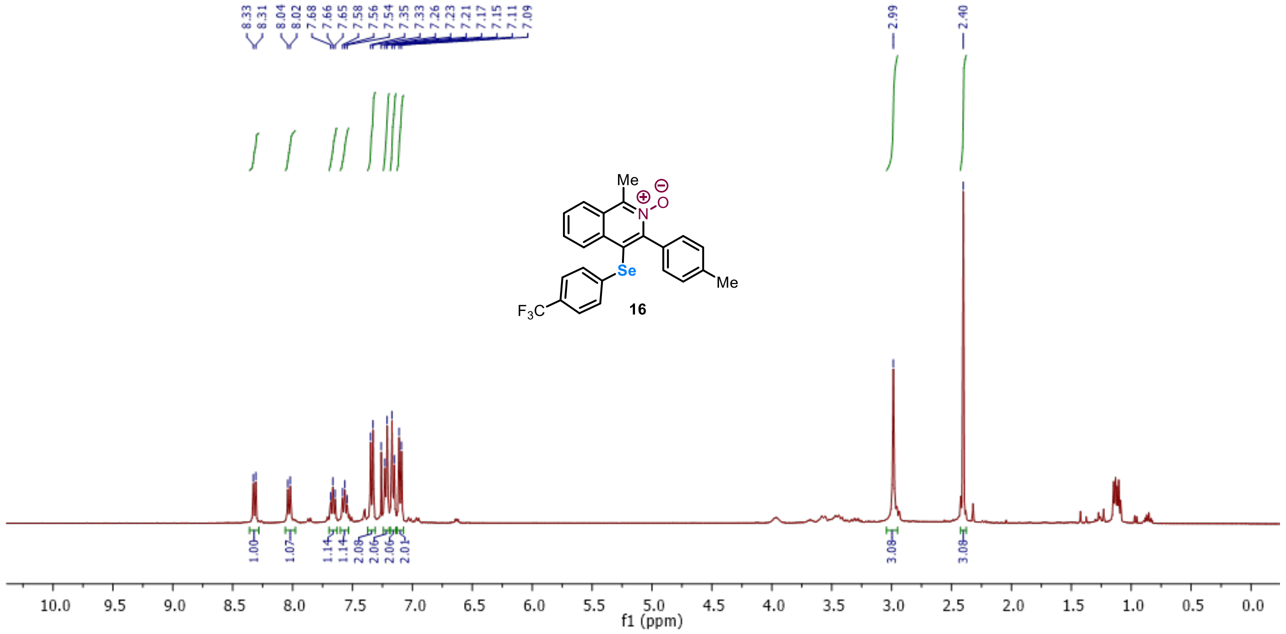

^{13}C NMR (101 MHz, $CDCl_3$)

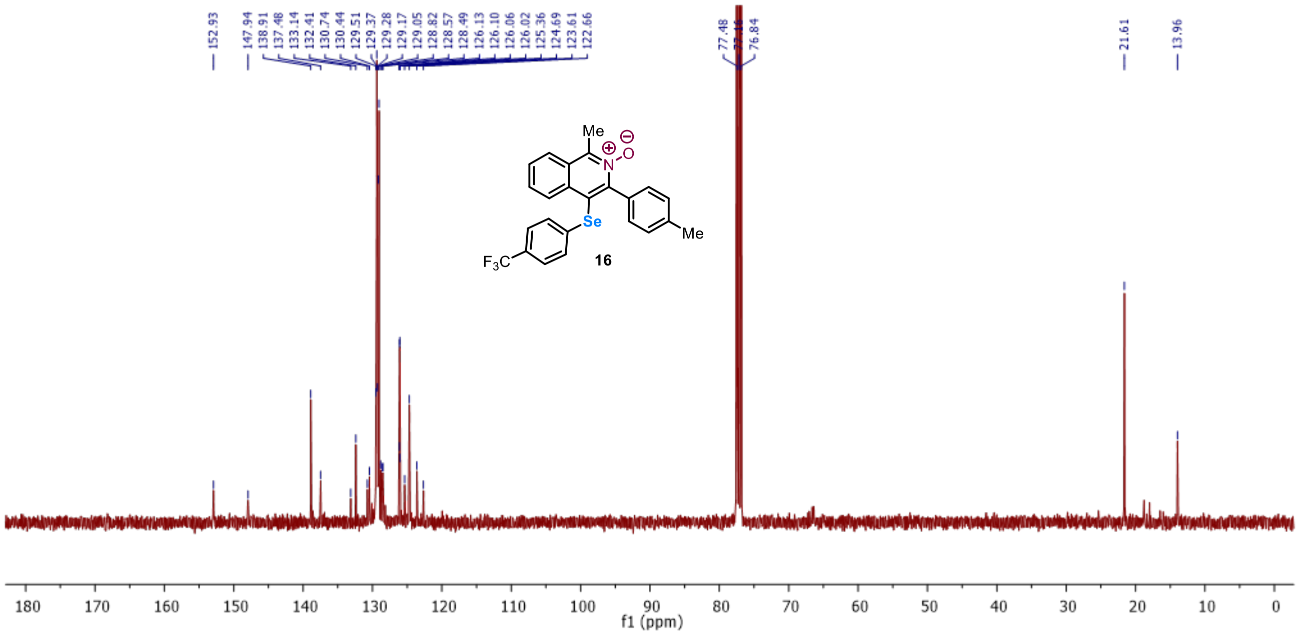

^{19}F NMR (376 MHz, $CDCl_3$)

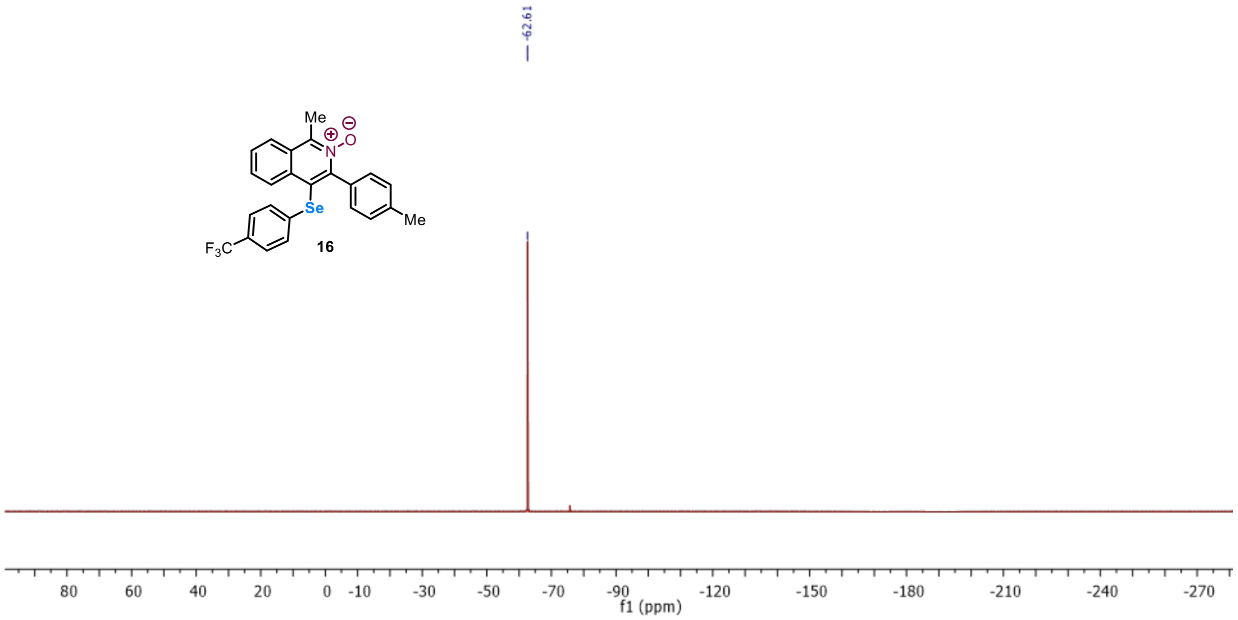

^{77}Se NMR (76 MHz, $CDCl_3$)

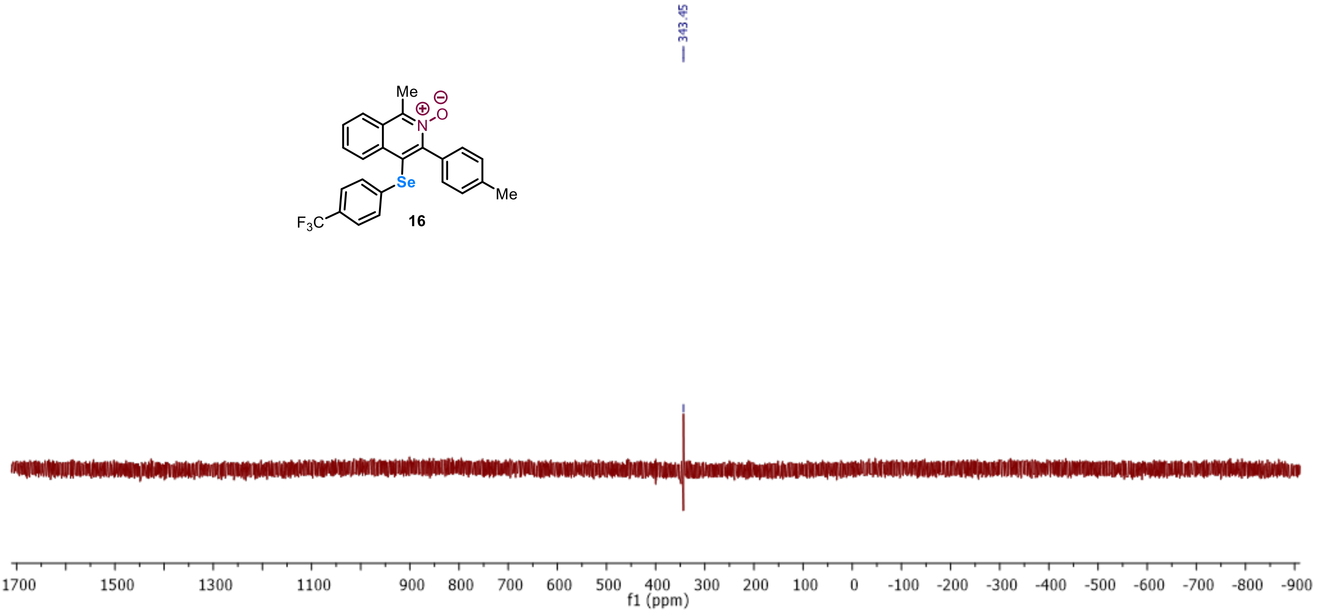

4-((3-Fluorophenyl)selanyl)-1-methyl-3-phenylisoquinoline 2-oxide (17)

^{1}H NMR (400 MHz, $CDCl_3$)

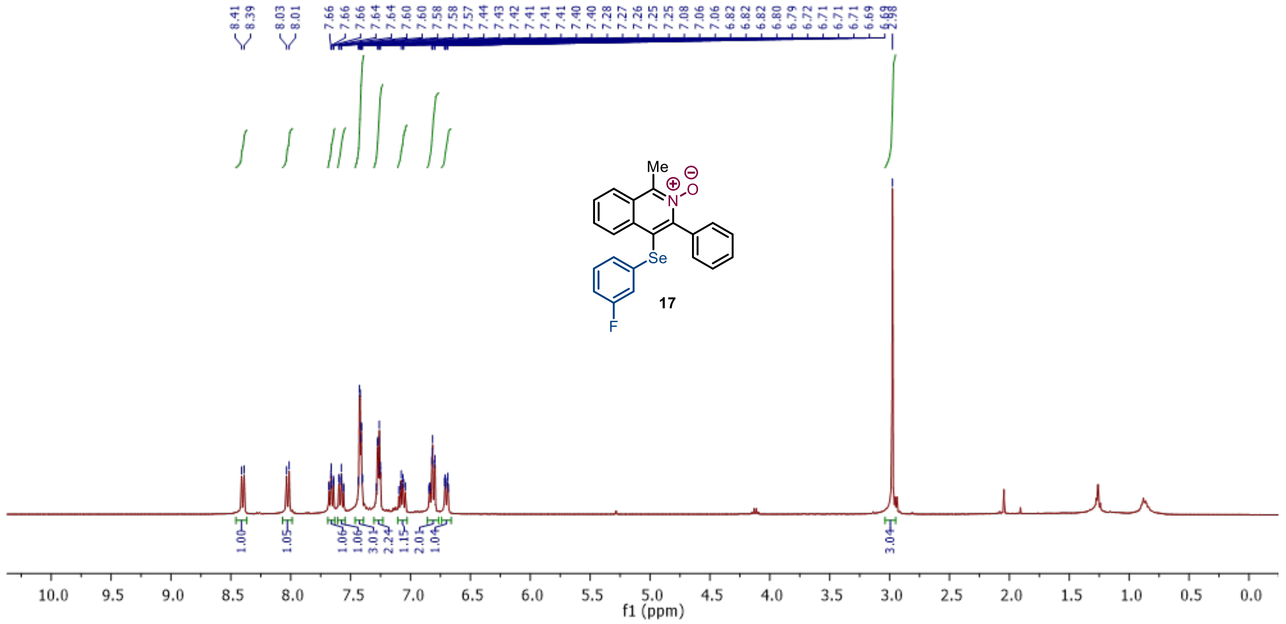

^{13}C NMR (101 MHz, $CDCl_3$)

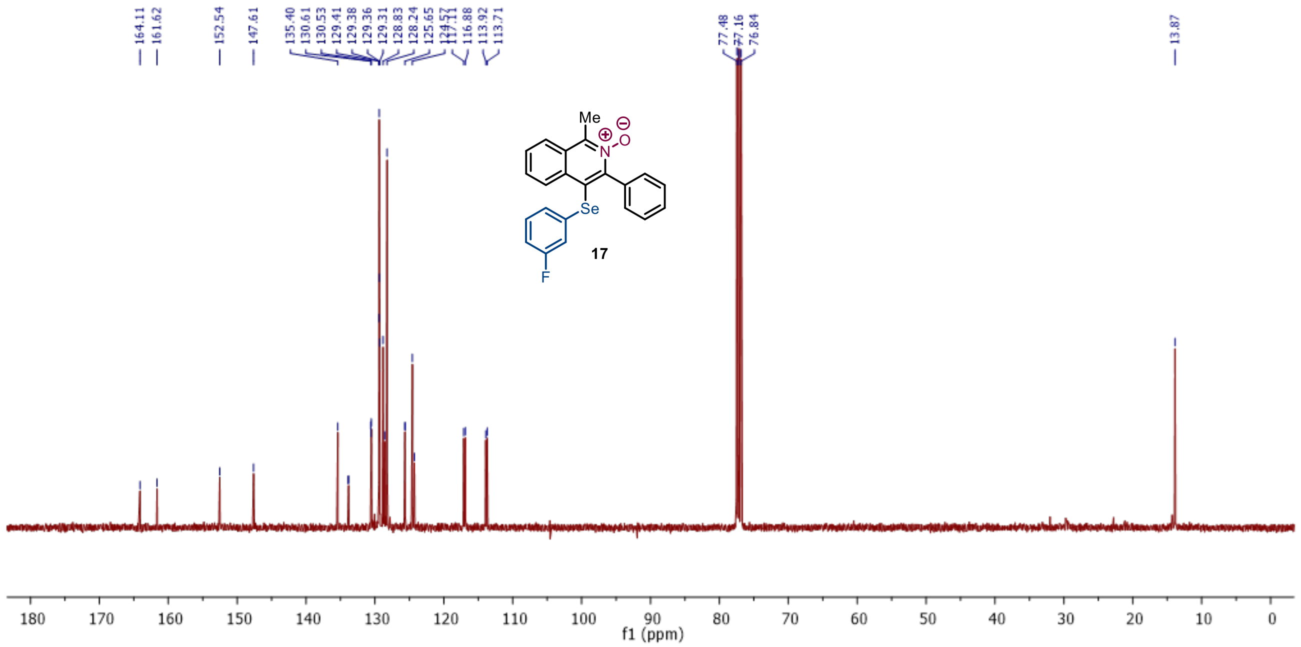

^{77}Se NMR (76 MHz, $CDCl_3$)

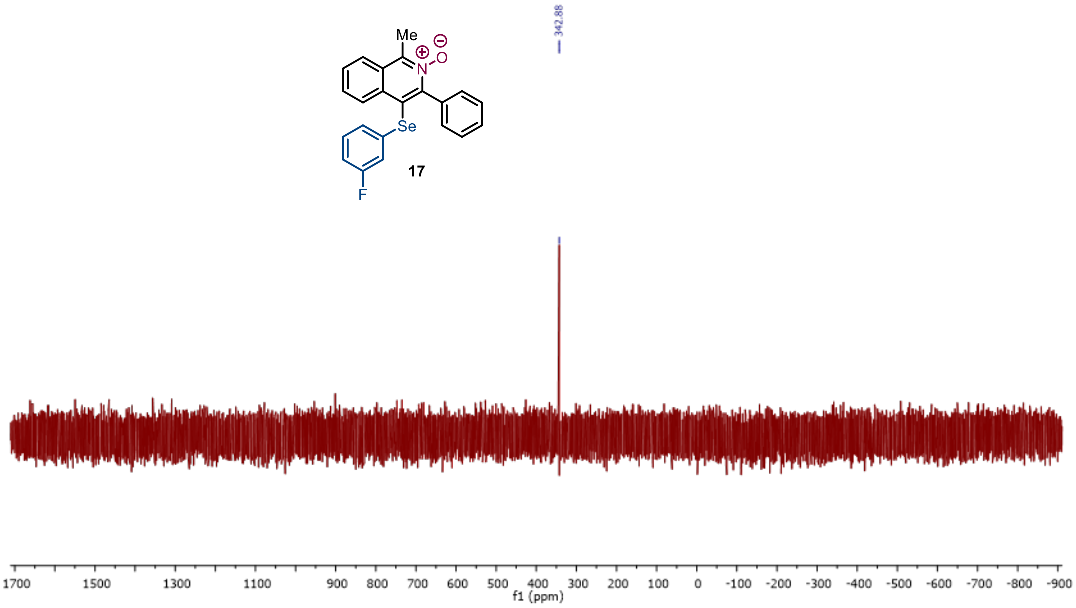

^{19}F NMR (376 MHz, $CDCl_3$)

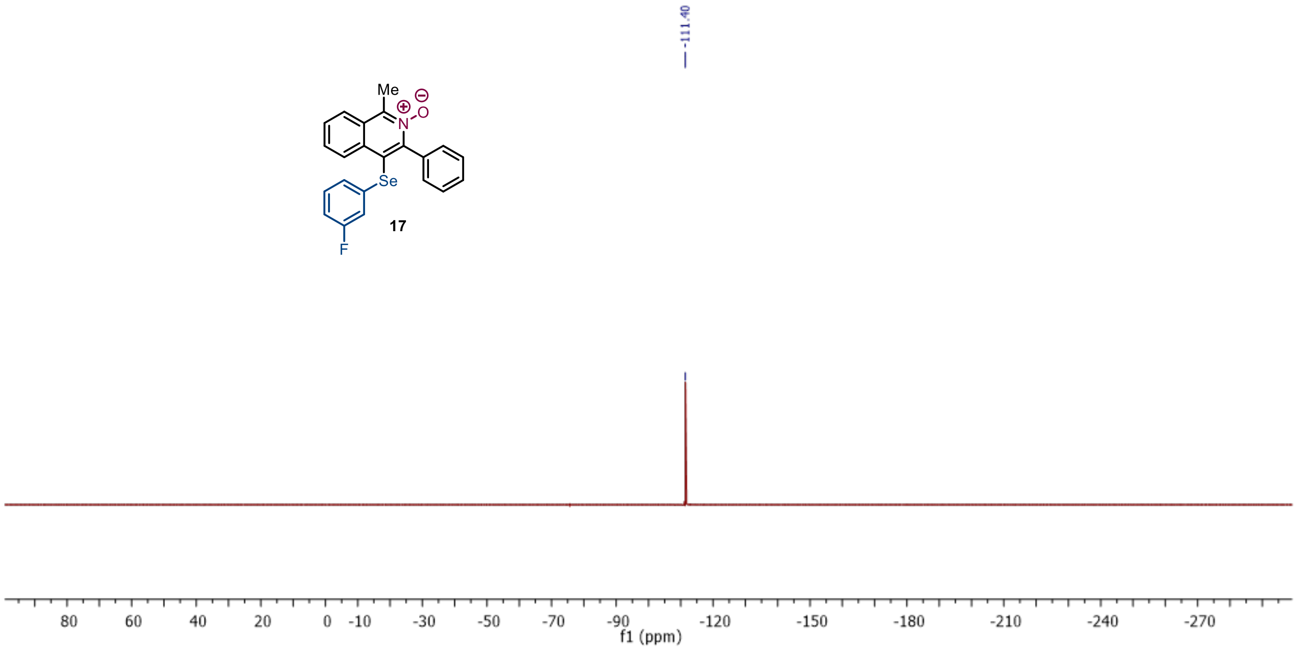

1-Methyl-4-(naphthalen-2-ylselanyl)-3-phenylisoquinoline 2-oxide (18)

^{1}H NMR (400 MHz, $CDCl_3$)

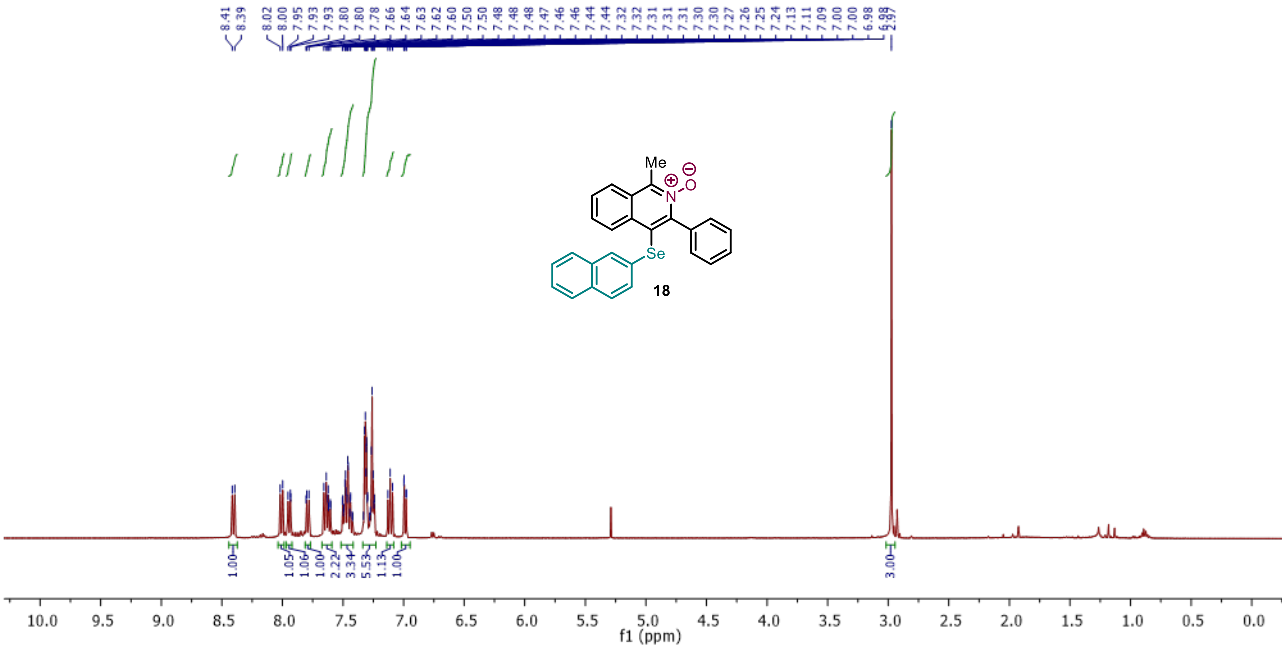

^{13}C NMR (101 MHz, $CDCl_3$)

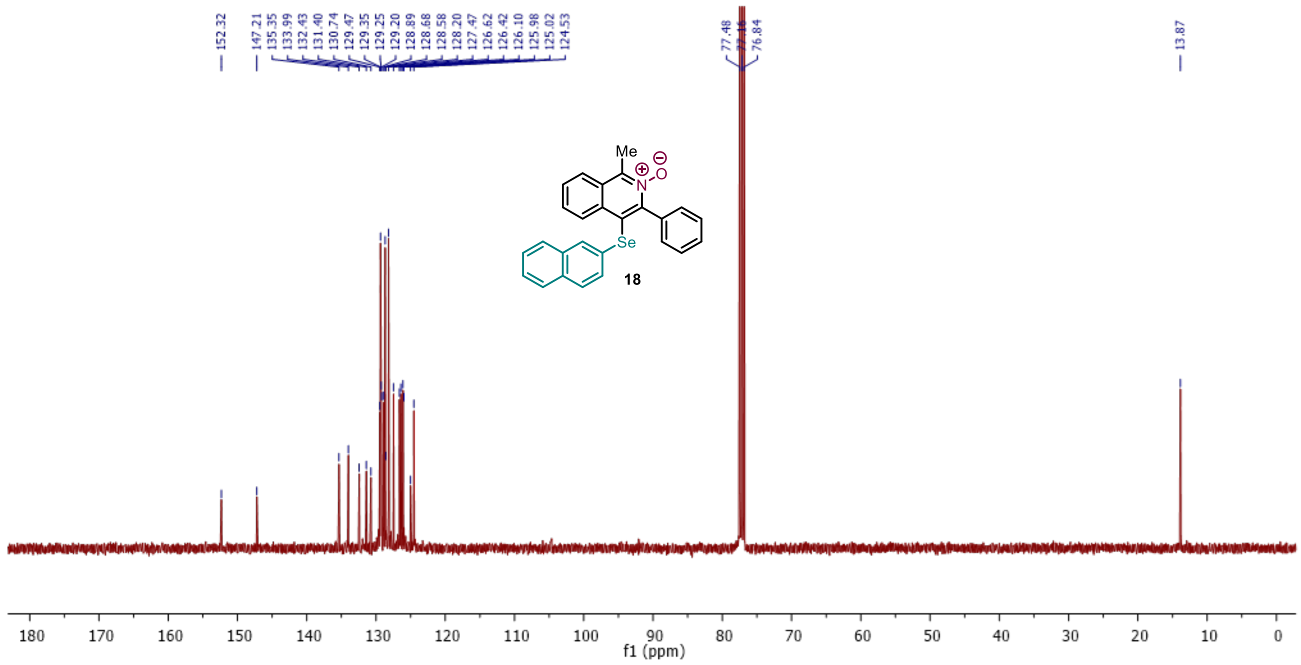

^{77}Se NMR (76 MHz, $CDCl_3$)

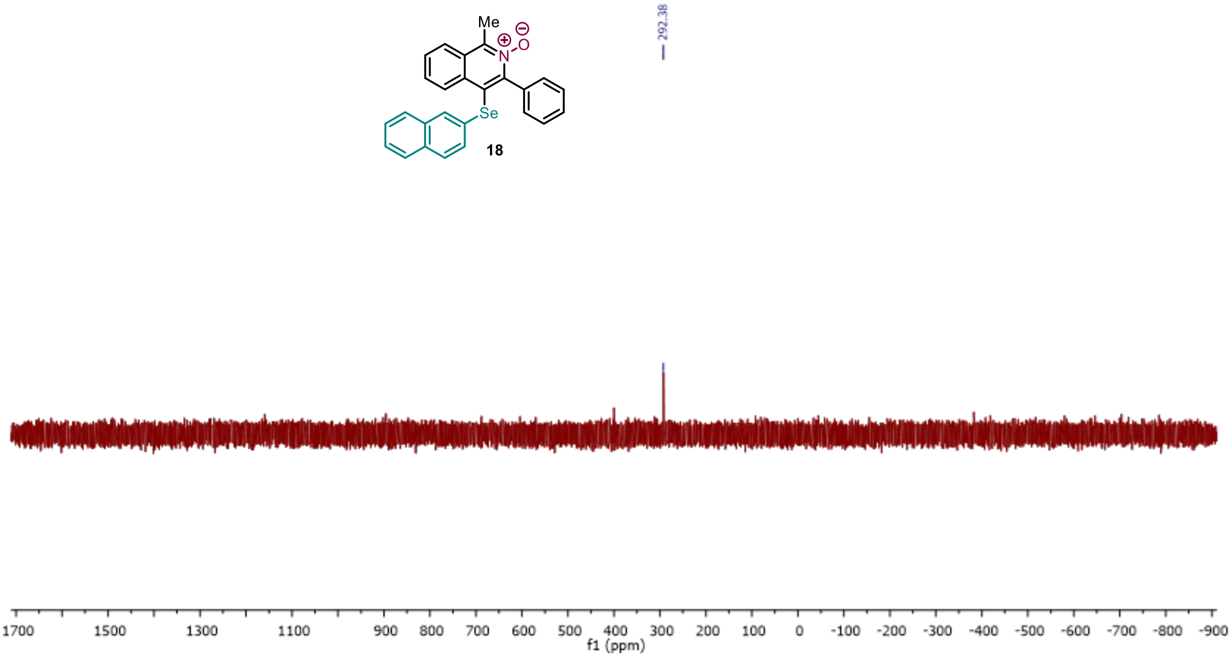

3-Phenyl-4-(phenylselanyl)isoquinoline 2-oxide (19)

^{1}H NMR (400 MHz, $CDCl_3$)

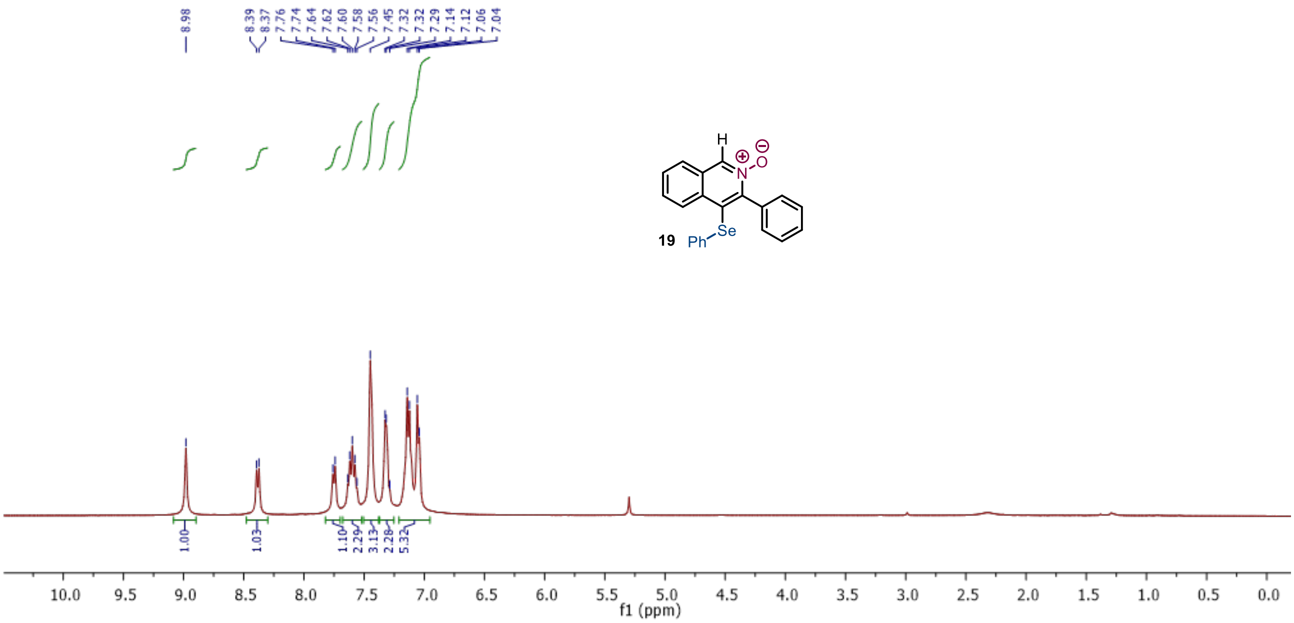

^{13}C NMR (101 MHz, $CDCl_3$)

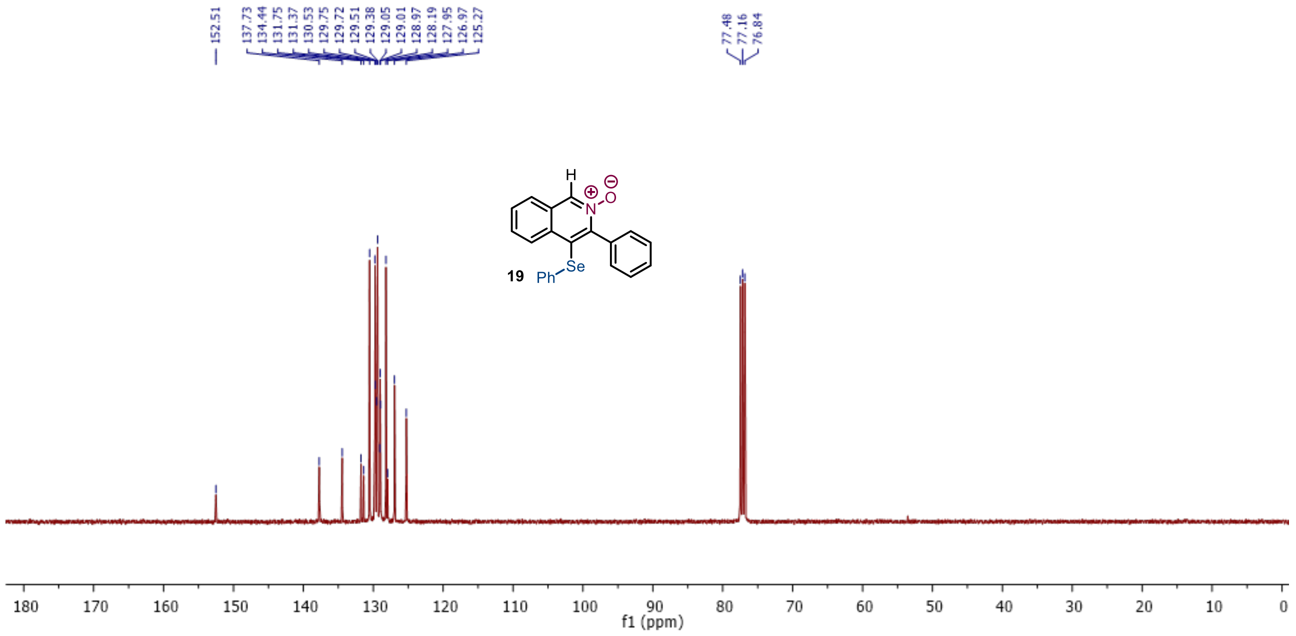

3-(4-Methoxyphenyl)-4-(phenylselanyl)isoquinoline 2-oxide(20)

^{1}H NMR (400 MHz, CDCl$_3$)

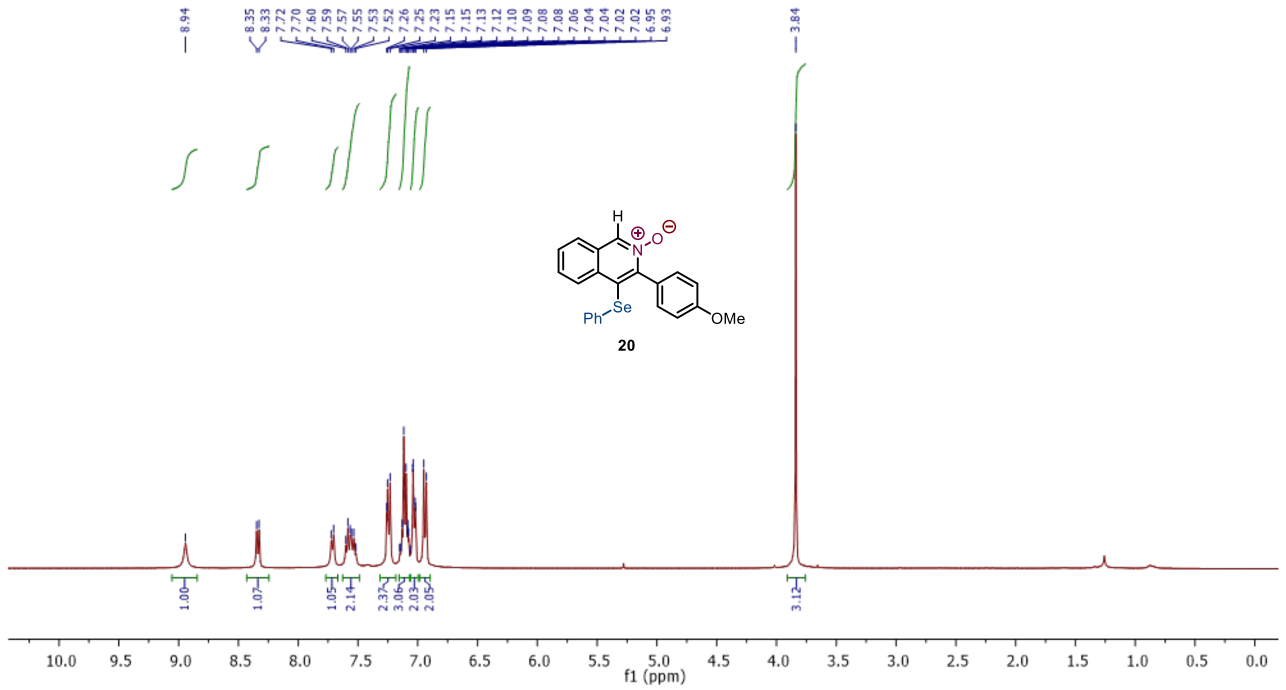

^{13}C NMR (101 MHz, CDCl$_3$)

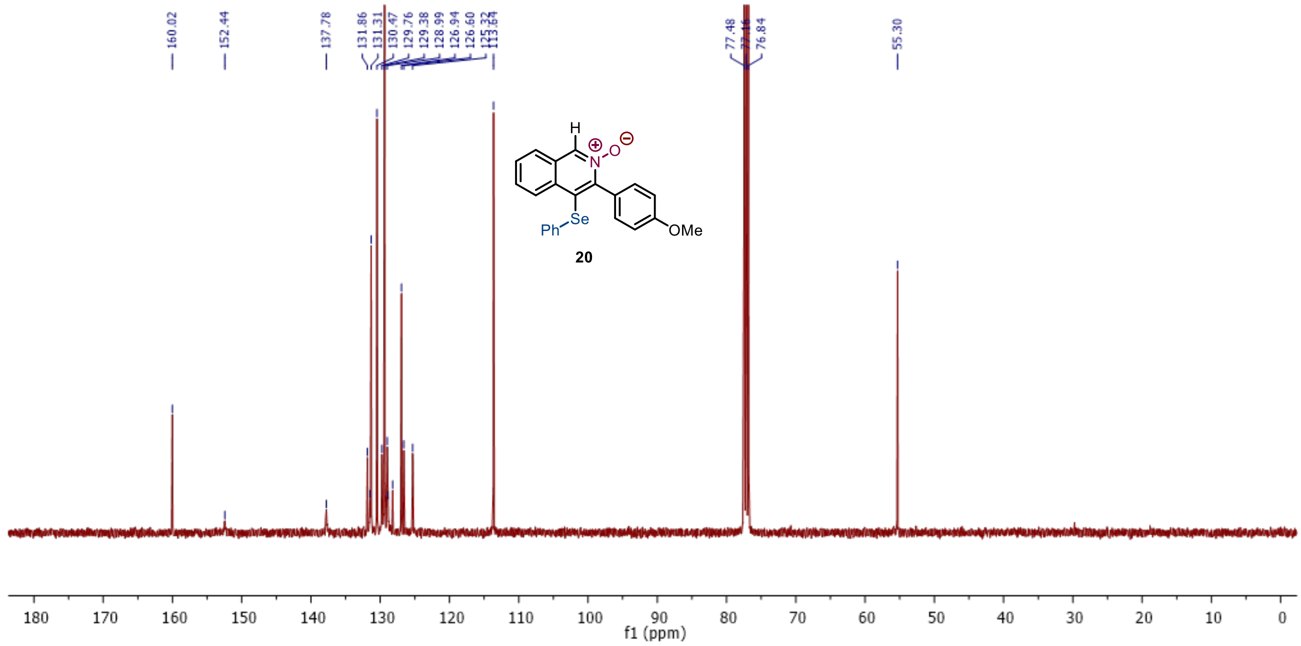

NMR (76 MHz, $CDCl_3$)

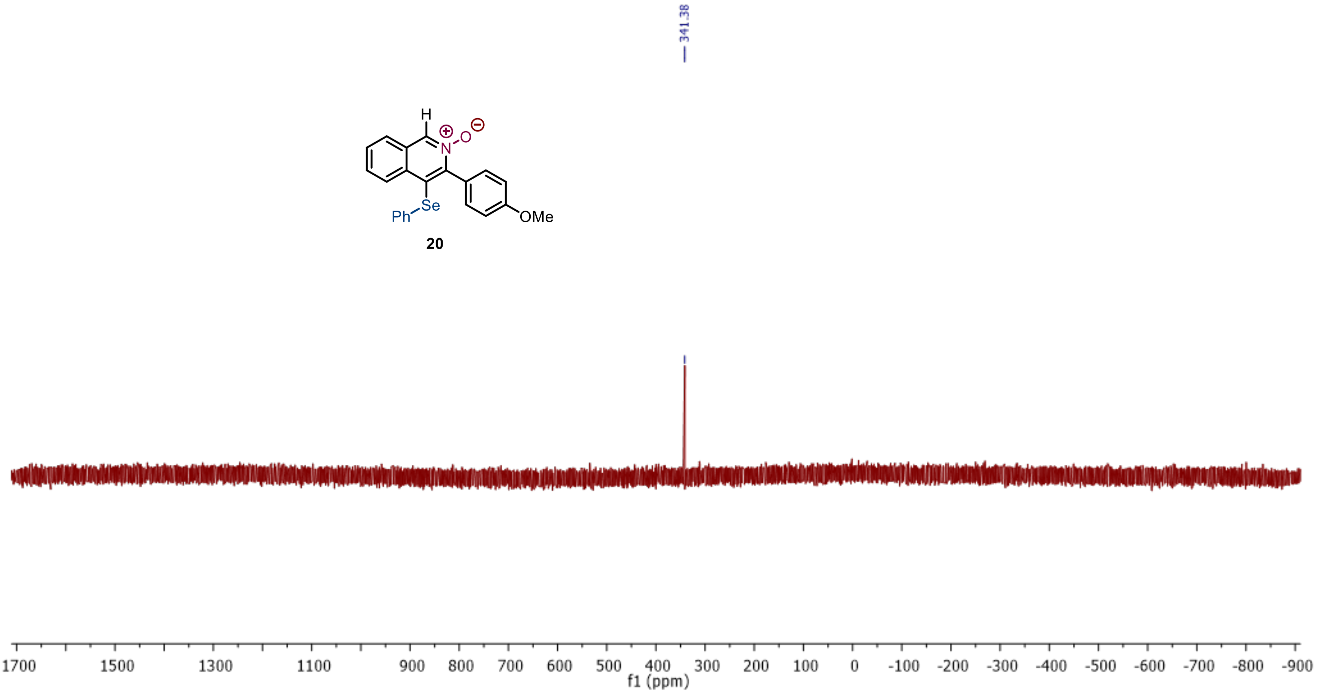

4-(Phenylselanyl)-3-(thiophen-2-yl)isoquinoline 2-oxide (21)

^1H NMR (400 MHz, $CDCl_3$)

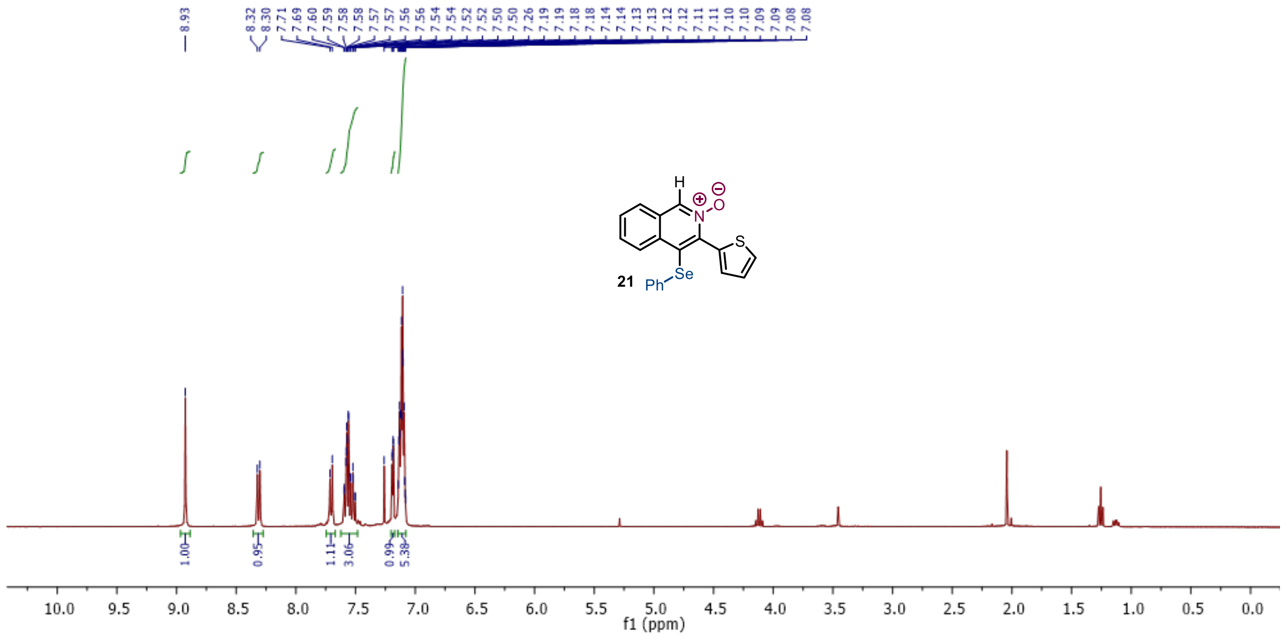

^{13}C NMR (101 MHz, CDCl$_3$)

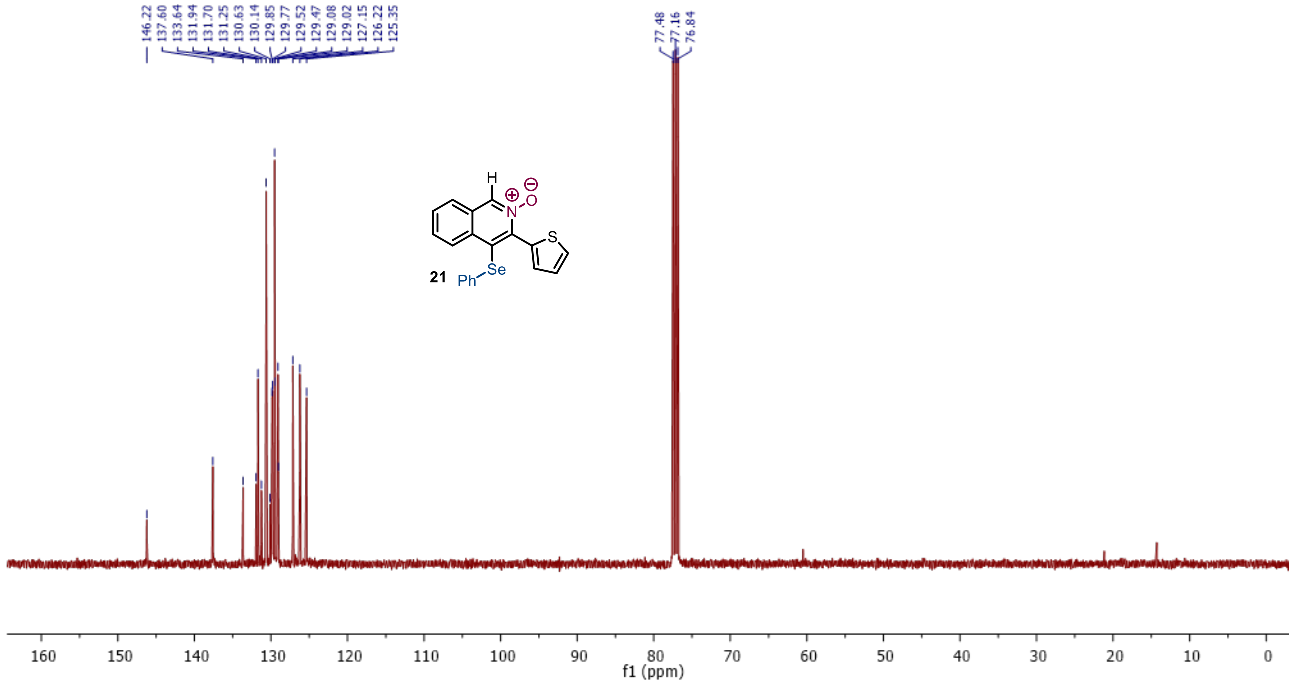

^{77}Se NMR (76 MHz, $CDCl_3$)

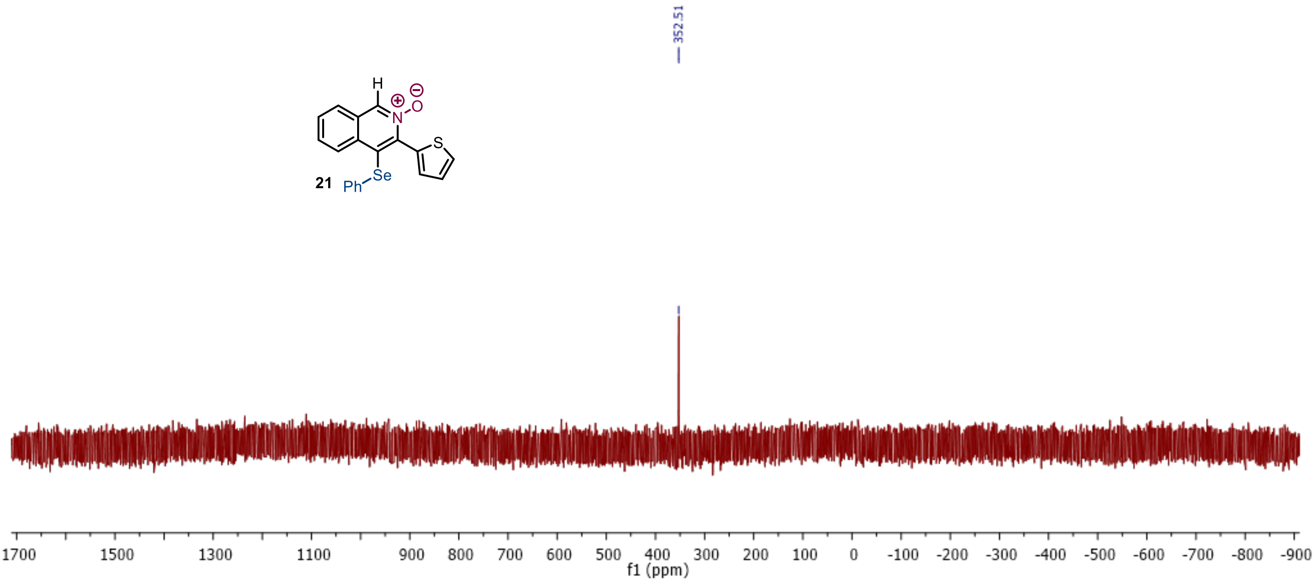

4-(Benzylselanyl)-3-(thiophen-2-yl)isoquinoline 2-oxide (22):

^{1}H NMR (400 MHz, $CDCl_3$)

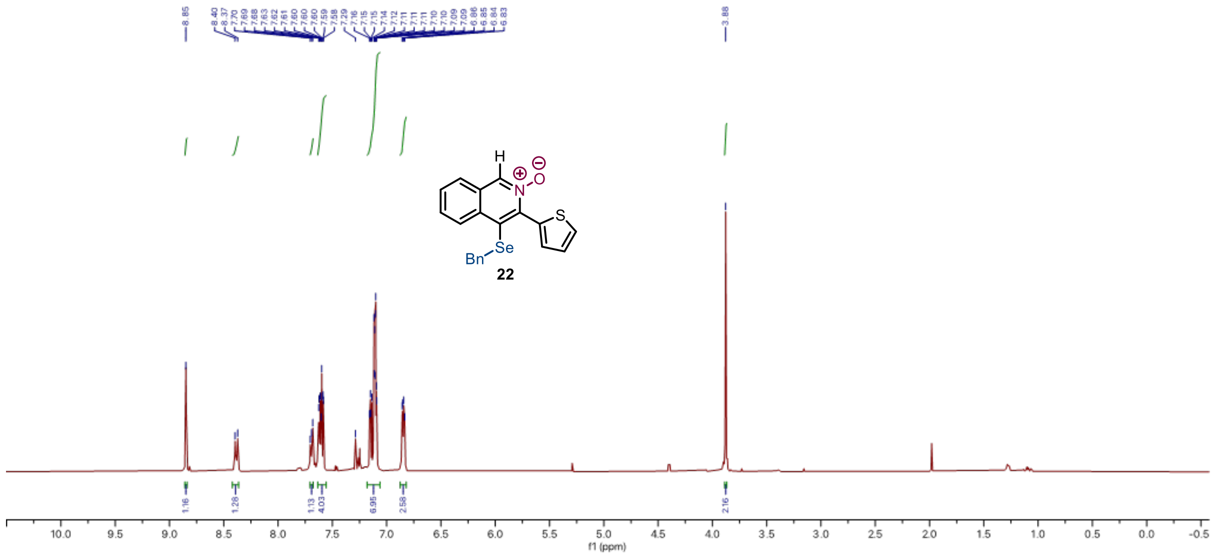

^{13}C NMR (101 MHz, $CDCl_3$)

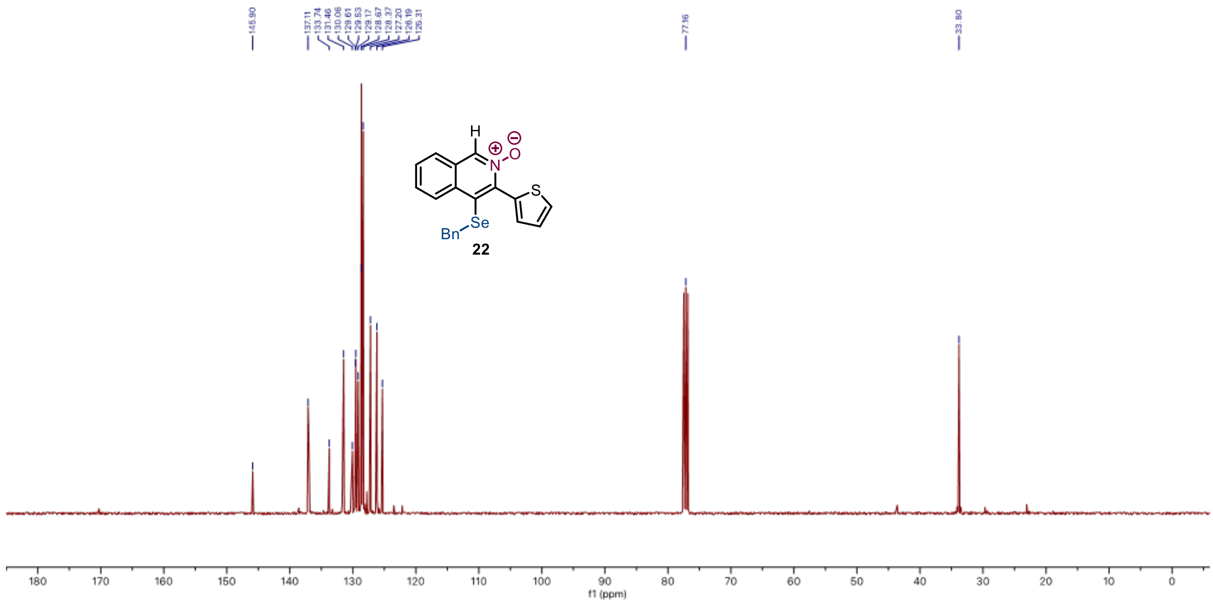

3-Phenyl-4-(p-tolylselanyl)isoquinoline 2-oxide (23)

^{1}H NMR (400 MHz, $CDCl_3$)

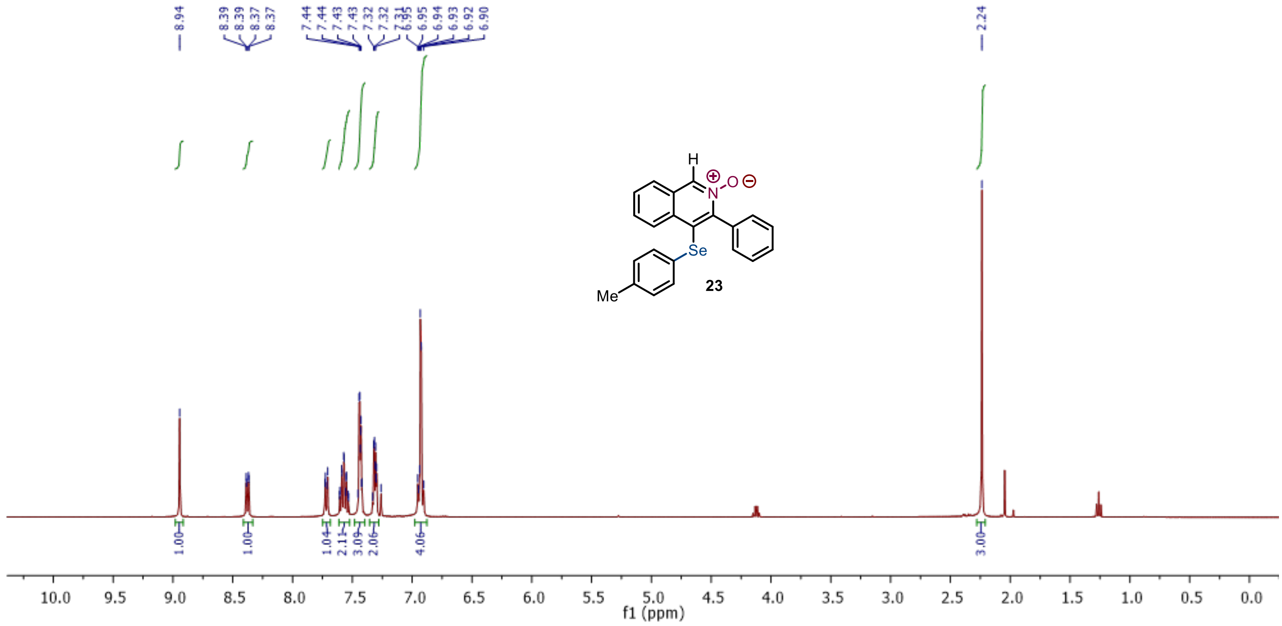

^{13}C NMR (101 MHz, $CDCl_3$)

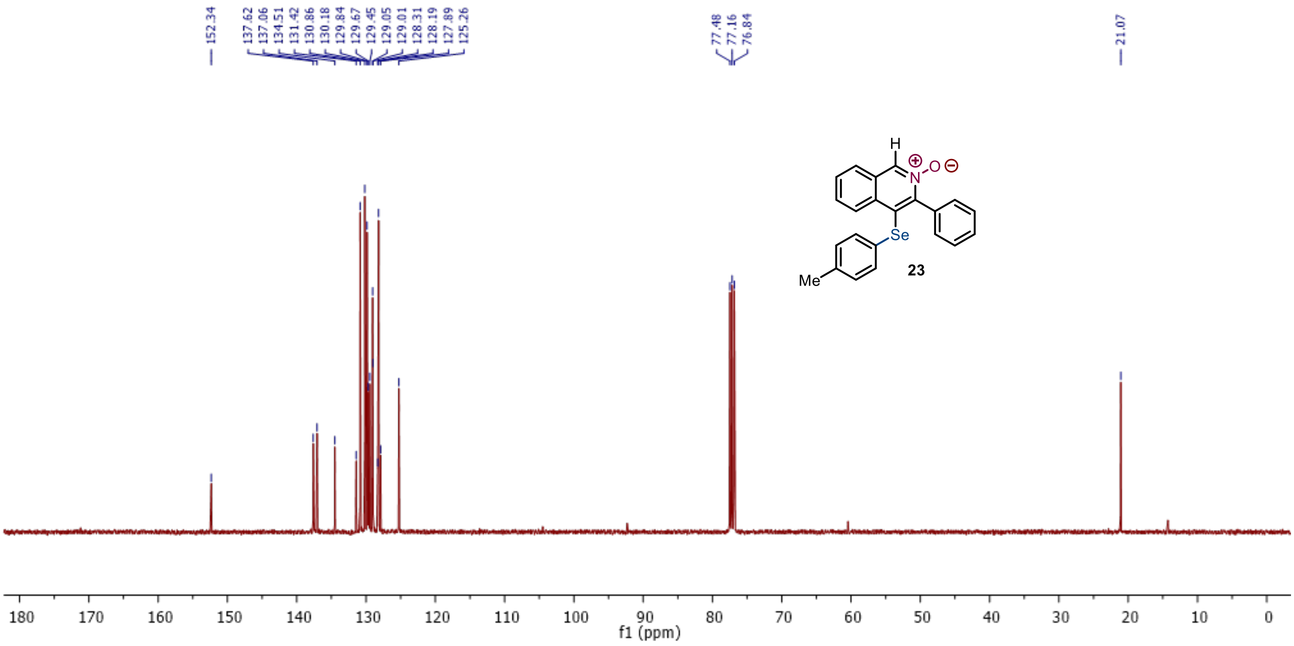

4-(Phenylselanyl)isoquinoline 2-oxide (24)

^{1}H NMR (400 MHz, CDCl$_3$)

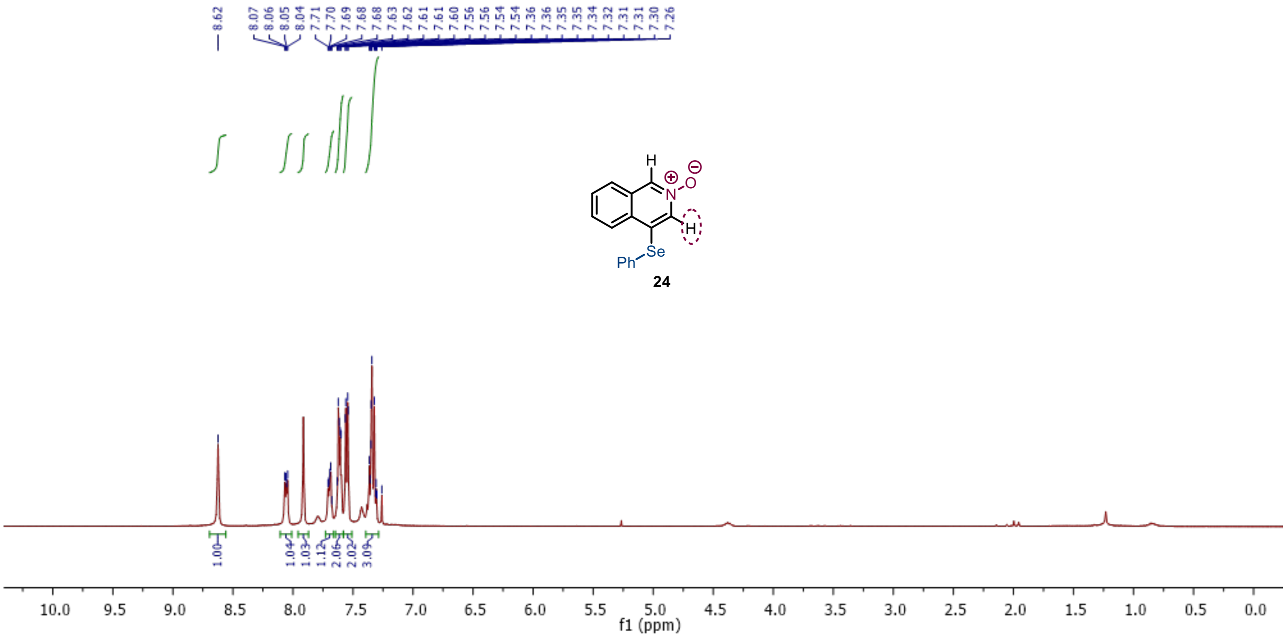

^{13}C NMR (101 MHz, CDCl$_3$)

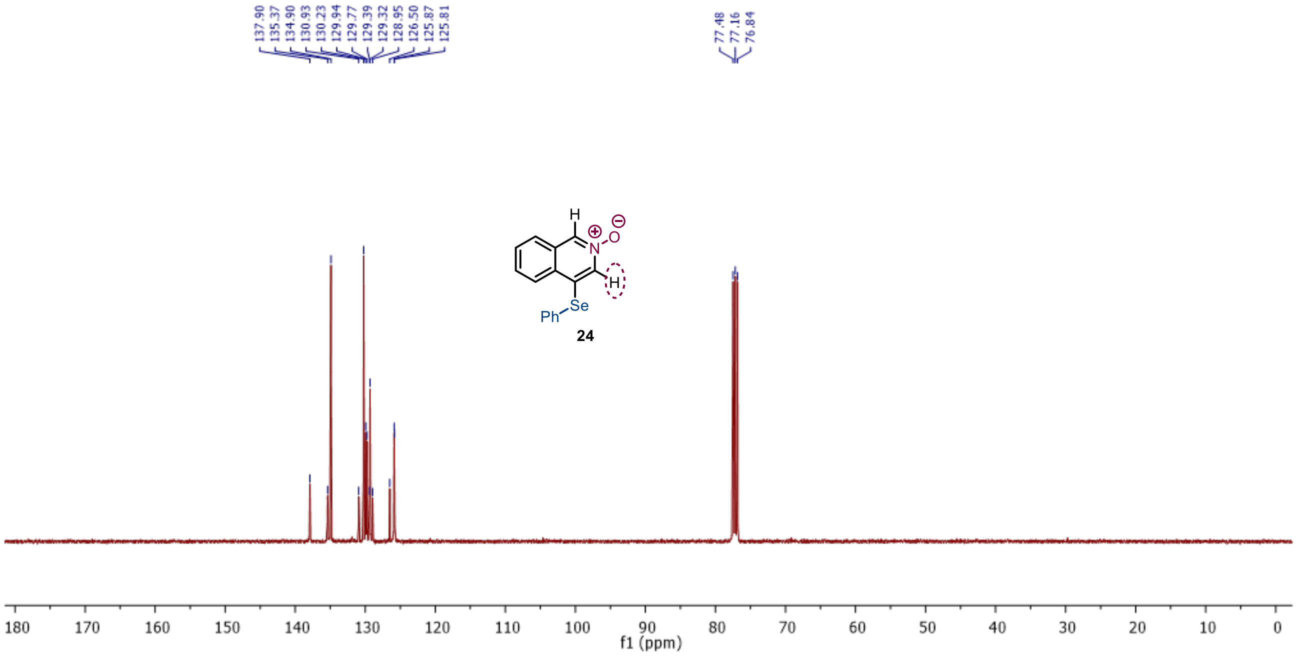

^{77}Se NMR (76 MHz, $CDCl_3$)

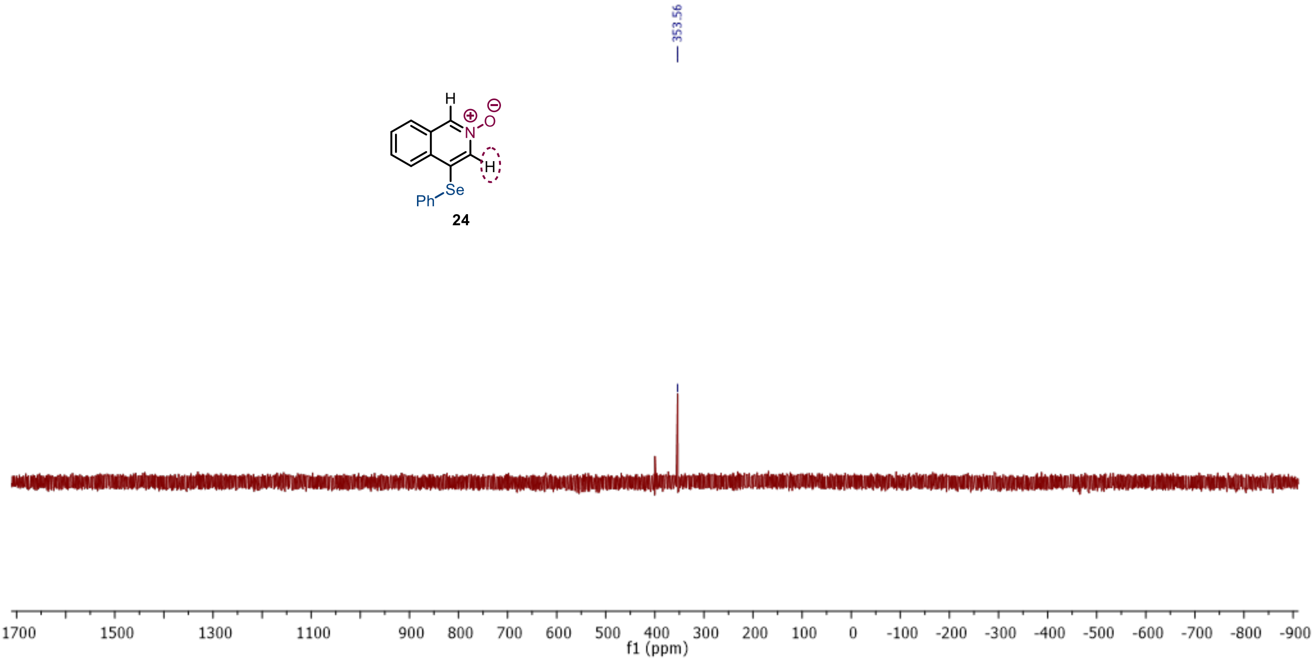

6,7-Dimethoxy-3-phenyl-4-(phenylselanyl)isoquinoline 2-oxide (25):

^{1}H NMR (400 MHz, $CDCl_3$)

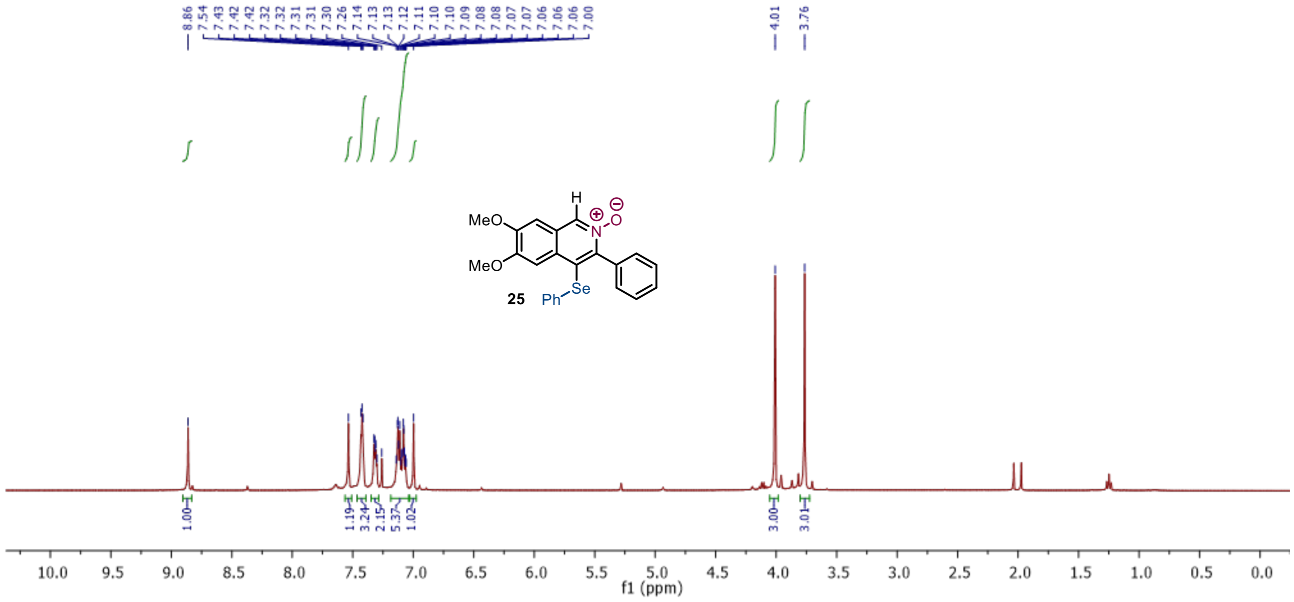

^{13}C NMR (101 MHz, $CDCl_3$)

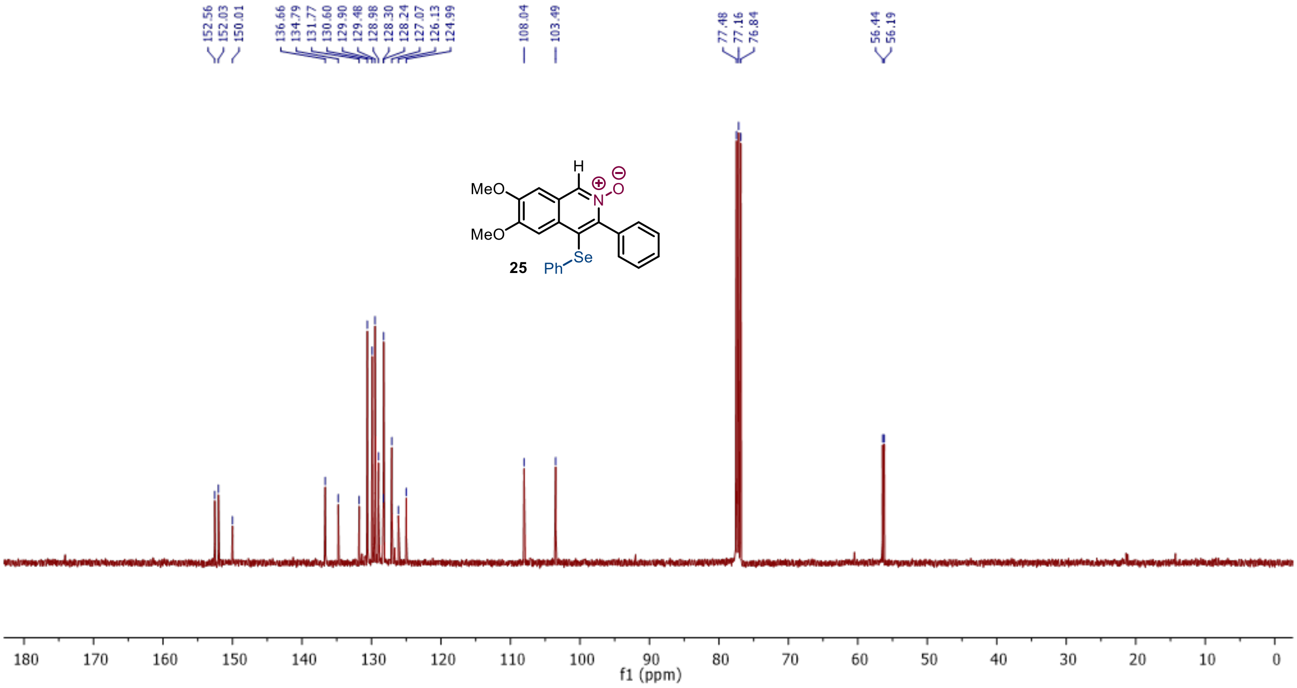

^{77}Se NMR (76 MHz, $CDCl_3$)

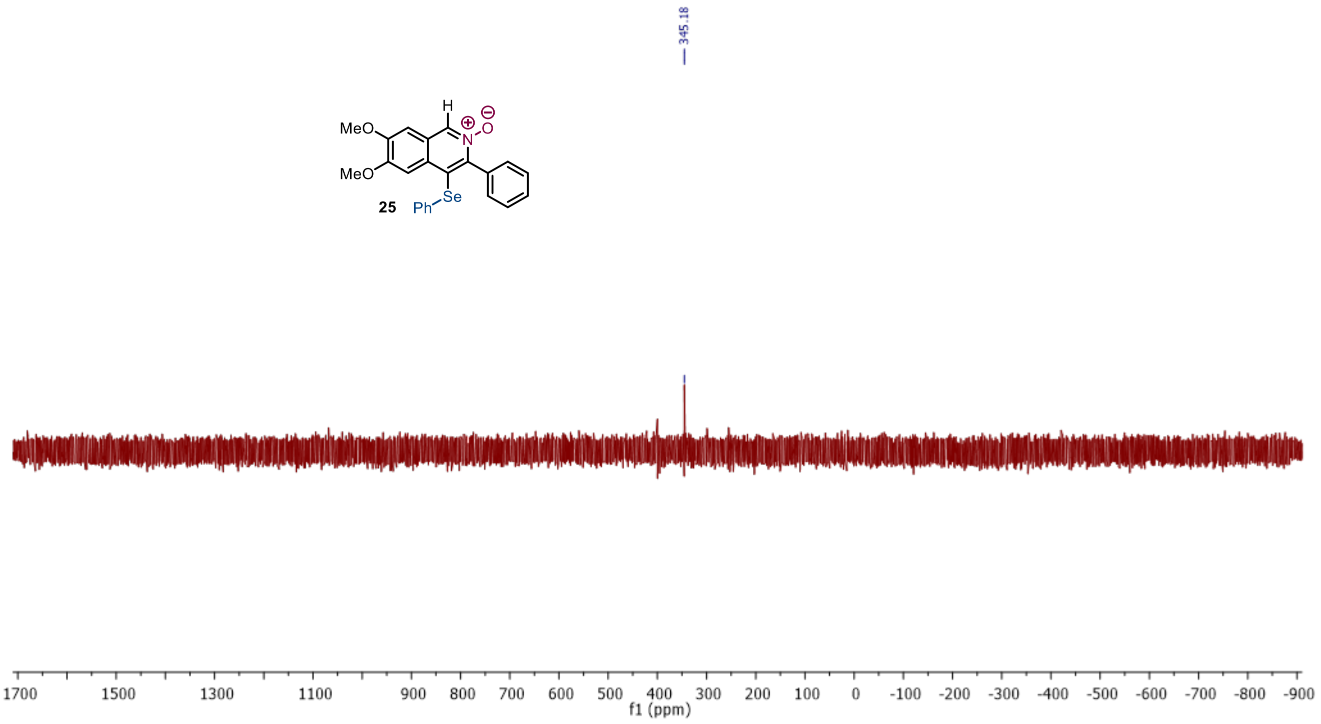

3-Phenyl-4-(phenyltellanyl)isoquinoline 2-oxide (26)

Detected a non-isolable N-oxide product without a tellurium group, along with the desired product **26**.

^{1}H NMR (400 MHz, $CDCl_3$)

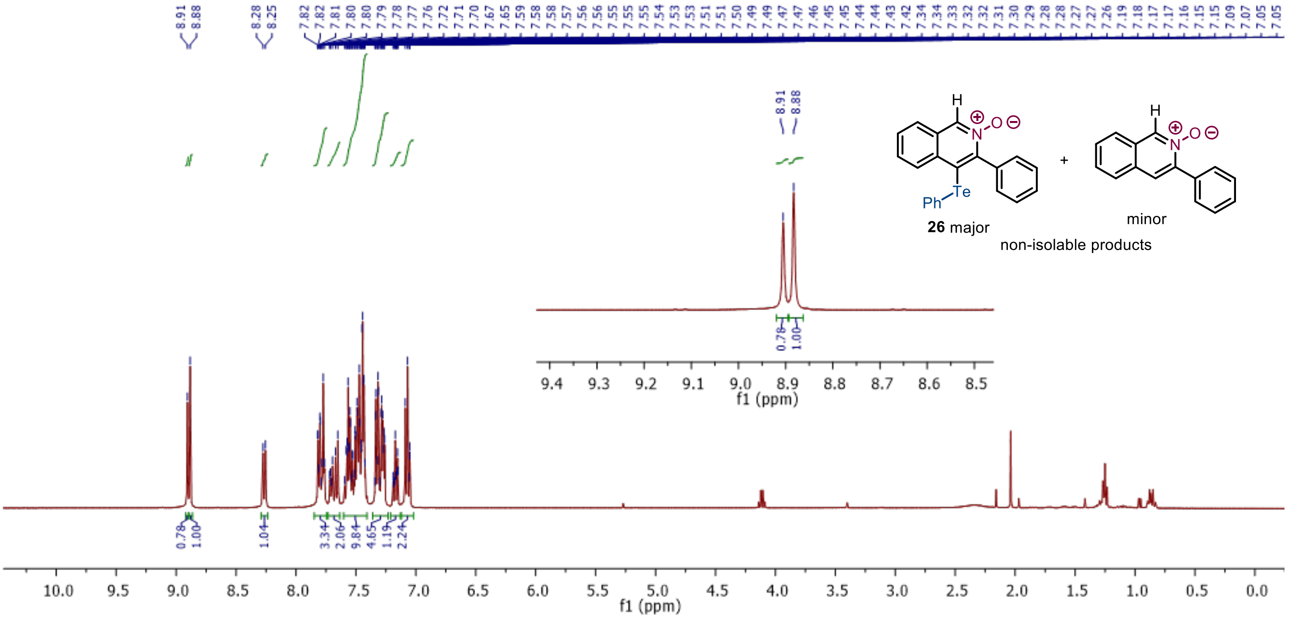

^{13}C NMR (101 MHz, $CDCl_3$)

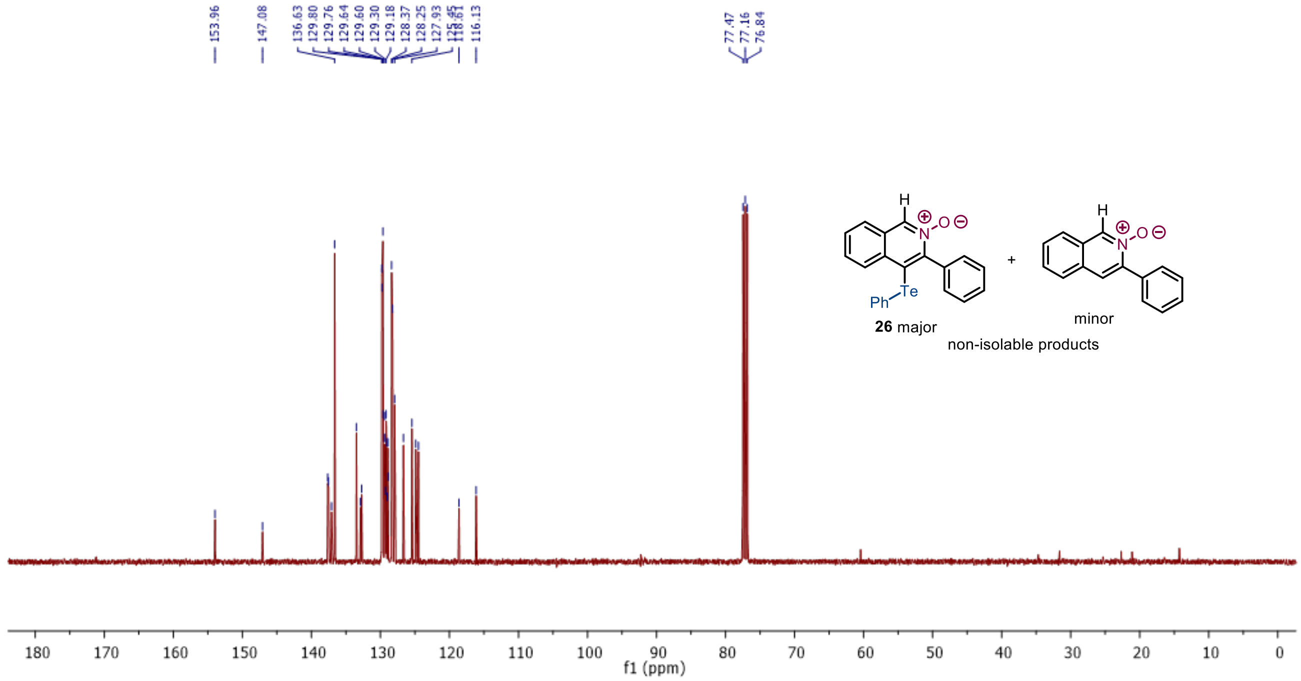

1-Methyl-3-phenyl-4-(phenyltellanyl)isoquinoline 2-oxide (27):

Detected a trace amount of non-isolable N-oxide product without a tellurium substituent.

^{1}H NMR (400 MHz, $CDCl_3$)

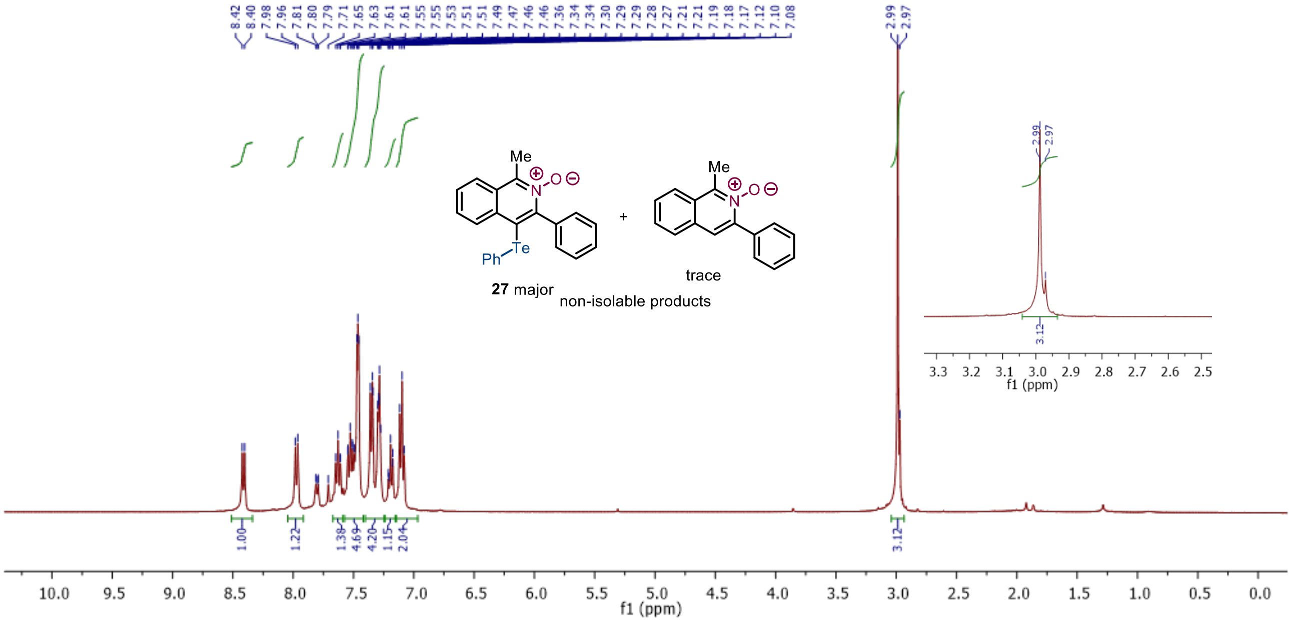

^{13}C NMR (101 MHz, $CDCl_3$)

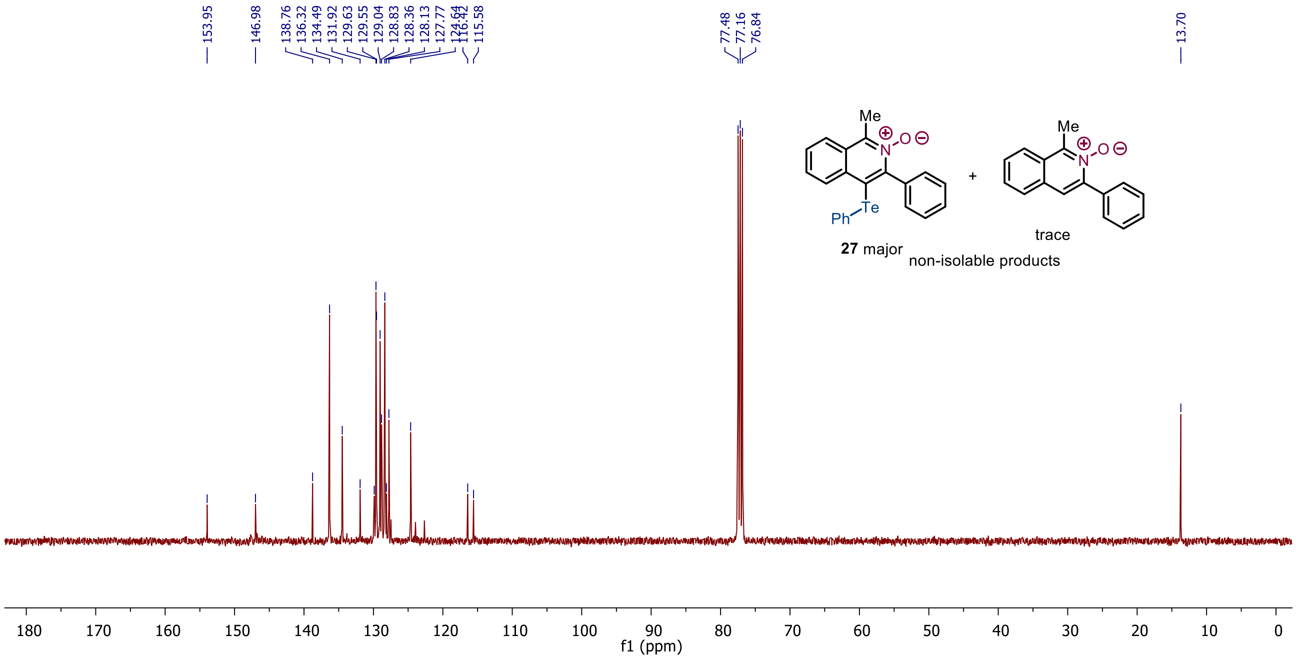

1-Methyl-3-phenyl-4-(phenylselanyl)isoquinolin-2-ium-2-yl)(tosyl)amide (28)

^{1}H NMR (400 MHz, $CDCl_3$)

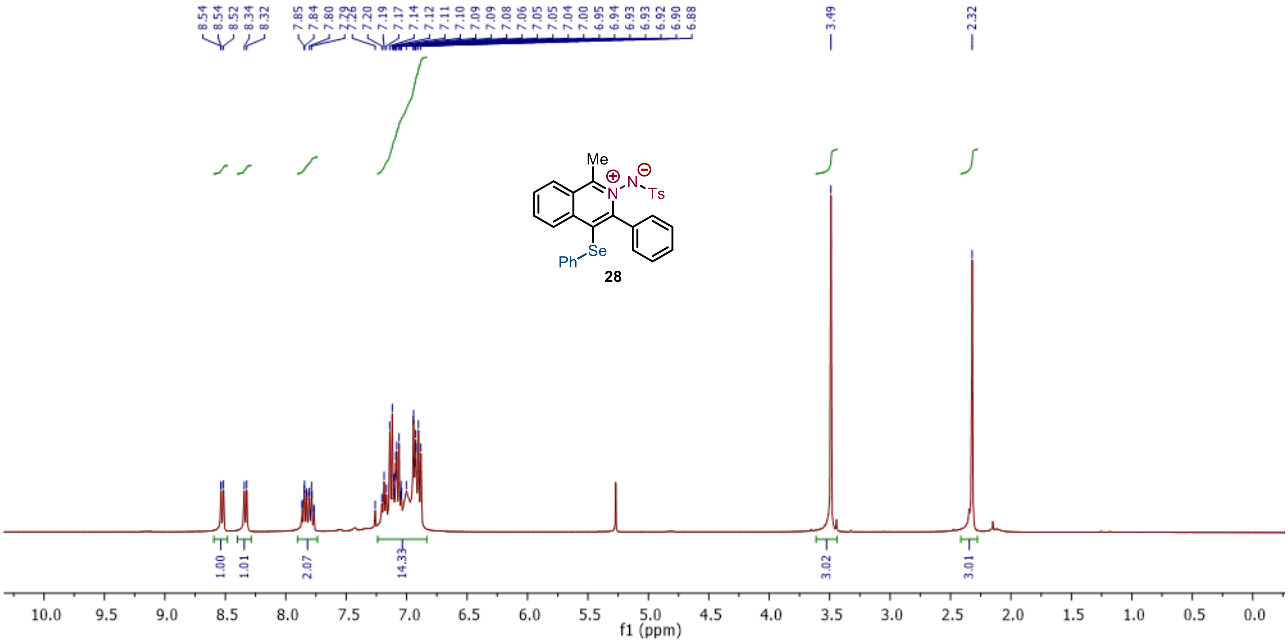

^{13}C NMR (101 MHz, $CDCl_3$)

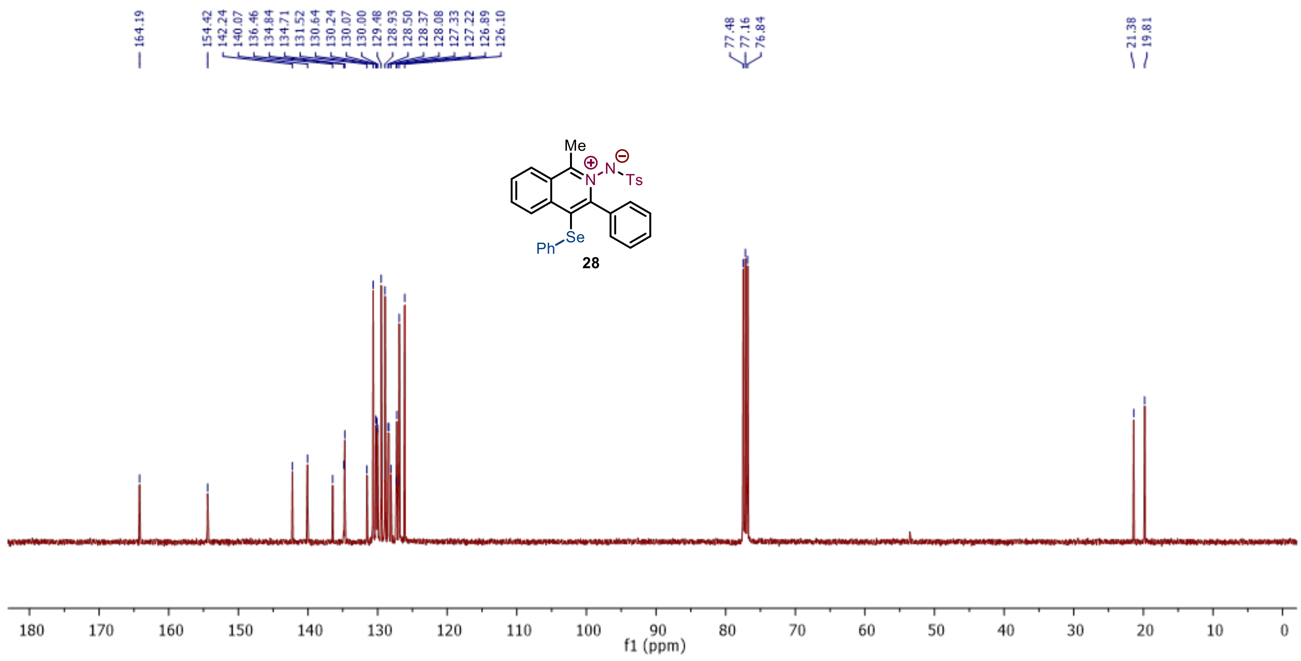

^{77}Se NMR (76 MHz, $CDCl_3$)

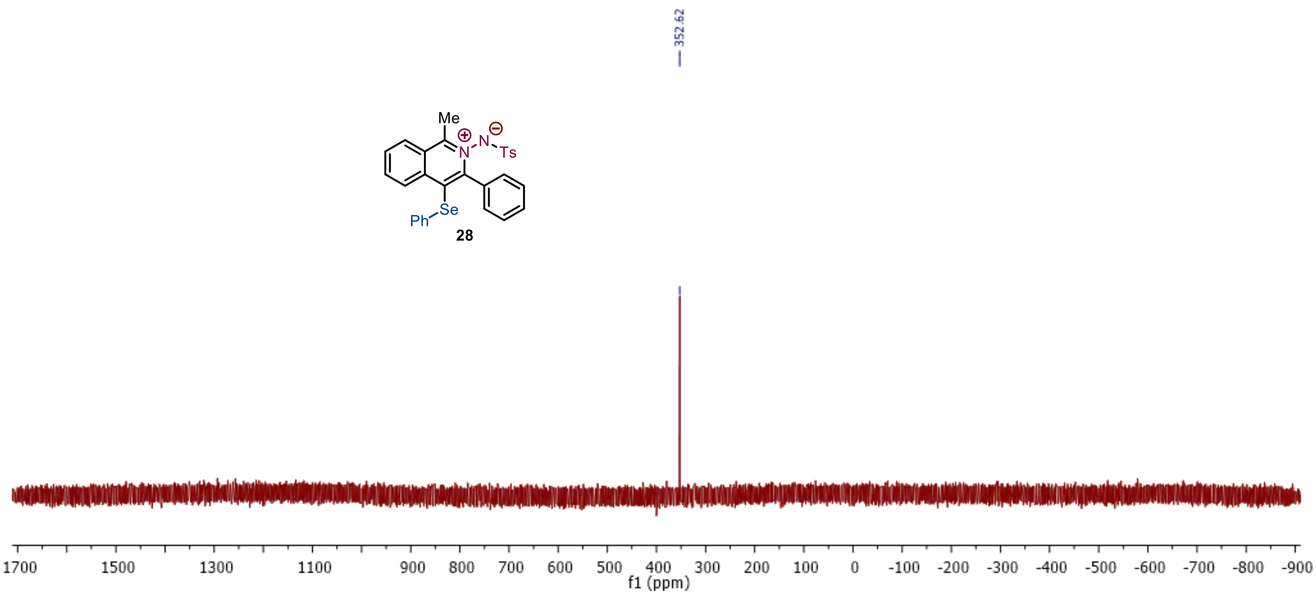

(3-(4-Methoxyphenyl)-1-methyl-4-(phenylselanyl)isoquinolin-2-ium-2-yl)(tosyl)amide (29)

^{1}H NMR (500 MHz, $CDCl_3$)

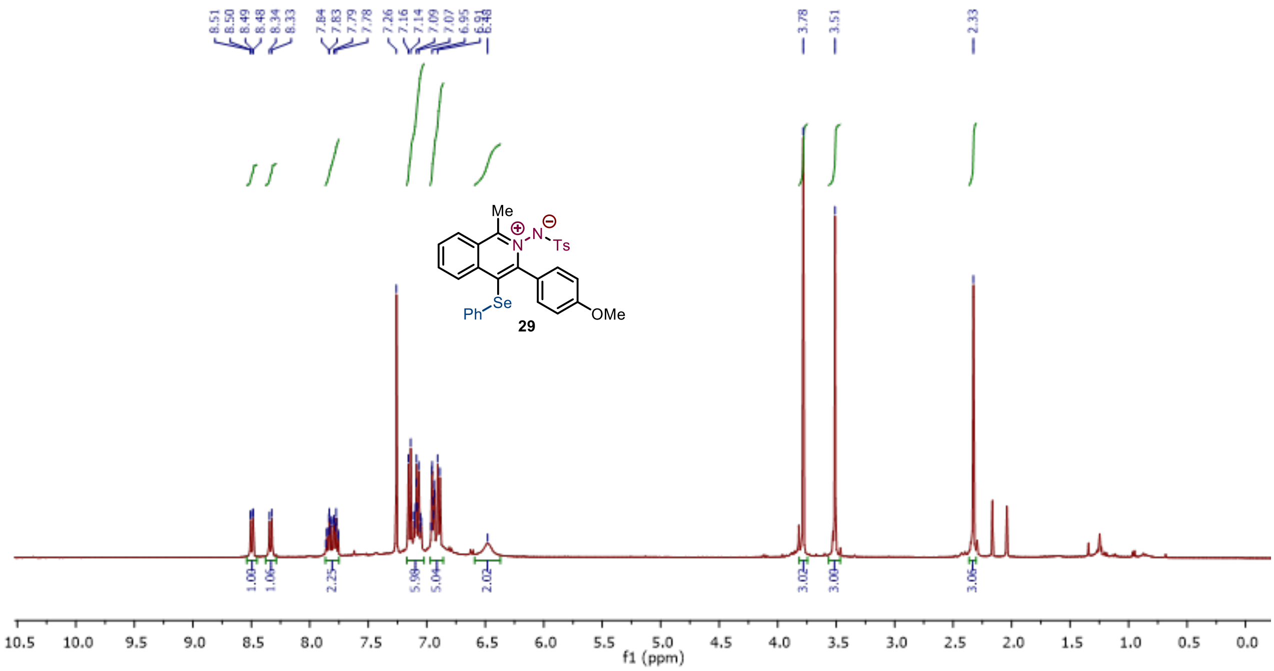

^{13}C NMR (126 MHz, $CDCl_3$)

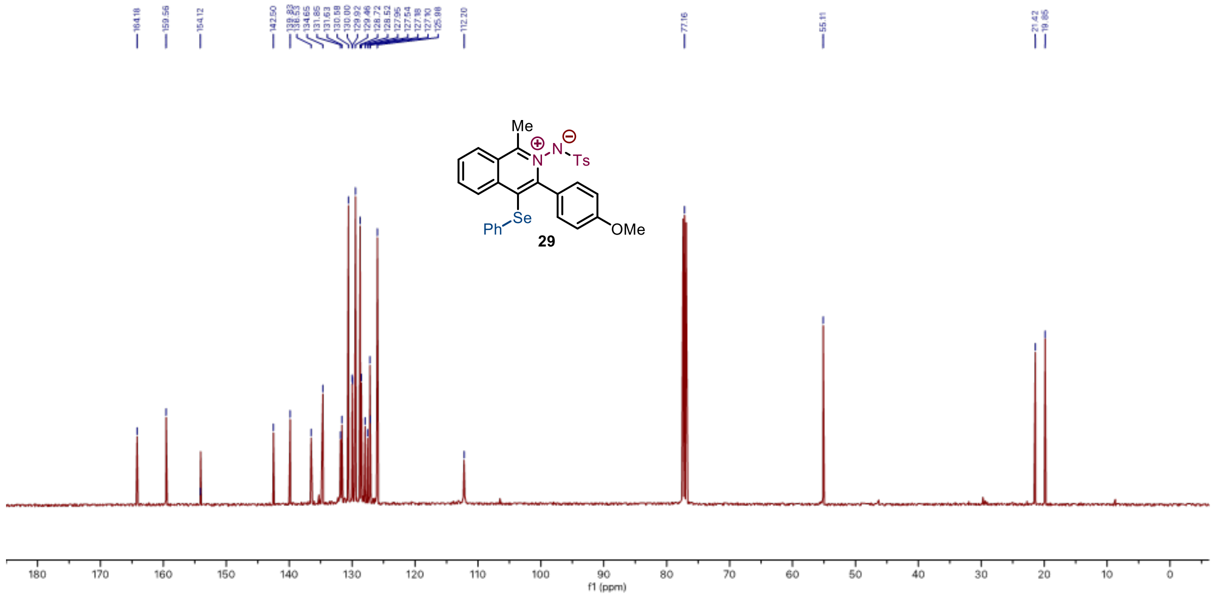

^{77}Se NMR (76 MHz, $CDCl_3$)

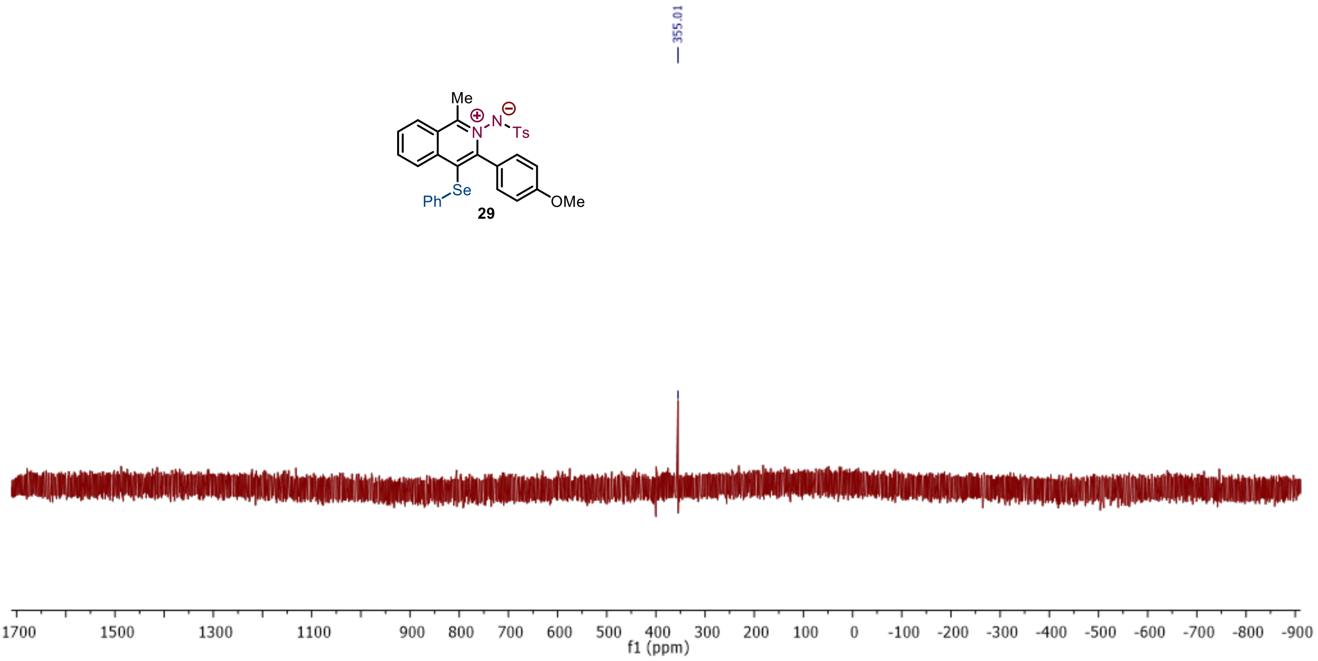

(3-Phenyl-4-(phenylselanyl)isoquinolin-2-ium-2-yl)(tosyl)amide (30)

^{1}H NMR (400 MHz, $CDCl_3$)

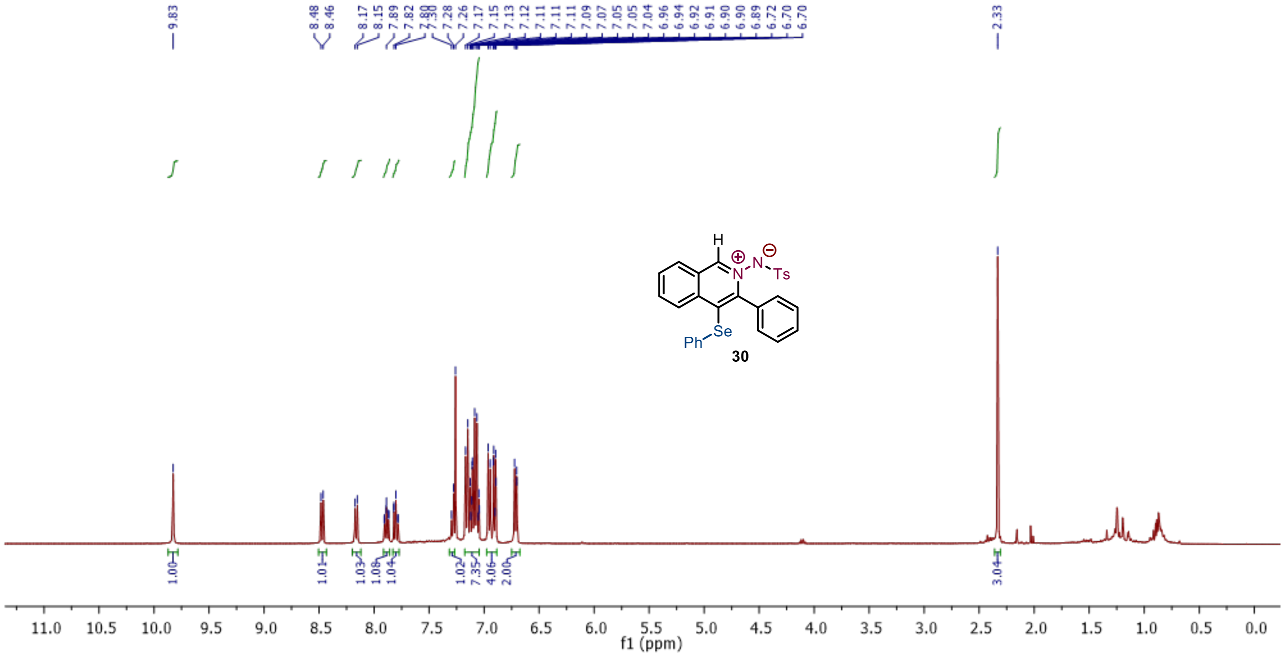

^{13}C NMR (101 MHz, $CDCl_3$)

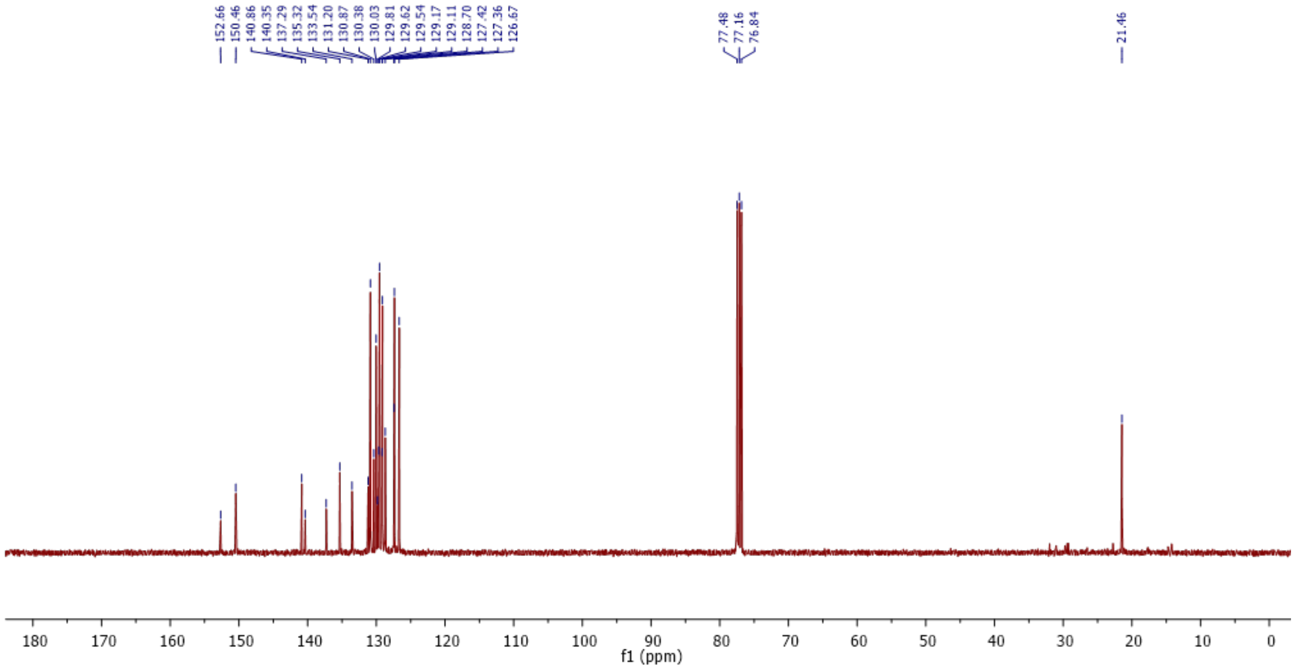

^{77}Se NMR (76 MHz, $CDCl_3$)

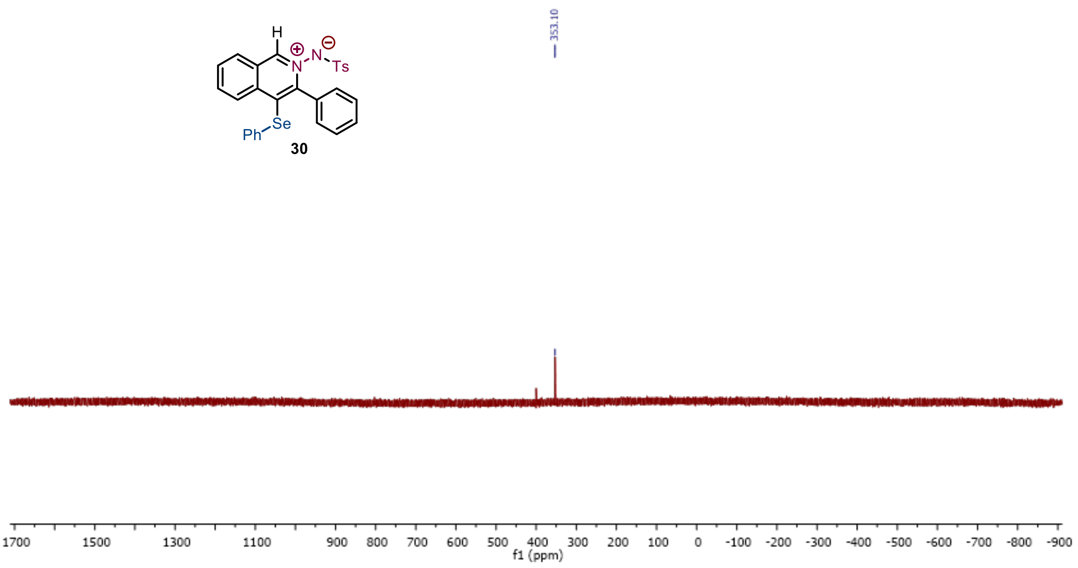
30

(3-(4-Methoxyphenyl)-4-(phenylselanyl)isoquinolin-2-ium-2-yl)(tosyl)amide (31):

^{1}H NMR (400 MHz, $CDCl_3$)

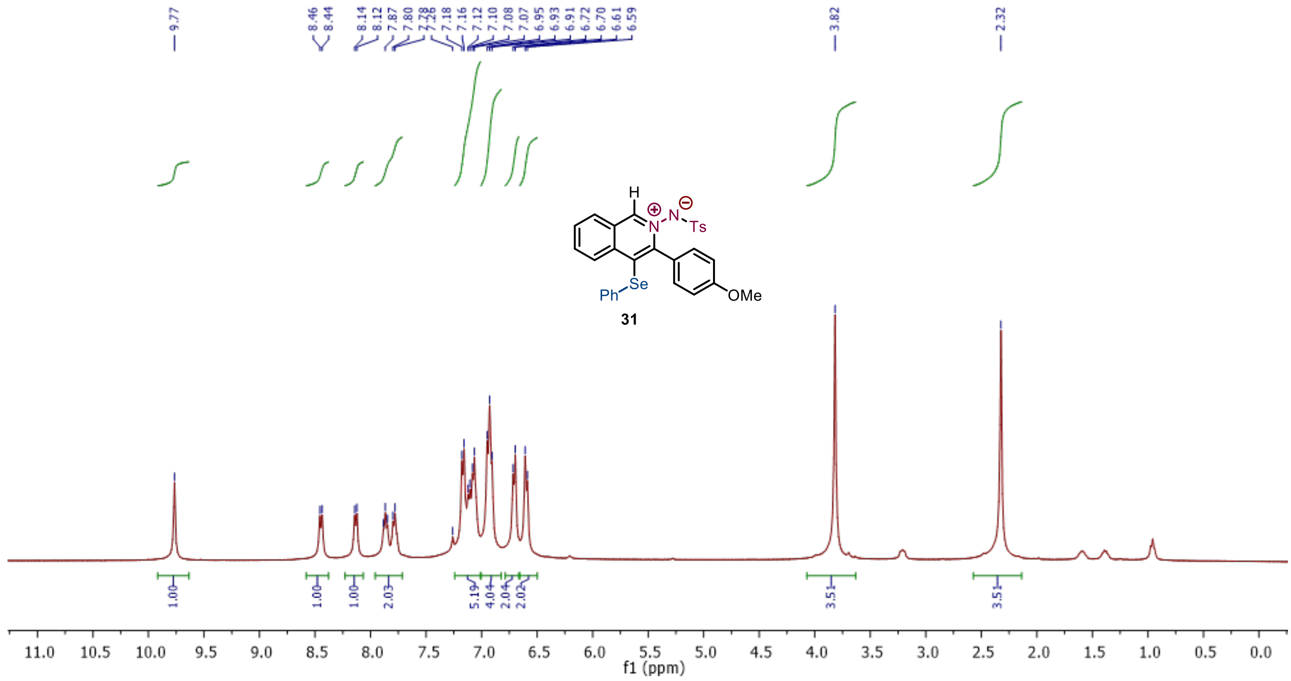

^{13}C NMR (101 MHz, $CDCl_3$)

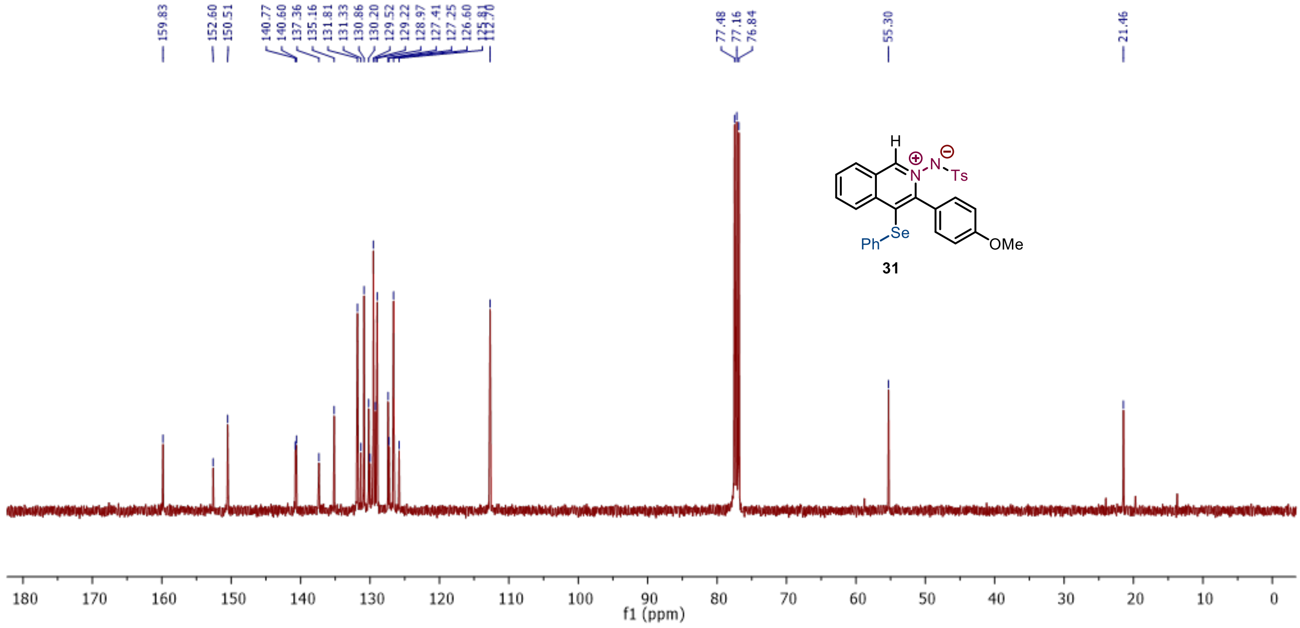

(3-phenyl-4-(p-tolylselanyl)isoquinolin-2-ium-2-yl)(tosyl)amide (32)

^{1}H NMR (400 MHz, $CDCl_3$)

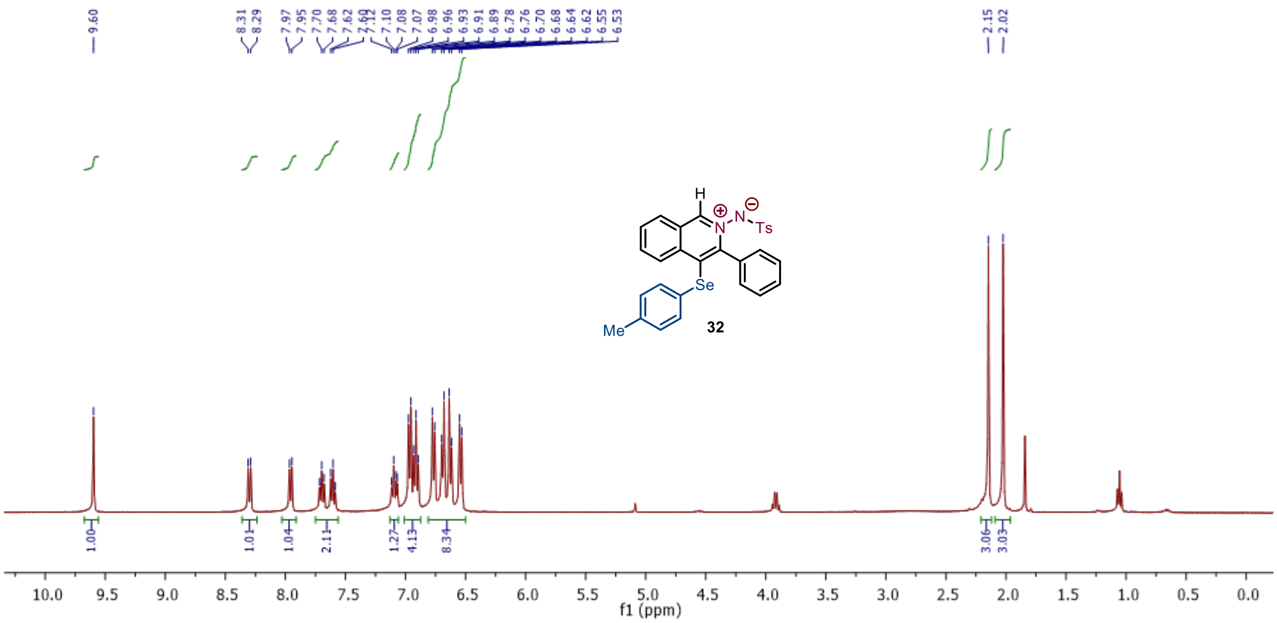

^{13}C NMR (101 MHz, $CDCl_3$)

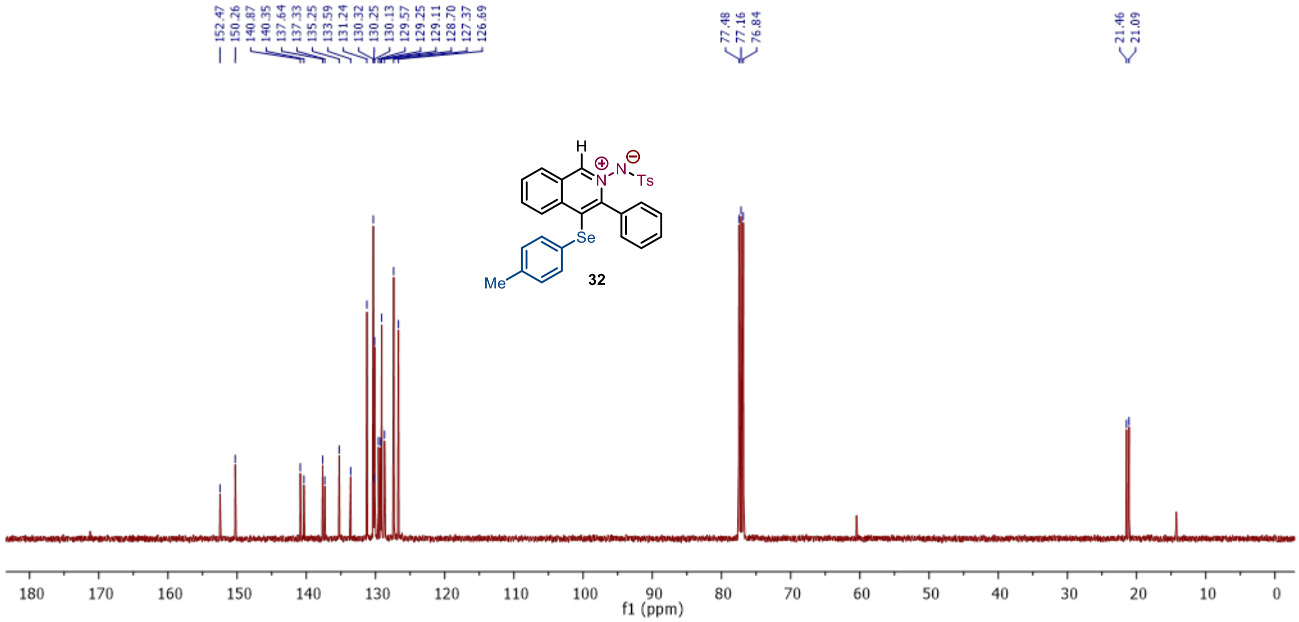

^{77}Se NMR (76 MHz, $CDCl_3$)

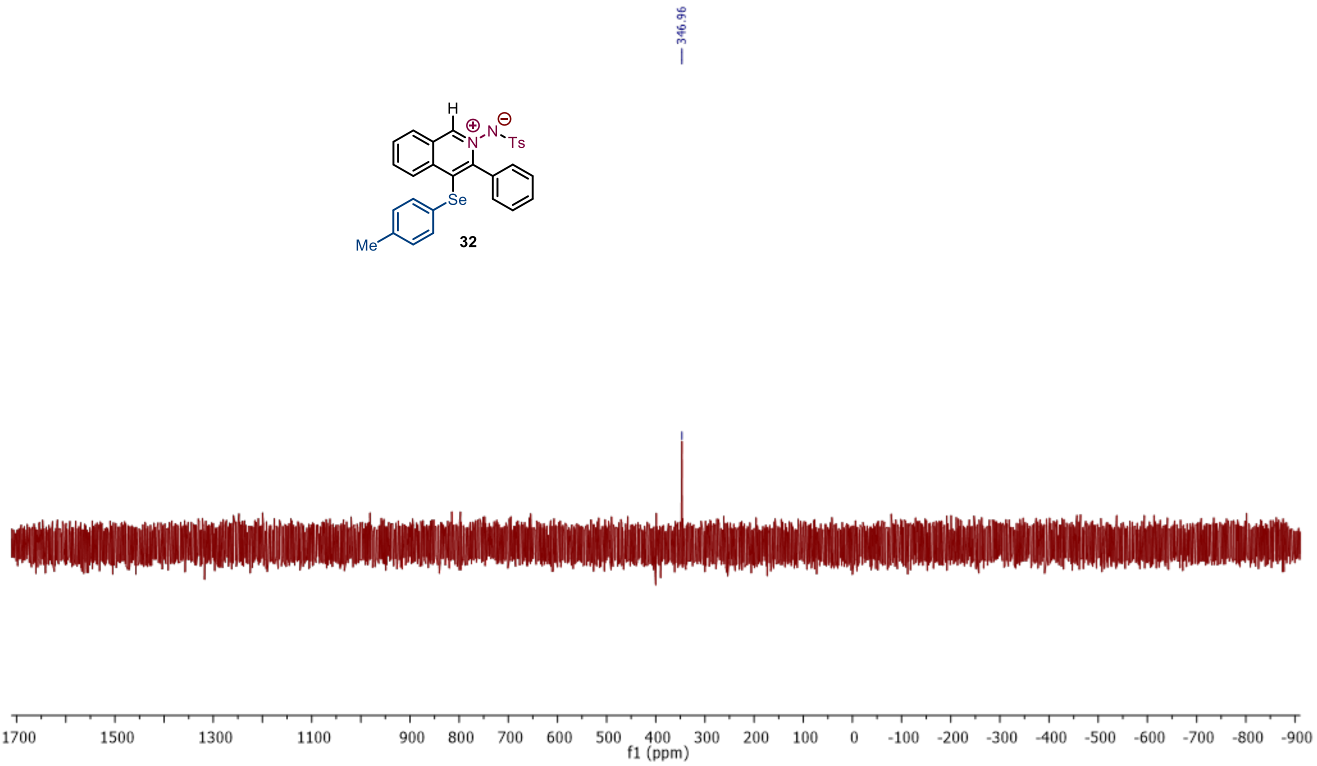

(3-Phenyl-4-(phenyltellanyl)isoquinolin-2-ium-2-yl)(tosyl)amide (33)

^{1}H NMR (400 MHz, $CDCl_3$)

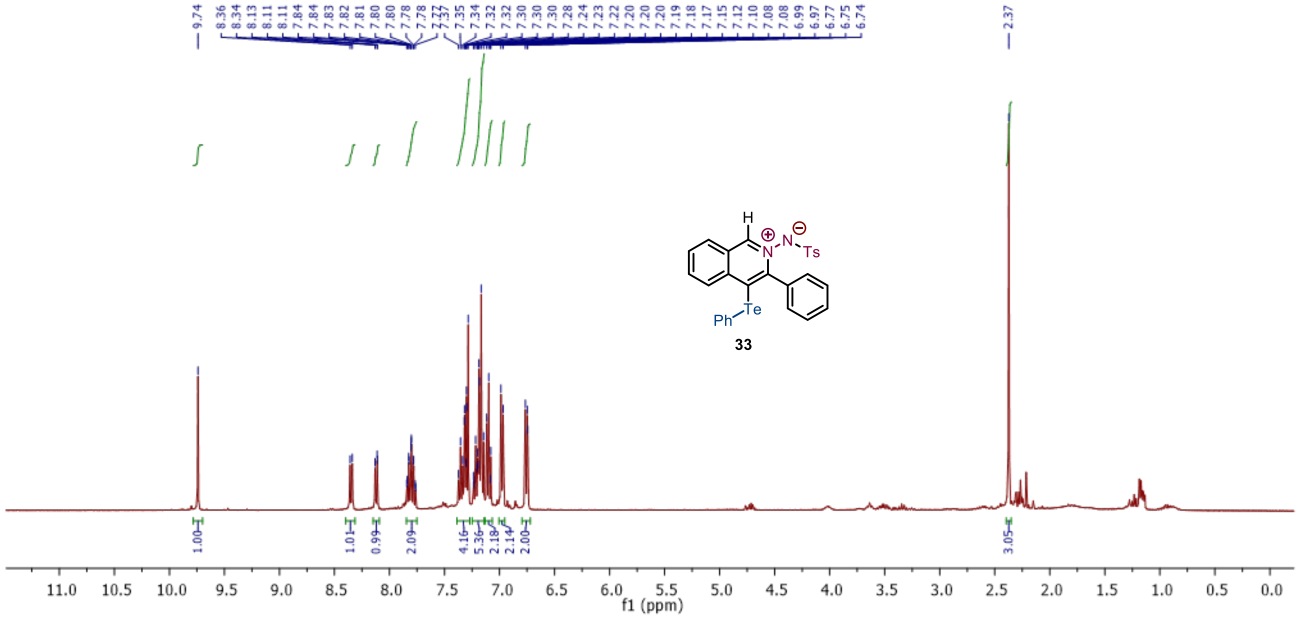

^{13}C NMR (101 MHz, $CDCl_3$)

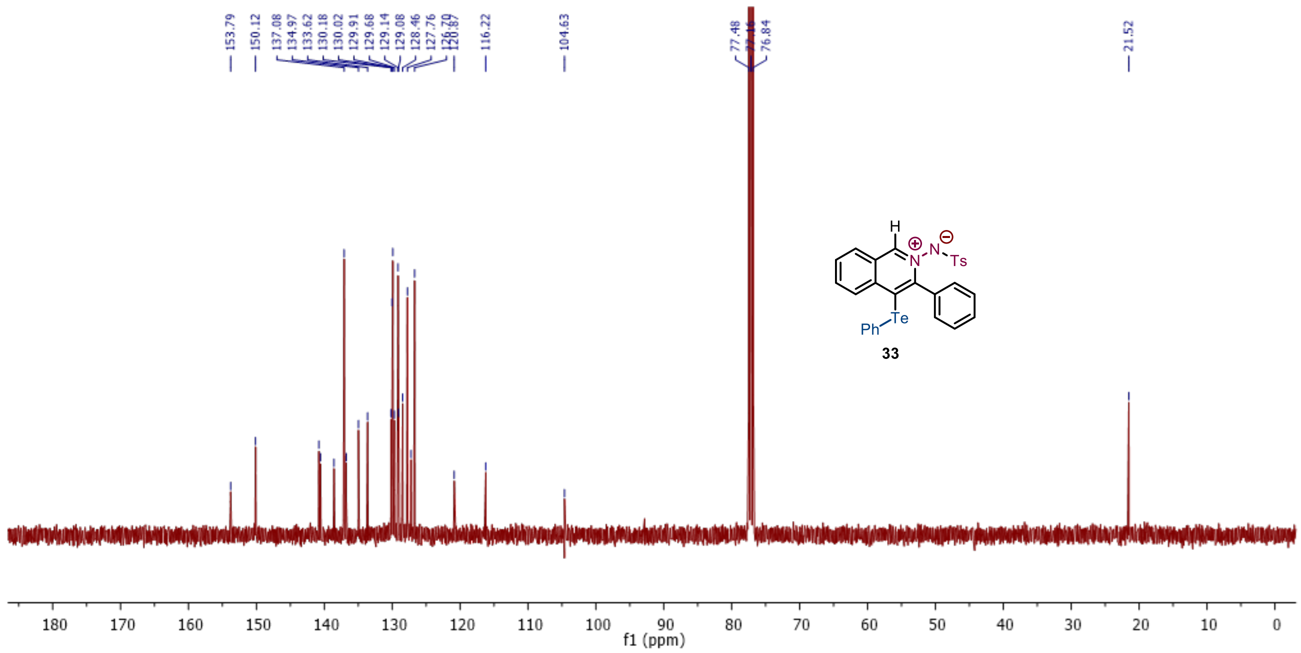

5-methyl-7-phenyl-8-(phenylselanyl)-1,6-naphthyridine 6-oxide (34)

^{1}H NMR (400 MHz, $CDCl_3$)

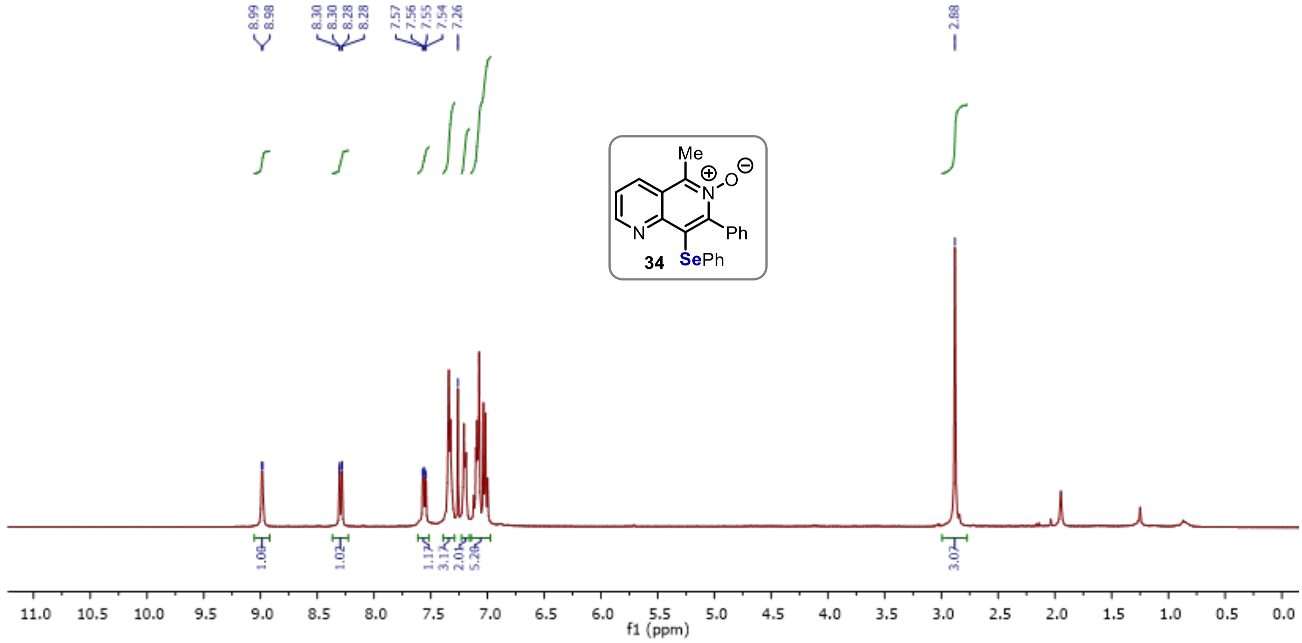

^{13}C NMR (101 MHz, $CDCl_3$)

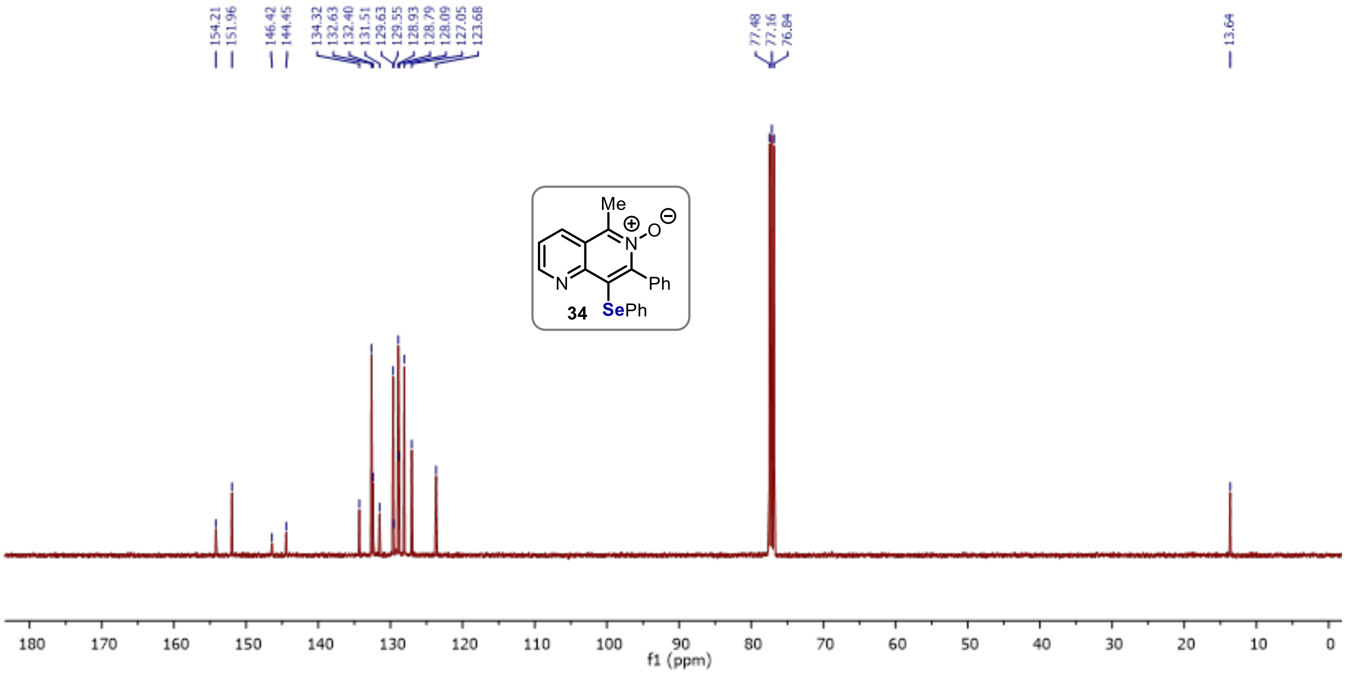

^{77}Se NMR (76 MHz, $CDCl_3$)

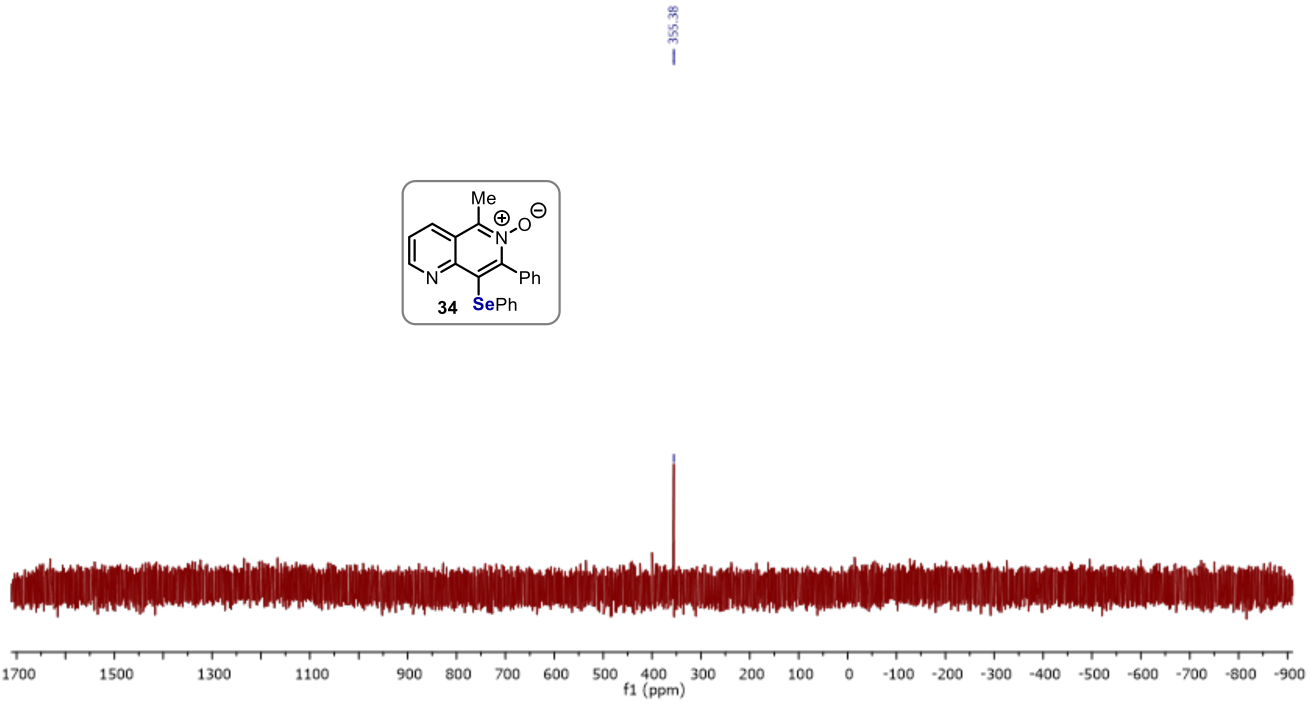

(*Z*)-3-Methyl-1-(phenyl(phenylselanyl)methylene)-1H-isoindole 2-oxide (4)

^{1}H NMR (400 MHz, $CDCl_3$)

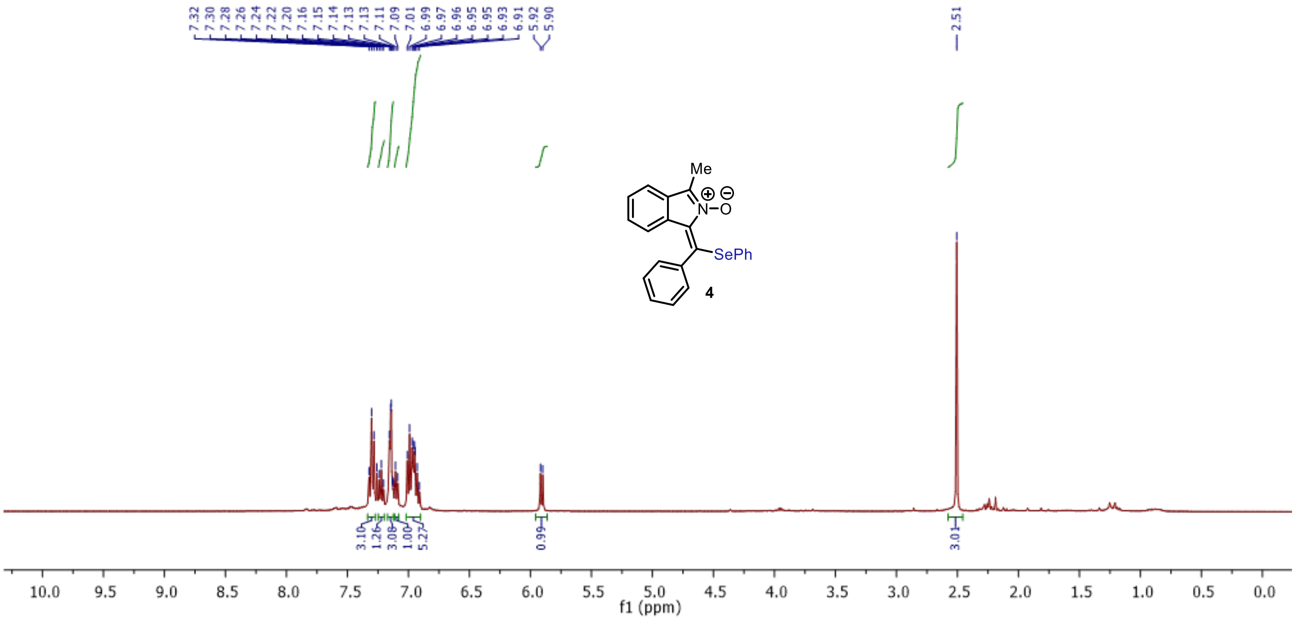

^{13}C NMR (101 MHz, $CDCl_3$)

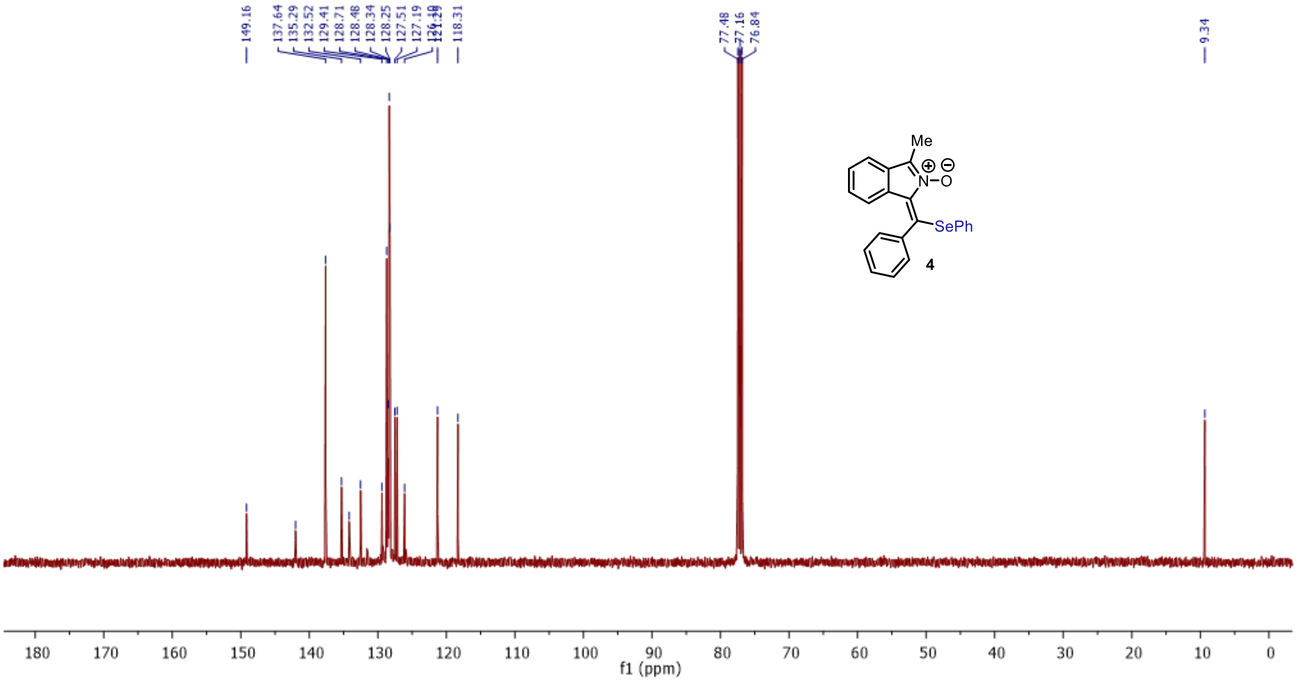

(*Z*)-3-Methyl-1-((phenylselanyl)(p-tolyl)methylene)-1H-isoindole 2-oxide (36)

^{1}H NMR (400 MHz, CDCl$_3$

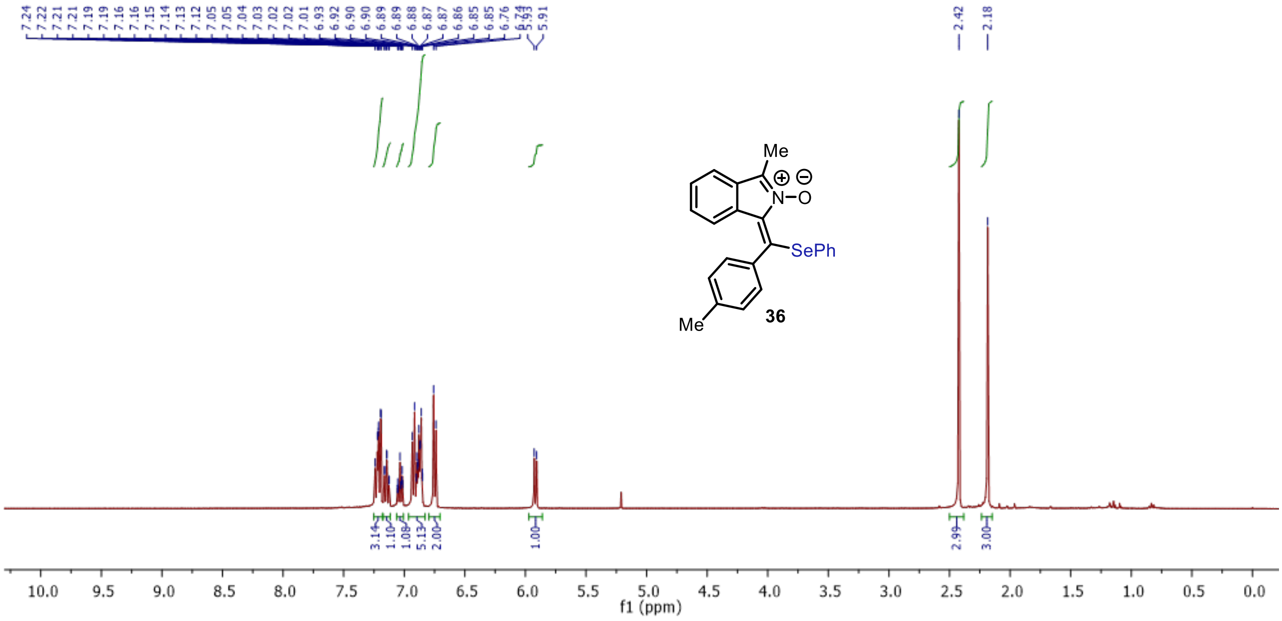

^{13}C NMR (101 MHz, CDCl$_3$)

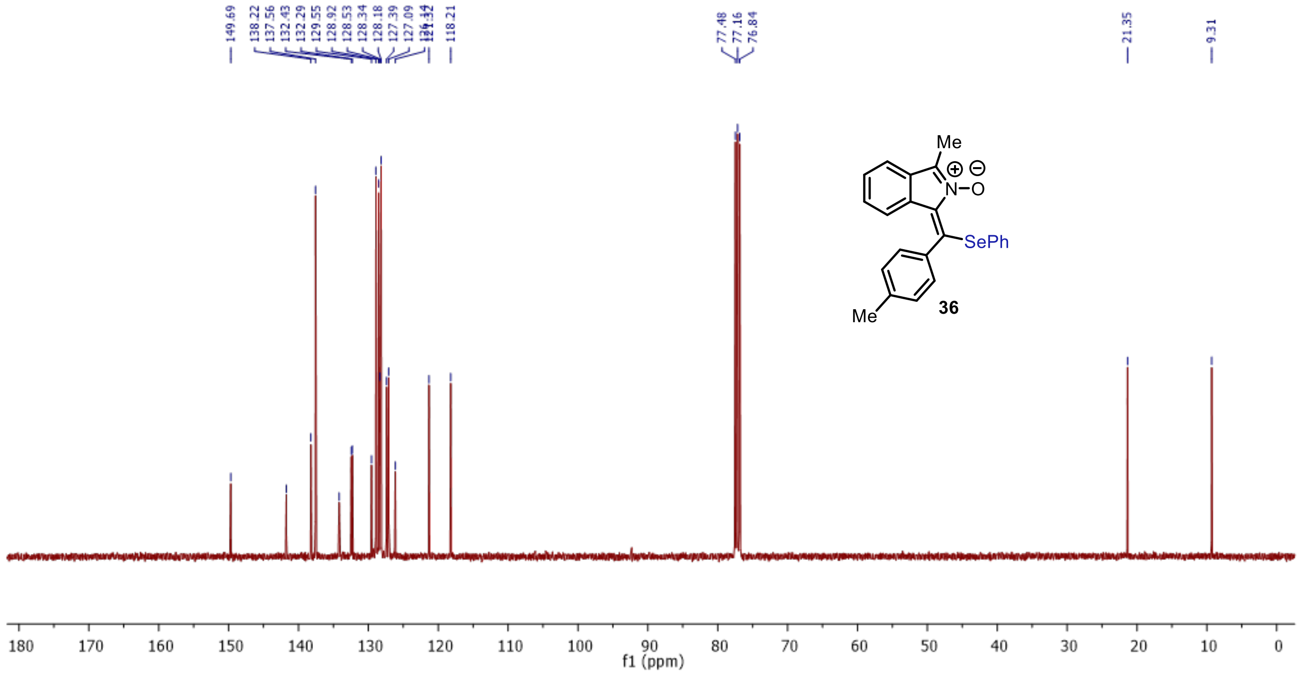

(Z)-3-Methyl-1-(phenyl(p-tolylselanyl)methylene)-1H-isoindole 2-oxide (37)

^{1}H NMR (400 MHz, $CDCl_3$)

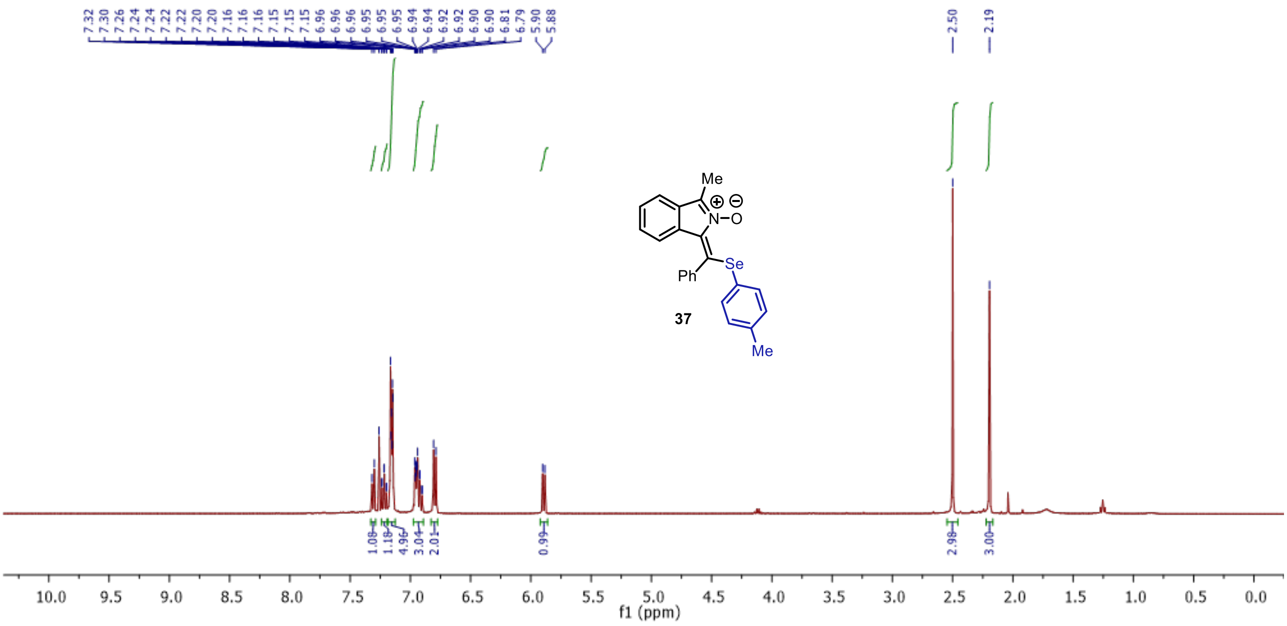

^{13}C NMR (101 MHz, $CDCl_3$)

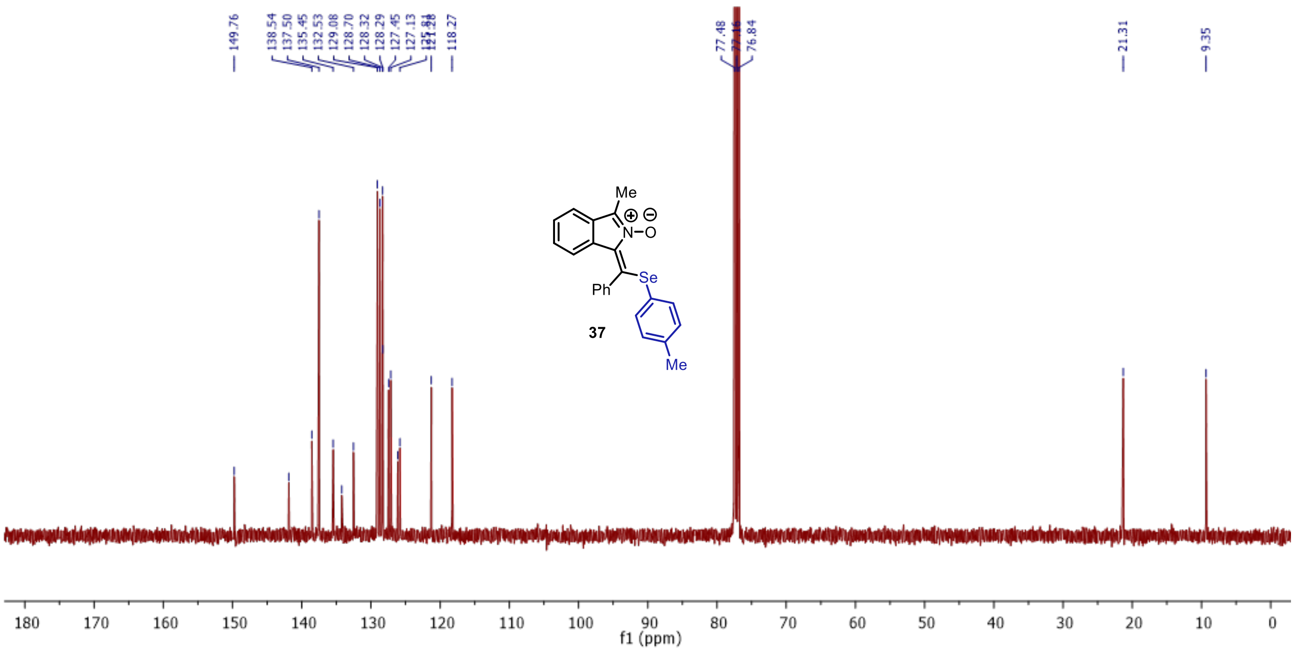

^{77}Se NMR (76 MHz, $CDCl_3$)

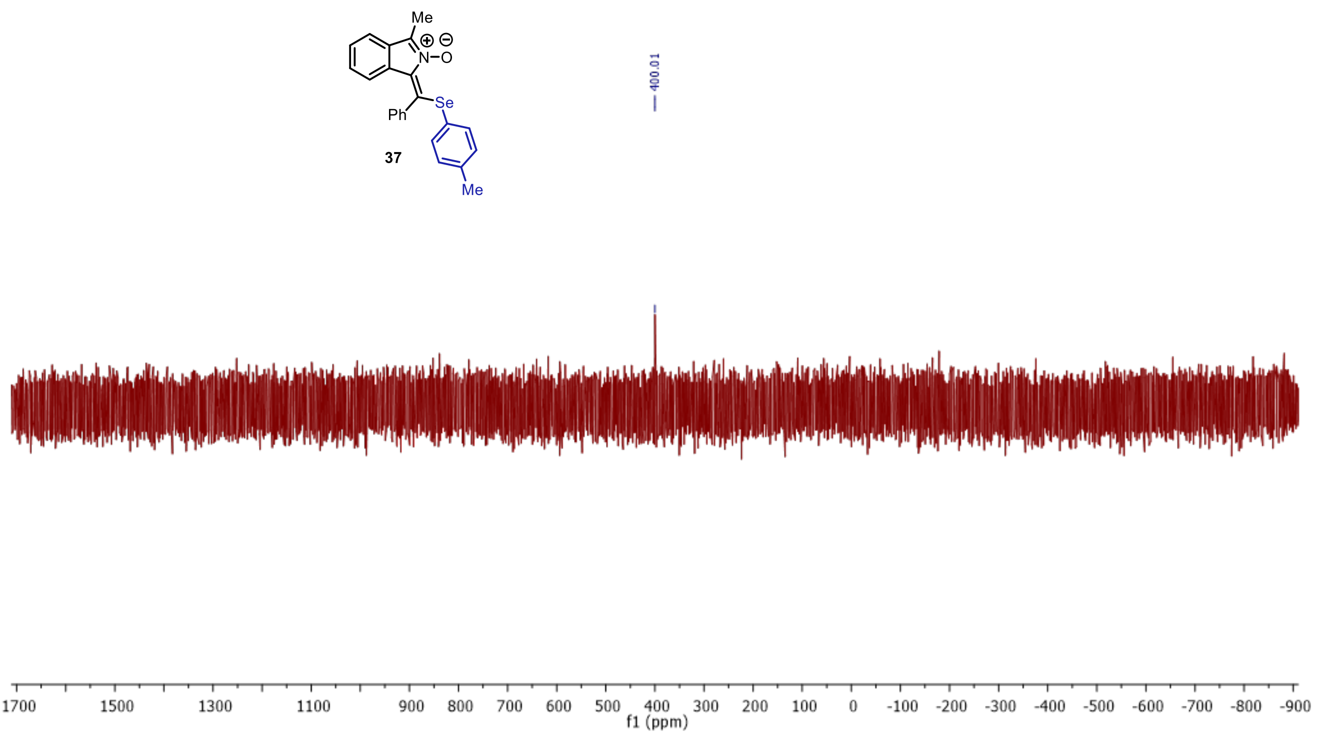

(Z)-1-((Benzylselanyl)(phenyl)methylene)-3-methyl-1H-isoindole 2-oxide (38)

^{1}H NMR (400 MHz, $CDCl_3$)

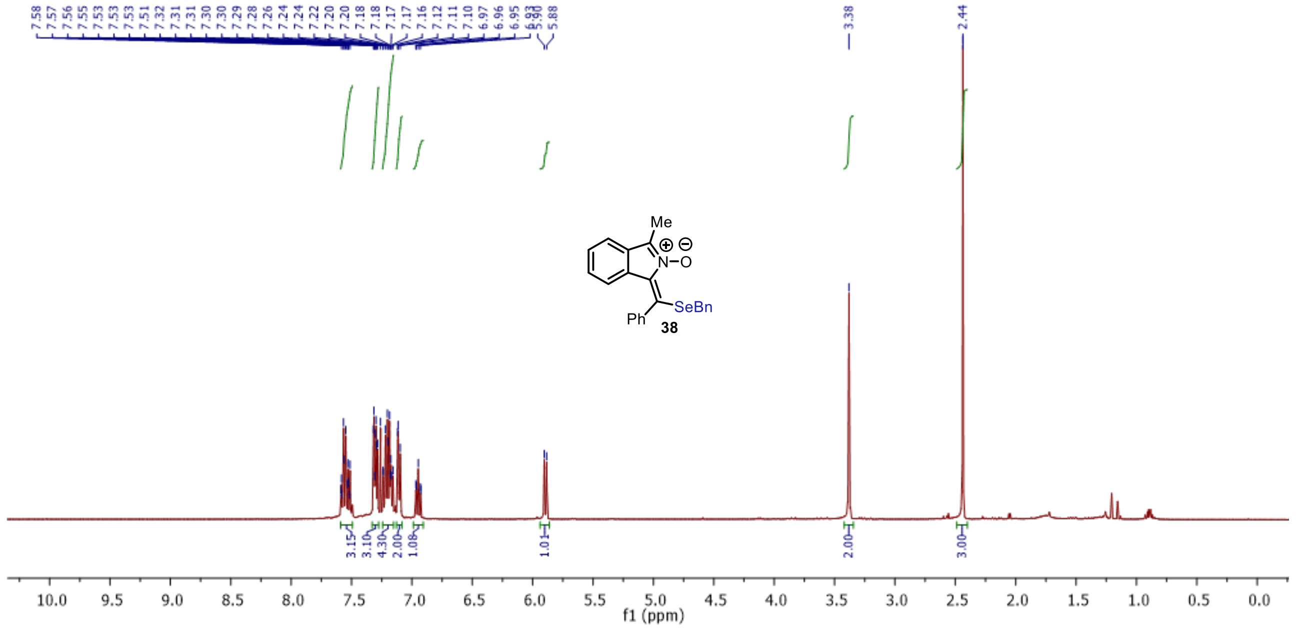

^{13}C NMR (101 MHz, $CDCl_3$)

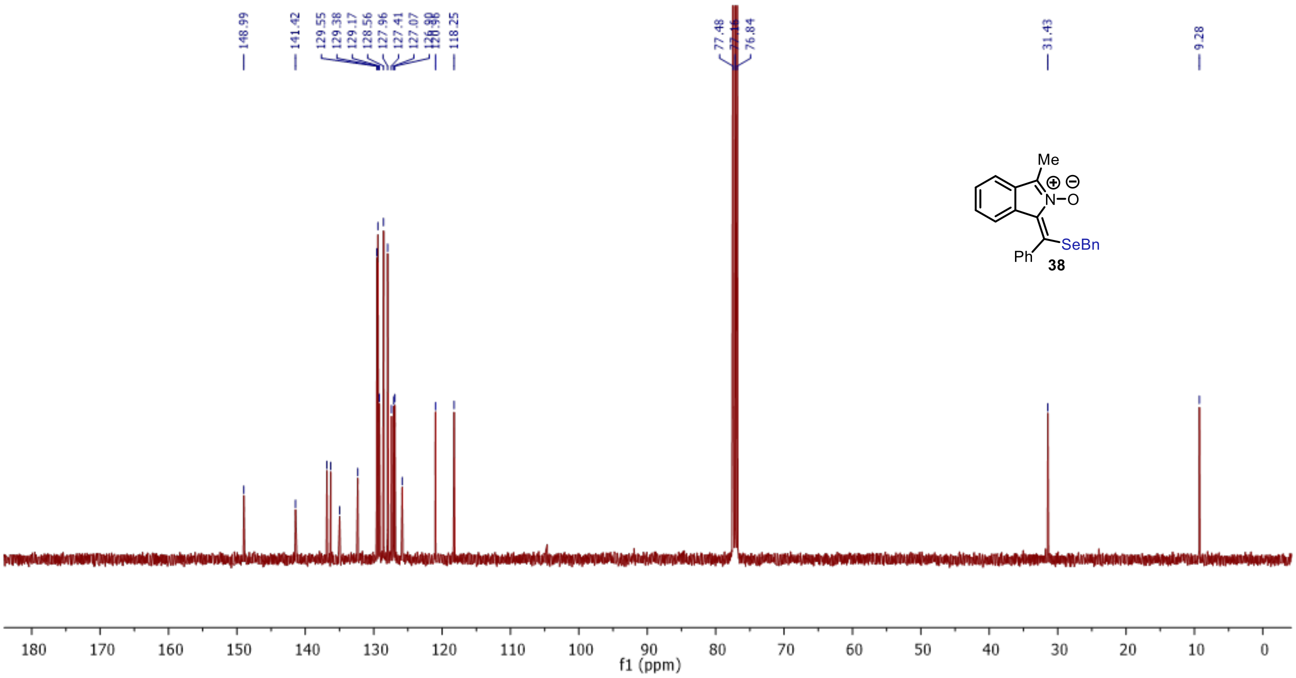

^{77}Se NMR (76 MHz, $CDCl_3$)

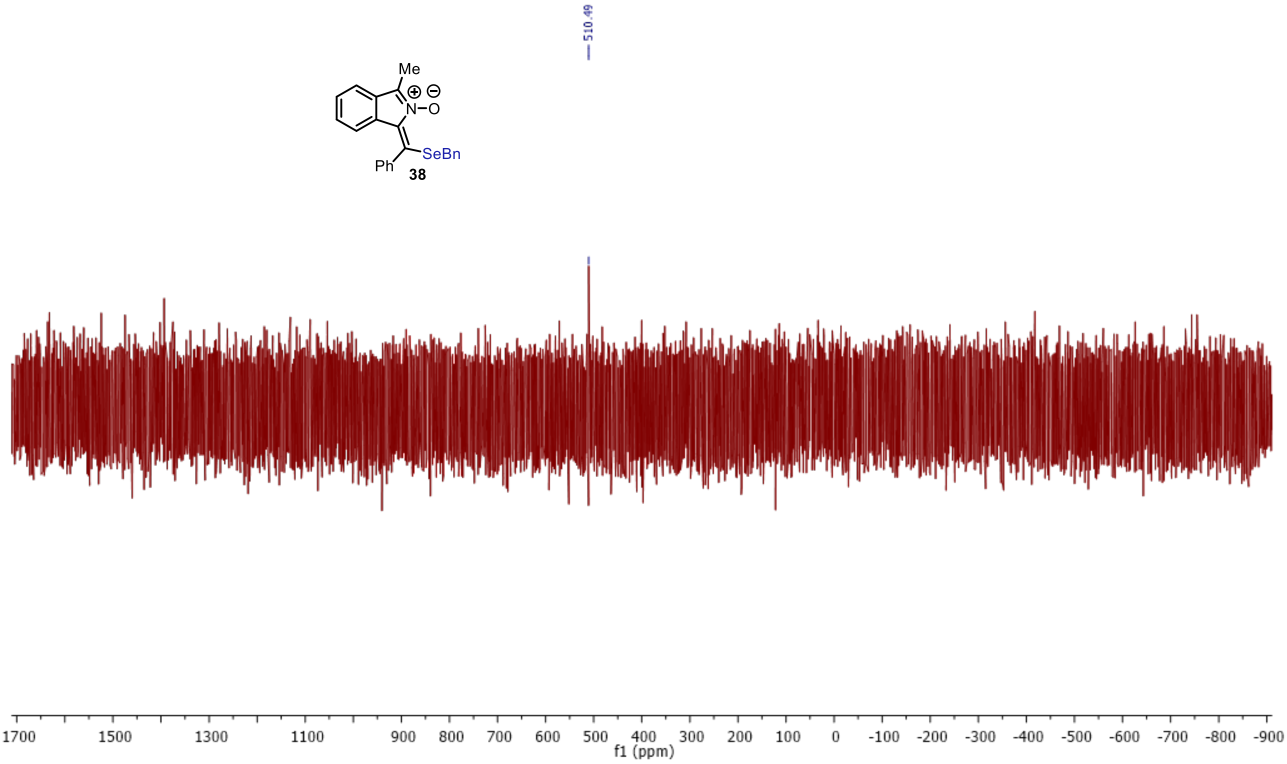

(*Z*)-1-((3-Chlorophenyl)(phenylselanyl)methylene)-3-methyl-1H-isoindole 2-oxide (39):

^{1}H NMR (400 MHz, $CDCl_3$)

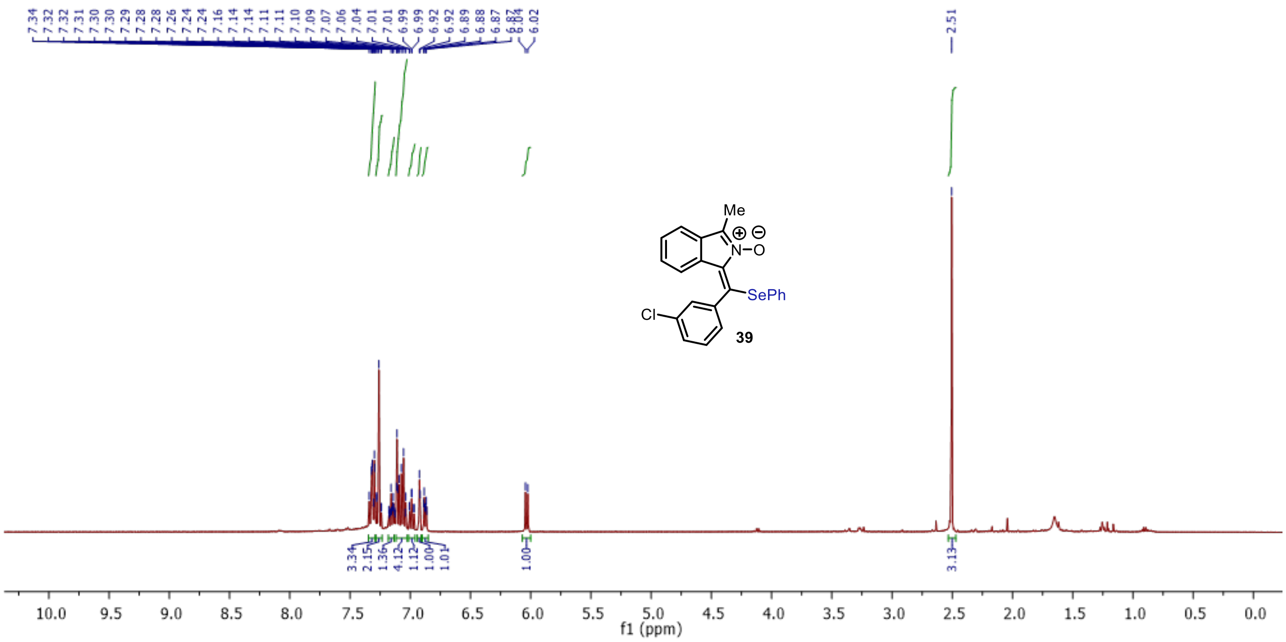

^{13}C NMR (101 MHz, $CDCl_3$)

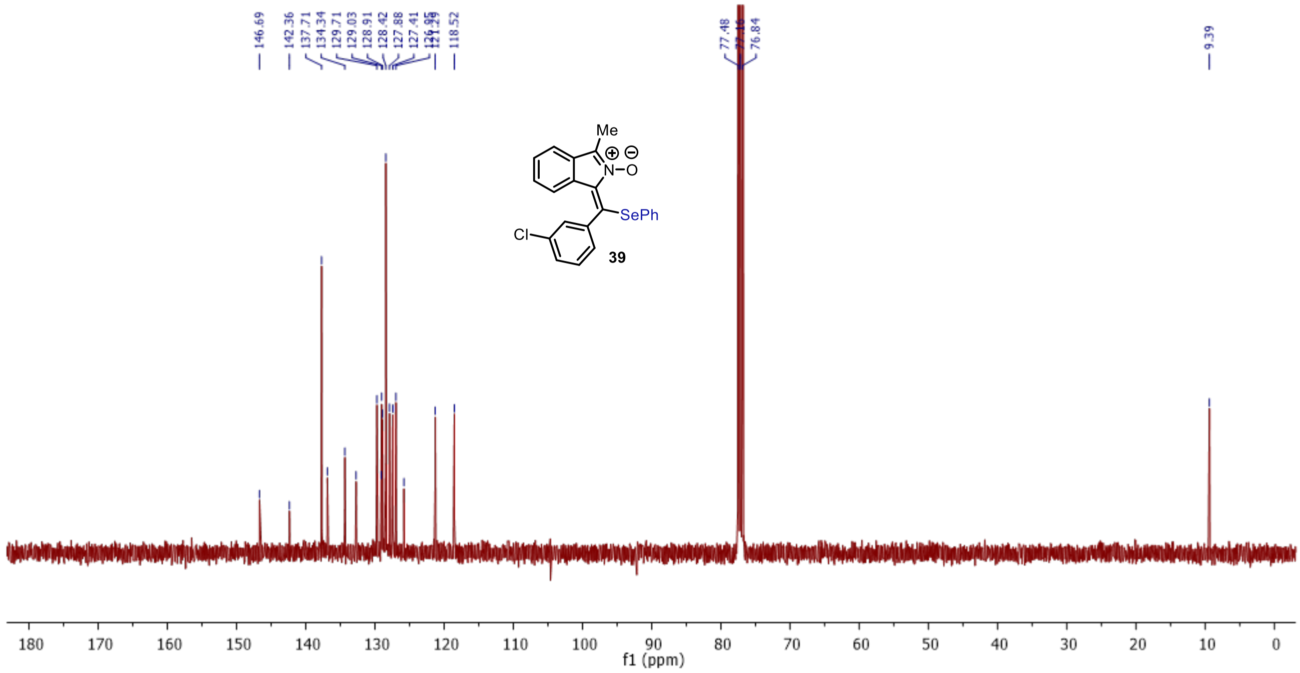

(*Z*)-1-((4-Methoxyphenyl)(phenylselanyl)methylene)-3-methyl-1H-isoindole 2-oxide (40)

^{1}H NMR (400 MHz, $CDCl_3$)

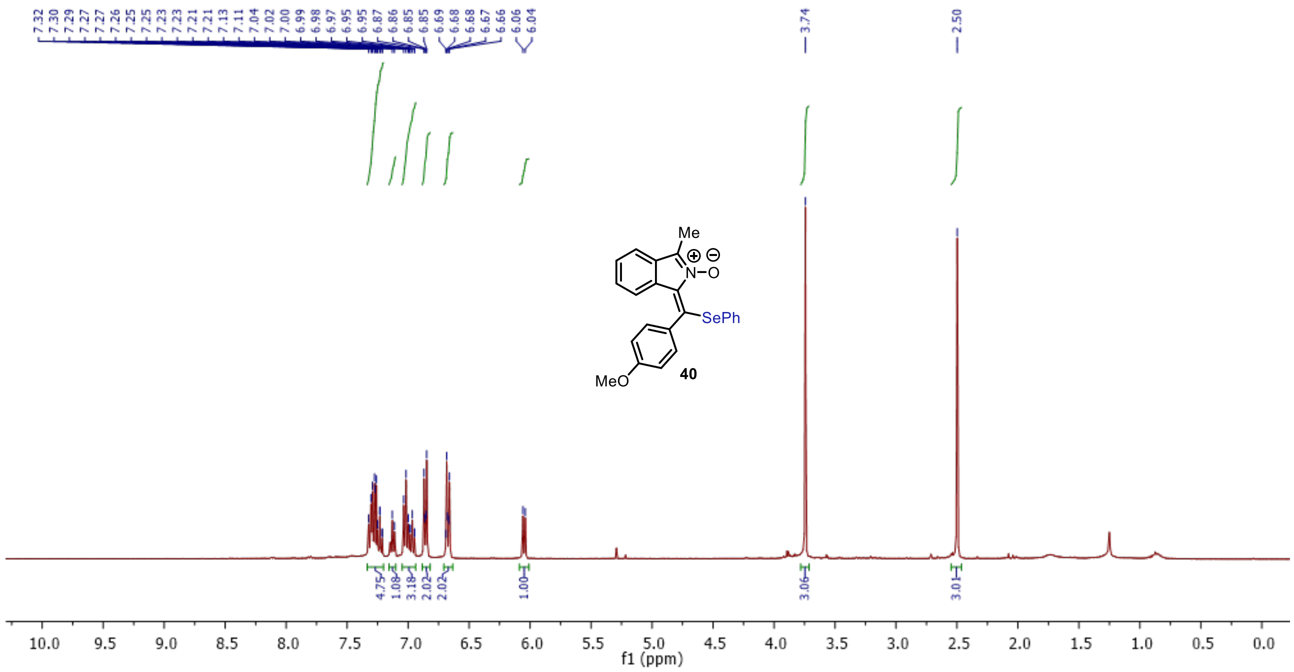

^{13}C NMR (101 MHz, $CDCl_3$)

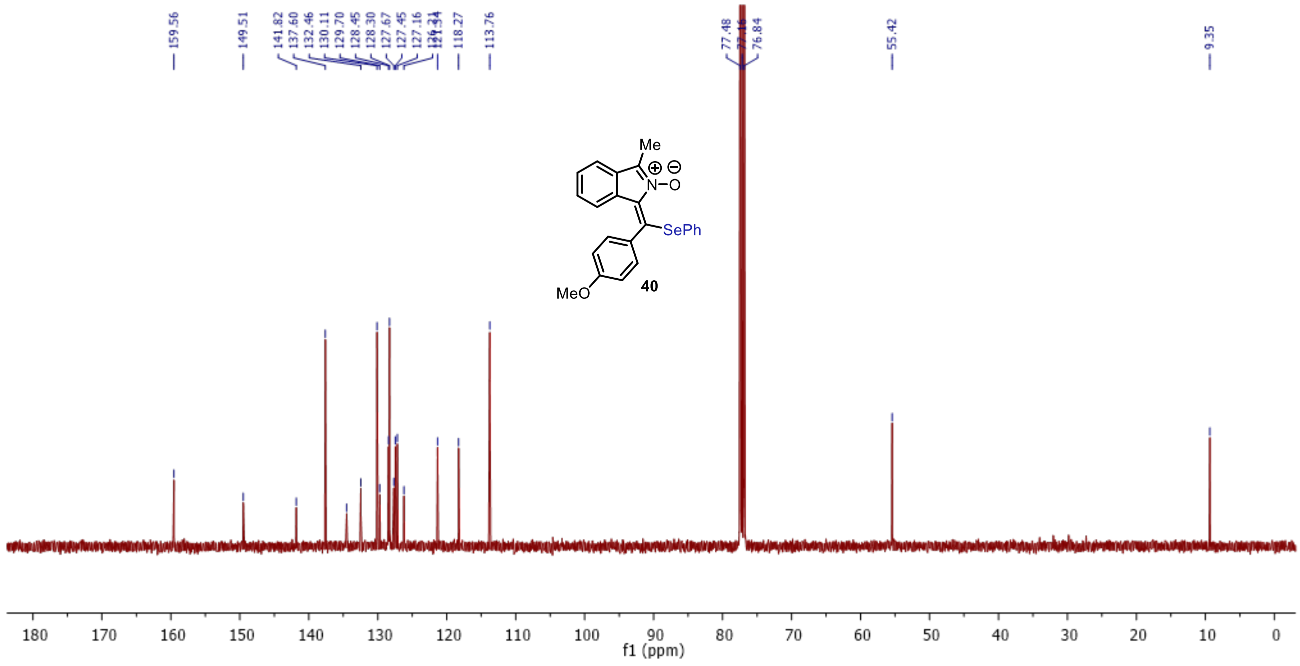

^{77}Se NMR (76 MHz, $CDCl_3$)

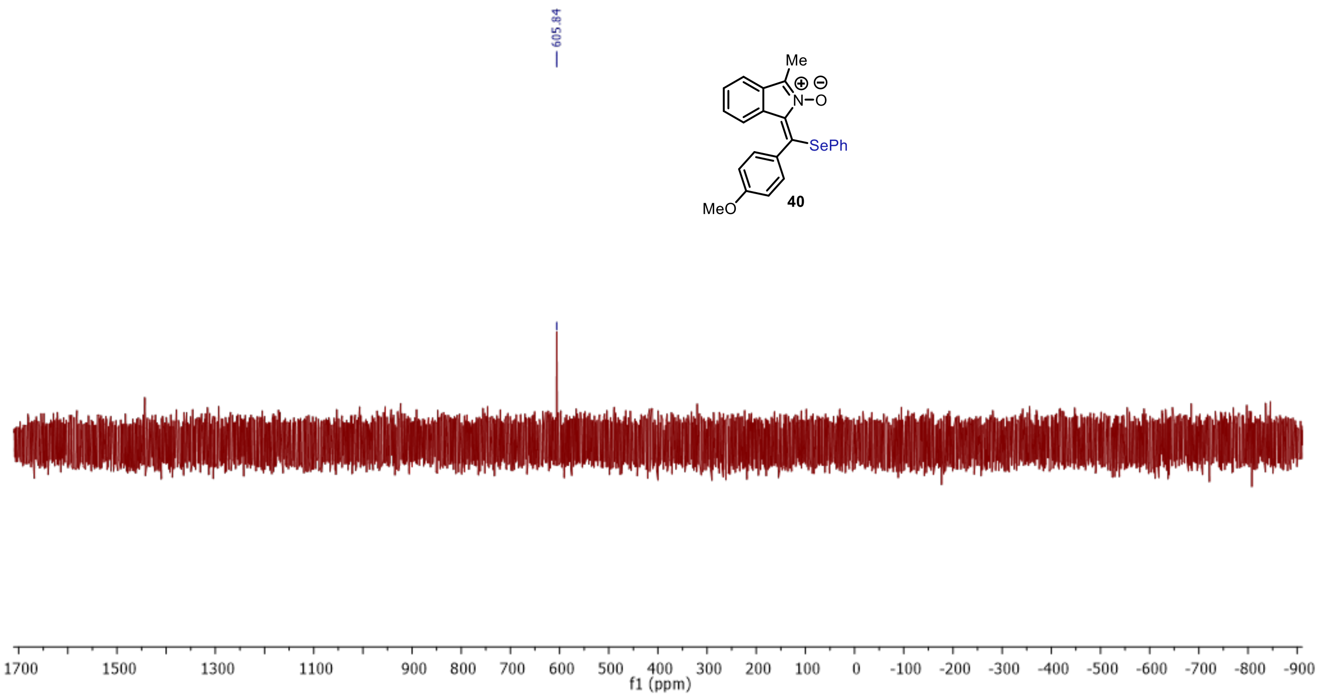

(Z)-1-((Benzylselanyl)(4-methoxyphenyl)methylene)-3-methyl-1H-isoindole 2-oxide (41)

There are non- isolable impurities in the product.

^{1}H NMR (400 MHz, $CDCl_3$)

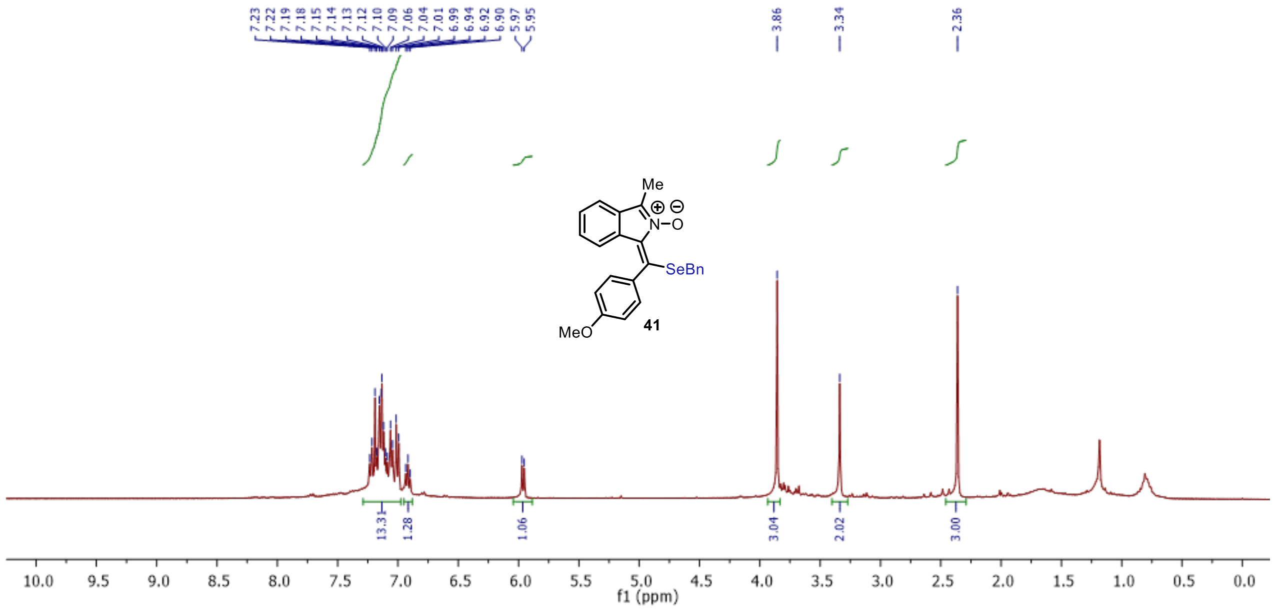

^{13}C NMR (101 MHz, $CDCl_3$)

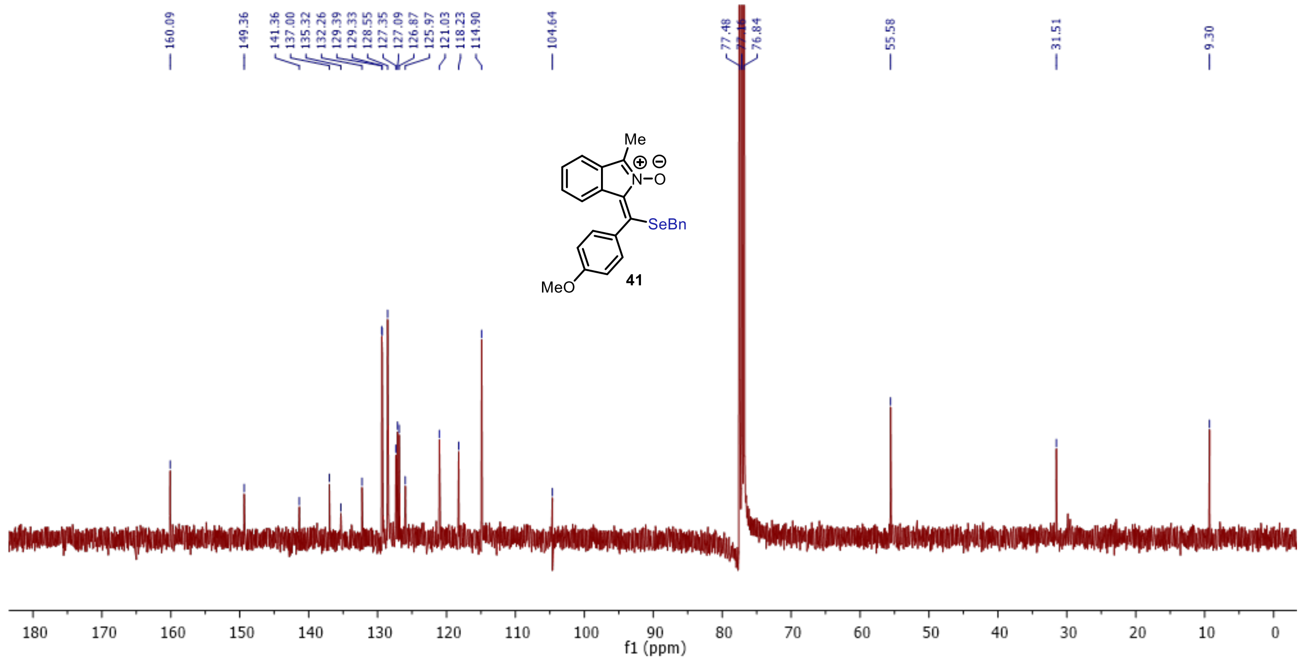

^{77}Se NMR (76 MHz, $CDCl_3$)

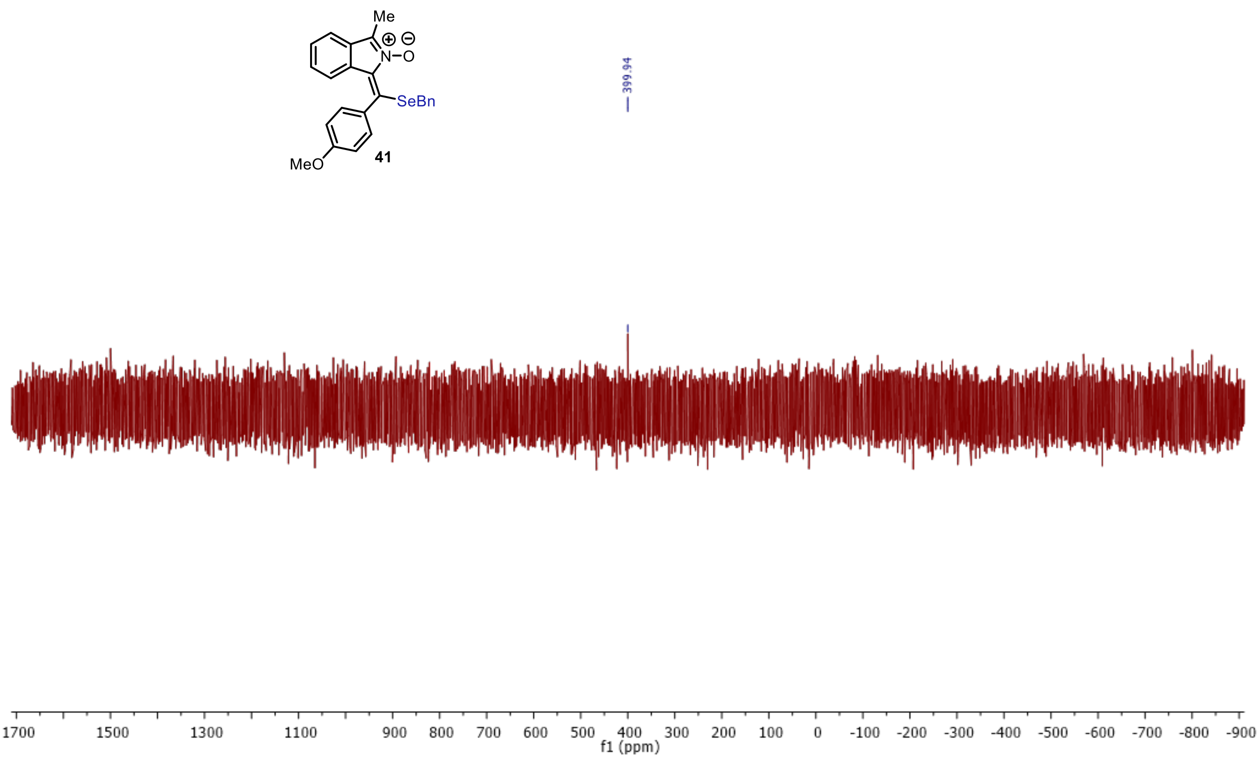

3-Methyl-1-(((4-phenoxyphenyl)selanyl)(phenyl)methylene)-1H-isoindole 2-oxide (major isomer 42)

^{1}H NMR (500 MHz, $CDCl_3$)

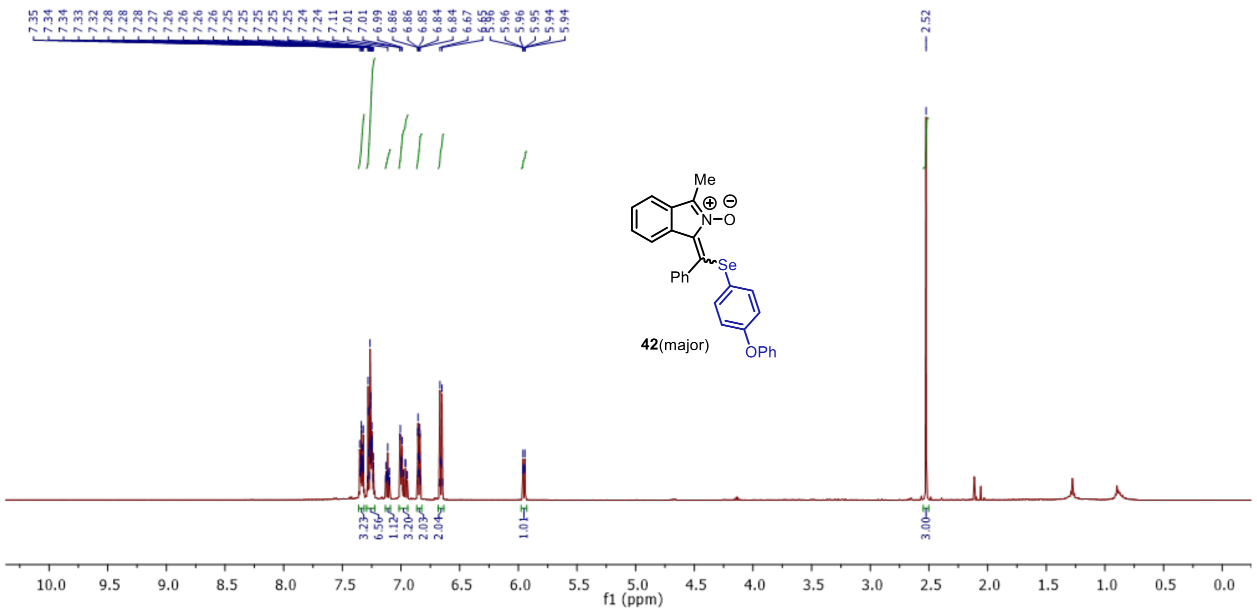

^{13}C NMR (126 MHz, $CDCl_3$)

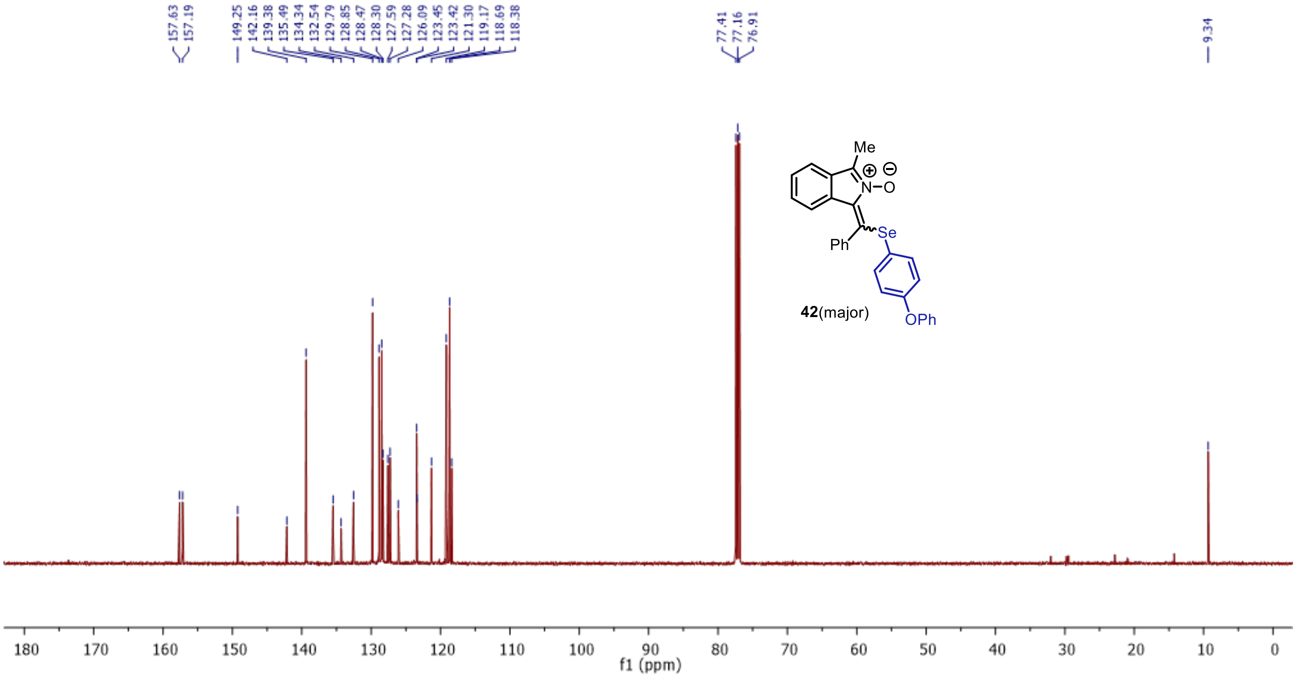

3-Methyl-1-(((4-phenoxyphenyl)selanyl)(phenyl)methylene)-1H-isoindole 2-oxide (minor isomer 42)

^{1}H NMR (400 MHz, $CDCl_3$)

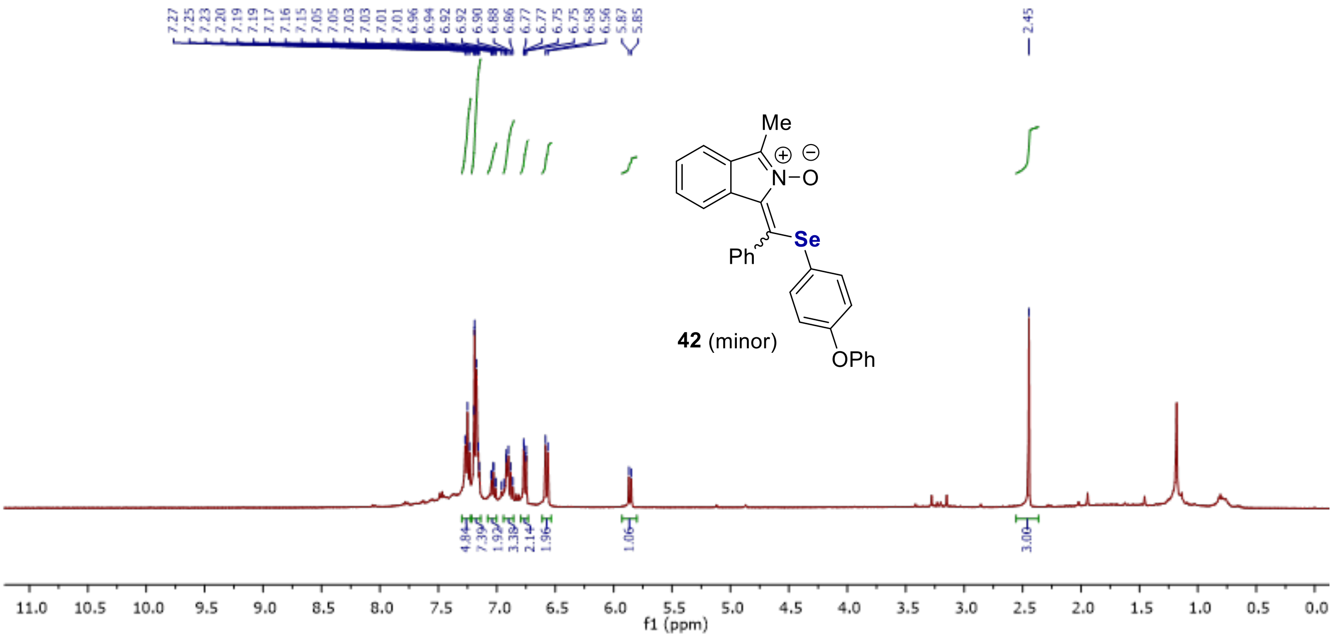

(*Z*)-3-Methyl-1-((phenylselanyl)(4-(trifluoromethyl)phenyl)methylene)-1H-isoindole 2-oxide (43)

^{1}H NMR (400 MHz, $CDCl_3$)

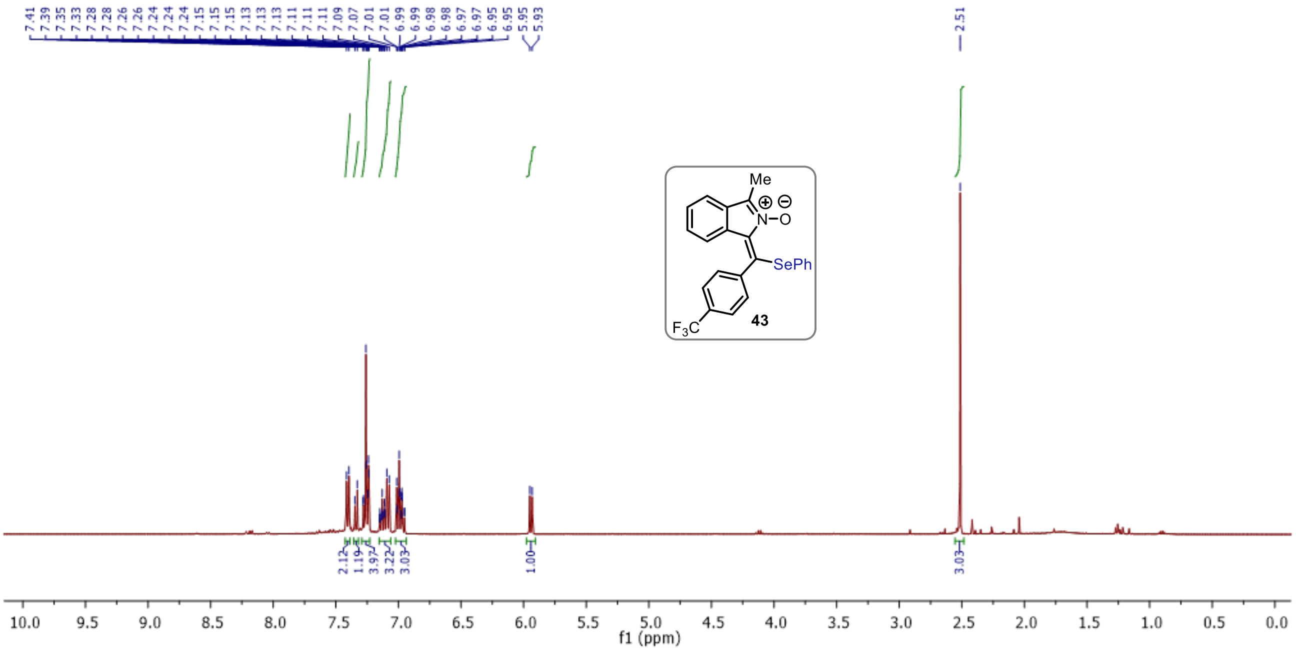

^{13}C NMR (101 MHz, $CDCl_3$)

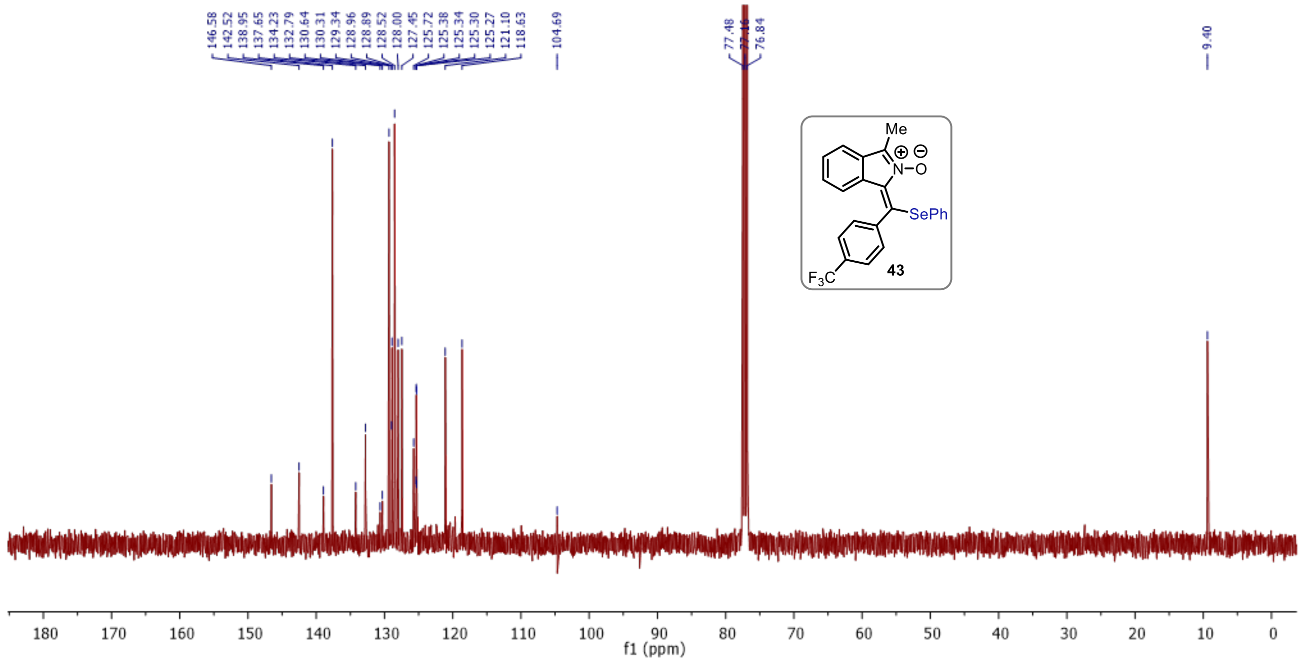

^{19}F NMR (376 MHz, $CDCl_3$)

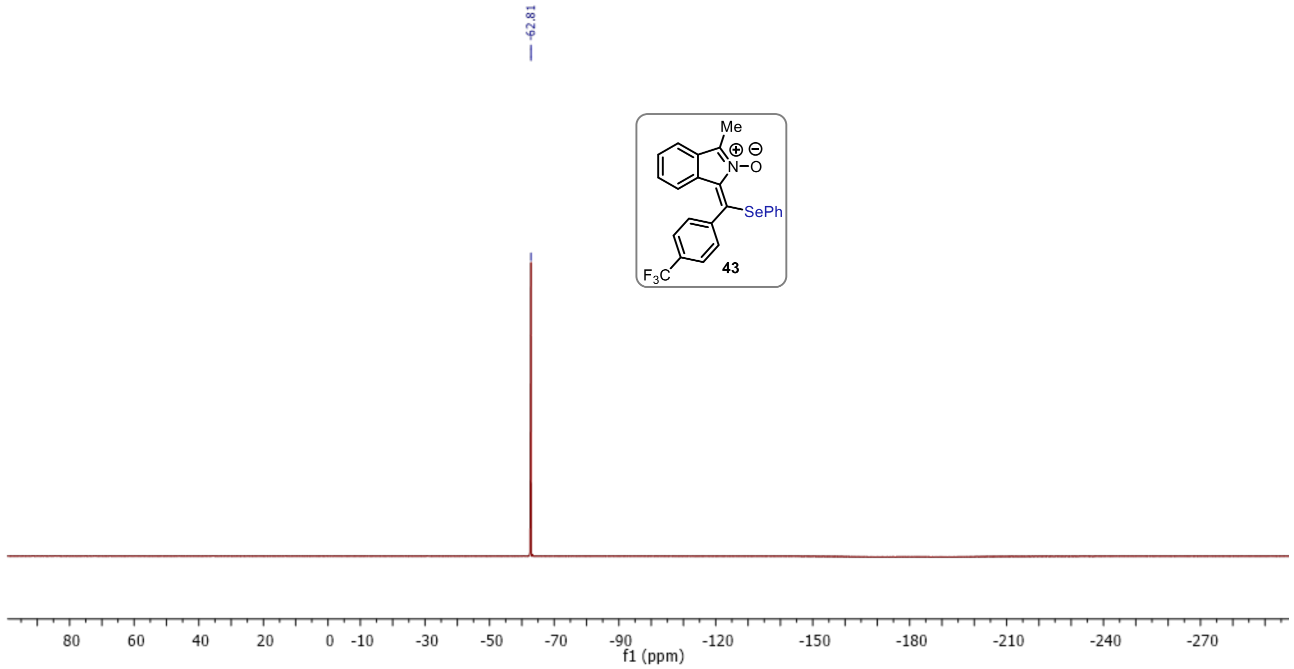

(*Z*)-1-((4-Fluorophenyl)(phenylselanyl)methylene)-3-methyl-1H-isoindole 2-oxide (44)

^{1}H NMR (400 MHz, $CDCl_3$)

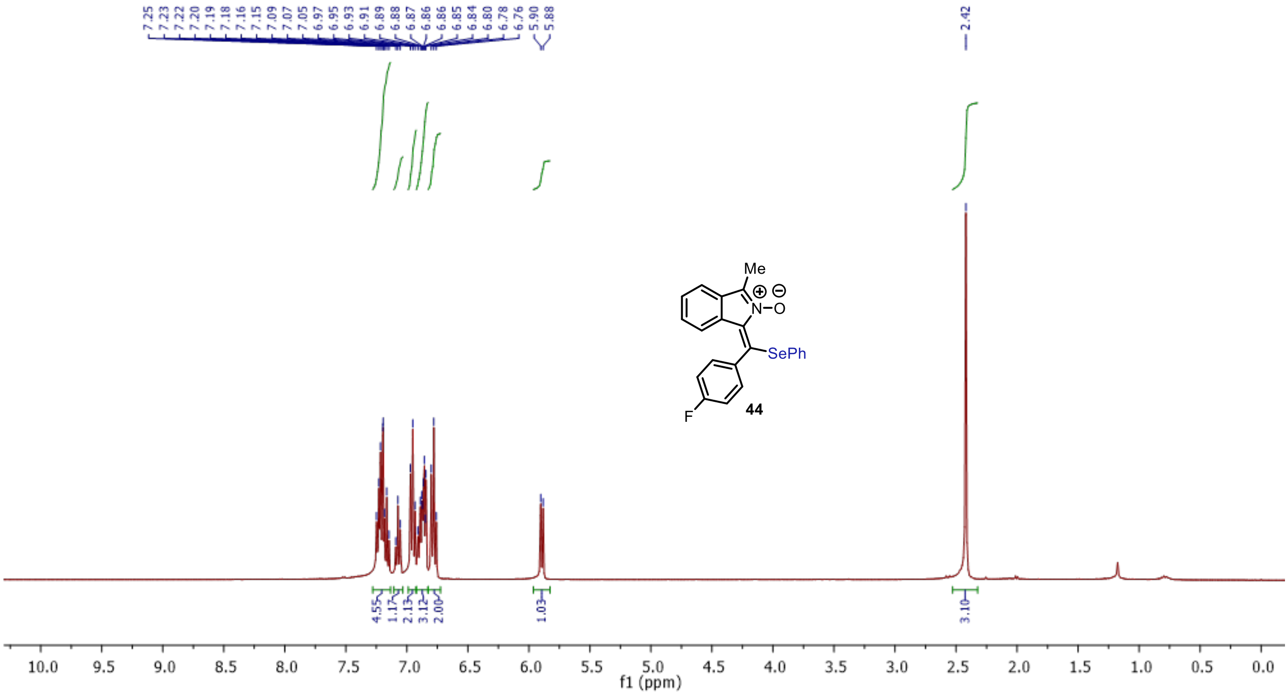

^{13}C NMR (101 MHz, $CDCl_3$)

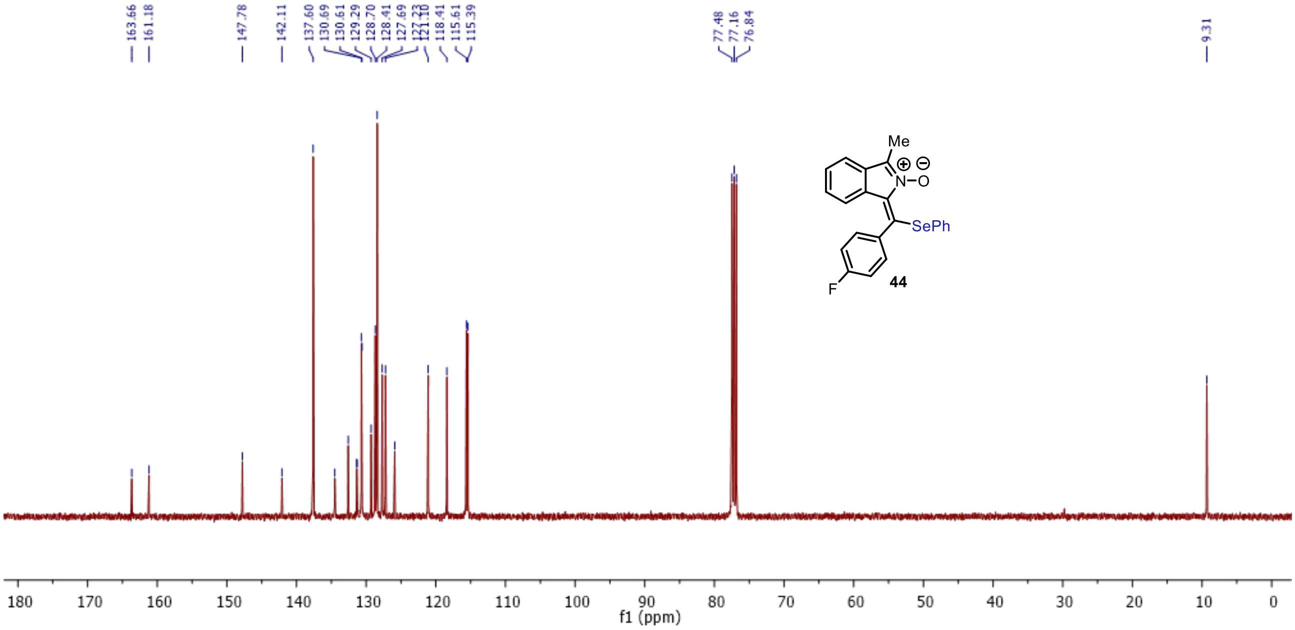

^{19}F NMR (376 MHz, $CDCl_3$)

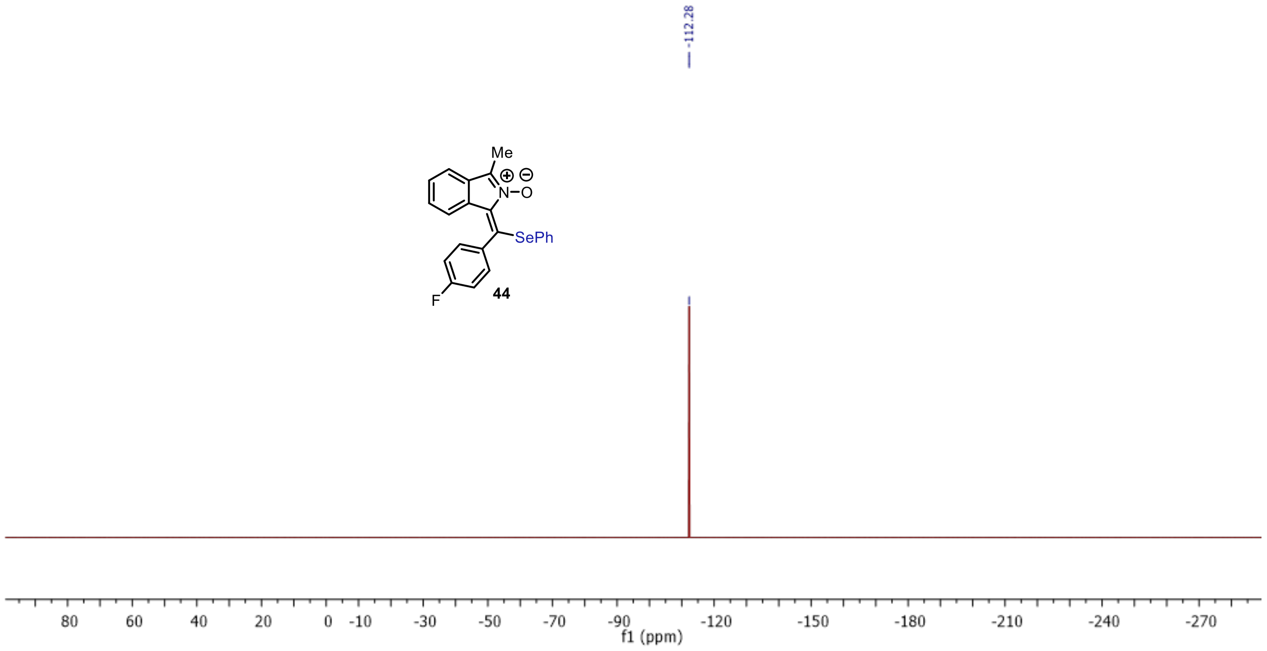

^{77}Se NMR (95 MHz, $CDCl_3$)

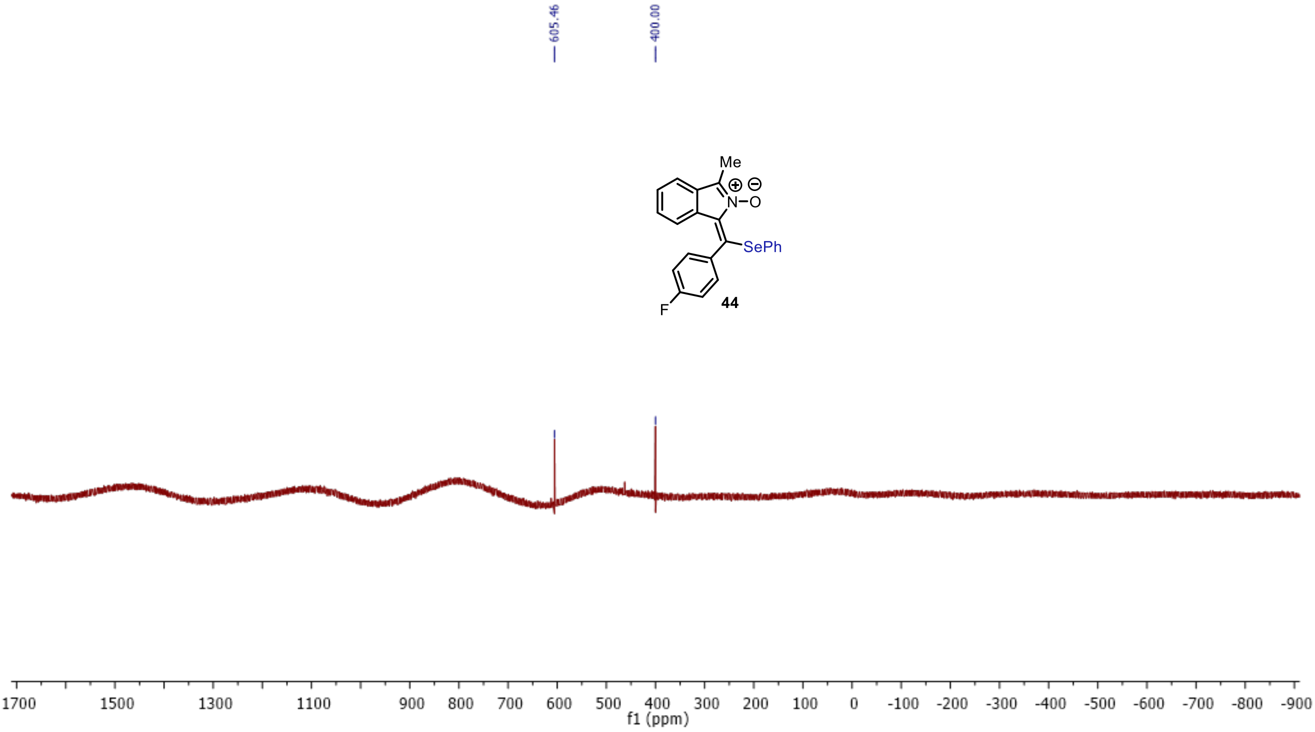

1-(((3-Fluorophenyl)selanyl)(phenyl)methylene)-3-methyl-1H-isoindole 2-oxide (45)

^{1}H NMR (400 MHz, $CDCl_3$)

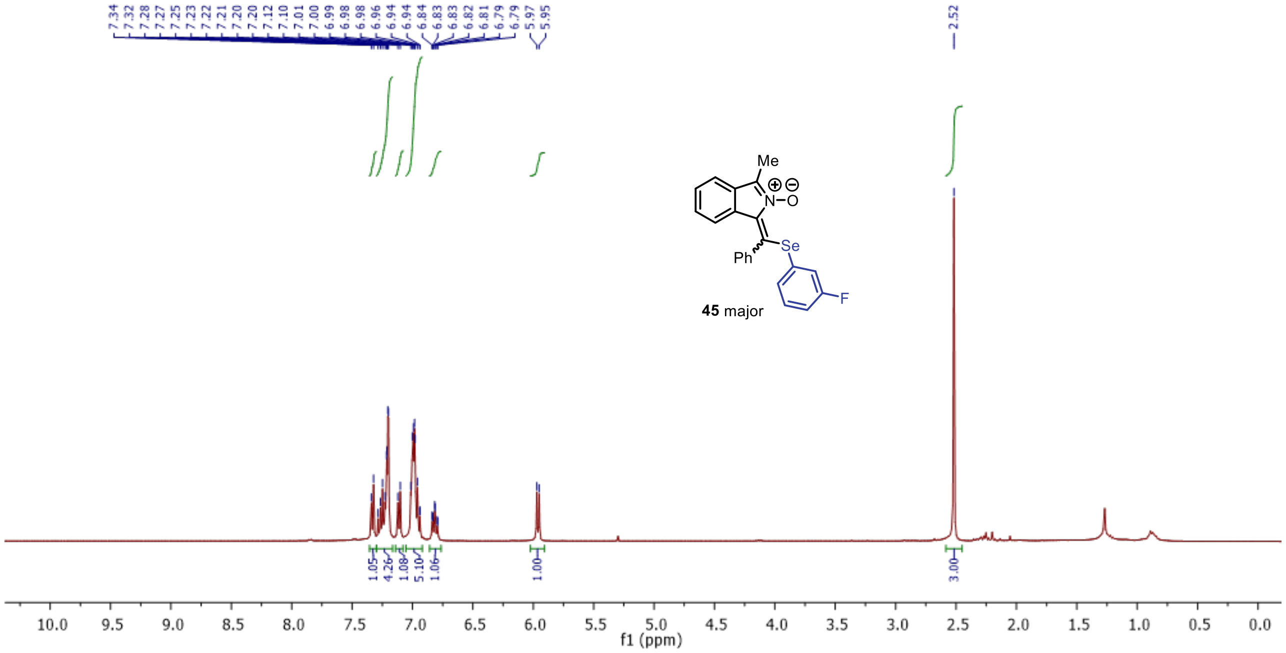

^{13}C NMR (101 MHz, $CDCl_3$)

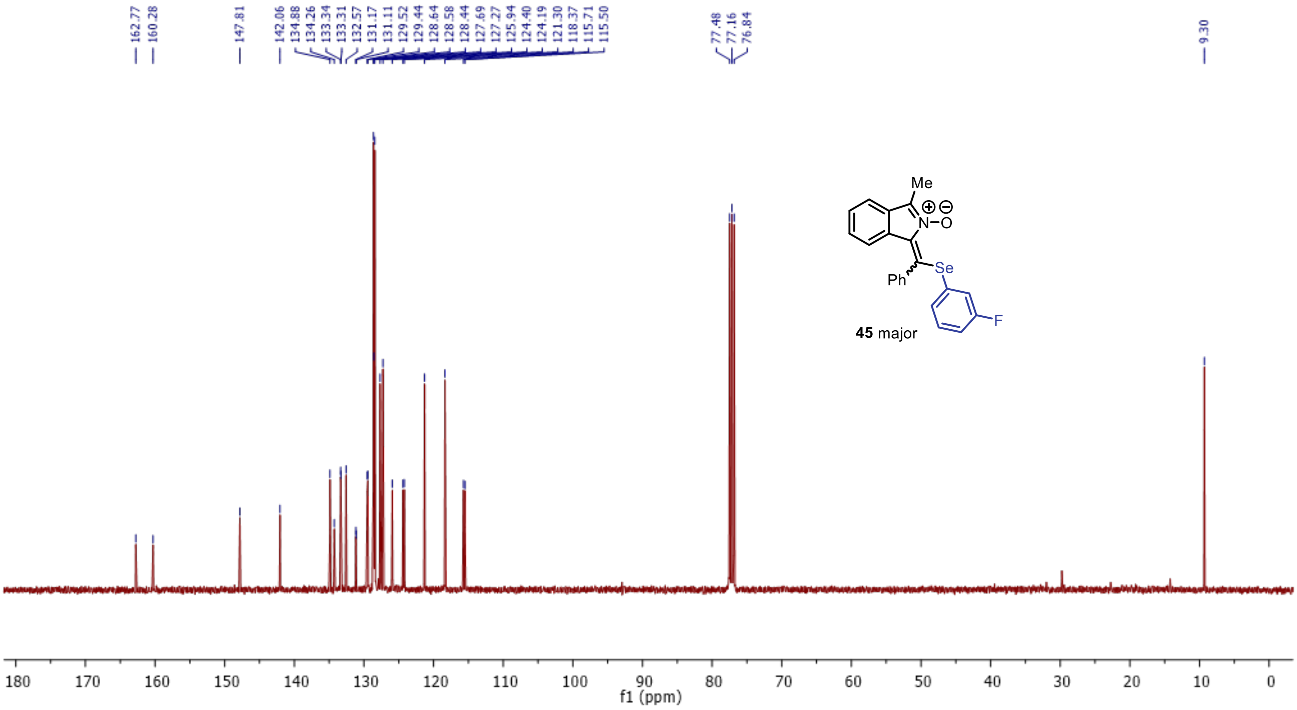

^{19}F NMR (376 MHz, $CDCl_3$)

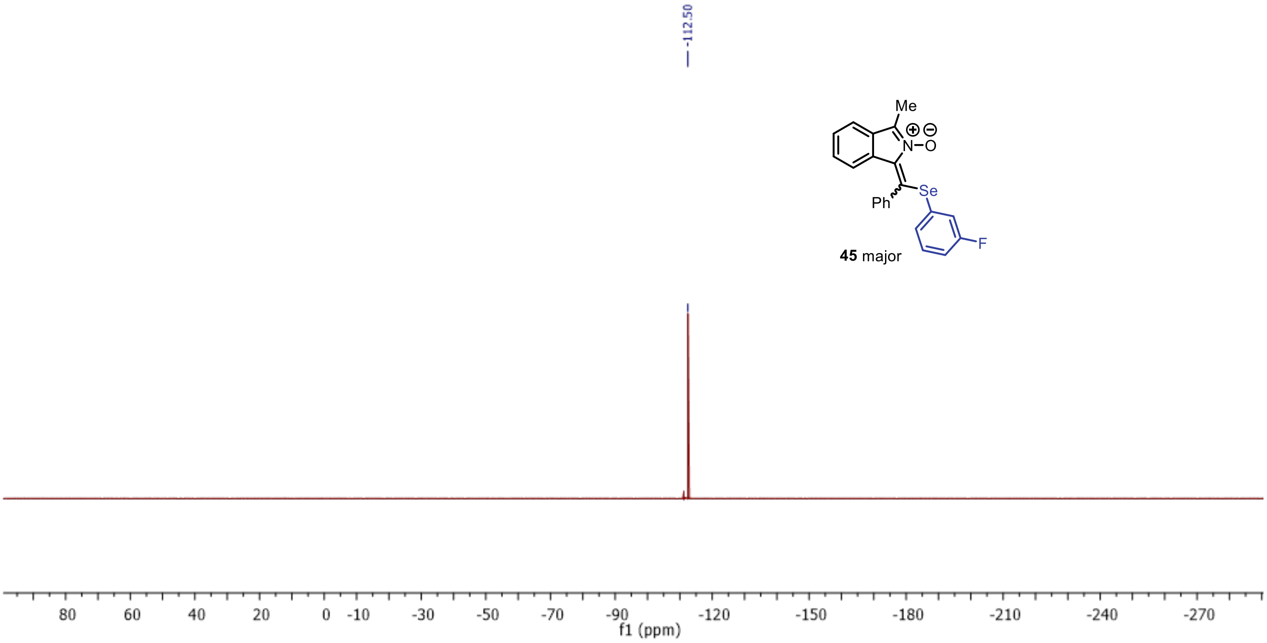

^{1}H NMR (400 MHz, $CDCl_3$)

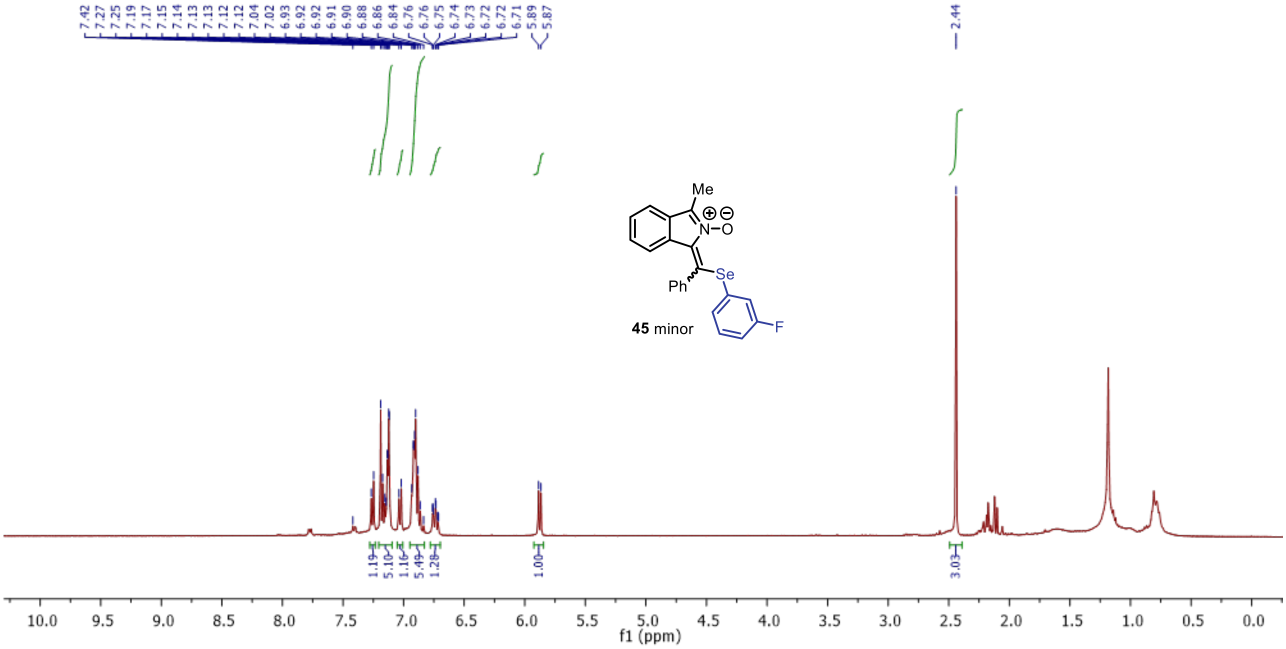

1-((4-Fluorophenyl)((3-fluorophenyl)selanyl)methylene)-3-methyl-1H-isoindole 2-oxide (46)

^{1}H NMR (400 MHz, $CDCl_3$)

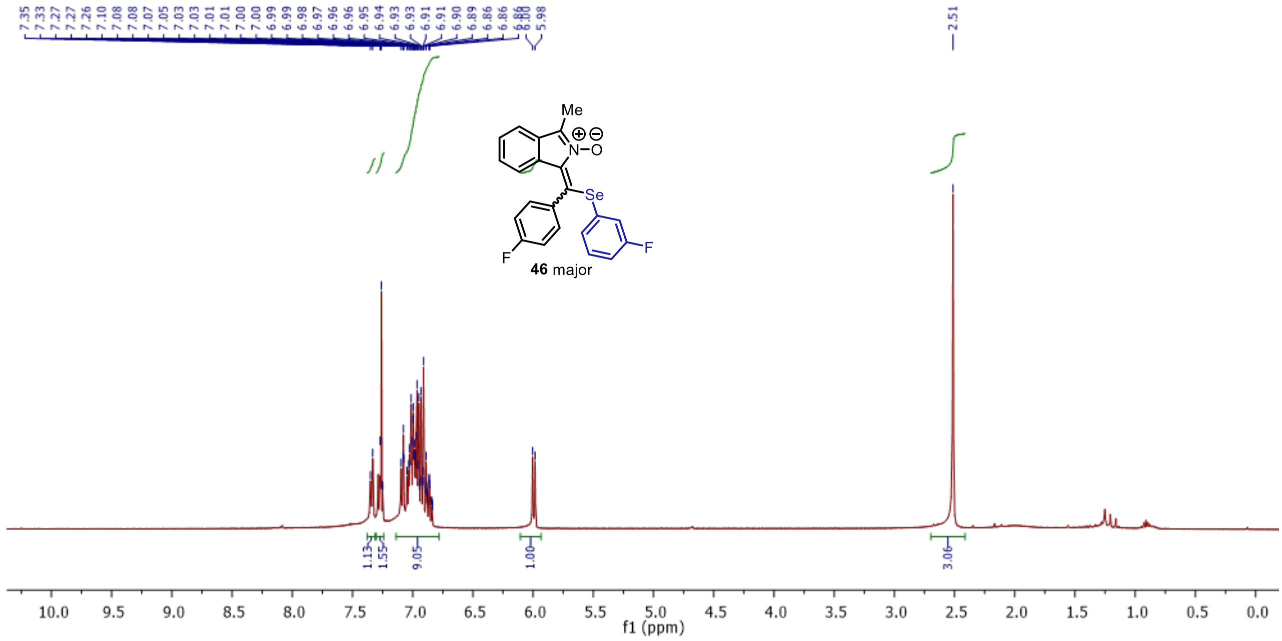

^{13}C NMR (101 MHz, $CDCl_3$)

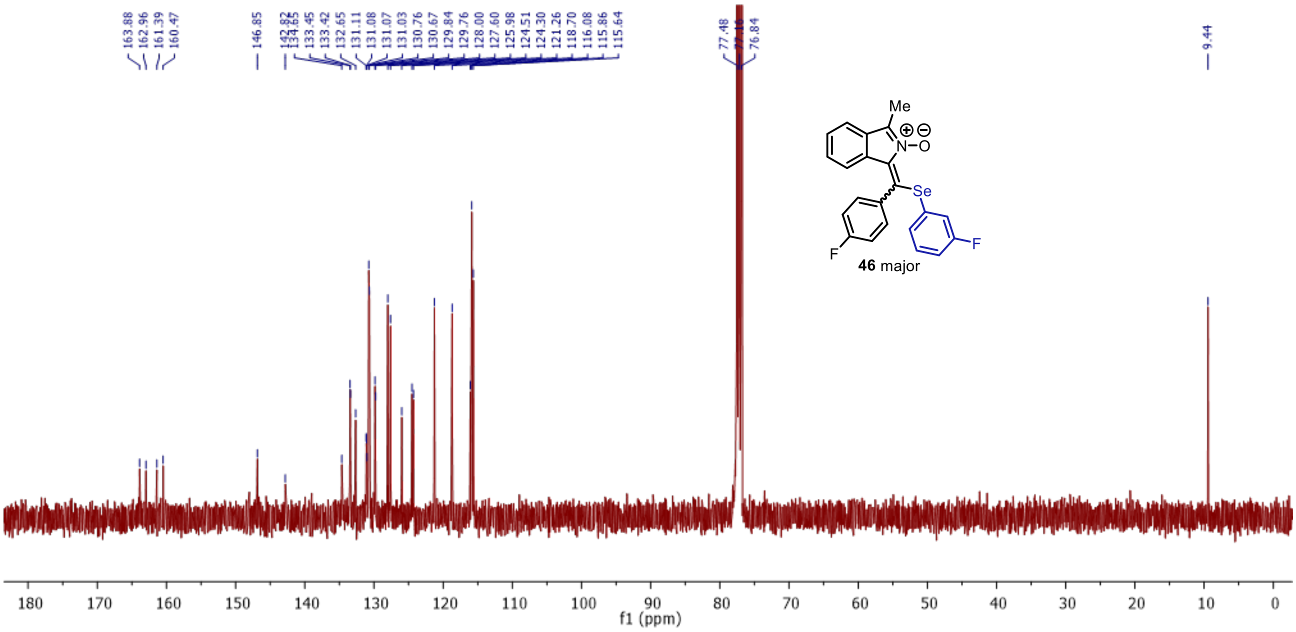

^{19}F NMR (376 MHz, $CDCl_3$)

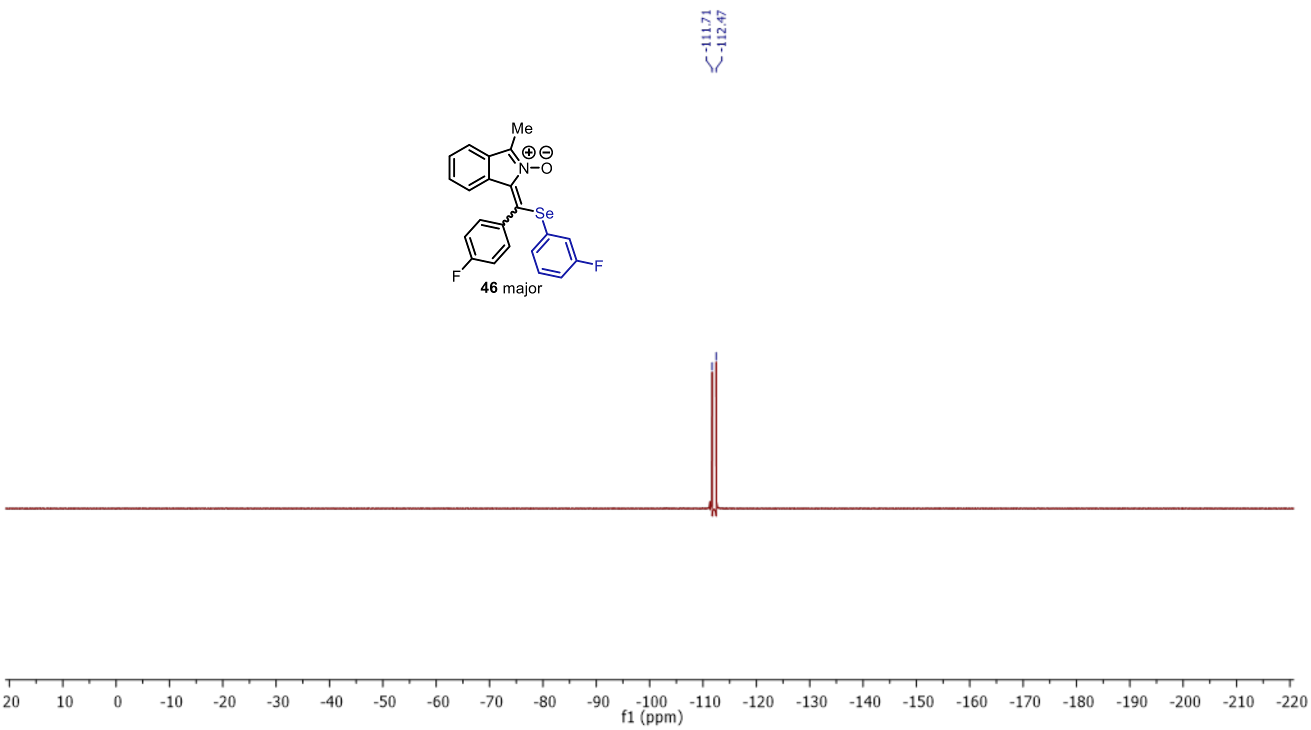

^{77}Se NMR (76 MHz, $CDCl_3$)

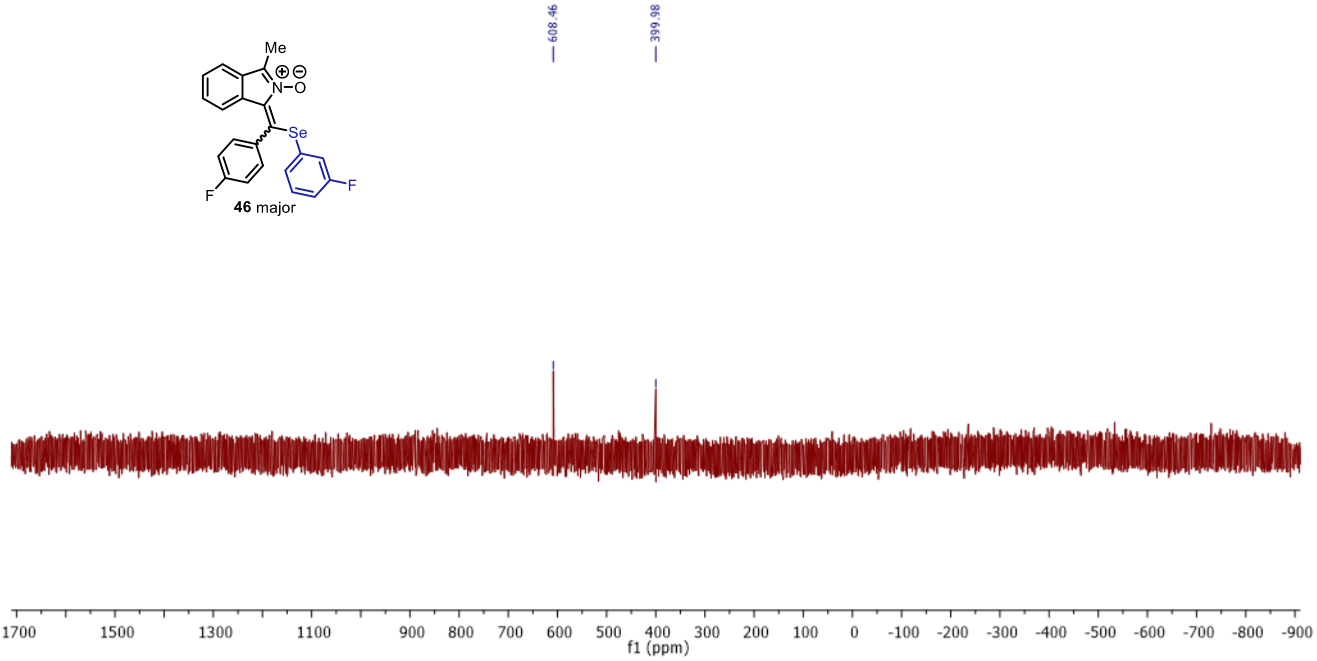

^{1}H NMR (400 MHz, $CDCl_3$)

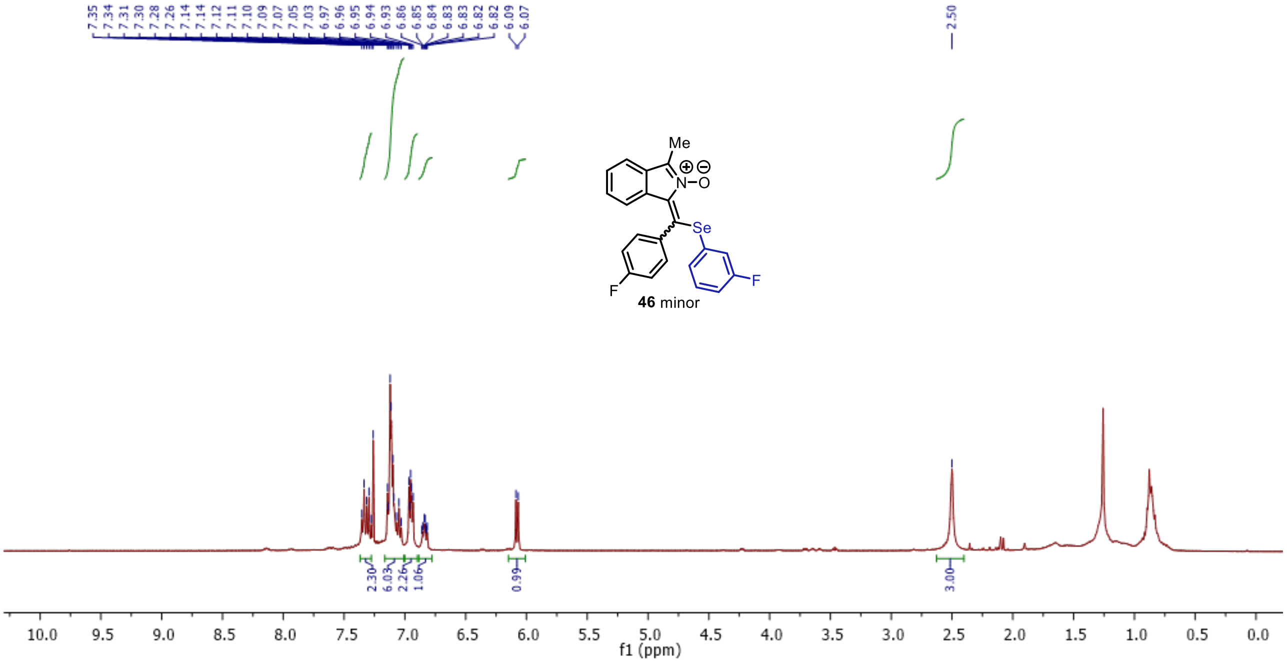

^{19}F NMR (376 MHz, $CDCl_3$

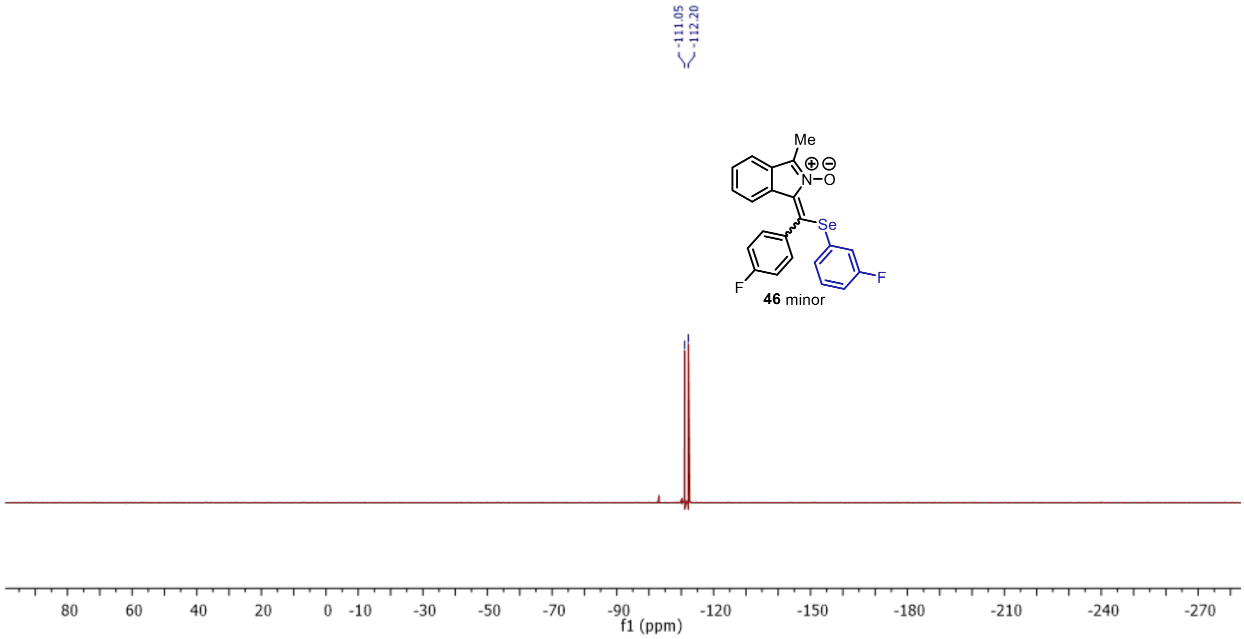

^{77}Se NMR (76 MHz, $CDCl_3$)

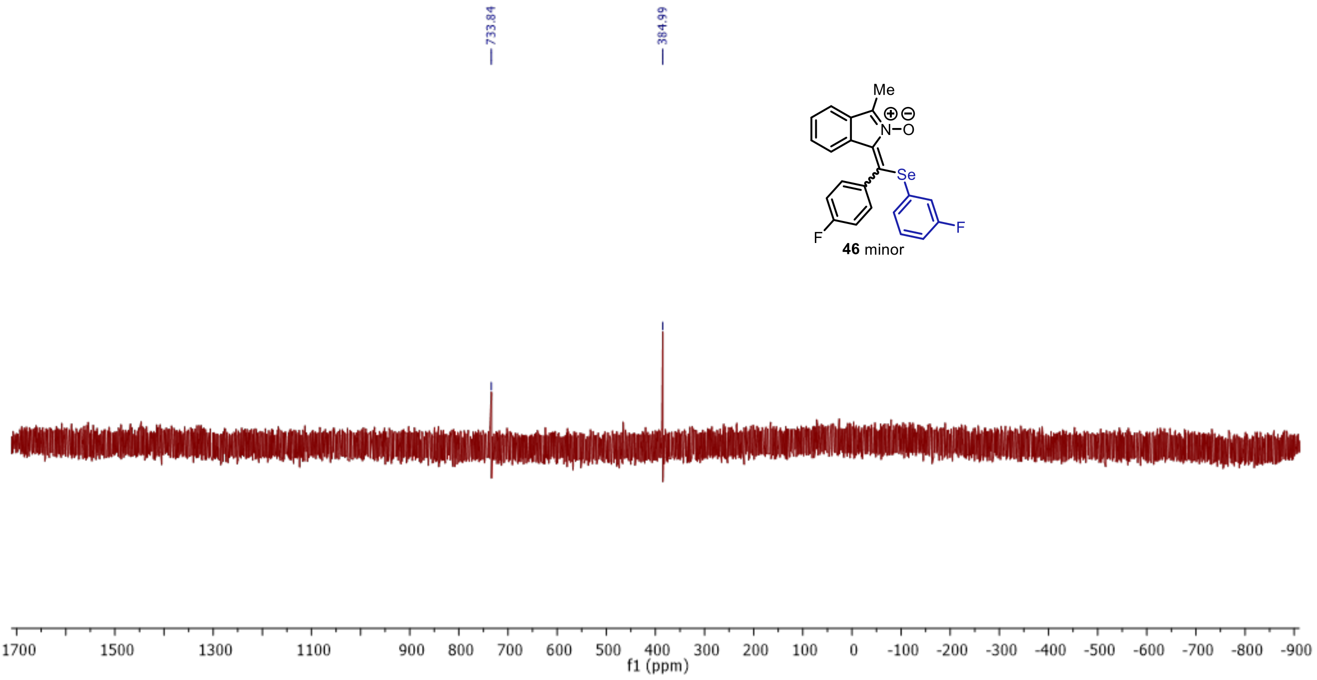

(*Z*)-5,6-Dimethoxy-3-methyl-1-(phenyl(phenylselanyl)methylene)-1H-isoindole 2-oxide (47):

^{1}H NMR (400 MHz, $CDCl_3$)

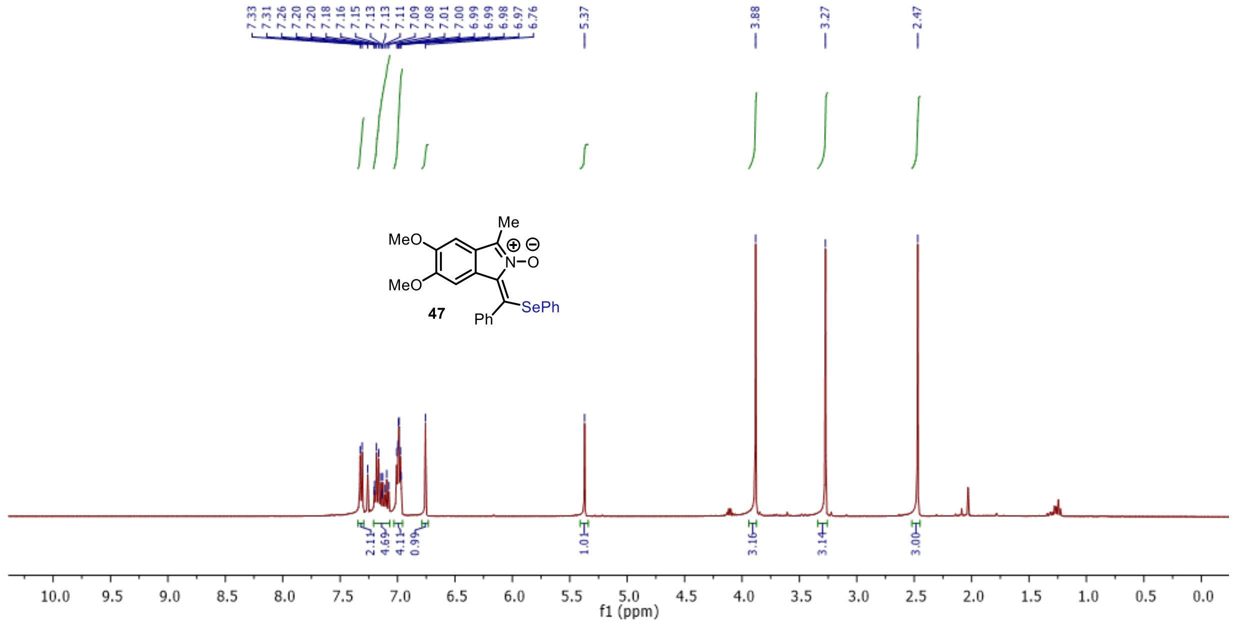

^{13}C NMR (101 MHz, $CDCl_3$)

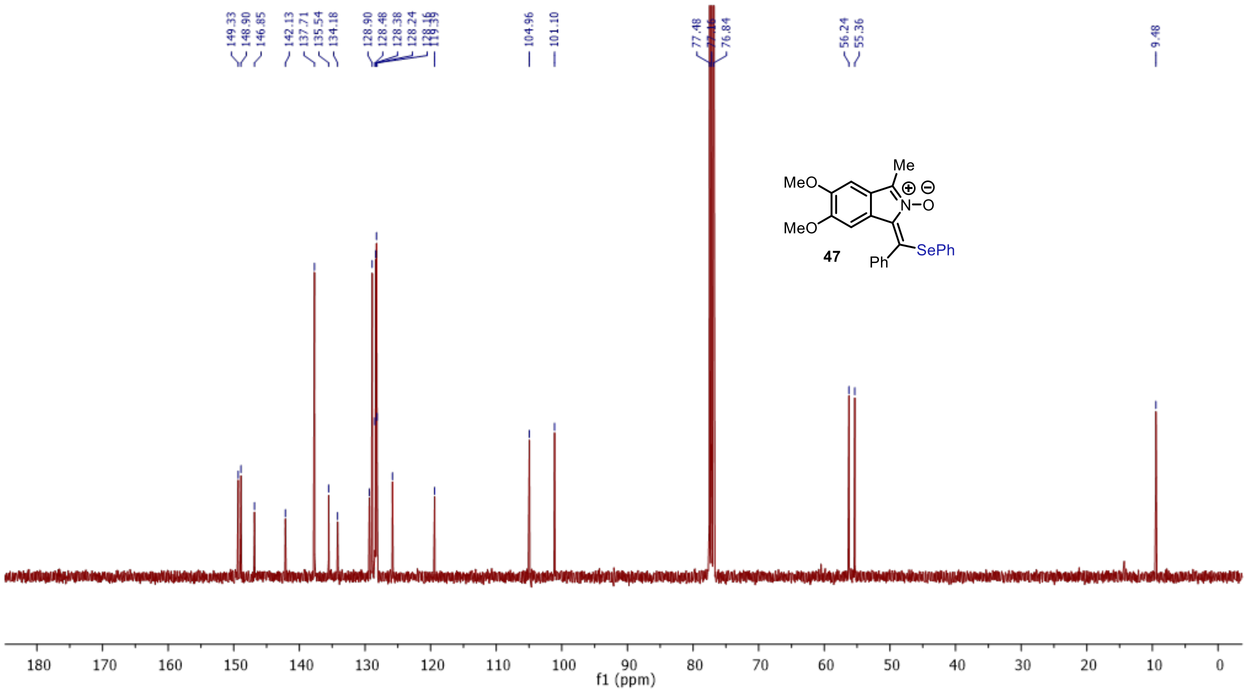

^{77}Se NMR (76 MHz, $CDCl_3$)

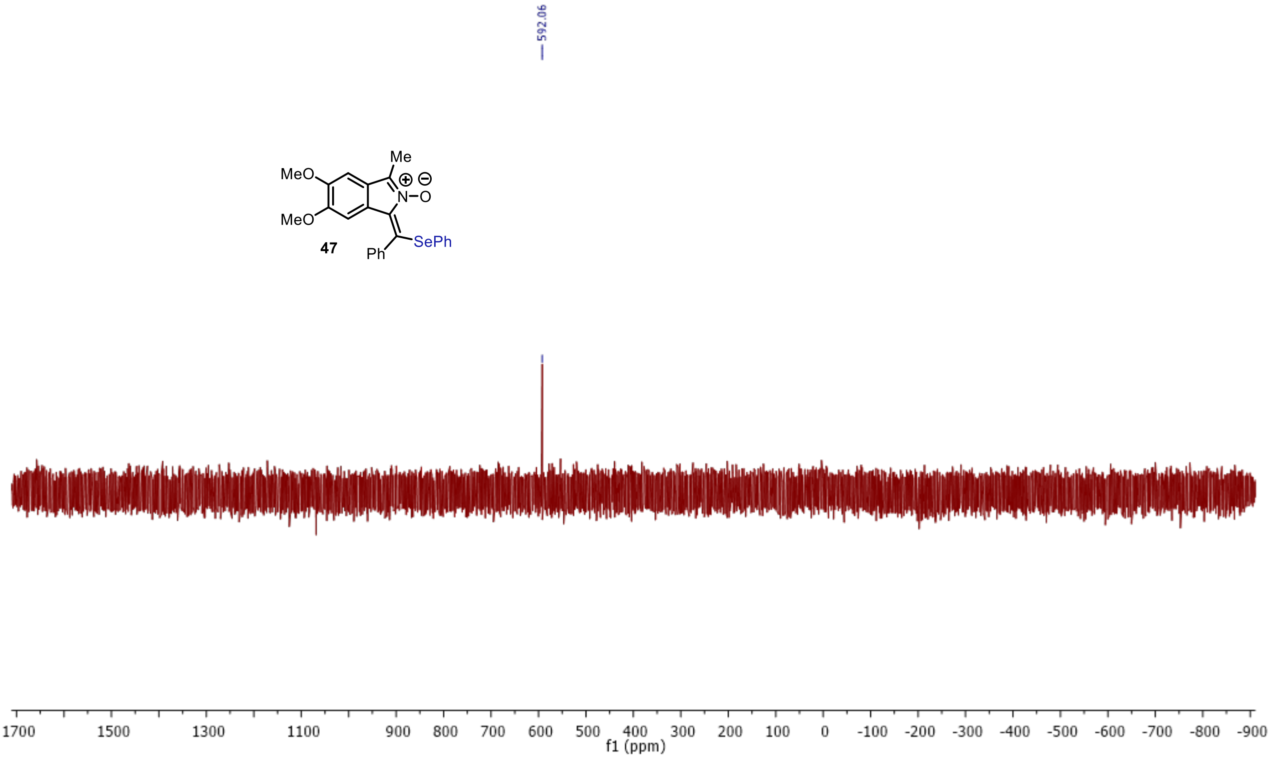

(*Z*)-5-methyl-7-(phenyl(phenylselanyl)methylene)-7H-pyrrolo[3,4-b]pyridine 6-oxide (48)

^{1}H NMR (400 MHz, $CDCl_3$)

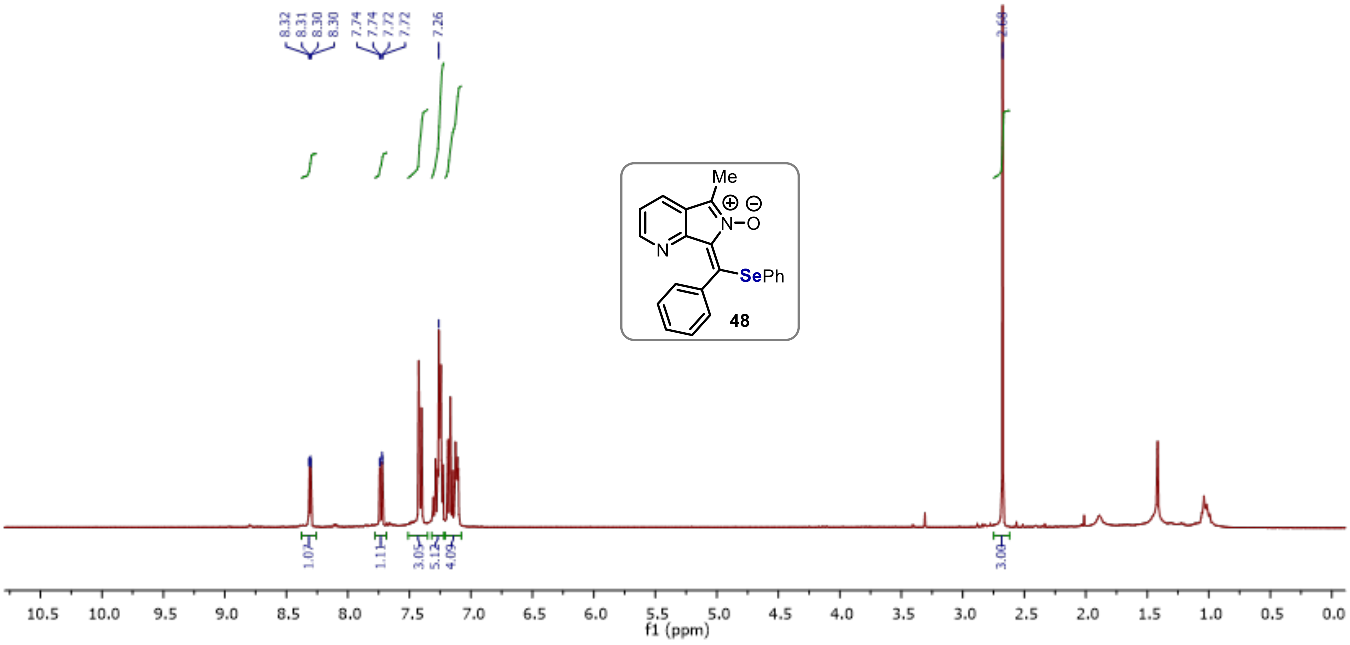

^{13}C NMR (101 MHz, $CDCl_3$)

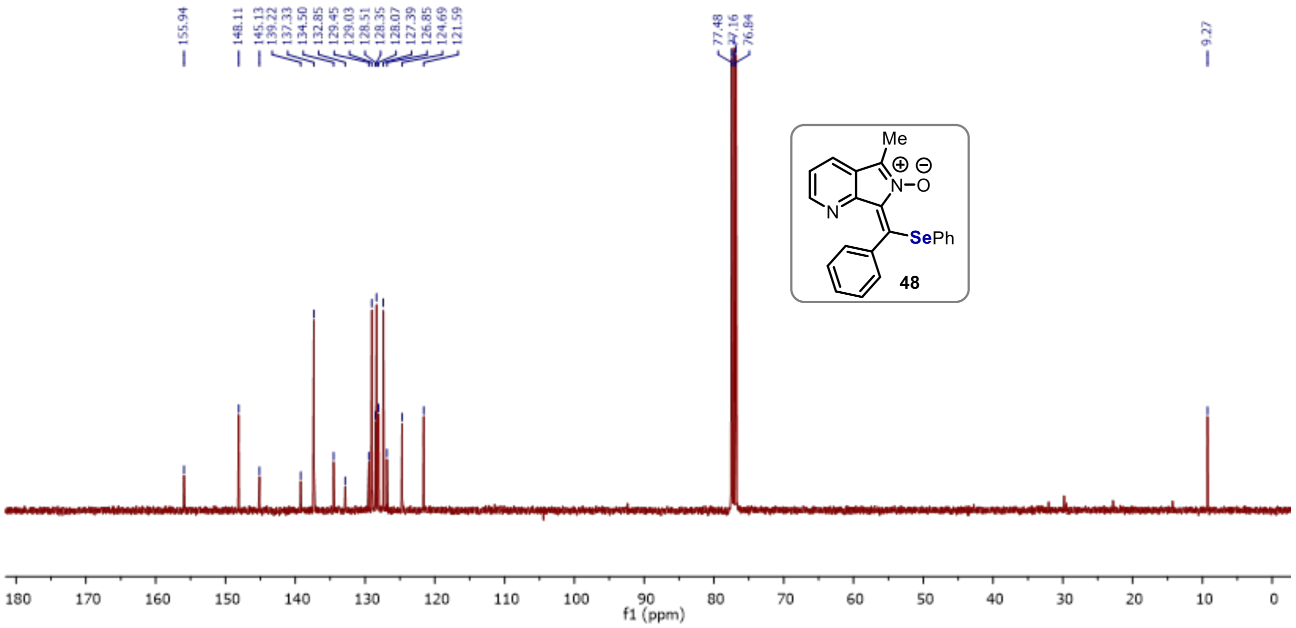

^{77}Se NMR (76 MHz, $CDCl_3$)

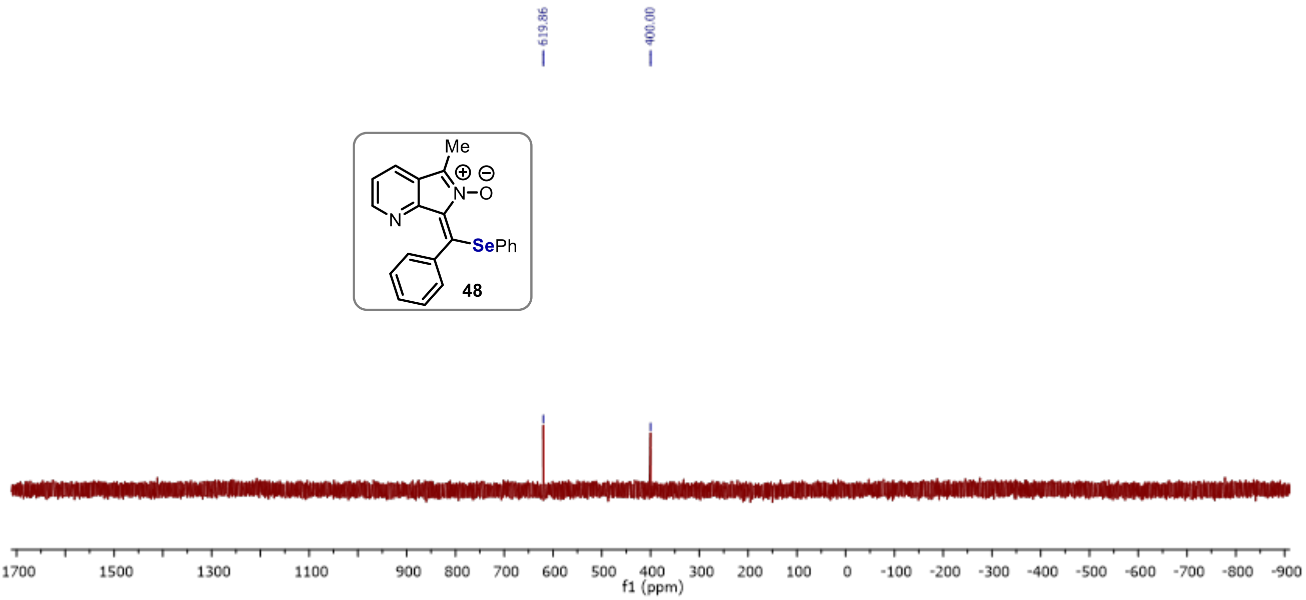

_N_,3-Diphenyl-4-(phenylselanyl)isoquinolin-1-amine (50):

^{1}H NMR (400 MHz, $CDCl_3$)

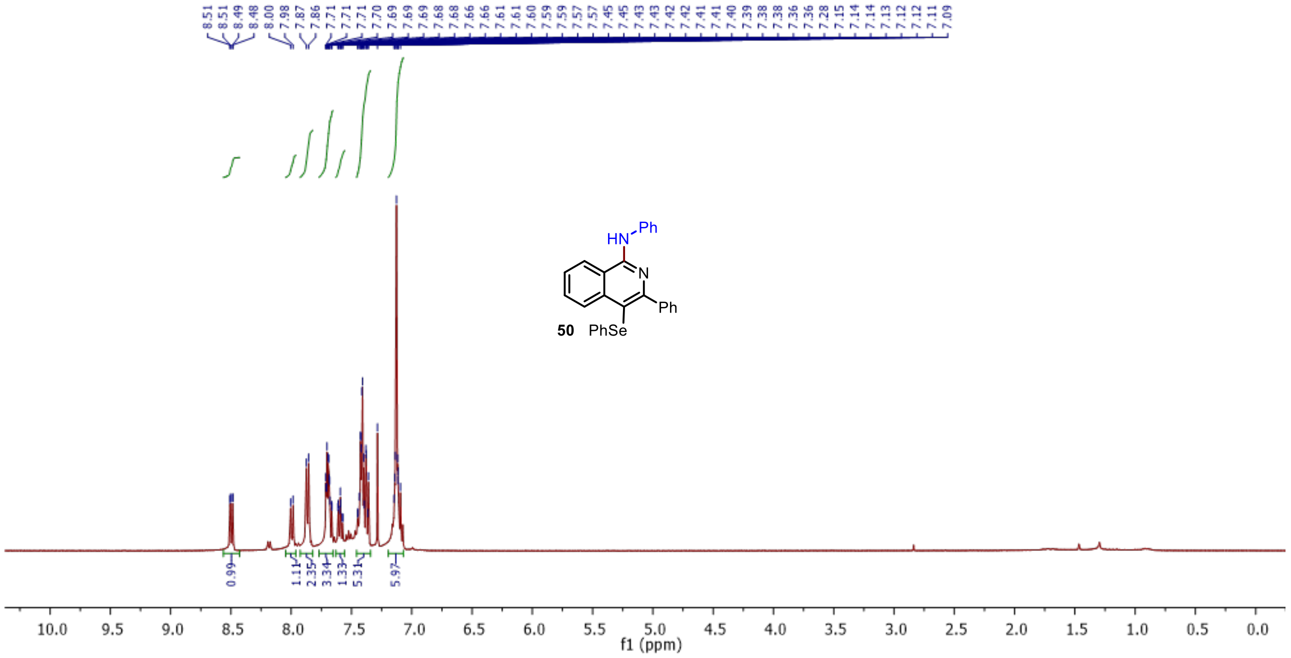

^{13}C NMR (101 MHz, $CDCl_3$)

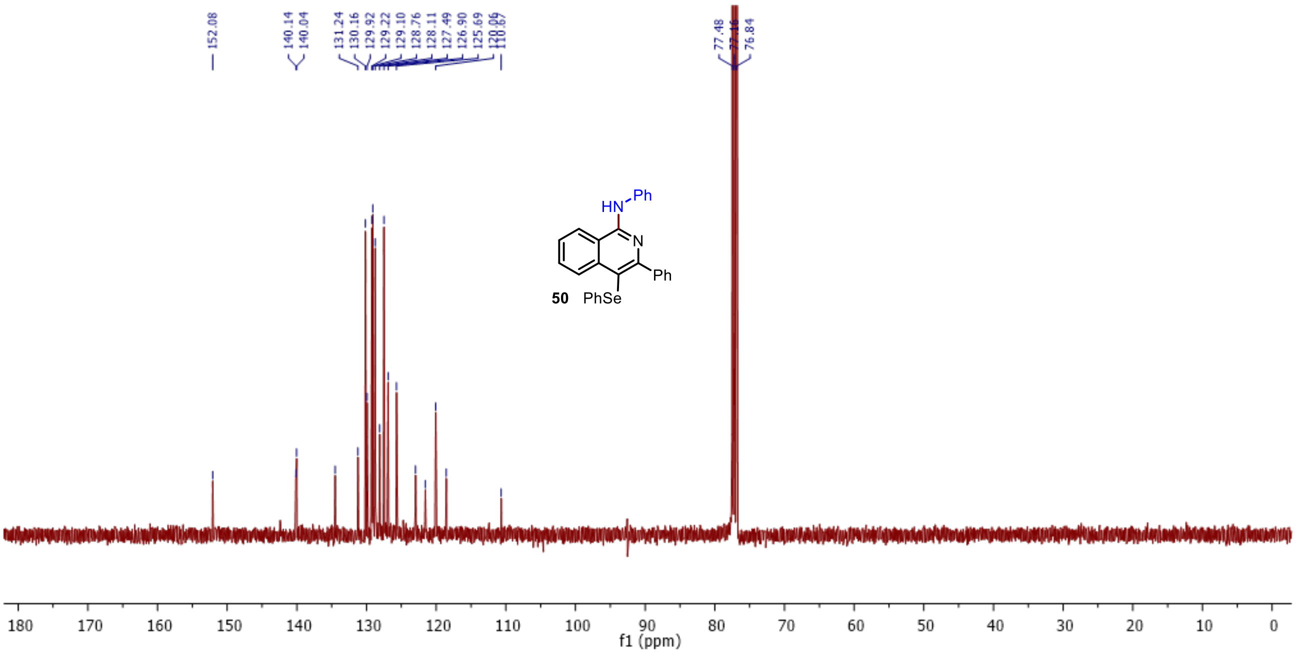

^{77}Se NMR (76 MHz, $CDCl_3$)

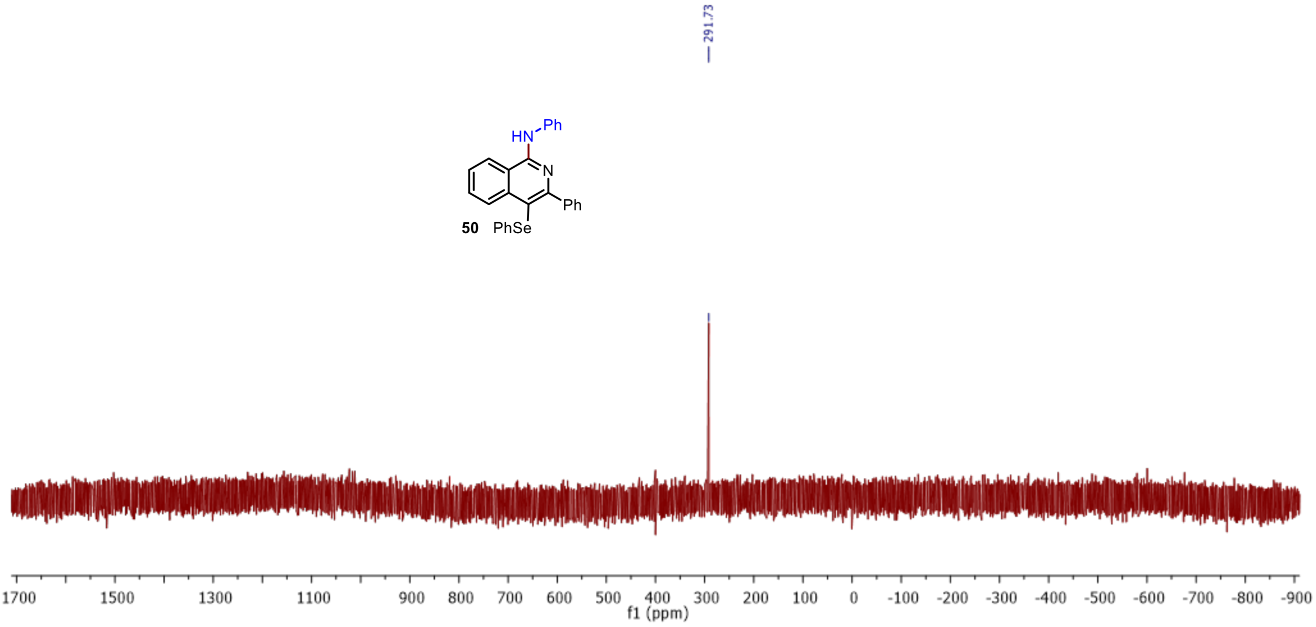

(*E*)-3-Phenyl-4-(phenylselanyl)-1-styrylisoquinoline (51)

^{1}H NMR (400 MHz, $CDCl_3$)

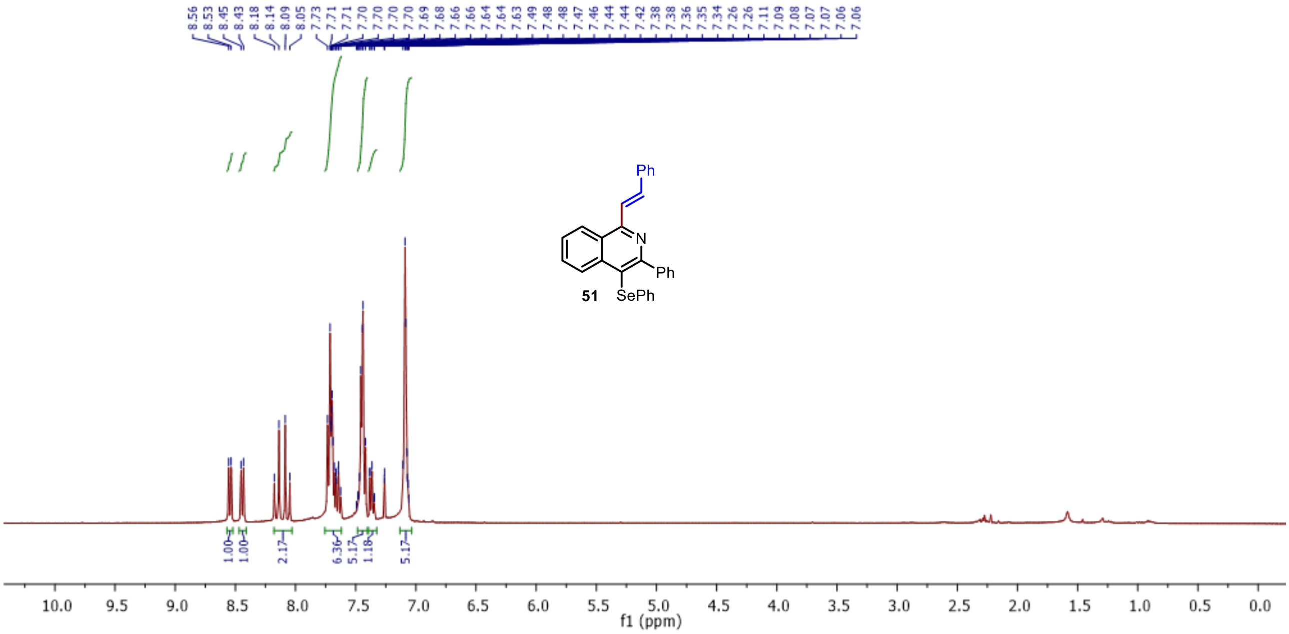

^{13}C NMR (101 MHz, $CDCl_3$)

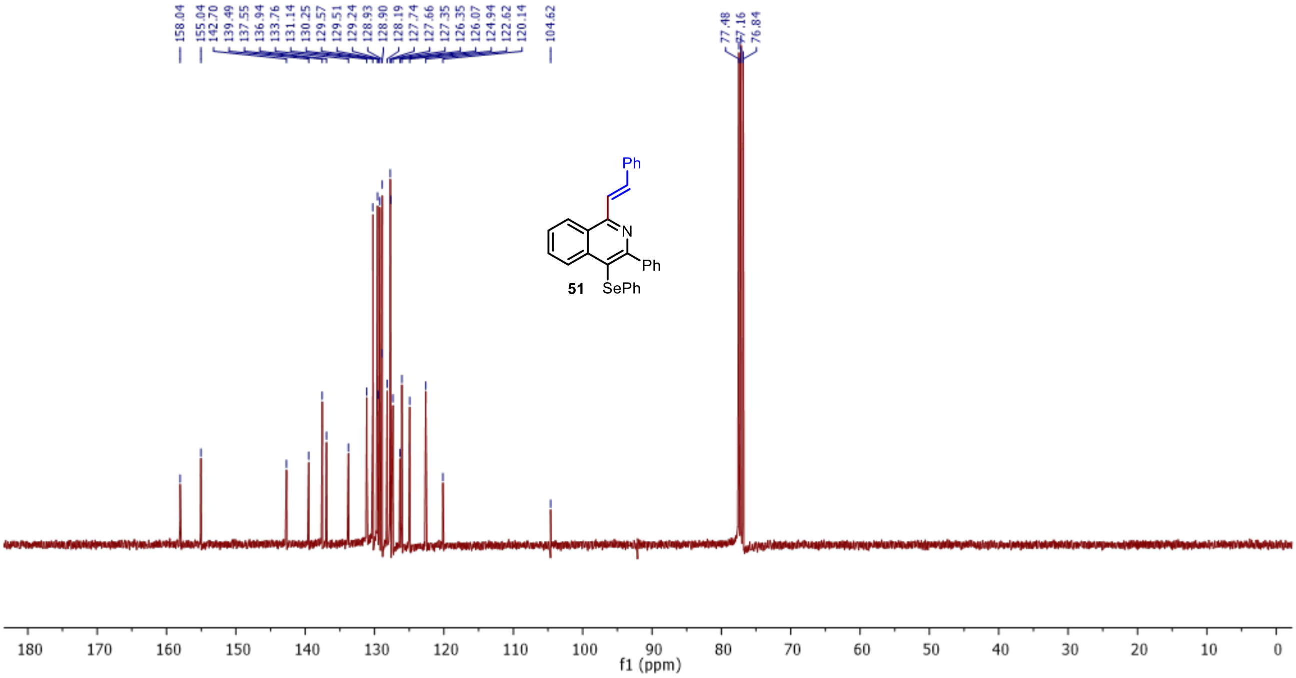

^{77}Se NMR (76 MHz, $CDCl_3$)

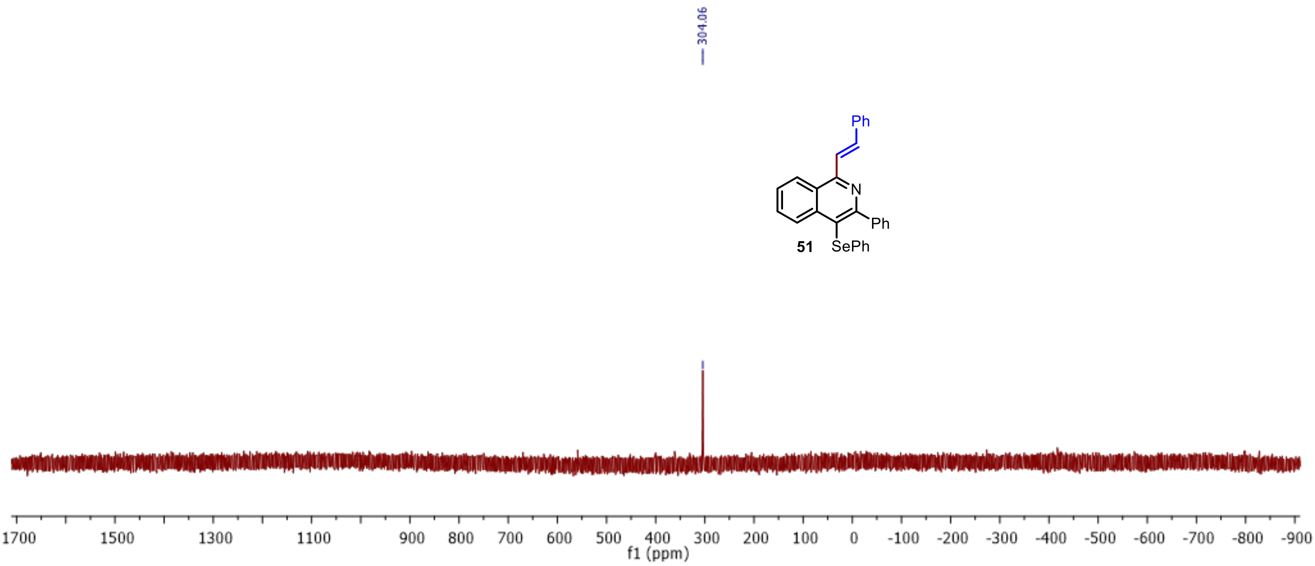

Diphenyl(3-phenyl-4-(phenylselanyl)isoquinolin-1-yl)phosphine oxide (52)

^{1}H NMR (400 MHz, $CDCl_3$)

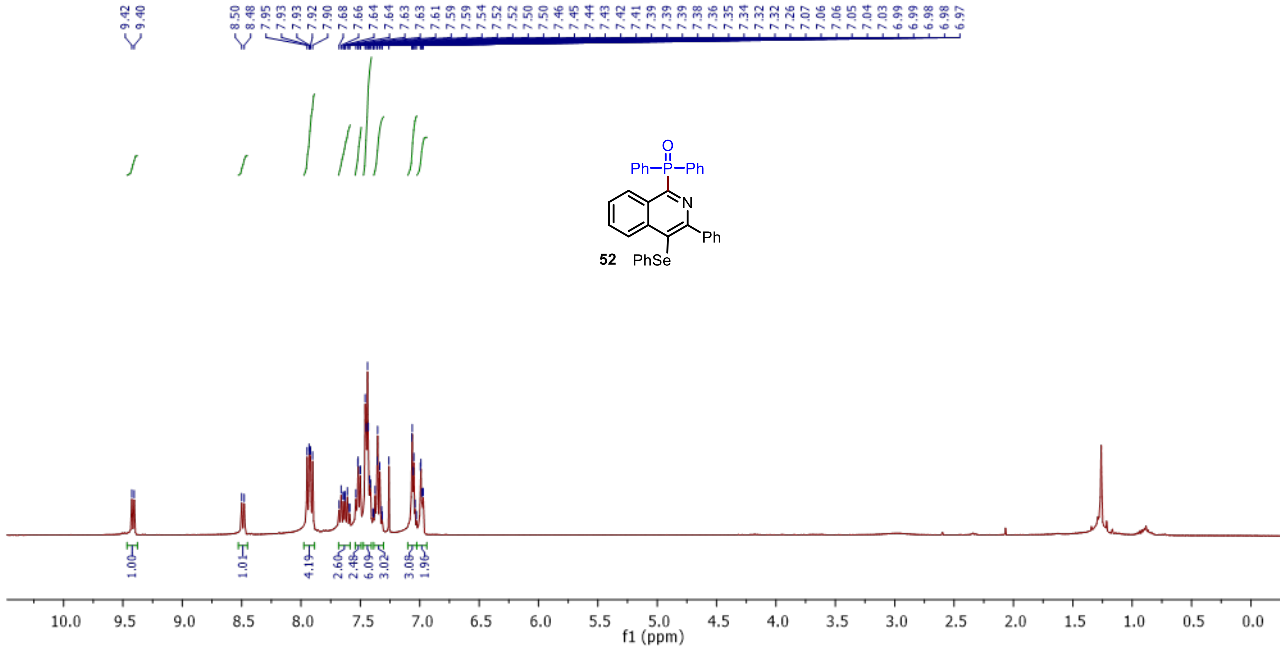

^{13}C NMR (101 MHz, $CDCl_3$)

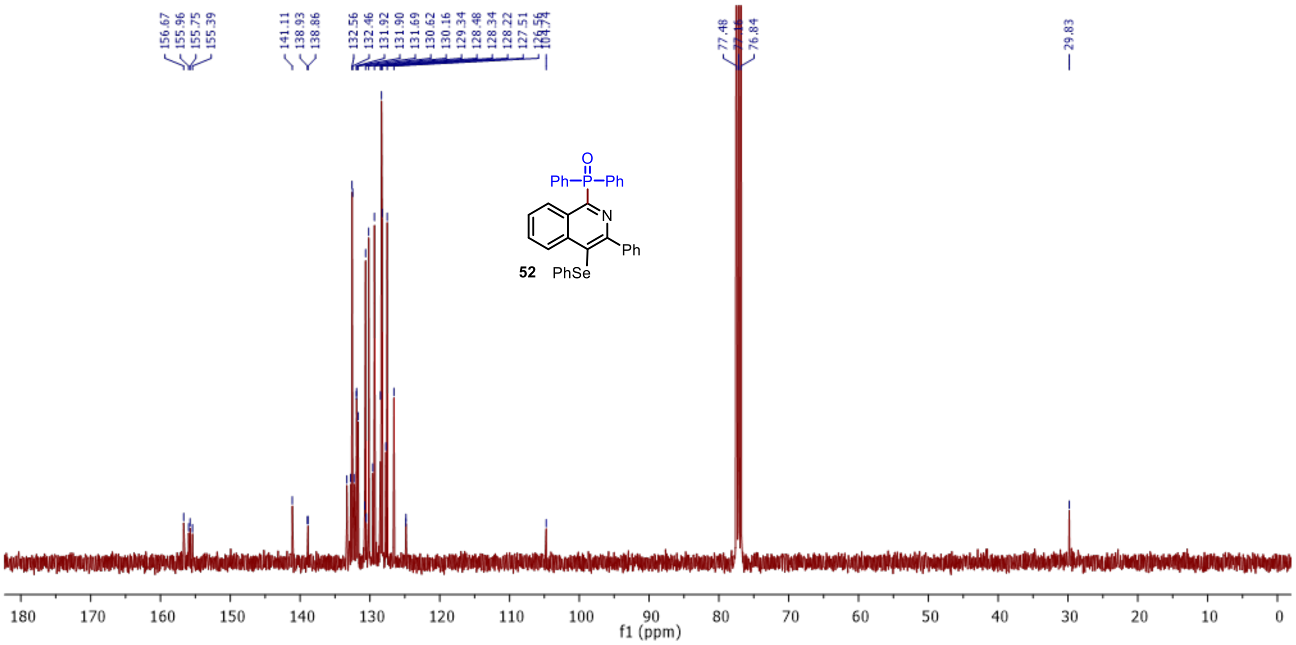

^{31}P NMR (162 MHz, $CDCl_3$)

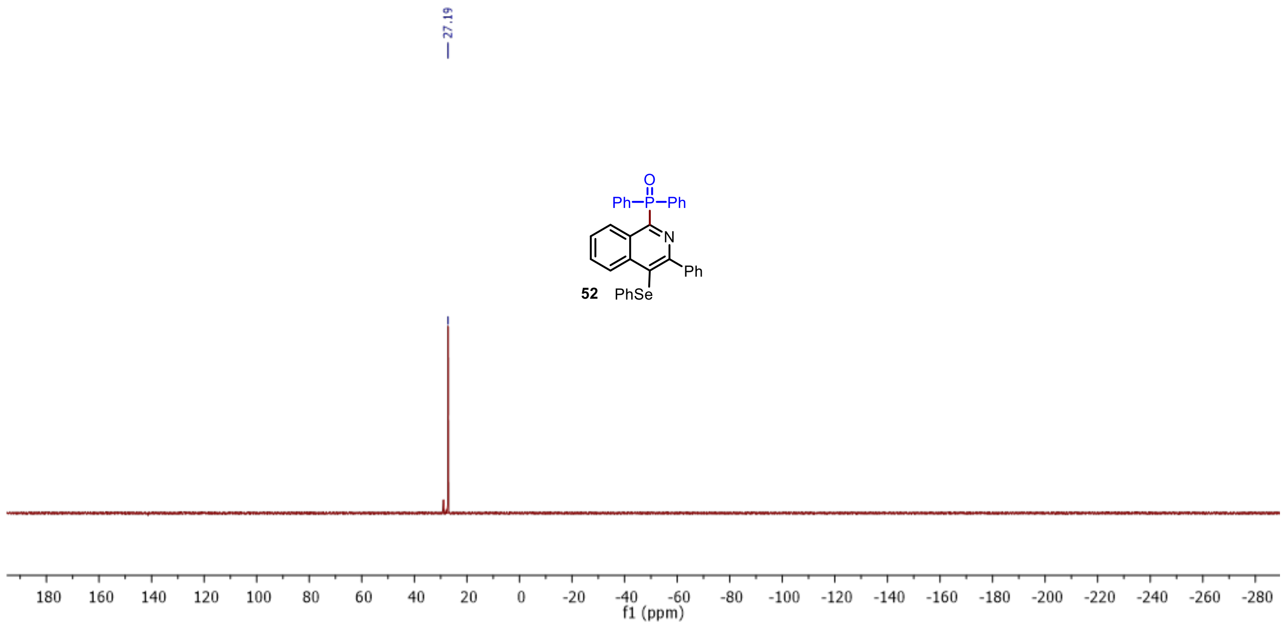

^{77}Se NMR (76 MHz, $CDCl_3$)

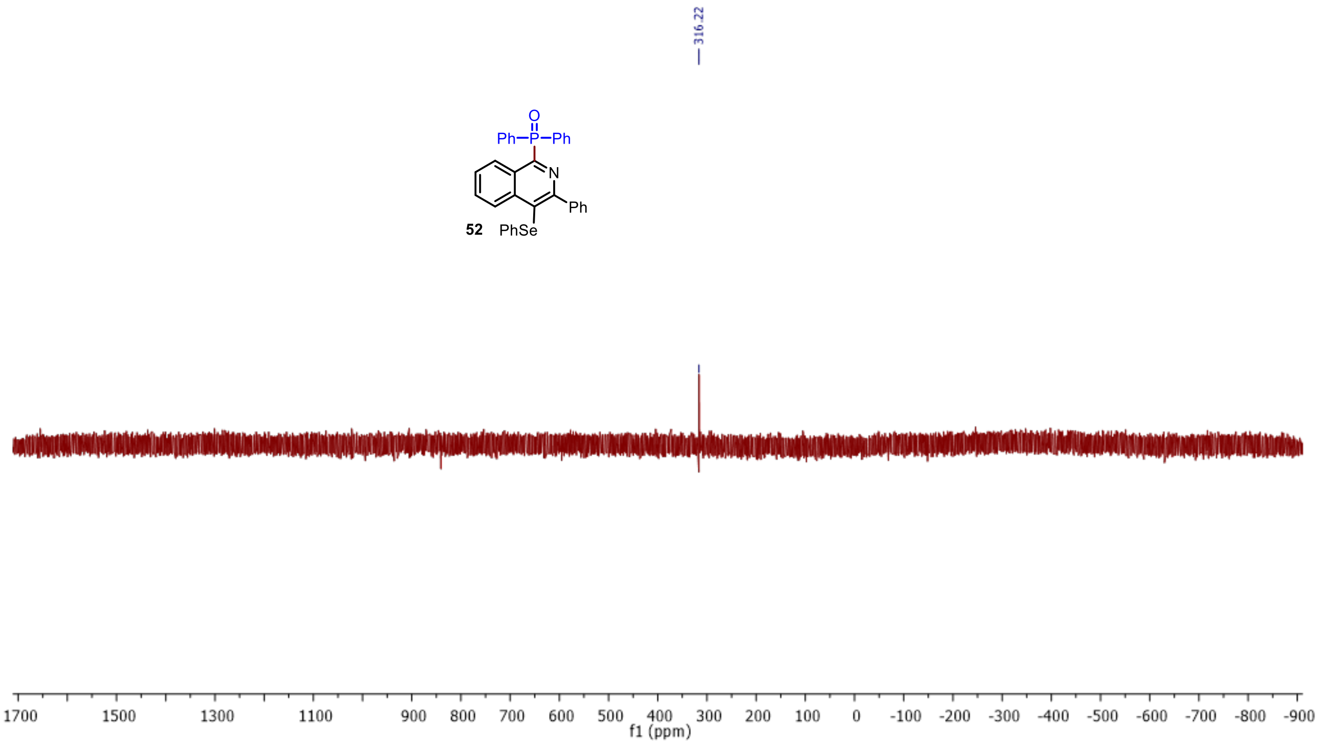

2-(3-Phenyl-4-(phenylselanyl)isoquinolin-1-yl)phenol (53):

^{1}H NMR (400 MHz, $CDCl_3$)

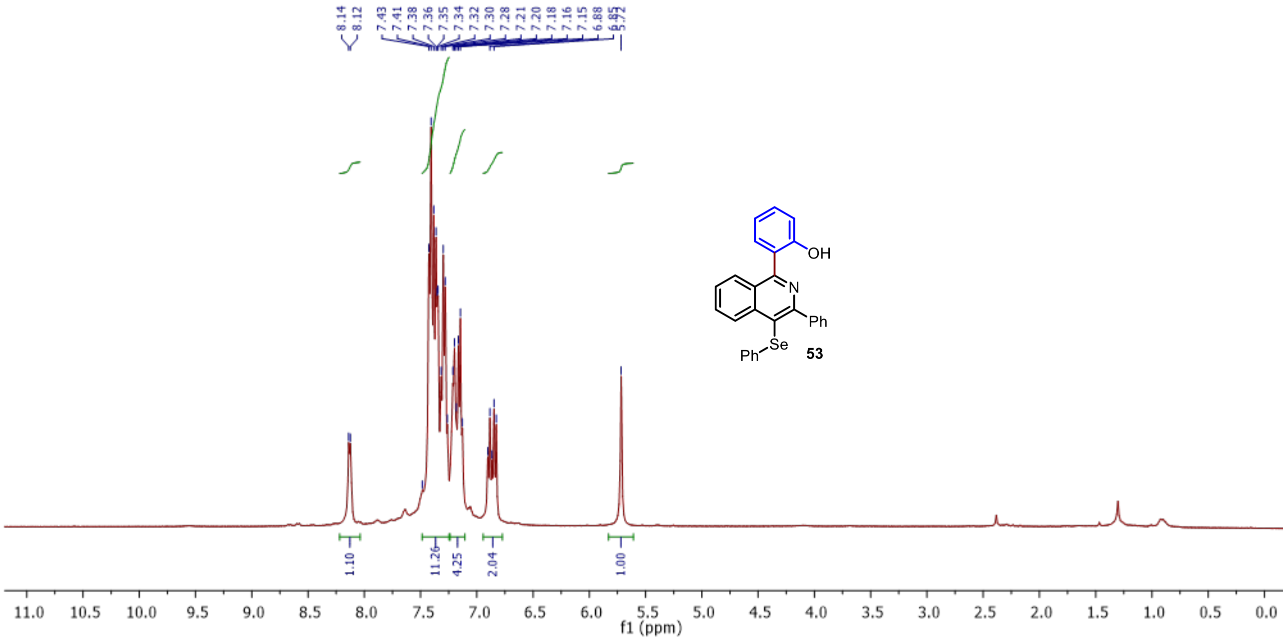

^{13}C NMR (101 MHz, $CDCl_3$)

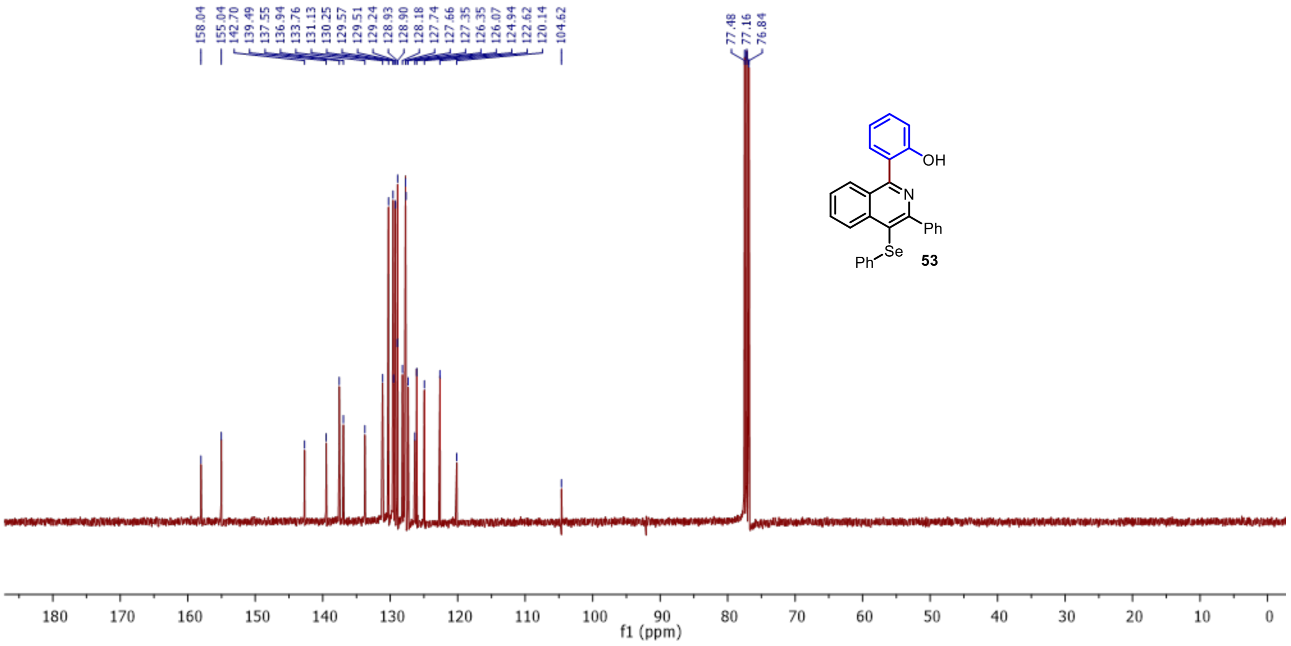

^{77}Se NMR (76 MHz, $CDCl_3$)

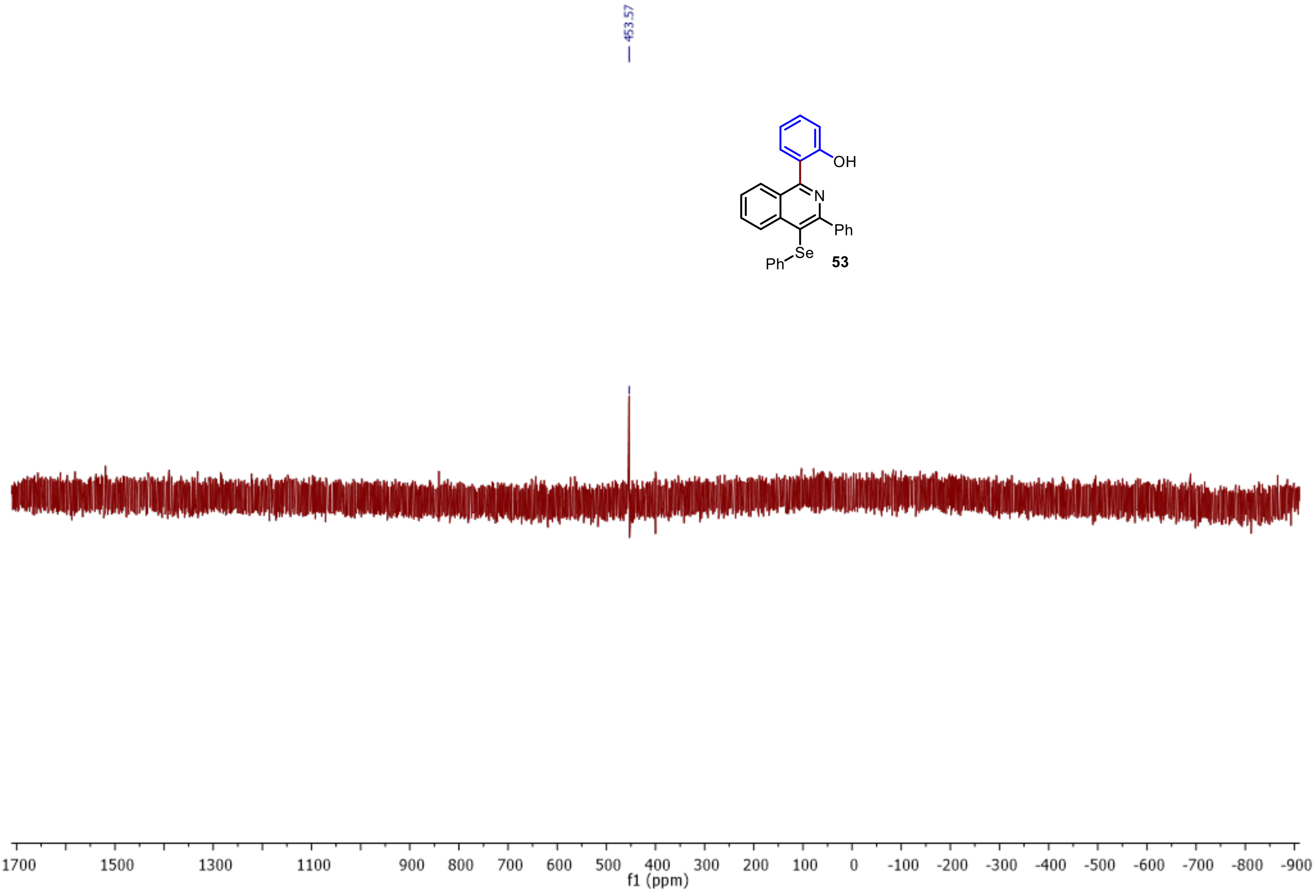

6-Phenyl-5-(phenylselanyl)indazolo[3,2-a]isoquinoline (54)

^{1}H NMR (400 MHz, $CDCl_3$)

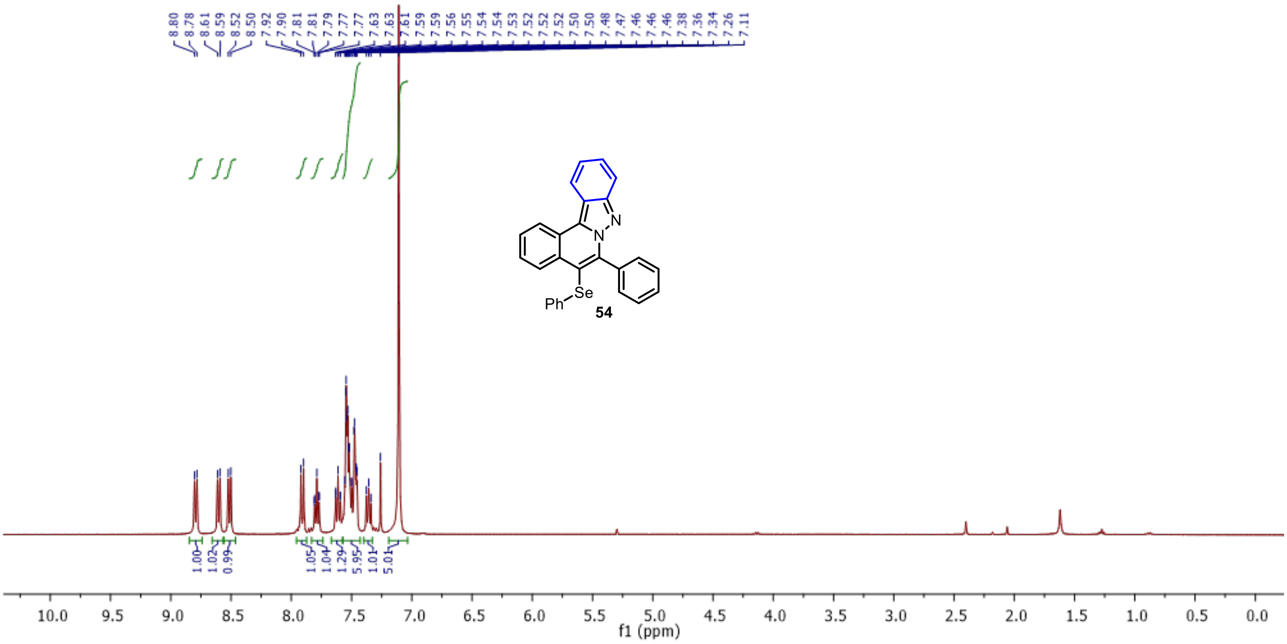

^{13}C NMR (101 MHz, $CDCl_3$)

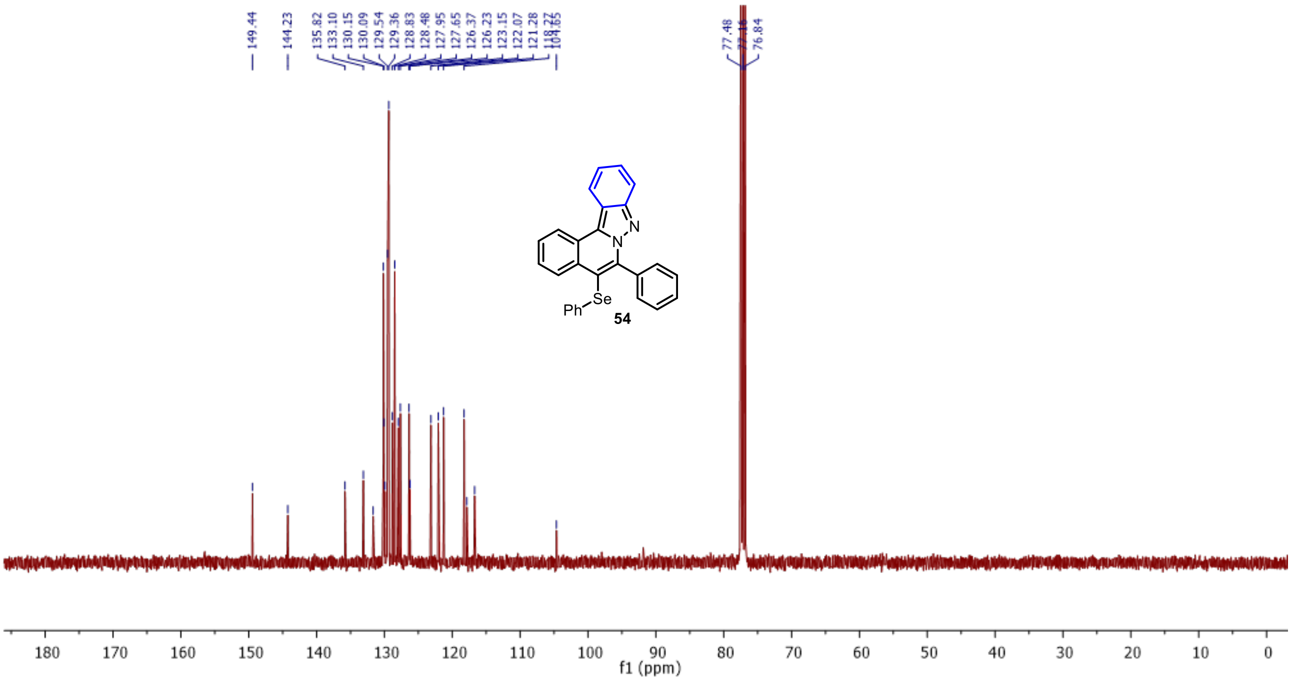

^{77}Se NMR (76 MHz, $CDCl_3$)

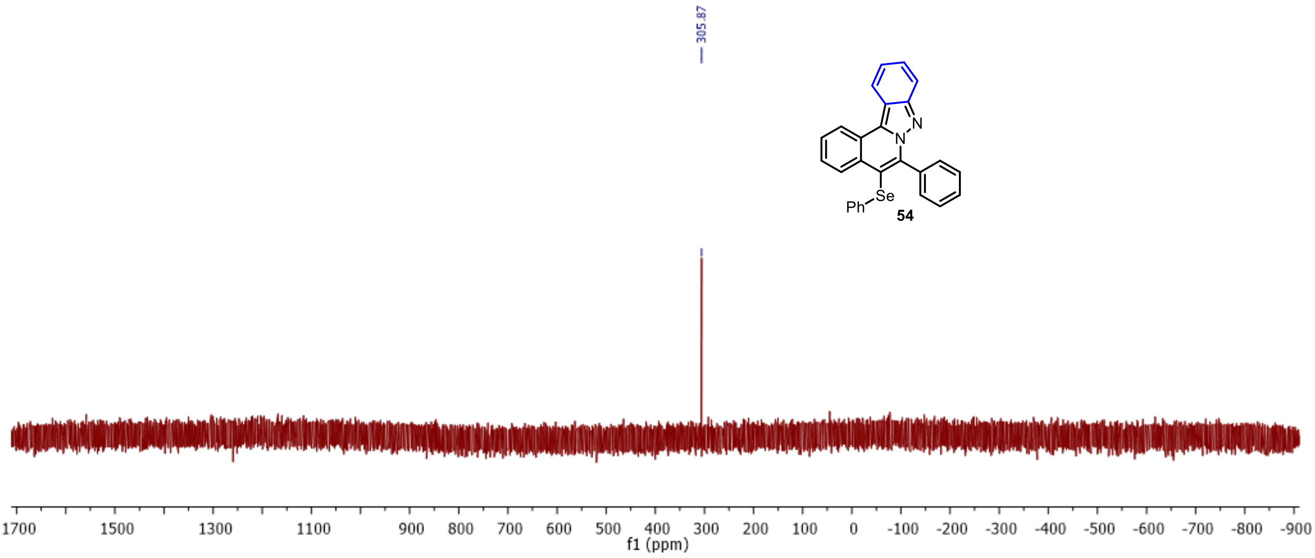

2-(4-Methoxyphenyl)-5-phenyl-6-(phenylselanyl)pyrazolo[5,1-a]isoquinoline (55):

^{1}H NMR (400 MHz, $CDCl_3$)

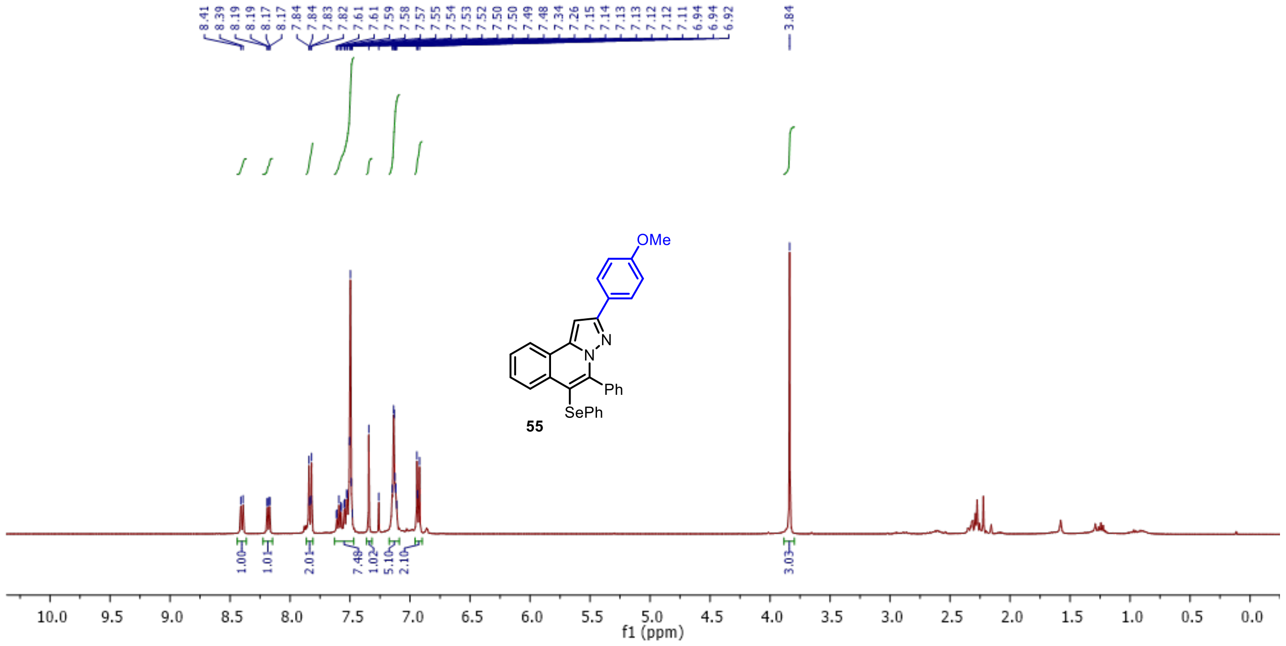

^{13}C NMR (101 MHz, $CDCl_3$)

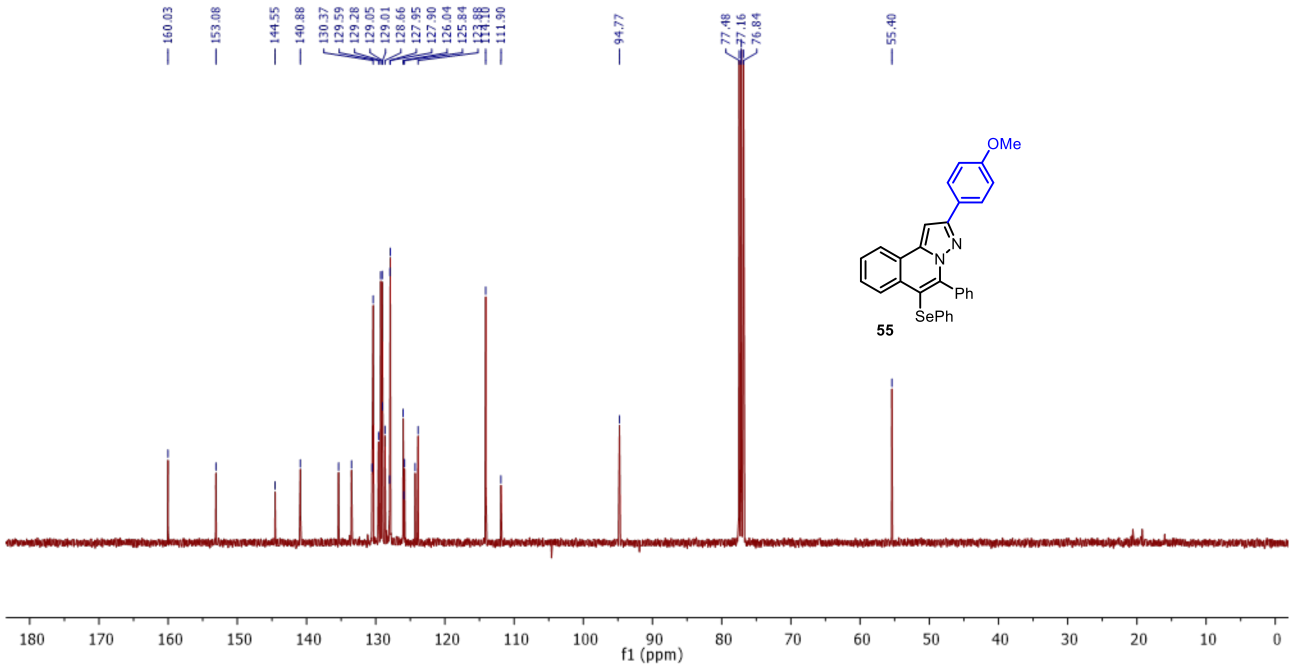

^{77}Se NMR (76 MHz, $CDCl_3$)

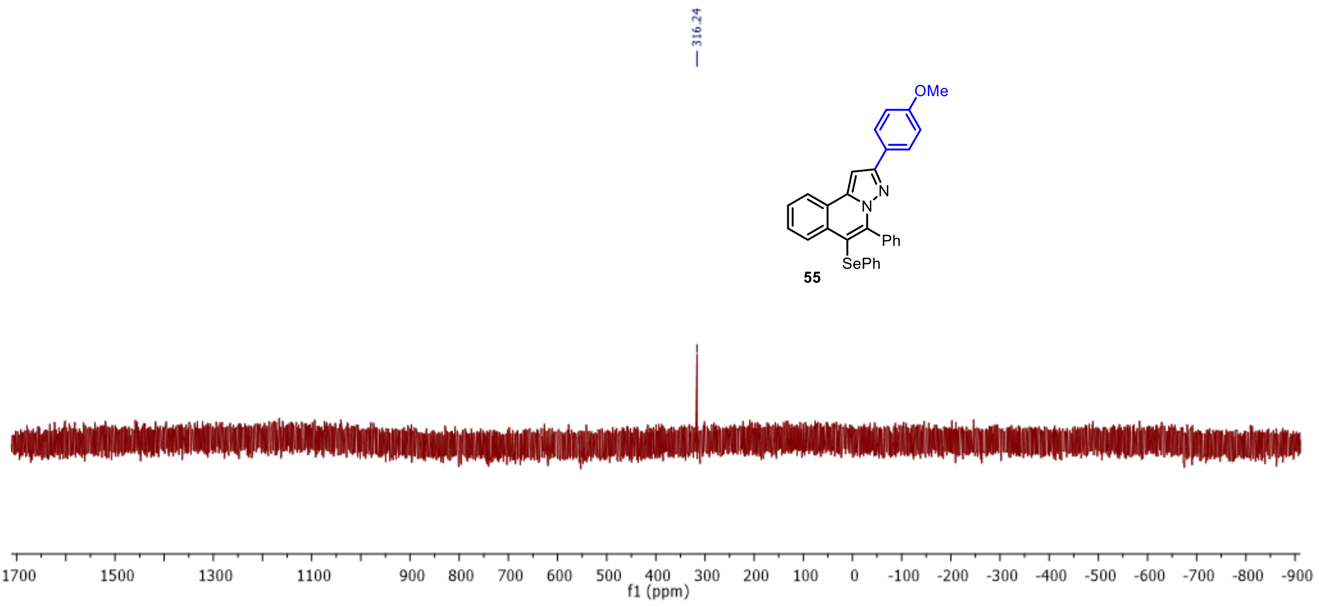

Supplement: Supplementary file 1 — Supporting Information [file ANIE-64-e202509811-s001.pdf]
